# Childhood predictors of charitable giving and helping across 22 countries in the Global Flourishing Study

**DOI:** 10.1038/s41598-024-77950-1

**Published:** 2025-04-30

**Authors:** Julia S. Nakamura, Robert D. Woodberry, Dorota Weziak-Bialowolska, Laura D. Kubzansky, Koichiro Shiba, R. Noah Padgett, Byron R. Johnson, Tyler J. VanderWeele

**Affiliations:** 1 Department of Psychology, University of British Columbia, 2136 West Mall, Vancouver, BC V6T 1Z4 Canada; 2 Institute for Studies of Religion, Baylor University, Waco, TX USA; 3 Department of Quantitative Methods and Information Technology, Kozminski University, Warsaw, Poland; 4 Human Flourishing Program, Institute for Quantitative Social Science, Harvard University, Cambridge, MA USA; 5 Department of Social and Behavioral Sciences, Harvard T.H. Chan School of Public Health, Boston, MA USA; 6 School of Public Health, Boston University, Boston, MA USA; 7 Department of Epidemiology, Harvard T.H. Chan School of Public Health, Boston, MA USA; 8 Department of Biostatistics, Harvard T.H. Chan School of Public Health, Boston, MA USA

**Keywords:** Global Flourishing Study, Prosocial behavior, Charitable giving, Donating, Helping strangers, Cross-national, Childhood, Adolescence, Epidemiology, Human behaviour

## Abstract

While prior work documents the individual and societal benefits of prosocial behaviors, less is known about how childhood experiences shape prosociality in adulthood. Using data from the Global Flourishing Study, a diverse and international sample of 202,898 individuals across 22 countries, we examined associations between 11 candidate childhood predictors (i.e., relationship with mother, relationship with father, parental marital status, financial status, experience of abuse, feeling like an outsider, childhood health, immigration status, religious service attendance, gender, age) with two prosocial behaviors in adulthood, charitable giving and helping strangers, and whether these associations varied by country. Random effects meta-analyses pooling estimates across all 22 countries showed evidence of associations between some candidate childhood predictors and an increased likelihood of both subsequent charitable giving and helping, and sensitivity analyses showed that associations with several (e.g., experiencing abuse, feeling like an outsider, age 12 religious service attendance) were at least moderately robust to unmeasured confounding. Of note, childhood factors did not uniformly predict both charitable giving and helping. Variations in the magnitude and direction of associations were also evident between countries, possibly reflecting diverse national influences on prosocial behaviors. With further research, these findings may inform policy and practice aimed at fostering prosociality around the world.

**Supplementary Information:**

The online version contains supplementary material available at 10.1038/s41598-024-77950-1.

## Introduction

Prosocial behaviors, which are voluntary acts intended to benefit others, play a vital role in fostering community and individual well-being^1^. Helping strangers and engaging in charitable giving are two common expressions of prosociality that benefit society^2^ and both have been positively associated with various health and well-being outcomes (e.g., reduced mortality risk, better psychosocial well-being)^3–6^. Because prosocial behaviors enhance individual and societal well-being, increasing prosociality is an important strategy for public health initiatives^7^. However, less is known about how to increase the prevalence of such behaviors because of limited research on the upstream correlates of prosociality.

We have some understanding of key determinants of prosocial behavior in adulthood and especially older adulthood^8,9^. These correlates include some health behaviors (e.g., physical activity), physical health conditions (e.g., physical functioning limitations, hypertension), and psychosocial factors (e.g., purpose in life). Still, there remains substantial unexplained variance. Little work considers whether early-life experiences shape prosociality in *adulthood* (most early-life studies assess prosociality in childhood) and most studies focus on North America and Europe.

Childhood is a critical period of development during which individuals begin to establish core beliefs, values, and social norms that shape their attitudes and behaviors later in life^10,11^. A large body of research has established that early-life experiences, especially negative ones, predict adulthood behavior and well-being^12^. Given the impact of childhood experiences on a wide array of health and well-being outcomes in adulthood, research has increasingly focused on ways to limit childhood experiences associated with negative outcomes and encourage those associated with positive outcomes. Indeed, the processes by which children and adolescents become kind, considerate, and helpful toward others have been an important area of inquiry^13^. However, few studies have systematically examined the influence of childhood experiences on the likelihood of engaging in prosocial behavior in adulthood.

We also have a limited understanding of whether childhood factors that shape prosocial behaviors differ across nations. Given the importance of prosociality for maintaining stable human societies, it is not surprising that values related to prosociality and altruism are found in many cultures. However, national comparisons reveal substantial diversity in adult cooperation and prosocial behaviors^14,15^. To deepen our understanding of the interplay of psychological and sociocultural influences on prosociality, a cultural developmental approach is needed^16^. Prior research suggests that although helping behaviors emerge early and at similar levels across diverse cultures^16^, differences in the likelihood or frequency of engaging in prosocial behaviors are already evident during childhood^17,18^.

### Literature review

Prior research on prosociality in general as well as specifically focused on charitable giving and helping, provides some insights into likely childhood predictors of prosociality in *adulthood*, including religious influences (e.g., childhood engagement in family faith activities)^19^, parental influences^20–22^, better socioeconomic status (SES)^23^, and the absence of negative family structure transitions (e.g., parental divorce)^23^. However, several childhood experiences (e.g., childhood adversity) show mixed findings, though this may be due to inconsistency in the measures of childhood adversity used across studies. For example, a recent meta-analysis found that early-life stress (measured by socioeconomic status, family unpredictability, exposures to abuse and neglect, or other stress in childhood or adolescence) was associated with lower prosociality in adulthood^24^. However, other studies have found that childhood adversity is associated with higher prosociality (as evidenced by associations between experiencing a traumatic event in childhood and elevated empathy in adulthood)^25^. Still other work has found null associations (e.g., between childhood physical abuse, sexual abuse and physical neglect, and adulthood prosocial tendencies)^26^.

### Limitations in prior research

While existing research has made meaningful contributions to our understanding of childhood predictors of prosociality in adulthood, there are several limitations we aim to address. First, most studies that assess childhood predictors of adulthood prosociality evaluate prosociality during emerging adulthood and fewer studies consider prosociality later in adulthood. To our knowledge, there is no research examining potential associations with a number of childhood factors, such as self-rated health in childhood, feeling like an outsider, and immigration status.

Second, prior studies have been inconsistent in how they measure prosociality which can make results difficult to compare. For example, some studies characterize prosociality according to levels of empathy, while others assess specific prosocial behaviors such as charitable giving. It may be of particular interest to evaluate prosocial behaviors such as charitable giving and helping, because these are modifiable, have been repeatedly associated with improved health and well-being outcomes^3–6^, and are directly tied to community well-being^27^. In the present study, we have used the same measures of these two key behavioral indicators of prosociality, charitable giving and helping, across the 22 countries we are examining.

Third, there is limited cross-national research in this area: de Guzman et al. (2014) states that, “the sampling of cultures is still somewhat limited. We still know little about the trajectory, correlates, and prosocial socialization experiences of children in less industrialized nations whose developmental landscape may be very different from children in North American samples more commonly represented in the literature.”^28^.

### The present study

In the present study, we used data from a diverse and international sample of 202,898 individuals across 22 countries to evaluate 11 childhood candidate predictors (e.g., childhood religious service attendance, childhood abuse; see Methods section for full list) of two commonly assessed prosocial behaviors in adulthood, charitable giving and helping. This study is nested within a broader group of studies investigating childhood predictors of human flourishing writ large. Because these analyses are part of a coordinated set of papers that are conducting analyses in parallel, we aimed to be as consistent as possible across all studies with analytic methods (so that any observed differences can be attributed to differences in constructs, and not differences in analytic choices). The linked methods were specifically crafted to allow for a panoramic view of childhood predictors of flourishing. The final set of candidate childhood predictors was carefully selected to broadly represent potential childhood determinants of any outcome. The choice of these indicators for use in the survey was a multi-phase process that is described in detail elsewhere^29^. The selected items were chosen based largely on theory and in consultation with collaborators as Gallup (who conducted the sampling) on which items were appropriate to keep. Still, multicollinearity issues led to some conceptually distinct variables being omitted or modified (though issues may still occur for specific outcomes and countries), and thus future work may benefit from exploring specific factors more deeply. For additional details on the assessments see the COS GFS codebook or other materials^30^.

First, we conducted an exploratory analysis to assess 11 childhood experiences, attributes, and circumstances as candidate predictors of charitable giving and helping in adulthood. We hypothesized that some factors would show meaningful associations with charitable giving and helping in adulthood, but we did not expect all childhood factors to predict adulthood prosociality.

Second, we assessed if these associations varied by country. We hypothesized that the strength of associations would vary by country, reflecting the influence of diverse sociocultural, economic, and health contexts that characterize each nation, although we did not have specific predictions for the direction of these differences.

Third, we assessed if the observed relationships were robust to potential unmeasured confounding, as assessed by *E*-values. We hypothesized that any observed associations would be robust against potential unmeasured confounding.

This hypothesis-generating approach allowed us to identify promising childhood predictors of two prosocial behaviors in adulthood, charitable giving and helping, which can undergo further investigation in future studies. The current study is also the first to investigate a consistently operationalized set of these predictors across a diverse group of countries, many with nationally representative samples, based on an inclusive, multistage process of variable selection informed by experts from around the world and from several of the countries under study (see^30,31^).

## Methods

The description of the methods below have been adapted from other materials^32^. Further methodological detail is available elsewhere^29,30,33–36^.

### Study population

The Global Flourishing Study (GFS) is a study of 202,898 participants from 22 geographically and culturally diverse countries, with nationally representative sampling within each country, concerning the distribution of determinants of well-being. Wave 1 of the data included the following countries and territories: Argentina, Australia, Brazil, Egypt, Germany, Hong Kong, India, Indonesia, Israel, Japan, Kenya, Mexico, Nigeria, the Philippines, Poland, South Africa, Spain, Sweden, Tanzania, Turkey, United Kingdom (UK), and the United States (US). The countries were selected to (a) maximize coverage of the world’s population, (b) ensure geographic, cultural, and religious diversity, and (c) prioritize feasibility and existing data collection infrastructure. Data collection was carried out by Gallup Inc. Data for Wave 1 were collected principally during 2023, with some countries beginning data collection in 2022 and exact dates varying by country^34^. Four additional waves of panel data on the participants will be collected annually from 2024 to 2027. The precise sampling design to ensure nationally representative samples varied by country and further details are available elsewhere^34^.

Survey items were all obtained via self-report (in face-to-face, telephone, or online surveys) and included aspects of well-being such as happiness, health, meaning, character, relationships, and financial stability^38^, along with other demographic, social, economic, political, religious, personality, childhood, community, health, and other variables. The data are publicly available through the Center for Open Science (https://www.cos.io/gfs). During the translation process, Gallup adhered to the TRAPD model (translation, review, adjudication, pretesting, and documentation) for cross-cultural survey research (ccsg.isr.umich.edu/chapters/translation/overview).

### Measures

We considered 11 childhood factors that comprised both internal attributes and external circumstances.

#### Childhood variables

*Relationship with mother during childhood* was assessed with the question: “Please think about your relationship with your mother when you were growing up. In general, would you say that relationship was very good, somewhat good, somewhat bad, or very bad?” Responses were dichotomized to very/somewhat good versus very/somewhat bad. An analogous variable was used for *relationship with father*. “Does not apply” was treated as a dichotomous control variable for respondents who did not have a mother or father due to death or absence. *Parental marital status during childhood* was assessed with responses of married, divorced, never married, and one or both had died. *Subjective financial status* was measured by asking participants: “Which one of these phrases comes closest to your own feelings about your family’s household income when you were growing up, such as when YOU were around 12 years old?” Responses were lived comfortably, got by, found it difficult, and found it very difficult. *Experience of abuse* was assessed with yes/no responses to “Were you ever physically or sexually abused when you were growing up? (yes/no)” *Feeling like an outsider while growing up* was assessed by asking participants: “When you were growing up, did you feel like an outsider in your family? (yes/no)”.

*Childhood health* was assessed with the item: “In general, how was your health when you were growing up? Was it excellent, very good, good, fair, or poor?” *Immigration status* was dichotomously assessed with one item: “Were you born in this country, or not?” Response options included ‘born in this country’ or ‘born in another country.’ *Religious attendance during childhood* was assessed with the item: “How often did YOU attend religious services or worship at a temple, mosque, shrine, church, or other religious building when YOU were around 12 years old?” with responses of at least once/week, one-to-three times/month, less than once/month, or never.

*Gender* was assessed as male, female, or other. *Continuous age* (year of birth) was classified as 18–24, 25–29, 30–39, 40–49, 50–59, 60–69, 70–79, and 80 or older. *Childhood religious tradition/affiliation* had response categories of Christianity, Islam, Hinduism, Buddhism, Judaism, Sikhism, Baha’i, Jainism, Shinto, Taoism, Confucianism, Primal/Animist/Folk religion, Spiritism, African-Derived, some other religion, or no religion/atheist/agnostic; precise response categories varied by country^39^. When the category no religion/atheist/agnostic had more than 5% of the within country sample size, this was used as the reference category; otherwise, the most prominent religious group was used. Additionally, all religious categories endorsed by less than 3% of the within country sample size were collapsed into a single religious category. *Racial/ethnic identity* was assessed in some, but not all, countries, and response categories were unique to each country. For inclusion in the childhood predictor regression analyses, racial/ethnic identity was collapsed into a binary variable of whether an individual was in the most prominent group versus a minority group (race plurality).

#### Outcome variables

Charitable giving was assessed with a single item, which asked, “In the past month, have you donated money to a charity?” Helping strangers was assessed with a single item, which asked, “In the past month, have you helped a stranger or someone you didn’t know who needed help?” Response options were binary yes/no for both items.

### Statistical analysis

We estimated descriptive statistics for the observed sample, weighted to be nationally representative within country, for each childhood demographic category. We fit a weighted modified Poisson regression model with complex survey adjusted standard errors within each country by regressing charitable giving and helping (in separate models) on all of the aforementioned childhood predictor variables simultaneously. In the primary analyses, country-specific regression coefficients were pooled using random effects meta-analyses of the regression coefficients^40,41^. For these, we report the meta-analytic average effect, confidence intervals for pooled estimates, the estimated proportions of effects across countries with effect sizes larger than 1.1 and smaller than 0.9, and I^2^ for evidence of variation within a given demographic category across countries^42^. Forest plots of estimates illustrate the heterogeneity of the associations by country (available in the online supplement).

Religious affiliation/tradition and race/ethnicity were used within country as control variables, when available, but these coefficients themselves were not included in the meta-analyses since categories/responses varied by country. All meta-analyses were conducted in **R **(R Core Team, 2024) using the metafor package^43^. Within each country, we conducted a global test of association of each childhood predictor variable with charitable giving and helping, and we computed a pooled (global) *p*-value^44^ across countries to test whether there was evidence of an association in any country. Bonferroni corrected *p*-value thresholds are provided based on the number of childhood demographic variables (i.e., *p*< .004)^45,46^. For each childhood predictor, we also calculated *E*-values to evaluate the sensitivity of results to unmeasured confounding. An *E*-value is the minimum strength of the association an unmeasured confounder must have with both the outcome and the predictor, above and beyond all measured covariates, for an unmeasured confounder to explain away an association^47^. As a supplementary analysis, we estimated population weighted meta-analyses of the regression coefficients. The population-weighted meta-analysis effectively treats each person in the 22 countries equally, rather than treating each of the 22 countries equally as the random effects meta-analysis does. All analyses were pre-registered with COS prior to data access, with only slight subsequent modification in the regression analyses due to multicollinearity of an expanded set the childhood predictors as shown in the pre-registration (https://doi.org/10.17605/OSF.IO/XNKA9); all code to reproduce analyses are openly available in an online repository^32^.

### Missing data

We imputed missing data on all variables using multivariate imputation by chained equations, and five imputed datasets were used^48–51^. To account for variation in the assessment of certain variables across countries (e.g., religious affiliation/tradition and race/ethnicity), we conducted the imputation process separately in each country. This within-country imputation approach ensured that the imputation models accurately reflected country-specific contexts and assessment methods. Sampling weights were included in the imputation models to account for specific-variable missingness that may have been related to probability of inclusion in the study.

### Accounting for complex sampling design

The GFS used different sampling schemes across countries based on availability of existing panels and recruitment needs^34^. All analyses accounted for the complex survey design components by including weights, primary sampling units, and strata. Additional methodological detail, including accounting for the complex sampling design, is provided elsewhere^36^.

## Results

### Descriptive statistics

Table 1 provides nationally representative (within each country) descriptive statistics on childhood predictors for the overall sample (all 22 countries combined) and other key sample characteristics. The sample comprised 51% females and was mostly evenly distributed across age groups (except for those age 80+). Participants more often reported having a very good relationship with their mother (63%) and father (53%), growing up in families with married parents (75%), perceiving their family’s financial status as “getting by” (41%), not experiencing abuse (82%), and not feeling like an outsider during childhood (84%). Participants also more often rated their health as ‘excellent’ (33%), identified as native to their country (94%), and attended religious services at least 1/week at age 12 (41%). The Supplementary Material provides nationally representative descriptive statistics by country (see Supplementary Tables S1a-S22a).

**Table 1:** Nationally representative childhood descriptive statistics and other key sample characteristics.

Characteristic	*N* = 202,898^1^
Relationship with mother	
Very good	127,836 (63%)
Somewhat good	52,439 (26%)
Somewhat bad	11,060 (5.5%)
Very bad	4,642 (2.3%)
Does not apply	5,965 (2.9%)
(Missing)	956 (0.5%)
Relationship with father	
Very good	107,742 (53%)
Somewhat good	55,714 (27%)
Somewhat bad	15,807 (7.8%)
Very bad	8,278 (4.1%)
Does not apply	13,985 (6.9%)
(Missing)	1,372 (0.7%)
Parent marital status	
Parents married	152,001 (75%)
Divorced	17,726 (8.7%)
Parents were never married	15,534 (7.7%)
One or both parents had died	7,794 (3.8%)
(Missing)	9,843 (4.9%)
Subjective financial status of family growing up	
Lived comfortably	70,861 (35%)
Got by	82,905 (41%)
Found it difficult	35,852 (18%)
Found it very difficult	12,606 (6.2%)
(Missing)	674 (0.3%)
Abuse	
Yes	29,139 (14%)
No	167,279 (82%)
(Missing)	6,479 (3.2%)
Outsider growing up	
Yes	28,732 (14%)
No	170,577 (84%)
(Missing)	3,589 (1.8%)
Self-rated health growing up	
Excellent	67,121 (33%)
Very good	63,086 (31%)
Good	47,378 (23%)
Fair	19,877 (9.8%)
Poor	4,906 (2.4%)
(Missing)	530 (0.3%)
Immigration status	
Born in this country	190,998 (94%)
Born in another country	9,791 (4.8%)
(Missing)	2,110 (1.0%)
Age 12 religious service attendance	
At least 1/week	83,237 (41%)
1–3/month	33,308 (16%)
< 1/month	36,928 (18%)
Never	47,445 (23%)
(Missing)	1,980 (1.0%)
Year of birth	
1998–2005; age 18–24	27,007 (13%)
1993–1998; age 25–29	20,700 (10%)
1983–1993; age 30–39	40,256 (20%)
1973–1983; age 40–49	34,464 (17%)
1963–1973; age 50–59	31,793 (16%)
1953–1963; age 60–69	27,763 (14%)
1943–1953; age 70–79	16,776 (8.3%)
1943 or earlier; age 80+	4,119 (2.0%)
(Missing)	20 (< 0.1%)
Gender	
Male	98,411 (49%)
Female	103,488 (51%)
Other	602 (0.3%)
(Missing)	397 (0.2%)
Country	
Argentina	6,724 (3.3%)
Australia	3,844 (1.9%)
Brazil	13,204 (6.5%)
Egypt	4,729 (2.3%)
Germany	9,506 (4.7%)
Hong Kong	3,012 (1.5%)
India	12,765 (6.3%)
Indonesia	6,992 (3.4%)
Israel	3,669 (1.8%)
Japan	20,543 (10%)
Kenya	11,389 (5.6%)
Mexico	5,776 (2.8%)
Nigeria	6,827 (3.4%)
Philippines	5,292 (2.6%)
Poland	10,389 (5.1%)
South Africa	2,651 (1.3%)
Spain	6,290 (3.1%)
Sweden	15,068 (7.4%)
Tanzania	9,075 (4.5%)
Turkey	1,473 (0.7%)
United Kingdom	5,368 (2.6%)
United States	38,312 (19%)

^1^n (%).

### Random effects meta-analytic estimates

Tables 2 and 3 show random effects meta-analytic estimates of *charitable giving* and *helping behavior*, respectively, regressed on the 11 candidate childhood predictors included in the model simultaneously in country-specific analyses. A number of childhood factors showed evidence of associations with charitable giving and helping in adulthood, supporting our first hypothesis.

**Table 2:** Random effects meta-analysis of regression of charitable giving on childhood predictors.

				Estimated Proportion of Effects by Threshold			
Variable	Category	RR	95% CI	< 0.90	> 1.10	I^2^	Global *p*-value
Relationship with mother	(Ref: Very bad/somewhat bad)						0.017*
	Very good/somewhat good	1.04	(0.98,1.11)	0.09	0.36	59.9	
Relationship with father	(Ref: Very bad/somewhat bad)						0.053
	Very good/somewhat good	1.05	(1.02,1.08)	0.00	0.00	15.8	
Parent marital status	(Ref: Parents married)						0.289
	Divorced	0.99	(0.96,1.03)	0.00	0.00	31.1	
	Single, never married	1.00	(0.95,1.05)	0.00	0.05	25.5	
	One or both parents had died	1.02	(0.99,1.05)	0.00	0.00	< 0.1‡	
Subjective financial status of family growing up	(Ref: Got by)						< 0.001**
	Lived comfortably	1.07	(1.04,1.11)	0.00	0.36	72.1	
	Found it difficult	0.97	(0.94,1.01)	0.09	0.00	58.3	
	Found it very difficult	1.00	(0.95,1.06)	0.05	0.14	32.8	
Abuse	(Ref: No)						< 0.001**
	Yes	1.11	(1.06,1.16)	0.00	0.52	72.1	
Outsider growing up	(Ref: No)						< 0.001**
	Yes	1.11	(1.04,1.18)	0.00	0.45	83.3	
Self-rated health growing up	(Ref: Good)						< 0.001**
	Excellent	1.08	(1.03,1.13)	0.05	0.36	71.6	
	Very good	1.06	(1.02,1.11)	0.05	0.32	70.1	
	Fair	0.99	(0.95,1.04)	0.05	0.00	39.7	
	Poor	1.08	(1.00,1.18)	0.05	0.36	47.2	
Immigration status	(Ref: Born in this country)						< 0.001**
	Born in another country	0.58	(0.19,1.72)	0.36	0.23	99.9	
Age 12 religious service attendance	(Ref: Never)						< 0.001**
	At least 1/week	1.33	(1.21,1.47)	0.05	0.82	90.7	
	1–3/month	1.28	(1.15,1.43)	0.05	0.73	92.3	
	< 1/month	1.12	(1.02,1.24)	0.09	0.55	90.5	
Year of birth	(Ref: 1998–2005; age 18–24)						< 0.001**
	1993–1998; age 25–29	1.22	(1.14,1.31)	0.00	0.82	75.2	
	1983–1993; age 30–39	1.26	(1.15,1.37)	0.00	0.64	89.2	
	1973–1983; age 40–49	1.28	(1.16,1.42)	0.09	0.77	91.0	
	1963–1973; age 50–59	1.28	(1.16,1.41)	0.09	0.77	88.1	
	1953–1963; age 60–69	1.34	(1.19,1.50)	0.05	0.77	88.6	
	1943–1953; age 70–79	1.42	(1.19,1.69)	0.14	0.77	92.0	
	1943 or earlier; age 80+	1.60	(1.35,1.90)	0.14	0.86	87.1	
Gender	(Ref: Male)						< 0.001**
	Female	0.94	(0.88,1.00)	0.41	0.14	94.9	
	Other	0.04	(0.00,0.51)	0.56	0.33	99.7	

*Note. N* = 202,898. *p < .05; **p < .004 (Bonferroni corrected threshold); ‡estimate of heterogeneity is likely unstable, please see our online supplement forest plots for more detail on heterogeneity of effects. ‡‡Group is very small (< 0.1% of the observed sample) within several countries leading large uncertainty in this estimate or even complete separation—be cautious about interpreting this estimate; CI = confidence interval; the estimated proportion of effects is the estimated proportion of effects above (or below) a threshold based on the calibrated effect sizes (Mathur & VanderWeele, 2020); *I*^2^ is an estimate of the variability in means due to heterogeneity across countries vs. sampling variability; the Global *p*-value corresponds to the joint test of the null hypothesis that the country-specific joint parameter Wald tests (all parameters within variable groups are zero) are all null all 22 countries; and additional details of heterogeneity of effects are available in the forest plots of our online supplemental material.

**Table 3:** Random effects meta-analysis of regression of helping on childhood predictors.

				Estimated Proportion of Effects by Threshold			
Variable	Category	RR	95% CI	< 0.90	> 1.10	I^2	Global *p*-value
Relationship with mother	(Ref: Very bad/somewhat bad)						0.418
	Very good/somewhat good	1.02	(0.99,1.04)	0.00	0.00	1.8	
Relationship with father	(Ref: Very bad/somewhat bad)						0.302
	Very good/somewhat good	1.02	(1.00,1.03)	0.00	0.00	< 0.1‡	
Parent marital status	(Ref: Parents married)						< 0.001**
	Divorced	1.04	(1.02,1.07)	0.00	0.00	< 0.1‡	
	Single, never married	1.04	(1.01,1.06)	0.00	0.05	30.0	
	One or both parents had died	1.03	(1.00,1.07)	0.00	0.05	21.0	
Subjective financial status of family growing up	(Ref: Got by)						0.002**
	Lived comfortably	1.03	(1.00,1.05)	0.00	0.05	59.3	
	Found it difficult	0.99	(0.98,1.01)	0.00	0.00	4.0	
	Found it very difficult	1.03	(1.00,1.06)	0.00	0.00	14.1	
Abuse	(Ref: No)						< 0.001**
	Yes	1.11	(1.08,1.16)	0.00	0.57	82.2	
Outsider growing up	(Ref: No)						< 0.001**
	Yes	1.08	(1.06,1.10)	0.00	0.27	42.4	
Self-rated health growing up	(Ref: Good)						< 0.001**
	Excellent	1.06	(1.02,1.10)	0.05	0.32	77.1	
	Very good	1.04	(1.00,1.07)	0.05	0.14	78.2	
	Fair	1.03	(1.00,1.07)	0.00	0.14	52.8	
	Poor	1.05	(1.00,1.09)	0.00	0.14	19.3	
Immigration status	(Ref: Born in this country)						< 0.001**
	Born in another country	1.04	(0.98,1.10)	0.09	0.32	74.9	
Age 12 religious service attendance	(Ref: Never)						< 0.001**
	At least 1/week	1.19	(1.10,1.29)	0.05	0.77	92.9	
	1–3/month	1.20	(1.10,1.31)	0.00	0.73	92.5	
	< 1/month	1.11	(1.05,1.17)	0.00	0.55	82.6	
Year of birth	(Ref: 1998–2005; age 18–24)						< 0.001**
	1993–1998; age 25–29	1.00	(0.96,1.04)	0.09	0.09	76.2	
	1983–1993; age 30–39	1.00	(0.95,1.05)	0.18	0.18	87.5	
	1973–1983; age 40–49	0.98	(0.92,1.04)	0.27	0.27	90.3	
	1963–1973; age 50–59	0.96	(0.88,1.05)	0.32	0.27	94.8	
	1953–1963; age 60–69	0.87	(0.78,0.98)	0.45	0.23	95.1	
	1943–1953; age 70–79	0.78	(0.67,0.90)	0.68	0.23	94.0	
	1943 or earlier; age 80+	0.71	(0.58,0.86)	0.64	0.14	88.0	
Gender	(Ref: Male)						< 0.001**
	Female	0.94	(0.90,0.98)	0.32	0.00	93.0	
	Other	0.25	(0.04,1.51)	0.33	0.39	99.9	

*Note. N* = 202,898. *p < .05; **p < .004 (Bonferroni corrected threshold); ‡estimate of heterogeneity is likely unstable, please see our online supplement forest plots for more detail on heterogeneity of effects. ‡‡Group is very small (< 0.1% of the observed sample) within several countries leading large uncertainty in this estimate or even complete separation—be cautious about interpreting this estimate; CI = confidence interval; the estimated proportion of effects is the estimated proportion of effects above (or below) a threshold based on the calibrated effect sizes (Mathur & VanderWeele, 2020); *I*^2^ is an estimate of the variability in means due to heterogeneity across countries vs. sampling variability; the Global *p*-value corresponds to the joint test of the null hypothesis that the country-specific joint parameter Wald tests (all parameters within variable groups are zero) are all null all 22 countries; and additional details of heterogeneity of effects are available in the forest plots of our online supplemental material.

#### Charitable giving

Table 2 shows random-effects meta-analytic estimates of *charitable giving* in adulthood regressed on the 11 candidate childhood predictors simultaneously in country-specific analyses. The meta-analyses provide evidence that several candidate childhood factors were associated with a modest increased likelihood of charitable giving in adulthood, including.

1. Having a very good/somewhat good versus a very bad/somewhat bad *relationship with father* (RR = 1.05, 95% CI: 1.02, 1.08);
2. *Subjective financial status* as ‘lived comfortably’ versus ‘got by*’* in childhood (RR = 1.07, 95% CI: 1.04, 1.11);
3. Experiencing *childhood abuse* versus not experiencing childhood abuse (RR = 1.11, 95% CI: 1.06, 1.16);
4. *Feeling like an outsider growing up* versus not feeling like an outsider growing up (RR = 1.11, 95% CI: 1.04, 1.18);
5. Having ‘excellent’ (RR = 1.08, 95% CI: 1.03, 1.13) or ‘very good’ (RR = 1.06, 95% CI: 1.02, 1.11) *self-rated health* versus ‘good’ self-rated health;
6. More frequent *religious service attendance at age 12*: compared to never attending services, those who attended religious services < 1/month (RR = 1.12, 95% CI: 1.02, 1.24), 1–3/month (RR = 1.28, 95% CI: 1.15, 1.43), and at least 1/week (RR = 1.33, 95% CI: 1.21, 1.47); and
7. Older *age*: all age groups were associated with an increased likelihood of charitable giving as compared to 18–24-year-olds, i.e., from age 25–29 (RR = 1.22, 95% CI: 1.14, 1.31) to age 80+ (RR = 1.60, 95% CI: 1.35, 1.90).

Pooling across countries, the meta-analytic estimates of the effect of relationship quality with one’s mother, parental marital status, immigration status, and gender showed little evidence of associations with charitable giving in adulthood above and beyond all the other variables included. However, these factors (with exception of quality of relationship with one’s father and parental marital status) showed associations with charitable giving in adulthood in at least one of analyzed countries, as indicated by global *p*-values < 0.05 and country-specific results.

#### Helping

Table 3 shows random effects meta-analytic estimates of *helping behavior* regressed on the 11 candidate childhood predictors simultaneously in country-specific analyses. Our meta-analysis showed that the following childhood factors were associated with an increased likelihood of helping in adulthood.

1. Having parents who were *divorced* (RR = 1.04, 95% CI: 1.02, 1.07), or *single*,* never married* (RR = 1.04, 95% CI: 1.01, 1.06) compared to having married parents;
2. Experiencing *childhood abuse* versus not experiencing childhood abuse (RR = 1.11, 95% CI: 1.08, 1.16);
3. *Feeling like an outsider growing up* versus not feeling like an outsider growing up (RR = 1.08, 95% CI: 1.06, 1.10);
4. Having ‘excellent’ versus ‘good’ *self-rated health* (RR = 1.06, 95% CI: 1.02, 1.10); and
5. More frequent *religious service attendance at age 12*: compared to never attending services, those who attended religious services < 1/month (RR = 1.11, 95% CI: 1.05, 1.17), 1–3/month (RR = 1.20, 95% CI: 1.10, 1.31), and at least 1/week (RR = 1.19, 95% CI: 1.10, 1.29).

On the other hand, helping declined in the oldest *age* groups compared to 18–24-year-olds, i.e., for age 60–69 (RR = 0.87, 95% CI: 0.78, 0.98); 70–79 (RR = 0.78, 95% CI: 0.67, 0.90); and 80+ (RR = 0.71, 95% CI: 0.58, 0.86). Being *female* (versus male) was also associated with a lower likelihood of helping (RR = 0.94, 95% CI: 0.90, 0.98). Pooling across countries, the meta-analytic estimates of the effect of relationship quality with one’s mother and father, subjective financial status of family, and immigration status showed little evidence of associations with helping in adulthood above and beyond all the other variables included. The global *p*-values < 0.05 and country-specific results suggest there was evidence of association between each candidate childhood predictor and helping in adulthood in at least one country, with the exception of quality of relationships with one’s mother and father.

### *E*-values

Tables 4 and 5 show *E*-values which suggested that a few of the observed associations were moderately robust to unmeasured confounding. Thus, our third hypothesis was partially supported, such that some of the observed relationships showed evidence of being robust against potential unmeasured confounding. For example, to explain away the association between religious service attendance 1/week at age 12 and charitable giving, an unmeasured confounder would have to be associated with both age 12 religious service attendance and charitable giving with risk ratios of 2.00 each (above and beyond the covariates already adjusted for). A confounder that had weaker associations with religious service attendance and charitable giving would not remove the association between them (Table 4). Similarly, to shift the confidence interval to include the null, an unmeasured confounder would have to be associated with both religious service attendance and charitable giving by risk ratios of 1.72. However, several other associations were not particularly robust to unmeasured confounding, especially for helping, as can be seen from the often modest *E*-values.

**Table 4:** Sensitivity of meta-analyzed childhood predictors to unmeasured confounding – charitable giving.

Variable	Category	E-value for Estimate	E-value for 95% CI
Relationship with mother	(Ref: Very bad/somewhat bad)		
	Very good/somewhat good	1.26	1.00
Relationship with father	(Ref: Very bad/somewhat bad)		
	Very good/somewhat good	1.28	1.14
Parent marital status	(Ref: Parents married)		
	Divorced	1.09	1.00
	Single, never married	1.03	1.00
	One or both parents had died	1.16	1.00
Subjective financial status of family growing up	(Ref: Got by)		
	Lived comfortably	1.35	1.23
	Found it difficult	1.19	1.00
	Found it very difficult	1.05	1.00
Abuse	(Ref: No)		
	Yes	1.45	1.30
Outsider growing up	(Ref: No)		
	Yes	1.46	1.26
Self-rated health growing up	(Ref: Good)		
	Excellent	1.36	1.20
	Very good	1.32	1.16
	Fair	1.08	1.00
	Poor	1.38	1.00
Immigration status	(Ref: Born in this country)		
	Born in another country	2.85	1.00
Age 12 religious service attendance	(Ref: Never)		
	At least 1/week	2.00	1.72
	1–3/month	1.89	1.56
	< 1/month	1.49	1.14
Year of birth	(Ref: 1998–2005; age 18–24)		
	1993–1998; age 25–29	1.75	1.55
	1983–1993; age 30–39	1.83	1.57
	1973–1983; age 40–49	1.88	1.58
	1963–1973; age 50–59	1.88	1.60
	1953–1963; age 60–69	2.01	1.67
	1943–1953; age 70–79	2.18	1.65
	1943 or earlier; age 80+	2.57	2.03
Gender	(Ref: Male)		
	Female	1.34	1.00
	Other	44.19	3.30

**Table 5:** Sensitivity of meta-analyzed childhood predictors to unmeasured confounding – helping.

Variable	Category	E-value for Estimate	E-value for 95% CI
Relationship with mother	(Ref: Very bad/somewhat bad)		
	Very good/somewhat good	1.15	1.00
Relationship with father	(Ref: Very bad/somewhat bad)		
	Very good/somewhat good	1.14	1.00
Parent marital status	(Ref: Parents married)		
	Divorced	1.26	1.18
	Single, never married	1.24	1.12
	One or both parents had died	1.21	1.00
Subjective financial status of family growing up	(Ref: Got by)		
	Lived comfortably	1.19	1.07
	Found it difficult	1.08	1.00
	Found it very difficult	1.21	1.06
Abuse	(Ref: No)		
	Yes	1.47	1.36
Outsider growing up	(Ref: No)		
	Yes	1.37	1.30
Self-rated health growing up	(Ref: Good)		
	Excellent	1.32	1.18
	Very good	1.23	1.00
	Fair	1.22	1.00
	Poor	1.26	1.06
Immigration status	(Ref: Born in this country)		
	Born in another country	1.24	1.00
Age 12 religious service attendance	(Ref: Never)		
	At least 1/week	1.68	1.44
	1–3/month	1.69	1.44
	< 1/month	1.45	1.27
Year of birth	(Ref: 1998–2005; age 18–24)		
	1993–1998; age 25–29	1.03	1.00
	1983–1993; age 30–39	1.04	1.00
	1973–1983; age 40–49	1.17	1.00
	1963–1973; age 50–59	1.26	1.00
	1953–1963; age 60–69	1.55	1.16
	1943–1953; age 70–79	1.90	1.46
	1943 or earlier; age 80+	2.17	1.58
Gender	(Ref: Male)		
	Female	1.32	1.16
	Other	7.50	1.00

### Country-specific associations

Supplementary Tables S1b-S22b provide the country-specific associations between candidate childhood predictors and charitable giving and helping, and Supplementary Tables S1c-S22c contain *E*-value analyses for these associations. Supplementary Figures S1-S27 provide forest plots for candidate childhood predictors by country. Results for meta-analyses using a population weighted approach and *E*-values for these analyses are presented in Supplementary Tables S23-S24, respectively. Our second hypothesis was supported, such that the strength and direction of associations varied by country. We comment below on some notable associations in specific countries.

#### Relationships with parents and parental marital status

Having a good relationship with one’s father was associated with a higher likelihood of charitable giving in Nigeria and Sweden, and having a good relationship with one’s mother was associated with a higher likelihood of charitable giving in Japan and Turkey, but a lower likelihood of charitable giving in Israel. Having a good relationship with one’s father was associated with increased helping only in Nigeria, and having a good relationship with one’s mother was associated with increased helping only in Kenya.

There was evidence that parental marital status of being divorced or never married/single, or having one or both parents die (all versus parents being married) were associated with likelihood of charitable giving and helping in several countries, but in different directions. For charitable giving, compared with having married parents, having divorced parents was associated with a higher likelihood of charitable giving in Germany and India, having never married parents was associated with higher likelihood of charitable giving in Egypt, and having one or both parents die was associated with a higher likelihood of charitable giving in Nigeria. However, in the US and Israel, having divorced parents was associated with a lower likelihood of charitable giving.

For helping, having divorced parents (versus parents being married) was associated with a higher likelihood of helping in Mexico and Spain; having never married parents was associated with a higher likelihood of helping in Spain and the UK, and having one or both parents die was associated with a higher likelihood of helping in Argentina and Poland.

#### Financial status

There was evidence that ‘living comfortably’ financially while growing up versus ‘getting by’ was associated with an increased likelihood of charitable giving in five countries: the Philippines, Nigeria, Poland, Kenya, and Egypt. In some countries (e.g., Tanzania, Hong Kong, India, Egypt), having a ‘difficult’ or ‘very difficult’ financial status growing up was associated with decreased charitable giving, while the association was reversed in other countries (e.g., Australia, Mexico).

For helping, there was evidence that ‘living comfortably’ financially versus ‘getting by’ was associated with a moderate increase in helping in adulthood across five countries (Hong Kong, Japan, Egypt, the Philippines, and Nigeria), but finding things ‘very difficult’ was also associated with increased helping in Argentina and the US. In Egypt, ‘living comfortably’ was associated with a higher likelihood of helping while ‘finding it difficult’ was associated with a decreased likelihood of helping.

#### Abuse

There was evidence in eight countries that experiencing physical or sexual abuse in childhood versus not was associated with a higher likelihood of charitable giving, with the effect sizes ranging from 12% in Brazil (95% CI: 1.03, 1.21) to 58% in Japan (95% CI: 1.33, 1.87). In 14 countries, there was evidence that experiencing physical or sexual abuse in childhood versus not was also associated with an increased likelihood of helping in adulthood, ranging from an 7% increase in Kenya (95% CI: 1.02, 1.12) and Brazil (95% CI: 1.02, 1.11) to a 59% increase in Japan (95% CI: 1.38, 1.83). Conversely, there was little evidence in any country that experiencing childhood abuse was ever associated with lower charitable giving or helping.

#### Feeling like an outsider

Feeling like an outsider during childhood versus not was also associated with higher likelihood of charitable giving in nine countries with the strongest effects in South Africa (RR = 1.60, 95% CI: 1.25, 2.04) and Japan (RR = 1.46, 95% CI: 1.24, 1.72); however it was associated with a lower likelihood of charitable giving in Australia (RR = 0.88, 95% CI: 0.78, 0.99). In ten countries, there was notable evidence that feeling like an outsider was associated with higher likelihood of helping, ranging from 6% in Brazil (95% CI: 1.01, 1.11) to 29% in Japan (95% CI: 1.13, 1.49).

#### Self-rated health

Findings for whether childhood self-rated health predicted adult prosocial behaviors were mixed between countries. ‘Excellent’ versus ‘good’ health during childhood was associated with a higher likelihood of charitable giving and helping in ten countries each. For charitable giving, the strongest associations were in South Africa, Israel and Hong Kong. However, in Spain, ‘excellent’ (versus ‘good’) health was associated with a lower likelihood of charitable giving. ‘Poor’ (versus ‘good’) self-rated health was associated with an increased likelihood of charitable giving in India and Poland, and a decreased likelihood in Israel.

For helping, perceptions of having ‘excellent’ or ‘poor’ self-rated health growing up (versus ‘good’) was associated with more helping in several countries. Exceptions were Poland (where ‘excellent’ and ‘very good’ self-rated health were both associated with a decreased likelihood of helping) and Hong Kong (where ‘fair’ self-rated health was associated with a decreased likelihood of helping).

#### Religious service attendance at age 12

There was notable evidence in 14 countries that religious service attendance at least 1/week at age 12 versus never attending was associated with an increased likelihood of charitable giving. However, the strength of association differed by country. In Japan and Israel, the association was strong, i.e., for those who attended at least 1/week in Japan the risk ratio was 2.20 (95% CI: 1.72, 2.80) and in Israel it was 2.31 (95% CI: 2.08, 2.58), whereas in Spain it was weaker (RR = 1.16, 95% CI: 1.02, 1.32). Conversely in Nigeria, the association between religious service attendance at age 12 and charitable giving appeared curvilinear, and those with moderate religious service attendance (i.e., 1–3/month and < 1/month) had a decreased likelihood of charitable giving relative to those who never attended. In all other countries with evidence of associations between religious service attendance and charitable giving, those who attended more versus never attended had a higher likelihood of giving.

Similarly, in 15 countries, attending religious services at least 1/week at age 12 versus never was associated with an increased likelihood of helping, though the association was much stronger in some countries (e.g., Japan; RR = 2.33, 95% CI: 1.88, 2.89) than in others (e.g., US; RR = 1.10, 95% CI: 1.03, 1.17). There was little evidence in any country that weekly or more attendance was associated with a decreased likelihood of helping.

#### Religious affiliation

Religious affiliation was associated with charitable giving and helping in several countries. However, these associations are difficult to summarize because the size of religious and non-religious groups varies so substantially between countries. Thus, the reference category was being non-religious in 14 countries. Most countries in which we observed an association between religious affiliation and charitable giving and/or helping had non-religious as a reference group. In most cases, individuals who were members of religious groups report giving and helping more than non-religious individuals. However, in several countries, the non-religious sample size was too small to evaluate, so other religious groups were chosen as reference categories. Another complication is that small religious groups were combined, possibly putting traditions with ‘high-giving/helping’ and ‘low-giving/helping’ in the same group.

#### Sociodemographic factors

Regarding sociodemographic factors, charitable giving generally increased with age, though there were some exceptions (e.g., in Tanzania, a decreased likelihood of charitable giving in the oldest age groups). Helping generally decreased with age, again with some exceptions (e.g., an increased likelihood of helping in the oldest age group in Hong Kong). Findings for gender were mixed, though in many countries, being female was associated with a lower likelihood of charitable giving and helping (e.g., Germany, Kenya, Mexico, Philippines, Spain, Tanzania), but more in others (e.g., Australia, Poland). Associations for immigration status were mixed for both charitable giving and helping. Regarding the former, in some countries being an immigrant was associated with an increased likelihood of charitable giving (e.g., Egypt, Tanzania), while in others – with a decreased likelihood of charitable giving (e.g., Israel, Spain, and the UK). Regarding the latter, being born in another country was associated with an increased likelihood of helping in Egypt, Spain, and the UK, but a decreased of helping in Israel. Reporting minority status was associated with a decreased likelihood of charitable giving in four countries, but in remaining countries there was little evidence of such an association. For helping, minority status was associated with an increased likelihood of helping in three countries and a decreased likelihood of helping in just Indonesia.

## Discussion

Using data from an international sample of 202,898 individuals across 22 countries, we assessed the associations between 11 childhood candidate predictors and both charitable giving and helping in adulthood. Our findings are similar to previous research for childhood religious involvement^19^, better relationships with parents^20–22^, and higher SES^23^, which were all associated with more prosociality. However, our findings differ from other research for some candidate predictors. For example, parental marital status was not associated with charitable giving in our study^23^. Overall, there were a number of small effect sizes (though many are comparable to those found in prior research on adulthood predictors of prosocial behaviours)^8^ and some surprising findings (e.g., both experiencing childhood abuse and feeling like an outsider growing up were associated with more charitable giving and helping). Some findings may be due to methodological constraints, such as self-report/retrospective bias. Multiple findings call for follow-up research to better isolate causation.

Several aspects of childhood environments may promote charitable giving and helping in adulthood. For example, religious service attendance at age 12 was associated with an increased likelihood of charitable giving and helping in adulthood both in the overall sample and in most individual countries. Those who attend religious services (regardless of how often) typically gave and helped more in adulthood than those who never attended. Moreover, in most countries, the association was monotonic: those who attended more gave and helped more. This is consistent with previous literature in which childhood engagement in religious activities has been associated with increased prosociality in adulthood^19^. There are many possible explanations for this association, including: cultural norms and values imparted by religion, continuation of religious beliefs and practices into adulthood, and modeling parental practices – amongst others.

Likewise, having a good relationship with one’s father was associated with charitable giving. This association was especially notable for charitable giving in Sweden, Nigeria and for helping in Nigeria. This may be because a good relationship with one’s father fosters a sense of security and greater emotional and financial resource availability, all of which may encourage subsequent prosociality. Having a good relationship with one’s mother was associated with an increased likelihood of charitable giving in Turkey and Japan and a decreased likelihood in Israel. A good relationship with one’s mother was associated with an increased likelihood of helping in Kenya. However, these effect sizes are small and may not be robust to unmeasured confounding.

There was some evidence that more healthy childhood environments were associated with increased charitable giving and/or helping (e.g., good relationships with parents, higher SES, better self-rated health, more frequent religious service attendance). However, our meta-analysis indicated that some adverse childhood factors (namely, experiencing physical or sexual abuse; feeling like an outsider; and having divorced parents or single parents) were also associated with increased charitable giving and/or helping in the overall sample and in several individual countries. In other countries, there were null or negative associations.

Contrary to what we might expect, those who have experienced childhood abuse may have increased empathy and compassion, which may promote some forms of prosociality^25,52^. Notably, these associations varied in magnitude, with effects ranging from 12 − 58% for charitable giving and from 7 − 59% for helping (see Results). Indeed, some who have experienced this kind of trauma may help others through an “altruism born of suffering” or posttraumatic growth^53,54,62^. An emerging body of research examines *posttraumatic growth*^55^ and *growth through loss and adversity*. Moreover, some prior work finds associations between increased traumatic events in one’s lifetime and increased helping^56^, as well as similar associations between childhood abuse and increased likelihood of volunteering in adulthood^57^. Engaging in prosocial behaviors may further serve as a means for children who experienced childhood adversity to create a positive self-identity through prosocial actions, cope with their experiences, and even seek out social connection with others. Alternatively, abuse might lead people to seek out an alternative family, such as in close knit religious groups, where helping and giving are often encouraged and modeled. Finally, people who experience abuse may feel shame and compensate with more socially desirable responses. However, these are all currently speculations. How and why specific countries in our sample encourage such growth among those who experienced abuse as a child or who felt like an outsider during childhood requires further research.

Similarly, having parents who were divorced or never married/single was associated with an increased likelihood of helping in both the overall sample and some countries, and associated with charitable giving in some countries. This conflicts with prior findings, in which parental divorce in adolescence was associated with lower subsequent charitable giving in adulthood^22^. However, in the US and Israel, having divorced parents was associated with a decreased likelihood of charitable giving, and much of the prior work has been conducted in the US. It is possible that in some countries, adverse family structures may increase the likelihood of charitable giving and helping due to a heightened sense of empathy and understanding of adversity, or an increased sense of community and social responsibility (individuals who experience family instability may develop a stronger sense of community and social responsibility as a way to find support and connection).

The same childhood factors were associated with higher prosociality in some countries and lower prosociality in others. For example, ‘excellent,’ ‘very good,’ ‘fair,’ and ‘poor’ self-rated health in childhood, versus ‘good’ self-rated health, were all associated with an increased likelihood of charitable giving and/or helping in adulthood in country-specific analyses. Overall, the pattern with childhood health was U-shaped. On the one hand, a foundation of adequate physical health in childhood may help foster the development of key cognitive, emotional, and social skills that facilitate prosociality. On the other hand, hardship related to health in childhood may increase empathy and prosociality. Similarly, for subjective financial status of family, associations were mixed in country-specific analyses. Greater monetary resources during childhood may translate to greater monetary resources in adulthood and the ability to give financially, while lower SES in childhood may foster empathy that increases prosociality. Further research is needed to better understand these mixed associations.

Finally, regarding sociodemographic factors, year of birth was associated with an increased likelihood of charitable giving but a decreased likelihood of helping strangers. This is likely more due to age-related changes than cohort effects. For instance, charitable giving increased with age, potentially due to increased financial stability, more disposable income/wealth, approaching death, and greater social responsibility in some countries (though in some countries, depending on pension systems, financial conditions are more unfavorable for older adults)^58^. However, there were some exceptions. For example, in Tanzania, charitable giving decreased with age, perhaps because the country is poorer, making it harder for individuals to accumulate financial resources over the life course^59^. In contrast, helping declined with age, primarily for those over 60. Perhaps among older people, mobility limitations, and lower self-rated health may impede helping strangers or reduce contact with strangers^8,9,60,63^.

Findings for gender were mixed for charitable giving and helping, though in many countries, being female was associated with a lower likelihood of helping strangers. This may have to do with men having less fear in interaction with strangers or with social stigma of women interacting with non-related men in some societies. Further, as described elsewhere^61^, males may have more resources and social capital that facilitate charitable giving and helping strangers, while females may do more “invisible work” in their homes (e.g., cleaning).

Our study has several limitations:

1. *Retrospective reporting of childhood predictors*: Childhood predictors were reported retrospectively, and thus recall bias is possible. Recall bias may be smaller for some variables (e.g., immigrant status) than others (e.g., subjective financial status of family growing up). However, recall bias would not explain away several of the observed associations unless charitable giving and helping bias retrospective assessments of the childhood predictors with a magnitude as strong as the observed associations themselves^62^.
2. *Scope of candidate childhood predictors*: We did not assess all childhood factors that might predict adulthood charitable giving and helping (e.g., civic education). In the initial analysis of the GFS data, across all coordinated studies reporting findings with Wave 1, the decision was made to use the same childhood predictors across all studies. This allows for easy comparison between studies about which childhood factors predict different measures of human flourishing. This orchestrated approach allows a panoramic view of the childhood ‘roots’ of human flourishing writ large. Future analyses can more precisely assess additional predictors of these specific outcomes.
3. *Measurement of prosociality*: We measured monthly prevalence of charitable giving and helping, rather than frequency and scope, which will be important to assess in future work. Elsewhere we evaluate the childhood predictors of volunteering^57^. Future work may benefit from evaluating other forms of prosociality (e.g., empathy, cooperation) which were not assessed in the GFS.
4. *Potential for unmeasured confounding*: There is always potential for confounding by unmeasured variables in observational research. However, we considered a range of potential confounding variables and *E*-value analyses suggested that some of the observed associations were moderately robust to unmeasured confounding.

This study also had many strengths: it was among the first studies to evaluate childhood predictors of charitable giving and helping in adulthood cross-nationally, using large, diverse, and nationally representative samples from 22 countries.

Our findings highlight the potential impact of early-life experiences, personal attributes, and familial or social circumstances that may shape adulthood prosociality. Some of our findings were surprising. Both positive (e.g., age 12 religious service attendance) and negative aspects of childhood environments (e.g., experiencing physical or sexual abuse, feeling like an outsider) were associated with an increased likelihood of charitable giving and helping in adulthood, and there was meaningful variation between countries in the magnitude and direction of associations. Positive aspects of childhood environments may be studied in future research to identify targets for interventions that foster adulthood prosociality, and further work is needed to understand when and why negative aspects of childhood environments promote adulthood prosociality. With further research, these findings may inform the eventual development of programs, policies, and interventions designed to foster prosociality, and in turn, promote societal and individual well-being around the world.

## Electronic Supplementary Material

Below is the link to the electronic supplementary material.

Supplementary Material 1

## Data Availability

Data for Wave 1 of the Global Flourishing Study is available through the Center for Open Science upon submission of a pre-registration (https://doi.org/10.17605/OSF.IO/3JTZ8). Please see https://www.cos.io/gfs-access-data for more information about data access. All analyses were pre-registered with COS prior to data access (https://osf.io/gz3uq/?view_only=d775a119a72b4695af471d7a0e874835); all code to reproduce analyses are openly available in an online repository (https://doi.org/10.17605/OSF.IO/VBYPE).
