# Supplementary material for: Childhood predictors of charitable giving and helping across 22 countries in the Global Flourishing Study
Source: Sci Rep. 2025 Apr 30;15:14493. doi: 10.1038/s41598-024-77950-1 (PMC12044005; doi:10.1038/s41598-024-77950-1)
Supplement: Supplementary file 1 — Supplementary Material 1 [file 41598_2024_77950_MOESM1_ESM.docx]

**Supplementary Material**

***Childhood Predictors of Charitable Giving and Helping Across 22 Countries in the Global Flourishing Study***

This online supplement to the Global Flourishing Study paper on charitable giving and helping has several important caveats to interpretation. First, estimating the within country group means can be unstable if the group size is small (<1%) of the country sample size. In such cases, the uncertainty in the estimate leads to a multiple imputation adjusted degrees of freedom less than 1. This means there is not enough information to evaluate the uncertainty in the estimate. We flagged such cases with a “*”. Secondly, in rare instances for childhood predictor analyses the confidence interval of the effect estimate can contradict the reported global *p*-value (e.g., for the single-category effects of relationship with mother). In such cases, the reported confidence interval is more robust with corrected degrees of freedom from the pooling across multiple imputations, whereas the global *p*-value is based on a Wald-type test and is less robust to uncertainty attributable to multiple imputation. Third, comparing results across countries should be done with caution due to possible measurement non-invariance and differences in translation.

**Supplementary Tables – Charitable Giving and Helping**

**Table S1a.** Nationally representative descriptive statistics for Argentina

**Table S1b.** Childhood predictors regression analysis results for Argentina

**Table S1c.** Sensitivity to unmeasured confounding of childhood predictors in Argentina

**Table S2a.** Nationally representative descriptive statistics for Australia

**Table S2b.** Childhood predictors regression analysis results for Australia

**Table S2c.** Sensitivity to unmeasured confounding of childhood predictors in Australia

**Table S3a.** Nationally representative descriptive statistics for Brazil

**Table S3b.** Childhood predictors regression analysis results for Brazil

**Table S3c.** Sensitivity to unmeasured confounding of childhood predictors in Brazil

**Table S4a.** Nationally representative descriptive statistics for Egypt

**Table S4b.** Childhood predictors regression analysis results for Egypt

**Table S4c.** Sensitivity to unmeasured confounding of childhood predictors in Egypt

**Table S5a.** Nationally representative descriptive statistics for Germany

**Table S5b.** Childhood predictors regression analysis results for Germany

**Table S5c.** Sensitivity to unmeasured confounding of childhood predictors in Germany

**Table S6a.** Nationally representative descriptive statistics for Hong Kong

**Table S6b.** Childhood predictors regression analysis results for Hong Kong

**Table S6c.** Sensitivity to unmeasured confounding of childhood predictors in Hong Kong

**Table S7a.** Nationally representative descriptive statistics for India

**Table S7b.** Childhood predictors regression analysis results for India

**Table S7c.** Sensitivity to unmeasured confounding of childhood predictors in India

**Table S8a.** Nationally representative descriptive statistics for Indonesia

**Table S8b.** Childhood predictors regression analysis results for Indonesia

**Table S8c.** Sensitivity to unmeasured confounding of childhood predictors in Indonesia

**Table S9a.** Nationally representative descriptive statistics for Israel

**Table S9b.** Childhood predictors regression analysis results for Israel

**Table S9c.** Sensitivity to unmeasured confounding of childhood predictors in Israel

**Table S10a.** Nationally representative descriptive statistics for Japan

**Table S10b.** Childhood predictors regression analysis results for Japan

**Table S10c.** Sensitivity to unmeasured confounding of childhood predictors in Japan

**Table S11a.** Nationally representative descriptive statistics for Kenya

**Table S11b.** Childhood predictors regression analysis results for Kenya

**Table S11c.** Sensitivity to unmeasured confounding of childhood predictors in Kenya

**Table S12a.** Nationally representative descriptive statistics for Mexico

**Table S12b.** Childhood predictors regression analysis results for Mexico

**Table S12c.** Sensitivity to unmeasured confounding of childhood predictors in Mexico

**Table S13a.** Nationally representative descriptive statistics for Nigeria

**Table S13b.** Childhood predictors regression analysis results for Nigeria

**Table S13c.** Sensitivity to unmeasured confounding of childhood predictors in Nigeria

**Table S14a.** Nationally representative descriptive statistics for Philippines

**Table S14b.** Childhood predictors regression analysis results for Philippines

**Table S14c.** Sensitivity to unmeasured confounding of childhood predictors in Philippines

**Table S15a.** Nationally representative descriptive statistics for Poland

**Table S15b.** Childhood predictors regression analysis results for Poland

**Table S15c.** Sensitivity to unmeasured confounding of childhood predictors in Poland

**Table S16a.** Nationally representative descriptive statistics for South Africa

**Table S16b.** Childhood predictors regression analysis results for South Africa

**Table S16c.** Sensitivity to unmeasured confounding of childhood predictors in South Africa

**Table S17a.** Nationally representative descriptive statistics for Spain

**Table S17b.** Childhood predictors regression analysis results for Spain

**Table S17c.** Sensitivity to unmeasured confounding of childhood predictors in Spain

**Table S18a.** Nationally representative descriptive statistics for Sweden

**Table S18b.** Childhood predictors regression analysis results for Sweden

**Table S18c.** Sensitivity to unmeasured confounding of childhood predictors in Sweden

**Table S19a.** Nationally representative descriptive statistics for Tanzania

**Table S19b.** Childhood predictors regression analysis results for Tanzania

**Table S19c.** Sensitivity to unmeasured confounding of childhood predictors in Tanzania

**Table S20a.** Nationally representative descriptive statistics for Turkey

**Table S20b.** Childhood predictors regression analysis results for Turkey

**Table S20c.** Sensitivity to unmeasured confounding of childhood predictors in Turkey

**Table S21a.** Nationally representative descriptive statistics for United Kingdom

**Table S21b.** Childhood predictors regression analysis results for United Kingdom

**Table S21c.** Sensitivity to unmeasured confounding of childhood predictors in United Kingdom

**Table S22a.** Nationally representative descriptive statistics for United States

**Table S22b.** Childhood predictors regression analysis results for United States

**Table S22c.** Sensitivity to unmeasured confounding of childhood predictors in United States

**Table S23a.** Population weighted meta-analysis of regression results – charitable giving

**Table S23b.** Population weighted meta-analysis of regression results - helping

**Table S24a.** Population weighted meta-analysis of E-values – charitable giving

**Table S24b.** Population weighted meta-analysis of E-values - helping

**Supplementary Figures**

**Figure S1.** Forest plot for ‘Relationship with mother’ – ‘Very good/somewhat good’ effect

**Figure S2.** Forest plot for ‘Relationship with father’ – ‘Very good/somewhat good’ effect

**Figure S3.** Forest plot for ‘Parent marital status’ – ‘Divorced’ effect

**Figure S4.** Forest plot for ‘Parent marital status’ – ‘Single, never married’ effect

**Figure S5.** Forest plot for ‘Parent marital status’ – ‘One or both parents had died’ effect

**Figure S6.** Forest plot for ‘Subjective financial status of family growing up’ – ‘Lived comfortably’ effect

**Figure S7.** Forest plot for ‘Subjective financial status of family growing up’ – ‘Found it difficult’ effect

**Figure S8.** Forest plot for ‘Subjective financial status of family growing up’ – ‘Found it very difficult’ effect

**Figure S9.** Forest plot for ‘Abuse’ – ‘Yes’ effect

**Figure S10.** Forest plot for ‘Outsider growing up’ – ‘Yes’ effect

**Figure S11.** Forest plot for ‘Self-rated health growing up’ – ‘Excellent’ effect

**Figure S12.** Forest plot for ‘Self-rated health growing up’ – ‘Very good’ effect

**Figure S13.** Forest plot for ‘Self-rated health growing up’ – ‘Fair’ effect

**Figure S14.** Forest plot for ‘Self-rated health growing up’ – ‘Poor’ effect

**Figure S15.** Forest plot for ‘Immigration status’ – ‘Born in another country’ effect

**Figure S16.** Forest plot for ‘Age 12 religious service attendance’ – ‘At least 1/week’ effect

**Figure S17.** Forest plot for ‘Age 12 religious service attendance’ – ‘1-3/month’ effect

**Figure S18.** Forest plot for ‘Age 12 religious service attendance’ – ‘<1/month’ effect

**Figure S19.** Forest plot for ‘Year of birth’ – ‘1993-1998; age 25-29’ effect

**Figure S20.** Forest plot for ‘Year of birth’ – ‘1983-1993; age 30-39’ effect

**Figure S21.** Forest plot for ‘Year of birth’ – ‘1973-1983; age 40-49’ effect

**Figure S22.** Forest plot for ‘Year of birth’ – ‘1963-1973; age 50-59’ effect

**Figure S23.** Forest plot for ‘Year of birth’ – ‘1953-1963; age 60-69’ effect

**Figure S24.** Forest plot for ‘Year of birth’ – ‘1943-1953; age 70-79’ effect

**Figure S25.** Forest plot for ‘Year of birth’ – ‘1943 or earlier; age 80+’ effect

**Figure S26.** Forest plot for ‘Gender’ – ‘Female’ effect

**Figure S27.** Forest plot for ‘Gender’ – ‘Other’ effect

***Table S1a. Nationally representative descriptive statistics for Argentina***

| **Characteristic** | **N = 6,724**^1^ |
| --- | --- |
| **Relationship with mother** |  |
| Very good | 4,463 (66%) |
| Somewhat good | 1,436 (21%) |
| Somewhat bad | 299 (4.4%) |
| Very bad | 216 (3.2%) |
| Does not apply | 273 (4.1%) |
| (Missing) | 36 (0.5%) |
| **Relationship with father** |  |
| Very good | 3,612 (54%) |
| Somewhat good | 1,537 (23%) |
| Somewhat bad | 440 (6.5%) |
| Very bad | 401 (6.0%) |
| Does not apply | 694 (10%) |
| (Missing) | 39 (0.6%) |
| **Parent marital status** |  |
| Parents married | 4,110 (61%) |
| Divorced | 637 (9.5%) |
| Parents were never married | 1,368 (20%) |
| One or both parents had died | 199 (3.0%) |
| (Missing) | 410 (6.1%) |
| **Subjective financial status of family growing up** |  |
| Lived comfortably | 2,042 (30%) |
| Got by | 2,305 (34%) |
| Found it difficult | 1,789 (27%) |
| Found it very difficult | 569 (8.5%) |
| (Missing) | 19 (0.3%) |
| **Abuse** |  |
| Yes | 1,302 (19%) |
| No | 5,271 (78%) |
| (Missing) | 151 (2.2%) |
| **Outsider growing up** |  |
| Yes | 1,165 (17%) |
| No | 5,458 (81%) |
| (Missing) | 101 (1.5%) |
| **Self-rated health growing up** |  |
| Excellent | 2,402 (36%) |
| Very good | 1,819 (27%) |
| Good | 1,830 (27%) |
| Fair | 505 (7.5%) |
| Poor | 156 (2.3%) |
| (Missing) | 12 (0.2%) |
| **Immigration status** |  |
| Born in this country | 6,346 (94%) |
| Born in another country | 348 (5.2%) |
| (Missing) | 29 (0.4%) |
| **Age 12 religious service attendance** |  |
| At least 1/week | 2,601 (39%) |
| 1-3/month | 1,204 (18%) |
| <1/month | 1,059 (16%) |
| Never | 1,808 (27%) |
| (Missing) | 53 (0.8%) |
| **Year of birth** |  |
| 1998-2005; age 18-24 | 1,108 (16%) |
| 1993-1998; age 25-29 | 719 (11%) |
| 1983-1993; age 30-39 | 1,432 (21%) |
| 1973-1983; age 40-49 | 1,254 (19%) |
| 1963-1973; age 50-59 | 1,014 (15%) |
| 1953-1963; age 60-69 | 730 (11%) |
| 1943-1953; age 70-79 | 356 (5.3%) |
| 1943 or earlier; age 80+ | 112 (1.7%) |
| (Missing) | 0 (0%) |
| **Gender** |  |
| Male | 3,143 (47%) |
| Female | 3,542 (53%) |
| Other | 21 (0.3%) |
| (Missing) | 18 (0.3%) |
| **Religious affiliation** |  |
| Christianity | 5,805 (86%) |
| Islam | 11 (0.2%) |
| Hinduism | 2 (<0.1%) |
| Buddhism | 3 (<0.1%) |
| Judaism | 51 (0.8%) |
| Sikhism | 5 (<0.1%) |
| Baha'i | 0 (0%) |
| Jainism | 0 (0%) |
| Shinto | 0 (0%) |
| Taoism | 1 (<0.1%) |
| Confucianism | 0 (0%) |
| Primal, Animist, or Folk religion | 17 (0.2%) |
| Spiritism | 0 (0%) |
| Umbanda, Candomble, and other African-derived religions | 0 (0%) |
| Chinese folk/traditional religion | 0 (0%) |
| Some other religion | 10 (0.2%) |
| No religion/Atheist/Agnostic | 697 (10%) |
| (Missing) | 122 (1.8%) |
| **Race/Ethnicity** |  |
| Asian | 43 (0.6%) |
| Black | 95 (1.4%) |
| Indigenous | 129 (1.9%) |
| Mestizo(a) | 1,801 (27%) |
| Mullato(a) | 75 (1.1%) |
| Other | 104 (1.5%) |
| White | 3,406 (51%) |
| (Missing) | 1,070 (16%) |
| ^1^n (%) | |

***Table S1b. Childhood predictors regression analysis results for Argentina***

|  | | Charitable giving | | | | Helping | | | |
| --- | --- | --- | --- | --- | --- | --- | --- | --- | --- |
| Variable | Category | Risk-Ratio | RR 95% CI | log(RR) SE | Global p-value | Risk-Ratio | RR 95% CI | log(RR) SE | Global p-value |
| Relationship with mother | (Ref: Very bad/somewhat bad) |  |  |  | 0.476 |  |  |  | 0.070 |
|  | Very good/somewhat good | 1.10 | (0.84,1.43) | 0.13 |  | 1.09 | (0.99,1.20) | 0.05 |  |
| Relationship with father | (Ref: Very bad/somewhat bad) |  |  |  | 0.127 |  |  |  | 0.694 |
|  | Very good/somewhat good | 1.17 | (0.96,1.43) | 0.10 |  | 1.01 | (0.94,1.09) | 0.04 |  |
| Parent marital status | (Ref: Parents married) |  |  |  | 0.462 |  |  |  | 0.038 |
|  | Divorced | 1.04 | (0.83,1.31) | 0.12 |  | 1.06 | (0.98,1.15) | 0.04 |  |
|  | Parents were never married | 1.01 | (0.84,1.22) | 0.10 |  | 1.01 | (0.94,1.08) | 0.04 |  |
|  | One or both parents had died | 1.32 | (0.92,1.91) | 0.19 |  | 1.16 | (1.03,1.31) | 0.06 |  |
| Subjective financial status of family growing up | (Ref: Got by) |  |  |  | 0.074 |  |  |  | 0.051 |
|  | Lived comfortably | 1.14 | (0.98,1.34) | 0.08 |  | 0.97 | (0.91,1.03) | 0.03 |  |
|  | Found it difficult | 0.92 | (0.77,1.10) | 0.09 |  | 1.01 | (0.95,1.07) | 0.03 |  |
|  | Found it very difficult | 1.07 | (0.82,1.41) | 0.14 |  | 1.10 | (1.01,1.20) | 0.05 |  |
| Abuse | (Ref: No) |  |  |  | 0.096 |  |  |  | <.001 |
|  | Yes | 1.15 | (0.97,1.36) | 0.09 |  | 1.16 | (1.09,1.22) | 0.03 |  |
| Outsider growing up | (Ref: No) |  |  |  | 0.009 |  |  |  | 0.137 |
|  | Yes | 1.27 | (1.06,1.53) | 0.09 |  | 1.05 | (0.98,1.12) | 0.03 |  |
| Self-rated health growing up | (Ref: Good) |  |  |  | 0.408 |  |  |  | 0.021 |
|  | Excellent | 1.03 | (0.86,1.23) | 0.09 |  | 1.11 | (1.04,1.18) | 0.03 |  |
|  | Very good | 1.14 | (0.95,1.36) | 0.09 |  | 1.08 | (1.00,1.15) | 0.04 |  |
|  | Fair | 0.91 | (0.69,1.21) | 0.14 |  | 1.04 | (0.94,1.16) | 0.05 |  |
|  | Poor | 1.11 | (0.67,1.83) | 0.26 |  | 0.96 | (0.79,1.17) | 0.10 |  |
| Immigration status | (Ref: Born in this country) |  |  |  | 0.655 |  |  |  | 0.764 |
|  | Born in another country | 0.94 | (0.69,1.26) | 0.15 |  | 0.98 | (0.88,1.11) | 0.06 |  |
| Age 12 religious service attendance | (Ref: Never) |  |  |  | <.001 |  |  |  | <.001 |
|  | At least 1/week | 1.52 | (1.26,1.82) | 0.09 |  | 1.19 | (1.11,1.28) | 0.04 |  |
|  | 1-3/month | 1.40 | (1.13,1.74) | 0.11 |  | 1.18 | (1.09,1.28) | 0.04 |  |
|  | < 1/month | 1.27 | (1.02,1.59) | 0.11 |  | 1.20 | (1.11,1.30) | 0.04 |  |
| Year of birth | (Ref: 1998-2005; current age: 18-24) |  |  |  | 0.505 |  |  |  | 0.060 |
|  | 1993-1998; age 25-29 | 1.10 | (0.83,1.46) | 0.14 |  | 1.01 | (0.91,1.12) | 0.05 |  |
|  | 1983-1993; age 30-39 | 1.13 | (0.90,1.43) | 0.12 |  | 1.09 | (1.00,1.18) | 0.04 |  |
|  | 1973-1983; age 40-49 | 1.04 | (0.82,1.32) | 0.12 |  | 1.04 | (0.95,1.13) | 0.04 |  |
|  | 1963-1973; age 50-59 | 1.11 | (0.86,1.43) | 0.13 |  | 1.05 | (0.96,1.15) | 0.05 |  |
|  | 1953-1963; age 60-69 | 1.11 | (0.84,1.47) | 0.14 |  | 1.00 | (0.89,1.11) | 0.06 |  |
|  | 1943-1953; age 70-79 | 1.45 | (1.04,2.02) | 0.17 |  | 0.93 | (0.80,1.08) | 0.08 |  |
|  | 1943 or earlier; age 80+ | 1.29 | (0.79,2.09) | 0.25 |  | 0.77 | (0.57,1.04) | 0.15 |  |
| Gender | (Ref: Male) |  |  |  | 0.427 |  |  |  | 0.485 |
|  | Female | 1.00 | (0.88,1.14) | 0.07 |  | 0.98 | (0.93,1.04) | 0.03 |  |
|  | Other | 0.45 | (0.14,1.50) | 0.61 |  | 1.16 | (0.85,1.59) | 0.16 |  |
| Religious affiliation | (Ref: No religion/Atheist/Agnostic) |  |  |  | 0.293 |  |  |  | 0.289 |
|  | Christianity | 1.18 | (0.90,1.56) | 0.14 |  | 0.99 | (0.91,1.08) | 0.04 |  |
|  | Collapsed affiliations with prevalence<3% | 1.47 | (0.85,2.54) | 0.28 |  | 1.11 | (0.94,1.32) | 0.09 |  |
| Race/ethnicity | (Ref: Plurality group) |  |  |  | 0.023 |  |  |  | 0.166 |
|  | Non-plurality groups | 0.85 | (0.73,0.99) | 0.08 |  | 0.97 | (0.91,1.03) | 0.03 |  |

***Table S1c. Sensitivity to unmeasured confounding of childhood predictors in Argentina***

|  | | Charitable giving | | Helping | |
| --- | --- | --- | --- | --- | --- |
| Variable | Category | E-value for Estimate | E-value for 95% CI | E-value for Estimate | E-value for 95% CI |
| Relationship with mother | (Ref: Very bad/somewhat bad) |  |  |  |  |
|  | Very good/somewhat good | 1.43 | 1.00 | 1.41 | 1.00 |
| Relationship with father | (Ref: Very bad/somewhat bad) |  |  |  |  |
|  | Very good/somewhat good | 1.61 | 1.00 | 1.13 | 1.00 |
| Parent marital status | (Ref: Parents married) |  |  |  |  |
|  | Divorced | 1.24 | 1.00 | 1.30 | 1.00 |
|  | Parents were never married | 1.11 | 1.00 | 1.10 | 1.00 |
|  | One or both parents had died | 1.98 | 1.00 | 1.60 | 1.22 |
| Subjective financial status of family growing up | (Ref: Got by) |  |  |  |  |
|  | Lived comfortably | 1.55 | 1.00 | 1.22 | 1.00 |
|  | Found it difficult | 1.40 | 1.00 | 1.11 | 1.00 |
|  | Found it very difficult | 1.36 | 1.00 | 1.43 | 1.08 |
| Abuse | (Ref: No) |  |  |  |  |
|  | Yes | 1.56 | 1.00 | 1.58 | 1.41 |
| Outsider growing up | (Ref: No) |  |  |  |  |
|  | Yes | 1.86 | 1.30 | 1.28 | 1.00 |
| Self-rated health growing up | (Ref: Good) |  |  |  |  |
|  | Excellent | 1.20 | 1.00 | 1.46 | 1.25 |
|  | Very good | 1.54 | 1.00 | 1.36 | 1.07 |
|  | Fair | 1.42 | 1.00 | 1.26 | 1.00 |
|  | Poor | 1.45 | 1.00 | 1.24 | 1.00 |
| Immigration status | (Ref: Born in this country) |  |  |  |  |
|  | Born in another country | 1.34 | 1.00 | 1.14 | 1.00 |
| Age 12 religious service attendance | (Ref: Never) |  |  |  |  |
|  | At least 1/week | 2.40 | 1.84 | 1.66 | 1.45 |
|  | 1-3/month | 2.15 | 1.51 | 1.64 | 1.39 |
|  | < 1/month | 1.86 | 1.15 | 1.68 | 1.45 |
| Year of birth | (Ref: 1998-2005; current age: 18-24) |  |  |  |  |
|  | 1993-1998; age 25-29 | 1.42 | 1.00 | 1.11 | 1.00 |
|  | 1983-1993; age 30-39 | 1.53 | 1.00 | 1.39 | 1.05 |
|  | 1973-1983; age 40-49 | 1.24 | 1.00 | 1.23 | 1.00 |
|  | 1963-1973; age 50-59 | 1.45 | 1.00 | 1.28 | 1.00 |
|  | 1953-1963; age 60-69 | 1.47 | 1.00 | 1.07 | 1.00 |
|  | 1943-1953; age 70-79 | 2.26 | 1.26 | 1.36 | 1.00 |
|  | 1943 or earlier; age 80+ | 1.89 | 1.00 | 1.92 | 1.00 |
| Gender | (Ref: Male) |  |  |  |  |
|  | Female | 1.01 | 1.00 | 1.14 | 1.00 |
|  | Other | 3.86 | 1.00 | 1.60 | 1.00 |
| Religious affiliation | (Ref: No religion/Atheist/Agnostic) |  |  |  |  |
|  | Christianity | 1.64 | 1.00 | 1.09 | 1.00 |
|  | Collapsed affiliations with prevalence<3% | 2.30 | 1.00 | 1.47 | 1.00 |
| Race/ethnicity | (Ref: Plurality group) |  |  |  |  |
|  | Non-plurality groups | 1.64 | 1.10 | 1.22 | 1.00 |

***Table S2a. Nationally representative descriptive statistics for Australia***

| **Characteristic** | **N = 3,844**^1^ |
| --- | --- |
| **Relationship with mother** |  |
| Very good | 2,554 (66%) |
| Somewhat good | 925 (24%) |
| Somewhat bad | 218 (5.7%) |
| Very bad | 107 (2.8%) |
| Does not apply | 32 (0.8%) |
| (Missing) | 7 (0.2%) |
| **Relationship with father** |  |
| Very good | 2,032 (53%) |
| Somewhat good | 1,144 (30%) |
| Somewhat bad | 315 (8.2%) |
| Very bad | 196 (5.1%) |
| Does not apply | 148 (3.9%) |
| (Missing) | 9 (0.2%) |
| **Parent marital status** |  |
| Parents married | 3,048 (79%) |
| Divorced | 462 (12%) |
| Parents were never married | 187 (4.9%) |
| One or both parents had died | 96 (2.5%) |
| (Missing) | 52 (1.4%) |
| **Subjective financial status of family growing up** |  |
| Lived comfortably | 1,756 (46%) |
| Got by | 1,496 (39%) |
| Found it difficult | 422 (11%) |
| Found it very difficult | 154 (4.0%) |
| (Missing) | 16 (0.4%) |
| **Abuse** |  |
| Yes | 995 (26%) |
| No | 2,790 (73%) |
| (Missing) | 59 (1.5%) |
| **Outsider growing up** |  |
| Yes | 756 (20%) |
| No | 3,062 (80%) |
| (Missing) | 26 (0.7%) |
| **Self-rated health growing up** |  |
| Excellent | 1,736 (45%) |
| Very good | 1,087 (28%) |
| Good | 603 (16%) |
| Fair | 308 (8.0%) |
| Poor | 106 (2.8%) |
| (Missing) | 4 (<0.1%) |
| **Immigration status** |  |
| Born in this country | 2,953 (77%) |
| Born in another country | 885 (23%) |
| (Missing) | 6 (0.2%) |
| **Age 12 religious service attendance** |  |
| At least 1/week | 1,362 (35%) |
| 1-3/month | 486 (13%) |
| <1/month | 600 (16%) |
| Never | 1,307 (34%) |
| (Missing) | 90 (2.3%) |
| **Year of birth** |  |
| 1998-2005; age 18-24 | 345 (9.0%) |
| 1993-1998; age 25-29 | 282 (7.3%) |
| 1983-1993; age 30-39 | 641 (17%) |
| 1973-1983; age 40-49 | 618 (16%) |
| 1963-1973; age 50-59 | 691 (18%) |
| 1953-1963; age 60-69 | 589 (15%) |
| 1943-1953; age 70-79 | 498 (13%) |
| 1943 or earlier; age 80+ | 178 (4.6%) |
| (Missing) | 2 (<0.1%) |
| **Gender** |  |
| Male | 1,861 (48%) |
| Female | 1,941 (50%) |
| Other | 36 (0.9%) |
| (Missing) | 6 (0.2%) |
| **Religious affiliation** |  |
| Christianity | 2,678 (70%) |
| Islam | 48 (1.2%) |
| Hinduism | 39 (1.0%) |
| Buddhism | 16 (0.4%) |
| Judaism | 29 (0.8%) |
| Sikhism | 6 (0.2%) |
| Baha'i | 5 (0.1%) |
| Jainism | 0 (0%) |
| Shinto | 0 (0%) |
| Taoism | 1 (<0.1%) |
| Confucianism | 0 (0%) |
| Primal, Animist, or Folk religion | 4 (<0.1%) |
| Spiritism | 0 (0%) |
| Umbanda, Candomble, and other African-derived religions | 0 (0%) |
| Chinese folk/traditional religion | 0 (0%) |
| Some other religion | 8 (0.2%) |
| No religion/Atheist/Agnostic | 990 (26%) |
| (Missing) | 21 (0.5%) |
| **Race/Ethnicity** |  |
| Aboriginal | 53 (1.4%) |
| Australian | 1,946 (51%) |
| Australian British/European | 1,047 (27%) |
| Chinese | 75 (1.9%) |
| Indian | 58 (1.5%) |
| Japanese | 1 (<0.1%) |
| Malay | 11 (0.3%) |
| New Zealander | 91 (2.4%) |
| Other | 163 (4.2%) |
| Other European | 357 (9.3%) |
| Russian | 7 (0.2%) |
| Samoan | 4 (0.1%) |
| Sinhalese | 1 (<0.1%) |
| Spanish | 2 (<0.1%) |
| Sri Lankan Moor | 1 (<0.1%) |
| Sri Lankan Tamil | 7 (0.2%) |
| Vietnamese | 7 (0.2%) |
| (Missing) | 14 (0.4%) |
| ^1^n (%) | |

***Table S2b. Childhood predictors regression analysis results for Australia***

|  | | Charitable giving | | | | Helping | | | |
| --- | --- | --- | --- | --- | --- | --- | --- | --- | --- |
| Variable | Category | Risk-Ratio | RR 95% CI | log(RR) SE | Global p-value | Risk-Ratio | RR 95% CI | log(RR) SE | Global p-value |
| Relationship with mother | (Ref: Very bad/somewhat bad) |  |  |  | 0.316 |  |  |  | 0.070 |
|  | Very good/somewhat good | 1.09 | (0.92,1.28) | 0.08 |  | 0.91 | (0.82,1.01) | 0.05 |  |
| Relationship with father | (Ref: Very bad/somewhat bad) |  |  |  | 0.826 |  |  |  | 0.843 |
|  | Very good/somewhat good | 1.01 | (0.89,1.15) | 0.07 |  | 1.00 | (0.90,1.10) | 0.05 |  |
| Parent marital status | (Ref: Parents married) |  |  |  | 0.521 |  |  |  | 0.574 |
|  | Divorced | 0.93 | (0.80,1.07) | 0.07 |  | 0.99 | (0.89,1.10) | 0.05 |  |
|  | Parents were never married | 0.93 | (0.72,1.20) | 0.13 |  | 1.10 | (0.94,1.28) | 0.08 |  |
|  | One or both parents had died | 1.10 | (0.86,1.40) | 0.12 |  | 0.95 | (0.75,1.21) | 0.12 |  |
| Subjective financial status of family growing up | (Ref: Got by) |  |  |  | 0.204 |  |  |  | 0.185 |
|  | Lived comfortably | 1.00 | (0.92,1.08) | 0.04 |  | 0.97 | (0.90,1.04) | 0.04 |  |
|  | Found it difficult | 1.04 | (0.92,1.19) | 0.07 |  | 0.99 | (0.88,1.10) | 0.05 |  |
|  | Found it very difficult | 1.22 | (1.01,1.49) | 0.10 |  | 1.13 | (0.98,1.30) | 0.07 |  |
| Abuse | (Ref: No) |  |  |  | 0.264 |  |  |  | 0.001 |
|  | Yes | 1.05 | (0.96,1.15) | 0.05 |  | 1.12 | (1.05,1.21) | 0.04 |  |
| Outsider growing up | (Ref: No) |  |  |  | 0.038 |  |  |  | 0.831 |
|  | Yes | 0.88 | (0.78,0.99) | 0.06 |  | 1.01 | (0.93,1.10) | 0.04 |  |
| Self-rated health growing up | (Ref: Good) |  |  |  | 0.872 |  |  |  | 0.329 |
|  | Excellent | 1.03 | (0.92,1.16) | 0.06 |  | 1.06 | (0.97,1.17) | 0.05 |  |
|  | Very good | 1.02 | (0.90,1.15) | 0.06 |  | 0.99 | (0.89,1.09) | 0.05 |  |
|  | Fair | 0.95 | (0.78,1.14) | 0.10 |  | 1.01 | (0.88,1.15) | 0.07 |  |
|  | Poor | 1.02 | (0.77,1.36) | 0.14 |  | 1.07 | (0.87,1.31) | 0.10 |  |
| Immigration status | (Ref: Born in this country) |  |  |  | 0.138 |  |  |  | 0.637 |
|  | Born in another country | 1.07 | (0.98,1.17) | 0.05 |  | 0.98 | (0.90,1.07) | 0.04 |  |
| Age 12 religious service attendance | (Ref: Never) |  |  |  | 0.376 |  |  |  | <.001 |
|  | At least 1/week | 1.09 | (0.99,1.21) | 0.05 |  | 1.19 | (1.09,1.29) | 0.04 |  |
|  | 1-3/month | 1.05 | (0.92,1.20) | 0.07 |  | 1.20 | (1.08,1.34) | 0.05 |  |
|  | < 1/month | 1.07 | (0.95,1.22) | 0.06 |  | 1.05 | (0.94,1.17) | 0.06 |  |
| Year of birth | (Ref: 1998-2005; current age: 18-24) |  |  |  | 0.004 |  |  |  | <.001 |
|  | 1993-1998; age 25-29 | 1.47 | (1.11,1.94) | 0.14 |  | 0.92 | (0.78,1.08) | 0.08 |  |
|  | 1983-1993; age 30-39 | 1.43 | (1.12,1.83) | 0.12 |  | 0.90 | (0.78,1.02) | 0.07 |  |
|  | 1973-1983; age 40-49 | 1.54 | (1.21,1.96) | 0.12 |  | 0.91 | (0.80,1.04) | 0.07 |  |
|  | 1963-1973; age 50-59 | 1.48 | (1.17,1.88) | 0.12 |  | 0.88 | (0.78,1.00) | 0.06 |  |
|  | 1953-1963; age 60-69 | 1.40 | (1.10,1.77) | 0.12 |  | 0.79 | (0.69,0.90) | 0.07 |  |
|  | 1943-1953; age 70-79 | 1.55 | (1.22,1.98) | 0.12 |  | 0.69 | (0.60,0.80) | 0.08 |  |
|  | 1943 or earlier; age 80+ | 1.69 | (1.31,2.19) | 0.13 |  | 0.59 | (0.48,0.73) | 0.11 |  |
| Gender | (Ref: Male) |  |  |  | 0.001 |  |  |  | 0.071 |
|  | Female | 1.15 | (1.07,1.24) | 0.04 |  | 1.07 | (1.01,1.14) | 0.03 |  |
|  | Other | 0.96 | (0.54,1.70) | 0.29 |  | 0.93 | (0.66,1.32) | 0.18 |  |
| Religious affiliation | (Ref: No religion/Atheist/Agnostic) |  |  |  | 0.399 |  |  |  | 0.464 |
|  | Christianity | 1.08 | (0.96,1.21) | 0.06 |  | 1.00 | (0.91,1.09) | 0.04 |  |
|  | Collapsed affiliations with prevalence<3% | 1.02 | (0.82,1.28) | 0.11 |  | 0.89 | (0.74,1.09) | 0.10 |  |
| Race/ethnicity | (Ref: Plurality group) |  |  |  | 0.653 |  |  |  | 0.497 |
|  | Non-plurality groups | 0.98 | (0.90,1.07) | 0.04 |  | 1.02 | (0.96,1.09) | 0.03 |  |

***Table S2c. Sensitivity to unmeasured confounding of childhood predictors in Australia***

|  | | Charitable giving | | Helping | |
| --- | --- | --- | --- | --- | --- |
| Variable | Category | E-value for Estimate | E-value for 95% CI | E-value for Estimate | E-value for 95% CI |
| Relationship with mother | (Ref: Very bad/somewhat bad) |  |  |  |  |
|  | Very good/somewhat good | 1.39 | 1.00 | 1.43 | 1.00 |
| Relationship with father | (Ref: Very bad/somewhat bad) |  |  |  |  |
|  | Very good/somewhat good | 1.13 | 1.00 | 1.04 | 1.00 |
| Parent marital status | (Ref: Parents married) |  |  |  |  |
|  | Divorced | 1.38 | 1.00 | 1.09 | 1.00 |
|  | Parents were never married | 1.36 | 1.00 | 1.42 | 1.00 |
|  | One or both parents had died | 1.43 | 1.00 | 1.27 | 1.00 |
| Subjective financial status of family growing up | (Ref: Got by) |  |  |  |  |
|  | Lived comfortably | 1.07 | 1.00 | 1.21 | 1.00 |
|  | Found it difficult | 1.26 | 1.00 | 1.14 | 1.00 |
|  | Found it very difficult | 1.75 | 1.09 | 1.51 | 1.00 |
| Abuse | (Ref: No) |  |  |  |  |
|  | Yes | 1.28 | 1.00 | 1.49 | 1.26 |
| Outsider growing up | (Ref: No) |  |  |  |  |
|  | Yes | 1.53 | 1.09 | 1.10 | 1.00 |
| Self-rated health growing up | (Ref: Good) |  |  |  |  |
|  | Excellent | 1.22 | 1.00 | 1.32 | 1.00 |
|  | Very good | 1.17 | 1.00 | 1.13 | 1.00 |
|  | Fair | 1.30 | 1.00 | 1.09 | 1.00 |
|  | Poor | 1.17 | 1.00 | 1.35 | 1.00 |
| Immigration status | (Ref: Born in this country) |  |  |  |  |
|  | Born in another country | 1.35 | 1.00 | 1.16 | 1.00 |
| Age 12 religious service attendance | (Ref: Never) |  |  |  |  |
|  | At least 1/week | 1.41 | 1.00 | 1.66 | 1.40 |
|  | 1-3/month | 1.29 | 1.00 | 1.70 | 1.39 |
|  | < 1/month | 1.35 | 1.00 | 1.29 | 1.00 |
| Year of birth | (Ref: 1998-2005; current age: 18-24) |  |  |  |  |
|  | 1993-1998; age 25-29 | 2.30 | 1.47 | 1.40 | 1.00 |
|  | 1983-1993; age 30-39 | 2.22 | 1.49 | 1.48 | 1.00 |
|  | 1973-1983; age 40-49 | 2.46 | 1.72 | 1.41 | 1.00 |
|  | 1963-1973; age 50-59 | 2.33 | 1.61 | 1.53 | 1.05 |
|  | 1953-1963; age 60-69 | 2.14 | 1.43 | 1.84 | 1.45 |
|  | 1943-1953; age 70-79 | 2.48 | 1.73 | 2.25 | 1.80 |
|  | 1943 or earlier; age 80+ | 2.78 | 1.95 | 2.77 | 2.07 |
| Gender | (Ref: Male) |  |  |  |  |
|  | Female | 1.56 | 1.33 | 1.35 | 1.10 |
|  | Other | 1.26 | 1.00 | 1.35 | 1.00 |
| Religious affiliation | (Ref: No religion/Atheist/Agnostic) |  |  |  |  |
|  | Christianity | 1.37 | 1.00 | 1.05 | 1.00 |
|  | Collapsed affiliations with prevalence<3% | 1.18 | 1.00 | 1.48 | 1.00 |
| Race/ethnicity | (Ref: Plurality group) |  |  |  |  |
|  | Non-plurality groups | 1.14 | 1.00 | 1.17 | 1.00 |

***Table S3a. Nationally representative descriptive statistics for Brazil***

| **Characteristic** | **N = 13,204**^1^ |
| --- | --- |
| **Relationship with mother** |  |
| Very good | 8,369 (63%) |
| Somewhat good | 3,559 (27%) |
| Somewhat bad | 483 (3.7%) |
| Very bad | 214 (1.6%) |
| Does not apply | 507 (3.8%) |
| (Missing) | 73 (0.6%) |
| **Relationship with father** |  |
| Very good | 6,364 (48%) |
| Somewhat good | 3,654 (28%) |
| Somewhat bad | 1,035 (7.8%) |
| Very bad | 756 (5.7%) |
| Does not apply | 1,303 (9.9%) |
| (Missing) | 93 (0.7%) |
| **Parent marital status** |  |
| Parents married | 8,546 (65%) |
| Divorced | 1,384 (10%) |
| Parents were never married | 1,985 (15%) |
| One or both parents had died | 508 (3.8%) |
| (Missing) | 781 (5.9%) |
| **Subjective financial status of family growing up** |  |
| Lived comfortably | 4,998 (38%) |
| Got by | 4,616 (35%) |
| Found it difficult | 2,484 (19%) |
| Found it very difficult | 1,027 (7.8%) |
| (Missing) | 79 (0.6%) |
| **Abuse** |  |
| Yes | 2,606 (20%) |
| No | 10,147 (77%) |
| (Missing) | 451 (3.4%) |
| **Outsider growing up** |  |
| Yes | 1,659 (13%) |
| No | 11,234 (85%) |
| (Missing) | 311 (2.4%) |
| **Self-rated health growing up** |  |
| Excellent | 5,312 (40%) |
| Very good | 3,392 (26%) |
| Good | 2,873 (22%) |
| Fair | 1,368 (10%) |
| Poor | 228 (1.7%) |
| (Missing) | 30 (0.2%) |
| **Immigration status** |  |
| Born in this country | 12,688 (96%) |
| Born in another country | 153 (1.2%) |
| (Missing) | 363 (2.7%) |
| **Age 12 religious service attendance** |  |
| At least 1/week | 6,306 (48%) |
| 1-3/month | 2,491 (19%) |
| <1/month | 2,629 (20%) |
| Never | 1,707 (13%) |
| (Missing) | 71 (0.5%) |
| **Year of birth** |  |
| 1998-2005; age 18-24 | 1,986 (15%) |
| 1993-1998; age 25-29 | 1,468 (11%) |
| 1983-1993; age 30-39 | 2,908 (22%) |
| 1973-1983; age 40-49 | 2,638 (20%) |
| 1963-1973; age 50-59 | 2,131 (16%) |
| 1953-1963; age 60-69 | 1,435 (11%) |
| 1943-1953; age 70-79 | 510 (3.9%) |
| 1943 or earlier; age 80+ | 126 (1.0%) |
| (Missing) | 0 (0%) |
| **Gender** |  |
| Male | 6,320 (48%) |
| Female | 6,820 (52%) |
| Other | 35 (0.3%) |
| (Missing) | 30 (0.2%) |
| **Religious affiliation** |  |
| Christianity | 11,403 (86%) |
| Islam | 15 (0.1%) |
| Hinduism | 1 (<0.1%) |
| Buddhism | 27 (0.2%) |
| Judaism | 40 (0.3%) |
| Sikhism | 0 (0%) |
| Baha'i | 1 (<0.1%) |
| Jainism | 4 (<0.1%) |
| Shinto | 4 (<0.1%) |
| Taoism | 1 (<0.1%) |
| Confucianism | 7 (<0.1%) |
| Primal, Animist, or Folk religion | 17 (0.1%) |
| Spiritism | 336 (2.5%) |
| Umbanda, Candomble, and other African-derived religions | 262 (2.0%) |
| Chinese folk/traditional religion | 0 (0%) |
| Some other religion | 87 (0.7%) |
| No religion/Atheist/Agnostic | 908 (6.9%) |
| (Missing) | 94 (0.7%) |
| **Race/Ethnicity** |  |
| Amarela | 238 (1.8%) |
| Branca | 5,169 (39%) |
| Indígena | 131 (1.0%) |
| Other | 61 (0.5%) |
| Parda | 5,125 (39%) |
| Preta | 1,615 (12%) |
| (Missing) | 865 (6.6%) |
| ^1^n (%) | |

***Table S3b. Childhood predictors regression analysis results for Brazil***

|  | | Charitable giving | | | | Helping | | | |
| --- | --- | --- | --- | --- | --- | --- | --- | --- | --- |
| Variable | Category | Risk-Ratio | RR 95% CI | log(RR) SE | Global p-value | Risk-Ratio | RR 95% CI | log(RR) SE | Global p-value |
| Relationship with mother | (Ref: Very bad/somewhat bad) |  |  |  | 0.640 |  |  |  | 0.835 |
|  | Very good/somewhat good | 0.97 | (0.85,1.11) | 0.07 |  | 1.00 | (0.94,1.07) | 0.03 |  |
| Relationship with father | (Ref: Very bad/somewhat bad) |  |  |  | 0.556 |  |  |  | 0.399 |
|  | Very good/somewhat good | 1.03 | (0.93,1.13) | 0.05 |  | 1.02 | (0.97,1.07) | 0.02 |  |
| Parent marital status | (Ref: Parents married) |  |  |  | 0.304 |  |  |  | 0.400 |
|  | Divorced | 0.94 | (0.84,1.05) | 0.06 |  | 1.03 | (0.98,1.09) | 0.03 |  |
|  | Parents were never married | 0.93 | (0.83,1.04) | 0.06 |  | 1.02 | (0.97,1.07) | 0.03 |  |
|  | One or both parents had died | 0.89 | (0.73,1.08) | 0.10 |  | 1.05 | (0.96,1.15) | 0.04 |  |
| Subjective financial status of family growing up | (Ref: Got by) |  |  |  | 0.390 |  |  |  | 0.700 |
|  | Lived comfortably | 1.03 | (0.96,1.12) | 0.04 |  | 1.00 | (0.97,1.04) | 0.02 |  |
|  | Found it difficult | 0.95 | (0.86,1.05) | 0.05 |  | 0.99 | (0.94,1.03) | 0.02 |  |
|  | Found it very difficult | 0.97 | (0.84,1.11) | 0.07 |  | 0.97 | (0.90,1.04) | 0.04 |  |
| Abuse | (Ref: No) |  |  |  | 0.010 |  |  |  | 0.002 |
|  | Yes | 1.12 | (1.03,1.21) | 0.04 |  | 1.07 | (1.02,1.11) | 0.02 |  |
| Outsider growing up | (Ref: No) |  |  |  | 0.406 |  |  |  | 0.019 |
|  | Yes | 1.04 | (0.94,1.15) | 0.05 |  | 1.06 | (1.01,1.11) | 0.02 |  |
| Self-rated health growing up | (Ref: Good) |  |  |  | 0.367 |  |  |  | 0.007 |
|  | Excellent | 1.06 | (0.97,1.17) | 0.05 |  | 1.06 | (1.02,1.11) | 0.02 |  |
|  | Very good | 1.06 | (0.96,1.16) | 0.05 |  | 1.06 | (1.01,1.11) | 0.02 |  |
|  | Fair | 0.96 | (0.84,1.09) | 0.07 |  | 0.97 | (0.91,1.04) | 0.03 |  |
|  | Poor | 1.07 | (0.83,1.40) | 0.13 |  | 1.02 | (0.90,1.16) | 0.06 |  |
| Immigration status | (Ref: Born in this country) |  |  |  | 0.768 |  |  |  | 0.593 |
|  | Born in another country | 1.04 | (0.77,1.42) | 0.16 |  | 0.96 | (0.80,1.13) | 0.09 |  |
| Age 12 religious service attendance | (Ref: Never) |  |  |  | <.001 |  |  |  | <.001 |
|  | At least 1/week | 1.38 | (1.22,1.57) | 0.06 |  | 1.14 | (1.07,1.21) | 0.03 |  |
|  | 1-3/month | 1.40 | (1.23,1.60) | 0.07 |  | 1.15 | (1.08,1.23) | 0.03 |  |
|  | < 1/month | 1.13 | (0.99,1.30) | 0.07 |  | 1.11 | (1.03,1.18) | 0.03 |  |
| Year of birth | (Ref: 1998-2005; current age: 18-24) |  |  |  | <.001 |  |  |  | <.001 |
|  | 1993-1998; age 25-29 | 1.26 | (1.09,1.46) | 0.07 |  | 1.08 | (1.01,1.15) | 0.03 |  |
|  | 1983-1993; age 30-39 | 1.46 | (1.30,1.65) | 0.06 |  | 1.08 | (1.02,1.14) | 0.03 |  |
|  | 1973-1983; age 40-49 | 1.73 | (1.53,1.96) | 0.06 |  | 1.12 | (1.05,1.18) | 0.03 |  |
|  | 1963-1973; age 50-59 | 1.75 | (1.54,2.00) | 0.07 |  | 1.15 | (1.08,1.22) | 0.03 |  |
|  | 1953-1963; age 60-69 | 2.20 | (1.90,2.55) | 0.07 |  | 1.12 | (1.04,1.20) | 0.04 |  |
|  | 1943-1953; age 70-79 | 2.81 | (2.36,3.35) | 0.09 |  | 1.11 | (0.99,1.25) | 0.06 |  |
|  | 1943 or earlier; age 80+ | 3.24 | (2.53,4.16) | 0.13 |  | 1.01 | (0.80,1.28) | 0.12 |  |
| Gender | (Ref: Male) |  |  |  | 0.498 |  |  |  | 0.011 |
|  | Female | 0.99 | (0.93,1.06) | 0.03 |  | 0.95 | (0.92,0.98) | 0.02 |  |
|  | Other | 0.64 | (0.29,1.38) | 0.39 |  | 0.92 | (0.66,1.26) | 0.16 |  |
| Religious affiliation | (Ref: No religion/Atheist/Agnostic) |  |  |  | 0.449 |  |  |  | 0.858 |
|  | Christianity | 1.02 | (0.88,1.18) | 0.08 |  | 0.98 | (0.92,1.05) | 0.03 |  |
|  | Collapsed affiliations with prevalence<3% | 1.11 | (0.91,1.34) | 0.10 |  | 0.98 | (0.90,1.08) | 0.05 |  |
| Race/ethnicity | (Ref: Plurality group) |  |  |  | 0.017 |  |  |  | 0.007 |
|  | Non-plurality groups | 0.92 | (0.86,0.99) | 0.04 |  | 1.05 | (1.01,1.09) | 0.02 |  |

***Table S3c. Sensitivity to unmeasured confounding of childhood predictors in Brazil***

|  | | Charitable giving | | Helping | |
| --- | --- | --- | --- | --- | --- |
| Variable | Category | E-value for Estimate | E-value for 95% CI | E-value for Estimate | E-value for 95% CI |
| Relationship with mother | (Ref: Very bad/somewhat bad) |  |  |  |  |
|  | Very good/somewhat good | 1.20 | 1.00 | 1.05 | 1.00 |
| Relationship with father | (Ref: Very bad/somewhat bad) |  |  |  |  |
|  | Very good/somewhat good | 1.20 | 1.00 | 1.16 | 1.00 |
| Parent marital status | (Ref: Parents married) |  |  |  |  |
|  | Divorced | 1.33 | 1.00 | 1.22 | 1.00 |
|  | Parents were never married | 1.37 | 1.00 | 1.15 | 1.00 |
|  | One or both parents had died | 1.50 | 1.00 | 1.29 | 1.00 |
| Subjective financial status of family growing up | (Ref: Got by) |  |  |  |  |
|  | Lived comfortably | 1.22 | 1.00 | 1.07 | 1.00 |
|  | Found it difficult | 1.28 | 1.00 | 1.13 | 1.00 |
|  | Found it very difficult | 1.21 | 1.00 | 1.22 | 1.00 |
| Abuse | (Ref: No) |  |  |  |  |
|  | Yes | 1.47 | 1.19 | 1.33 | 1.18 |
| Outsider growing up | (Ref: No) |  |  |  |  |
|  | Yes | 1.24 | 1.00 | 1.30 | 1.10 |
| Self-rated health growing up | (Ref: Good) |  |  |  |  |
|  | Excellent | 1.33 | 1.00 | 1.32 | 1.14 |
|  | Very good | 1.30 | 1.00 | 1.31 | 1.10 |
|  | Fair | 1.27 | 1.00 | 1.21 | 1.00 |
|  | Poor | 1.36 | 1.00 | 1.16 | 1.00 |
| Immigration status | (Ref: Born in this country) |  |  |  |  |
|  | Born in another country | 1.26 | 1.00 | 1.27 | 1.00 |
| Age 12 religious service attendance | (Ref: Never) |  |  |  |  |
|  | At least 1/week | 2.11 | 1.74 | 1.53 | 1.34 |
|  | 1-3/month | 2.15 | 1.75 | 1.57 | 1.36 |
|  | < 1/month | 1.52 | 1.00 | 1.45 | 1.22 |
| Year of birth | (Ref: 1998-2005; current age: 18-24) |  |  |  |  |
|  | 1993-1998; age 25-29 | 1.83 | 1.40 | 1.37 | 1.13 |
|  | 1983-1993; age 30-39 | 2.29 | 1.92 | 1.37 | 1.18 |
|  | 1973-1983; age 40-49 | 2.86 | 2.44 | 1.47 | 1.30 |
|  | 1963-1973; age 50-59 | 2.90 | 2.44 | 1.57 | 1.39 |
|  | 1953-1963; age 60-69 | 3.83 | 3.22 | 1.47 | 1.23 |
|  | 1943-1953; age 70-79 | 5.07 | 4.16 | 1.47 | 1.00 |
|  | 1943 or earlier; age 80+ | 5.94 | 4.49 | 1.12 | 1.00 |
| Gender | (Ref: Male) |  |  |  |  |
|  | Female | 1.09 | 1.00 | 1.29 | 1.15 |
|  | Other | 2.52 | 1.00 | 1.41 | 1.00 |
| Religious affiliation | (Ref: No religion/Atheist/Agnostic) |  |  |  |  |
|  | Christianity | 1.15 | 1.00 | 1.16 | 1.00 |
|  | Collapsed affiliations with prevalence<3% | 1.45 | 1.00 | 1.14 | 1.00 |
| Race/ethnicity | (Ref: Plurality group) |  |  |  |  |
|  | Non-plurality groups | 1.39 | 1.13 | 1.27 | 1.12 |

***Table S4a. Nationally representative descriptive statistics for Egypt***

| **Characteristic** | **N = 4,729**^1^ |
| --- | --- |
| **Relationship with mother** |  |
| Very good | 4,110 (87%) |
| Somewhat good | 505 (11%) |
| Somewhat bad | 21 (0.4%) |
| Very bad | 10 (0.2%) |
| Does not apply | 83 (1.8%) |
| (Missing) | 0 (0%) |
| **Relationship with father** |  |
| Very good | 3,713 (79%) |
| Somewhat good | 683 (14%) |
| Somewhat bad | 56 (1.2%) |
| Very bad | 30 (0.6%) |
| Does not apply | 233 (4.9%) |
| (Missing) | 14 (0.3%) |
| **Parent marital status** |  |
| Parents married | 4,049 (86%) |
| Divorced | 131 (2.8%) |
| Parents were never married | 9 (0.2%) |
| One or both parents had died | 485 (10%) |
| (Missing) | 55 (1.2%) |
| **Subjective financial status of family growing up** |  |
| Lived comfortably | 1,251 (26%) |
| Got by | 2,352 (50%) |
| Found it difficult | 857 (18%) |
| Found it very difficult | 268 (5.7%) |
| (Missing) | 1 (<0.1%) |
| **Abuse** |  |
| Yes | 405 (8.6%) |
| No | 4,293 (91%) |
| (Missing) | 30 (0.6%) |
| **Outsider growing up** |  |
| Yes | 260 (5.5%) |
| No | 4,456 (94%) |
| (Missing) | 13 (0.3%) |
| **Self-rated health growing up** |  |
| Excellent | 2,687 (57%) |
| Very good | 1,174 (25%) |
| Good | 497 (11%) |
| Fair | 265 (5.6%) |
| Poor | 106 (2.2%) |
| (Missing) | 1 (<0.1%) |
| **Immigration status** |  |
| Born in this country | 4,713 (100%) |
| Born in another country | 16 (0.3%) |
| (Missing) | 1 (<0.1%) |
| **Age 12 religious service attendance** |  |
| At least 1/week | 2,307 (49%) |
| 1-3/month | 570 (12%) |
| <1/month | 629 (13%) |
| Never | 1,165 (25%) |
| (Missing) | 57 (1.2%) |
| **Year of birth** |  |
| 1998-2005; age 18-24 | 960 (20%) |
| 1993-1998; age 25-29 | 607 (13%) |
| 1983-1993; age 30-39 | 1,204 (25%) |
| 1973-1983; age 40-49 | 897 (19%) |
| 1963-1973; age 50-59 | 613 (13%) |
| 1953-1963; age 60-69 | 387 (8.2%) |
| 1943-1953; age 70-79 | 54 (1.1%) |
| 1943 or earlier; age 80+ | 7 (0.2%) |
| (Missing) | 0 (0%) |
| **Gender** |  |
| Male | 2,394 (51%) |
| Female | 2,334 (49%) |
| Other | 0 (0%) |
| (Missing) | 0 (<0.1%) |
| **Religious affiliation** |  |
| Christianity | 123 (2.6%) |
| Islam | 4,602 (97%) |
| Hinduism | 0 (0%) |
| Buddhism | 0 (0%) |
| Judaism | 0 (0%) |
| Sikhism | 0 (0%) |
| Baha'i | 0 (0%) |
| Jainism | 1 (<0.1%) |
| Shinto | 0 (0%) |
| Taoism | 0 (<0.1%) |
| Confucianism | 0 (0%) |
| Primal, Animist, or Folk religion | 0 (0%) |
| Spiritism | 0 (0%) |
| Umbanda, Candomble, and other African-derived religions | 0 (0%) |
| Chinese folk/traditional religion | 0 (0%) |
| Some other religion | 0 (0%) |
| No religion/Atheist/Agnostic | 0 (0%) |
| (Missing) | 3 (<0.1%) |
| **Race/Ethnicity** |  |
| Arab | 4,585 (97%) |
| Bedouin Arab | 4 (<0.1%) |
| Greek | 1 (<0.1%) |
| Nubian | 27 (0.6%) |
| Turkish | 9 (0.2%) |
| (Missing) | 102 (2.2%) |
| ^1^n (%) | |

***Table S4b. Childhood predictors regression analysis results for Egypt***

|  | | Charitable giving | | | | Helping | | | |
| --- | --- | --- | --- | --- | --- | --- | --- | --- | --- |
| Variable | Category | Risk-Ratio | RR 95% CI | log(RR) SE | Global p-value | Risk-Ratio | RR 95% CI | log(RR) SE | Global p-value |
| Relationship with mother | (Ref: Very bad/somewhat bad) |  |  |  | 0.210 |  |  |  | 0.678 |
|  | Very good/somewhat good | 0.88 | (0.72,1.08) | 0.10 |  | 0.96 | (0.81,1.15) | 0.09 |  |
| Relationship with father | (Ref: Very bad/somewhat bad) |  |  |  | 0.076 |  |  |  | 0.503 |
|  | Very good/somewhat good | 1.18 | (0.98,1.41) | 0.09 |  | 1.03 | (0.90,1.18) | 0.07 |  |
| Parent marital status | (Ref: Parents married) |  |  |  | 0.217 |  |  |  | 0.329 |
|  | Divorced | 0.92 | (0.76,1.12) | 0.10 |  | 1.08 | (0.96,1.22) | 0.06 |  |
|  | Parents were never married | 1.39 | (1.02,1.90) | 0.16 |  | 1.18 | (0.93,1.50) | 0.12 |  |
|  | One or both parents had died | 0.97 | (0.87,1.09) | 0.06 |  | 1.01 | (0.92,1.09) | 0.04 |  |
| Subjective financial status of family growing up | (Ref: Got by) |  |  |  | <.001 |  |  |  | <.001 |
|  | Lived comfortably | 1.15 | (1.08,1.22) | 0.03 |  | 1.08 | (1.02,1.14) | 0.03 |  |
|  | Found it difficult | 0.83 | (0.75,0.91) | 0.05 |  | 0.92 | (0.86,0.99) | 0.03 |  |
|  | Found it very difficult | 0.91 | (0.79,1.06) | 0.08 |  | 1.02 | (0.94,1.10) | 0.04 |  |
| Abuse | (Ref: No) |  |  |  | 0.359 |  |  |  | 0.652 |
|  | Yes | 0.95 | (0.85,1.06) | 0.06 |  | 1.01 | (0.95,1.09) | 0.04 |  |
| Outsider growing up | (Ref: No) |  |  |  | 0.024 |  |  |  | 0.267 |
|  | Yes | 1.13 | (1.02,1.26) | 0.06 |  | 1.05 | (0.96,1.15) | 0.05 |  |
| Self-rated health growing up | (Ref: Good) |  |  |  | 0.727 |  |  |  | <.001 |
|  | Excellent | 0.97 | (0.86,1.09) | 0.06 |  | 1.07 | (0.99,1.15) | 0.04 |  |
|  | Very good | 0.94 | (0.84,1.06) | 0.06 |  | 0.96 | (0.87,1.04) | 0.05 |  |
|  | Fair | 0.94 | (0.81,1.10) | 0.08 |  | 1.14 | (1.02,1.27) | 0.05 |  |
|  | Poor | 1.04 | (0.87,1.25) | 0.09 |  | 0.94 | (0.78,1.14) | 0.10 |  |
| Immigration status | (Ref: Born in this country) |  |  |  | <.001 |  |  |  | 0.002 |
|  | Born in another country | 1.50 | (1.24,1.81) | 0.10 |  | 1.24 | (1.08,1.41) | 0.07 |  |
| Age 12 religious service attendance | (Ref: Never) |  |  |  | <.001 |  |  |  | <.001 |
|  | At least 1/week | 1.30 | (1.20,1.41) | 0.04 |  | 1.20 | (1.13,1.27) | 0.03 |  |
|  | 1-3/month | 1.33 | (1.21,1.47) | 0.05 |  | 1.16 | (1.06,1.26) | 0.04 |  |
|  | < 1/month | 1.17 | (1.06,1.30) | 0.05 |  | 1.05 | (0.97,1.14) | 0.04 |  |
| Year of birth | (Ref: 1998-2005; current age: 18-24) |  |  |  | <.001 |  |  |  | 0.024 |
|  | 1993-1998; age 25-29 | 1.29 | (1.15,1.46) | 0.06 |  | 1.11 | (1.03,1.20) | 0.04 |  |
|  | 1983-1993; age 30-39 | 1.33 | (1.21,1.47) | 0.05 |  | 1.11 | (1.04,1.19) | 0.03 |  |
|  | 1973-1983; age 40-49 | 1.27 | (1.14,1.42) | 0.06 |  | 1.12 | (1.03,1.22) | 0.04 |  |
|  | 1963-1973; age 50-59 | 1.26 | (1.12,1.42) | 0.06 |  | 1.15 | (1.07,1.23) | 0.04 |  |
|  | 1953-1963; age 60-69 | 1.35 | (1.19,1.54) | 0.06 |  | 1.09 | (0.98,1.21) | 0.05 |  |
|  | 1943-1953; age 70-79 | 1.26 | (0.92,1.71) | 0.16 |  | 0.89 | (0.67,1.18) | 0.14 |  |
|  | 1943 or earlier; age 80+ | 1.47 | (0.76,2.84) | 0.34 |  | 1.30 | (0.98,1.72) | 0.14 |  |
| Gender | (Ref: Male) |  |  |  | 0.410 |  |  |  | 0.245 |
|  | Female | 0.97 | (0.92,1.04) | 0.03 |  | 0.97 | (0.93,1.02) | 0.02 |  |
| Religious affiliation | (Ref: Islam) |  |  |  | 0.070 |  |  |  | 0.033 |
|  | Collapsed affiliations with prevalence<3% | 0.78 | (0.60,1.03) | 0.14 |  | 0.77 | (0.60,0.99) | 0.13 |  |
| Race/ethnicity | (Ref: Plurality group) |  |  |  | 0.544 |  |  |  | 0.356 |
|  | Non-plurality groups | 0.86 | (0.52,1.42) | 0.26 |  | 0.78 | (0.45,1.33) | 0.27 |  |

***Table S4c. Sensitivity to unmeasured confounding of childhood predictors in Egypt***

|  | | Charitable giving | | Helping | |
| --- | --- | --- | --- | --- | --- |
| Variable | Category | E-value for Estimate | E-value for 95% CI | E-value for Estimate | E-value for 95% CI |
| Relationship with mother | (Ref: Very bad/somewhat bad) |  |  |  |  |
|  | Very good/somewhat good | 1.52 | 1.00 | 1.23 | 1.00 |
| Relationship with father | (Ref: Very bad/somewhat bad) |  |  |  |  |
|  | Very good/somewhat good | 1.63 | 1.00 | 1.22 | 1.00 |
| Parent marital status | (Ref: Parents married) |  |  |  |  |
|  | Divorced | 1.40 | 1.00 | 1.38 | 1.00 |
|  | Parents were never married | 2.13 | 1.16 | 1.64 | 1.00 |
|  | One or both parents had died | 1.21 | 1.00 | 1.08 | 1.00 |
| Subjective financial status of family growing up | (Ref: Got by) |  |  |  |  |
|  | Lived comfortably | 1.56 | 1.37 | 1.37 | 1.17 |
|  | Found it difficult | 1.71 | 1.42 | 1.39 | 1.13 |
|  | Found it very difficult | 1.41 | 1.00 | 1.15 | 1.00 |
| Abuse | (Ref: No) |  |  |  |  |
|  | Yes | 1.29 | 1.00 | 1.14 | 1.00 |
| Outsider growing up | (Ref: No) |  |  |  |  |
|  | Yes | 1.52 | 1.15 | 1.29 | 1.00 |
| Self-rated health growing up | (Ref: Good) |  |  |  |  |
|  | Excellent | 1.23 | 1.00 | 1.33 | 1.00 |
|  | Very good | 1.31 | 1.00 | 1.27 | 1.00 |
|  | Fair | 1.31 | 1.00 | 1.53 | 1.17 |
|  | Poor | 1.26 | 1.00 | 1.31 | 1.00 |
| Immigration status | (Ref: Born in this country) |  |  |  |  |
|  | Born in another country | 2.36 | 1.78 | 1.78 | 1.38 |
| Age 12 religious service attendance | (Ref: Never) |  |  |  |  |
|  | At least 1/week | 1.92 | 1.68 | 1.69 | 1.52 |
|  | 1-3/month | 2.00 | 1.70 | 1.58 | 1.31 |
|  | < 1/month | 1.62 | 1.30 | 1.28 | 1.00 |
| Year of birth | (Ref: 1998-2005; current age: 18-24) |  |  |  |  |
|  | 1993-1998; age 25-29 | 1.91 | 1.57 | 1.47 | 1.20 |
|  | 1983-1993; age 30-39 | 1.99 | 1.71 | 1.46 | 1.23 |
|  | 1973-1983; age 40-49 | 1.86 | 1.53 | 1.49 | 1.22 |
|  | 1963-1973; age 50-59 | 1.84 | 1.48 | 1.56 | 1.34 |
|  | 1953-1963; age 60-69 | 2.05 | 1.67 | 1.39 | 1.00 |
|  | 1943-1953; age 70-79 | 1.82 | 1.00 | 1.50 | 1.00 |
|  | 1943 or earlier; age 80+ | 2.31 | 1.00 | 1.92 | 1.00 |
| Gender | (Ref: Male) |  |  |  |  |
|  | Female | 1.19 | 1.00 | 1.20 | 1.00 |
| Religious affiliation | (Ref: Islam) |  |  |  |  |
|  | Collapsed affiliations with prevalence<3% | 1.87 | 1.00 | 1.92 | 1.14 |
| Race/ethnicity | (Ref: Plurality group) |  |  |  |  |
|  | Non-plurality groups | 1.61 | 1.00 | 1.90 | 1.00 |

***Table S5a. Nationally representative descriptive statistics for Germany***

| **Characteristic** | **N = 9,506**^1^ |
| --- | --- |
| **Relationship with mother** |  |
| Very good | 5,497 (58%) |
| Somewhat good | 3,031 (32%) |
| Somewhat bad | 496 (5.2%) |
| Very bad | 187 (2.0%) |
| Does not apply | 241 (2.5%) |
| (Missing) | 54 (0.6%) |
| **Relationship with father** |  |
| Very good | 4,652 (49%) |
| Somewhat good | 3,012 (32%) |
| Somewhat bad | 846 (8.9%) |
| Very bad | 385 (4.0%) |
| Does not apply | 538 (5.7%) |
| (Missing) | 73 (0.8%) |
| **Parent marital status** |  |
| Parents married | 7,620 (80%) |
| Divorced | 927 (9.8%) |
| Parents were never married | 578 (6.1%) |
| One or both parents had died | 245 (2.6%) |
| (Missing) | 136 (1.4%) |
| **Subjective financial status of family growing up** |  |
| Lived comfortably | 3,177 (33%) |
| Got by | 4,508 (47%) |
| Found it difficult | 1,481 (16%) |
| Found it very difficult | 314 (3.3%) |
| (Missing) | 26 (0.3%) |
| **Abuse** |  |
| Yes | 1,086 (11%) |
| No | 8,321 (88%) |
| (Missing) | 99 (1.0%) |
| **Outsider growing up** |  |
| Yes | 1,105 (12%) |
| No | 8,262 (87%) |
| (Missing) | 139 (1.5%) |
| **Self-rated health growing up** |  |
| Excellent | 2,633 (28%) |
| Very good | 3,518 (37%) |
| Good | 2,582 (27%) |
| Fair | 612 (6.4%) |
| Poor | 134 (1.4%) |
| (Missing) | 26 (0.3%) |
| **Immigration status** |  |
| Born in this country | 8,722 (92%) |
| Born in another country | 744 (7.8%) |
| (Missing) | 40 (0.4%) |
| **Age 12 religious service attendance** |  |
| At least 1/week | 1,943 (20%) |
| 1-3/month | 1,899 (20%) |
| <1/month | 2,887 (30%) |
| Never | 2,749 (29%) |
| (Missing) | 27 (0.3%) |
| **Year of birth** |  |
| 1998-2005; age 18-24 | 829 (8.7%) |
| 1993-1998; age 25-29 | 774 (8.1%) |
| 1983-1993; age 30-39 | 1,438 (15%) |
| 1973-1983; age 40-49 | 1,494 (16%) |
| 1963-1973; age 50-59 | 1,729 (18%) |
| 1953-1963; age 60-69 | 1,915 (20%) |
| 1943-1953; age 70-79 | 1,137 (12%) |
| 1943 or earlier; age 80+ | 190 (2.0%) |
| (Missing) | 0 (0%) |
| **Gender** |  |
| Male | 4,641 (49%) |
| Female | 4,843 (51%) |
| Other | 11 (0.1%) |
| (Missing) | 11 (0.1%) |
| **Religious affiliation** |  |
| Christianity | 5,751 (61%) |
| Islam | 350 (3.7%) |
| Hinduism | 15 (0.2%) |
| Buddhism | 25 (0.3%) |
| Judaism | 18 (0.2%) |
| Sikhism | 5 (<0.1%) |
| Baha'i | 2 (<0.1%) |
| Jainism | 1 (<0.1%) |
| Shinto | 0 (0%) |
| Taoism | 0 (0%) |
| Confucianism | 4 (<0.1%) |
| Primal, Animist, or Folk religion | 19 (0.2%) |
| Spiritism | 0 (0%) |
| Umbanda, Candomble, and other African-derived religions | 0 (0%) |
| Chinese folk/traditional religion | 0 (0%) |
| Some other religion | 67 (0.7%) |
| No religion/Atheist/Agnostic | 3,163 (33%) |
| (Missing) | 85 (0.9%) |
| ^1^n (%) | |

***Table S5b. Childhood predictors regression analysis results for Germany***

|  | | Charitable giving | | | | Helping | | | |
| --- | --- | --- | --- | --- | --- | --- | --- | --- | --- |
| Variable | Category | Risk-Ratio | RR 95% CI | log(RR) SE | Global p-value | Risk-Ratio | RR 95% CI | log(RR) SE | Global p-value |
| Relationship with mother | (Ref: Very bad/somewhat bad) |  |  |  | 0.039 |  |  |  | 0.156 |
|  | Very good/somewhat good | 1.16 | (1.00,1.33) | 0.07 |  | 1.07 | (0.97,1.18) | 0.05 |  |
| Relationship with father | (Ref: Very bad/somewhat bad) |  |  |  | 0.625 |  |  |  | 0.915 |
|  | Very good/somewhat good | 1.02 | (0.92,1.14) | 0.05 |  | 1.00 | (0.93,1.08) | 0.04 |  |
| Parent marital status | (Ref: Parents married) |  |  |  | 0.046 |  |  |  | 0.044 |
|  | Divorced | 1.12 | (1.01,1.25) | 0.06 |  | 1.08 | (1.00,1.16) | 0.04 |  |
|  | Parents were never married | 1.13 | (0.99,1.29) | 0.07 |  | 1.02 | (0.93,1.13) | 0.05 |  |
|  | One or both parents had died | 1.11 | (0.93,1.32) | 0.09 |  | 0.86 | (0.72,1.02) | 0.09 |  |
| Subjective financial status of family growing up | (Ref: Got by) |  |  |  | 0.221 |  |  |  | 0.562 |
|  | Lived comfortably | 1.06 | (0.98,1.15) | 0.04 |  | 1.00 | (0.94,1.06) | 0.03 |  |
|  | Found it difficult | 0.95 | (0.86,1.05) | 0.05 |  | 0.98 | (0.91,1.06) | 0.04 |  |
|  | Found it very difficult | 0.91 | (0.73,1.12) | 0.11 |  | 1.07 | (0.95,1.22) | 0.06 |  |
| Abuse | (Ref: No) |  |  |  | <.001 |  |  |  | <.001 |
|  | Yes | 1.18 | (1.08,1.29) | 0.05 |  | 1.21 | (1.14,1.29) | 0.03 |  |
| Outsider growing up | (Ref: No) |  |  |  | 0.635 |  |  |  | 0.007 |
|  | Yes | 1.02 | (0.93,1.12) | 0.05 |  | 1.09 | (1.02,1.16) | 0.03 |  |
| Self-rated health growing up | (Ref: Good) |  |  |  | 0.180 |  |  |  | 0.079 |
|  | Excellent | 0.97 | (0.88,1.07) | 0.05 |  | 0.95 | (0.88,1.02) | 0.04 |  |
|  | Very good | 0.93 | (0.85,1.01) | 0.04 |  | 1.01 | (0.94,1.07) | 0.03 |  |
|  | Fair | 0.91 | (0.78,1.06) | 0.08 |  | 1.10 | (1.00,1.21) | 0.05 |  |
|  | Poor | 1.12 | (0.87,1.45) | 0.13 |  | 1.02 | (0.87,1.19) | 0.08 |  |
| Immigration status | (Ref: Born in this country) |  |  |  | 0.398 |  |  |  | 0.066 |
|  | Born in another country | 1.06 | (0.93,1.20) | 0.07 |  | 1.09 | (0.99,1.20) | 0.05 |  |
| Age 12 religious service attendance | (Ref: Never) |  |  |  | 0.143 |  |  |  | 0.412 |
|  | At least 1/week | 1.04 | (0.95,1.14) | 0.05 |  | 0.98 | (0.91,1.06) | 0.04 |  |
|  | 1-3/month | 1.02 | (0.92,1.12) | 0.05 |  | 1.02 | (0.95,1.10) | 0.04 |  |
|  | < 1/month | 0.95 | (0.86,1.04) | 0.05 |  | 0.97 | (0.91,1.04) | 0.03 |  |
| Year of birth | (Ref: 1998-2005; current age: 18-24) |  |  |  | <.001 |  |  |  | <.001 |
|  | 1993-1998; age 25-29 | 1.52 | (1.26,1.83) | 0.10 |  | 1.00 | (0.91,1.10) | 0.05 |  |
|  | 1983-1993; age 30-39 | 1.71 | (1.44,2.02) | 0.09 |  | 0.95 | (0.87,1.03) | 0.04 |  |
|  | 1973-1983; age 40-49 | 1.59 | (1.34,1.89) | 0.09 |  | 0.88 | (0.81,0.97) | 0.05 |  |
|  | 1963-1973; age 50-59 | 1.39 | (1.16,1.65) | 0.09 |  | 0.72 | (0.66,0.80) | 0.05 |  |
|  | 1953-1963; age 60-69 | 1.53 | (1.29,1.83) | 0.09 |  | 0.70 | (0.64,0.78) | 0.05 |  |
|  | 1943-1953; age 70-79 | 1.55 | (1.29,1.87) | 0.09 |  | 0.54 | (0.48,0.61) | 0.06 |  |
|  | 1943 or earlier; age 80+ | 1.95 | (1.56,2.44) | 0.12 |  | 0.51 | (0.40,0.65) | 0.12 |  |
| Gender | (Ref: Male) |  |  |  | <.001 |  |  |  | <.001 |
|  | Female | 0.74 | (0.70,0.80) | 0.03 |  | 0.79 | (0.75,0.83) | 0.03 |  |
|  | Other | 0.61 | (0.27,1.35) | 0.41 |  | 1.05 | (0.80,1.36) | 0.13 |  |
| Religious affiliation | (Ref: No religion/Atheist/Agnostic) |  |  |  | <.001 |  |  |  | <.001 |
|  | Islam | 4.88 | (4.16,5.71) | 0.08 |  | 1.46 | (1.25,1.70) | 0.08 |  |
|  | Christianity | 3.11 | (2.74,3.52) | 0.06 |  | 1.94 | (1.80,2.09) | 0.04 |  |
|  | Collapsed affiliations with prevalence<3% | 2.19 | (1.54,3.11) | 0.18 |  | 1.88 | (1.56,2.27) | 0.10 |  |
| Race/ethnicity | (Ref: Plurality group) |  |  |  |  |  |  |  |  |

***Table S5c. Sensitivity to unmeasured confounding of childhood predictors in Germany***

|  | | Charitable giving | | Helping | |
| --- | --- | --- | --- | --- | --- |
| Variable | Category | E-value for Estimate | E-value for 95% CI | E-value for Estimate | E-value for 95% CI |
| Relationship with mother | (Ref: Very bad/somewhat bad) |  |  |  |  |
|  | Very good/somewhat good | 1.58 | 1.07 | 1.34 | 1.00 |
| Relationship with father | (Ref: Very bad/somewhat bad) |  |  |  |  |
|  | Very good/somewhat good | 1.18 | 1.00 | 1.07 | 1.00 |
| Parent marital status | (Ref: Parents married) |  |  |  |  |
|  | Divorced | 1.50 | 1.10 | 1.36 | 1.04 |
|  | Parents were never married | 1.52 | 1.00 | 1.17 | 1.00 |
|  | One or both parents had died | 1.45 | 1.00 | 1.61 | 1.00 |
| Subjective financial status of family growing up | (Ref: Got by) |  |  |  |  |
|  | Lived comfortably | 1.31 | 1.00 | 1.02 | 1.00 |
|  | Found it difficult | 1.28 | 1.00 | 1.18 | 1.00 |
|  | Found it very difficult | 1.44 | 1.00 | 1.35 | 1.00 |
| Abuse | (Ref: No) |  |  |  |  |
|  | Yes | 1.64 | 1.36 | 1.72 | 1.54 |
| Outsider growing up | (Ref: No) |  |  |  |  |
|  | Yes | 1.17 | 1.00 | 1.40 | 1.18 |
| Self-rated health growing up | (Ref: Good) |  |  |  |  |
|  | Excellent | 1.20 | 1.00 | 1.29 | 1.00 |
|  | Very good | 1.38 | 1.00 | 1.09 | 1.00 |
|  | Fair | 1.44 | 1.00 | 1.43 | 1.03 |
|  | Poor | 1.50 | 1.00 | 1.15 | 1.00 |
| Immigration status | (Ref: Born in this country) |  |  |  |  |
|  | Born in another country | 1.30 | 1.00 | 1.40 | 1.00 |
| Age 12 religious service attendance | (Ref: Never) |  |  |  |  |
|  | At least 1/week | 1.25 | 1.00 | 1.15 | 1.00 |
|  | 1-3/month | 1.15 | 1.00 | 1.17 | 1.00 |
|  | < 1/month | 1.30 | 1.00 | 1.20 | 1.00 |
| Year of birth | (Ref: 1998-2005; current age: 18-24) |  |  |  |  |
|  | 1993-1998; age 25-29 | 2.40 | 1.83 | 1.04 | 1.00 |
|  | 1983-1993; age 30-39 | 2.81 | 2.24 | 1.29 | 1.00 |
|  | 1973-1983; age 40-49 | 2.56 | 2.01 | 1.52 | 1.22 |
|  | 1963-1973; age 50-59 | 2.12 | 1.60 | 2.11 | 1.82 |
|  | 1953-1963; age 60-69 | 2.44 | 1.90 | 2.19 | 1.90 |
|  | 1943-1953; age 70-79 | 2.48 | 1.91 | 3.10 | 2.65 |
|  | 1943 or earlier; age 80+ | 3.31 | 2.49 | 3.35 | 2.45 |
| Gender | (Ref: Male) |  |  |  |  |
|  | Female | 2.02 | 1.82 | 1.86 | 1.71 |
|  | Other | 2.68 | 1.00 | 1.27 | 1.00 |
| Religious affiliation | (Ref: No religion/Atheist/Agnostic) |  |  |  |  |
|  | Islam | 9.23 | 7.79 | 2.28 | 1.81 |
|  | Christianity | 5.66 | 4.92 | 3.29 | 3.00 |
|  | Collapsed affiliations with prevalence<3% | 3.80 | 2.46 | 3.17 | 2.50 |
| Race/ethnicity | (Ref: Plurality group) |  |  |  |  |

***Table S6a. Nationally representative descriptive statistics for Hong Kong***

| **Characteristic** | **N = 3,012**^1^ |
| --- | --- |
| **Relationship with mother** |  |
| Very good | 1,077 (36%) |
| Somewhat good | 1,164 (39%) |
| Somewhat bad | 293 (9.7%) |
| Very bad | 49 (1.6%) |
| Does not apply | 426 (14%) |
| (Missing) | 3 (<0.1%) |
| **Relationship with father** |  |
| Very good | 868 (29%) |
| Somewhat good | 1,089 (36%) |
| Somewhat bad | 393 (13%) |
| Very bad | 102 (3.4%) |
| Does not apply | 557 (19%) |
| (Missing) | 3 (0.1%) |
| **Parent marital status** |  |
| Parents married | 2,752 (91%) |
| Divorced | 114 (3.8%) |
| Parents were never married | 40 (1.3%) |
| One or both parents had died | 50 (1.7%) |
| (Missing) | 56 (1.8%) |
| **Subjective financial status of family growing up** |  |
| Lived comfortably | 906 (30%) |
| Got by | 1,527 (51%) |
| Found it difficult | 473 (16%) |
| Found it very difficult | 84 (2.8%) |
| (Missing) | 22 (0.7%) |
| **Abuse** |  |
| Yes | 318 (11%) |
| No | 2,688 (89%) |
| (Missing) | 5 (0.2%) |
| **Outsider growing up** |  |
| Yes | 664 (22%) |
| No | 2,224 (74%) |
| (Missing) | 124 (4.1%) |
| **Self-rated health growing up** |  |
| Excellent | 545 (18%) |
| Very good | 1,073 (36%) |
| Good | 863 (29%) |
| Fair | 426 (14%) |
| Poor | 91 (3.0%) |
| (Missing) | 13 (0.4%) |
| **Immigration status** |  |
| Born in this country | 2,637 (88%) |
| Born in another country | 321 (11%) |
| (Missing) | 53 (1.8%) |
| **Age 12 religious service attendance** |  |
| At least 1/week | 432 (14%) |
| 1-3/month | 528 (18%) |
| <1/month | 753 (25%) |
| Never | 1,295 (43%) |
| (Missing) | 4 (0.1%) |
| **Year of birth** |  |
| 1998-2005; age 18-24 | 217 (7.2%) |
| 1993-1998; age 25-29 | 198 (6.6%) |
| 1983-1993; age 30-39 | 507 (17%) |
| 1973-1983; age 40-49 | 580 (19%) |
| 1963-1973; age 50-59 | 711 (24%) |
| 1953-1963; age 60-69 | 620 (21%) |
| 1943-1953; age 70-79 | 164 (5.5%) |
| 1943 or earlier; age 80+ | 15 (0.5%) |
| (Missing) | 0 (0%) |
| **Gender** |  |
| Male | 1,390 (46%) |
| Female | 1,620 (54%) |
| Other | 2 (<0.1%) |
| (Missing) | 0 (0%) |
| **Religious affiliation** |  |
| Christianity | 715 (24%) |
| Islam | 86 (2.9%) |
| Hinduism | 27 (0.9%) |
| Buddhism | 323 (11%) |
| Judaism | 16 (0.5%) |
| Sikhism | 4 (0.1%) |
| Baha'i | 0 (0%) |
| Jainism | 1 (<0.1%) |
| Shinto | 18 (0.6%) |
| Taoism | 81 (2.7%) |
| Confucianism | 10 (0.3%) |
| Primal, Animist, or Folk religion | 15 (0.5%) |
| Spiritism | 0 (0%) |
| Umbanda, Candomble, and other African-derived religions | 0 (0%) |
| Chinese folk/traditional religion | 108 (3.6%) |
| Some other religion | 5 (0.2%) |
| No religion/Atheist/Agnostic | 1,601 (53%) |
| (Missing) | 1 (<0.1%) |
| **Race/Ethnicity** |  |
| Chinese (Cantonese) | 1,930 (64%) |
| Chinese (Chaoshan) | 201 (6.7%) |
| Chinese (Fujianese) | 117 (3.9%) |
| Chinese (Hakka) | 121 (4.0%) |
| Chinese (Other ethnicity) | 264 (8.8%) |
| Chinese (Shanghainese) | 89 (2.9%) |
| East Asian (Korean, Japanese) | 10 (0.3%) |
| Other | 4 (0.1%) |
| South Asian (Indian, Nepalese, Pakistani) | 17 (0.6%) |
| Southeast Asian (Filipino, Indonesian, Thailand) | 46 (1.5%) |
| Taiwanese | 14 (0.4%) |
| White | 15 (0.5%) |
| (Missing) | 184 (6.1%) |
| ^1^n (%) | |

***Table S6b. Childhood predictors regression analysis results for Hong Kong***

|  | | Charitable giving | | | | Helping | | | |
| --- | --- | --- | --- | --- | --- | --- | --- | --- | --- |
| Variable | Category | Risk-Ratio | RR 95% CI | log(RR) SE | Global p-value | Risk-Ratio | RR 95% CI | log(RR) SE | Global p-value |
| Relationship with mother | (Ref: Very bad/somewhat bad) |  |  |  | 0.859 |  |  |  | 0.347 |
|  | Very good/somewhat good | 0.99 | (0.84,1.17) | 0.09 |  | 0.94 | (0.84,1.07) | 0.06 |  |
| Relationship with father | (Ref: Very bad/somewhat bad) |  |  |  | 0.250 |  |  |  | 0.380 |
|  | Very good/somewhat good | 1.10 | (0.93,1.30) | 0.09 |  | 0.95 | (0.85,1.06) | 0.06 |  |
| Parent marital status | (Ref: Parents married) |  |  |  | 0.987 |  |  |  | 0.089 |
|  | Divorced | 0.99 | (0.72,1.35) | 0.16 |  | 0.84 | (0.66,1.06) | 0.12 |  |
|  | Parents were never married | 0.97 | (0.61,1.55) | 0.24 |  | 0.87 | (0.59,1.27) | 0.19 |  |
|  | One or both parents had died | 0.94 | (0.56,1.57) | 0.26 |  | 1.28 | (0.98,1.68) | 0.14 |  |
| Subjective financial status of family growing up | (Ref: Got by) |  |  |  | 0.014 |  |  |  | 0.005 |
|  | Lived comfortably | 1.06 | (0.96,1.18) | 0.05 |  | 1.13 | (1.05,1.22) | 0.04 |  |
|  | Found it difficult | 0.77 | (0.64,0.94) | 0.10 |  | 1.07 | (0.95,1.22) | 0.06 |  |
|  | Found it very difficult | 0.72 | (0.41,1.26) | 0.28 |  | 0.83 | (0.55,1.24) | 0.21 |  |
| Abuse | (Ref: No) |  |  |  | 0.100 |  |  |  | 0.499 |
|  | Yes | 1.11 | (0.98,1.26) | 0.07 |  | 1.04 | (0.93,1.16) | 0.06 |  |
| Outsider growing up | (Ref: No) |  |  |  | 0.035 |  |  |  | 0.096 |
|  | Yes | 1.12 | (1.01,1.24) | 0.05 |  | 1.07 | (0.98,1.16) | 0.04 |  |
| Self-rated health growing up | (Ref: Good) |  |  |  | 0.002 |  |  |  | <.001 |
|  | Excellent | 1.25 | (1.06,1.47) | 0.08 |  | 1.16 | (1.04,1.30) | 0.06 |  |
|  | Very good | 1.32 | (1.15,1.51) | 0.07 |  | 1.10 | (0.99,1.21) | 0.05 |  |
|  | Fair | 1.16 | (0.96,1.41) | 0.10 |  | 0.82 | (0.71,0.95) | 0.08 |  |
|  | Poor | 1.34 | (0.83,2.16) | 0.24 |  | 0.90 | (0.64,1.27) | 0.18 |  |
| Immigration status | (Ref: Born in this country) |  |  |  | 0.124 |  |  |  | 0.387 |
|  | Born in another country | 0.86 | (0.71,1.04) | 0.10 |  | 0.94 | (0.81,1.08) | 0.07 |  |
| Age 12 religious service attendance | (Ref: Never) |  |  |  | <.001 |  |  |  | <.001 |
|  | At least 1/week | 1.36 | (1.15,1.60) | 0.08 |  | 1.37 | (1.20,1.57) | 0.07 |  |
|  | 1-3/month | 1.28 | (1.11,1.49) | 0.08 |  | 1.35 | (1.20,1.52) | 0.06 |  |
|  | < 1/month | 0.99 | (0.85,1.15) | 0.08 |  | 1.27 | (1.13,1.41) | 0.06 |  |
| Year of birth | (Ref: 1998-2005; current age: 18-24) |  |  |  | <.001 |  |  |  | <.001 |
|  | 1993-1998; age 25-29 | 1.08 | (0.89,1.31) | 0.10 |  | 1.01 | (0.86,1.19) | 0.08 |  |
|  | 1983-1993; age 30-39 | 1.04 | (0.89,1.23) | 0.08 |  | 1.07 | (0.96,1.20) | 0.06 |  |
|  | 1973-1983; age 40-49 | 1.17 | (1.01,1.36) | 0.08 |  | 0.95 | (0.84,1.06) | 0.06 |  |
|  | 1963-1973; age 50-59 | 1.22 | (1.05,1.41) | 0.08 |  | 1.00 | (0.90,1.11) | 0.05 |  |
|  | 1953-1963; age 60-69 | 1.05 | (0.86,1.29) | 0.10 |  | 0.96 | (0.84,1.11) | 0.07 |  |
|  | 1943-1953; age 70-79 | 0.53 | (0.30,0.96) | 0.30 |  | 0.78 | (0.54,1.11) | 0.18 |  |
|  | 1943 or earlier; age 80+ | 1.84 | (1.39,2.43) | 0.14 |  | 1.41 | (1.18,1.68) | 0.09 |  |
| Gender | (Ref: Male) |  |  |  | <.001 |  |  |  | <.001 |
|  | Female | 0.95 | (0.87,1.04) | 0.05 |  | 1.07 | (1.00,1.15) | 0.04 |  |
|  | Other | 0.00 | (0.00,0.00) | 0.72 |  | 0.00 | (0.00,0.00) | 0.72 |  |
| Religious affiliation | (Ref: No religion/Atheist/Agnostic) |  |  |  | <.001 |  |  |  | 0.168 |
|  | Buddhism | 1.38 | (1.19,1.61) | 0.08 |  | 1.14 | (1.01,1.27) | 0.06 |  |
|  | Chinese folk/traditional religion | 1.46 | (1.23,1.74) | 0.09 |  | 1.02 | (0.85,1.21) | 0.09 |  |
|  | Christianity | 1.22 | (1.05,1.41) | 0.08 |  | 1.11 | (1.00,1.24) | 0.05 |  |
|  | Collapsed affiliations with prevalence<3% | 1.42 | (1.19,1.68) | 0.09 |  | 1.12 | (0.98,1.27) | 0.07 |  |
| Race/ethnicity | (Ref: Plurality group) |  |  |  | 0.301 |  |  |  | 0.302 |
|  | Non-plurality groups | 1.05 | (0.95,1.16) | 0.05 |  | 1.04 | (0.96,1.12) | 0.04 |  |

***Table S6c. Sensitivity to unmeasured confounding of childhood predictors in Hong Kong***

|  | | Charitable giving | | Helping | |
| --- | --- | --- | --- | --- | --- |
| Variable | Category | E-value for Estimate | E-value for 95% CI | E-value for Estimate | E-value for 95% CI |
| Relationship with mother | (Ref: Very bad/somewhat bad) |  |  |  |  |
|  | Very good/somewhat good | 1.11 | 1.00 | 1.31 | 1.00 |
| Relationship with father | (Ref: Very bad/somewhat bad) |  |  |  |  |
|  | Very good/somewhat good | 1.43 | 1.00 | 1.28 | 1.00 |
| Parent marital status | (Ref: Parents married) |  |  |  |  |
|  | Divorced | 1.14 | 1.00 | 1.67 | 1.00 |
|  | Parents were never married | 1.20 | 1.00 | 1.57 | 1.00 |
|  | One or both parents had died | 1.32 | 1.00 | 1.89 | 1.00 |
| Subjective financial status of family growing up | (Ref: Got by) |  |  |  |  |
|  | Lived comfortably | 1.33 | 1.00 | 1.53 | 1.29 |
|  | Found it difficult | 1.91 | 1.33 | 1.36 | 1.00 |
|  | Found it very difficult | 2.12 | 1.00 | 1.71 | 1.00 |
| Abuse | (Ref: No) |  |  |  |  |
|  | Yes | 1.46 | 1.00 | 1.24 | 1.00 |
| Outsider growing up | (Ref: No) |  |  |  |  |
|  | Yes | 1.48 | 1.09 | 1.34 | 1.00 |
| Self-rated health growing up | (Ref: Good) |  |  |  |  |
|  | Excellent | 1.81 | 1.33 | 1.60 | 1.25 |
|  | Very good | 1.96 | 1.57 | 1.42 | 1.00 |
|  | Fair | 1.59 | 1.00 | 1.73 | 1.27 |
|  | Poor | 2.01 | 1.00 | 1.47 | 1.00 |
| Immigration status | (Ref: Born in this country) |  |  |  |  |
|  | Born in another country | 1.60 | 1.00 | 1.33 | 1.00 |
| Age 12 religious service attendance | (Ref: Never) |  |  |  |  |
|  | At least 1/week | 2.05 | 1.56 | 2.09 | 1.70 |
|  | 1-3/month | 1.88 | 1.45 | 2.04 | 1.69 |
|  | < 1/month | 1.11 | 1.00 | 1.85 | 1.52 |
| Year of birth | (Ref: 1998-2005; current age: 18-24) |  |  |  |  |
|  | 1993-1998; age 25-29 | 1.37 | 1.00 | 1.12 | 1.00 |
|  | 1983-1993; age 30-39 | 1.26 | 1.00 | 1.35 | 1.00 |
|  | 1973-1983; age 40-49 | 1.62 | 1.08 | 1.30 | 1.00 |
|  | 1963-1973; age 50-59 | 1.74 | 1.28 | 1.02 | 1.00 |
|  | 1953-1963; age 60-69 | 1.29 | 1.00 | 1.24 | 1.00 |
|  | 1943-1953; age 70-79 | 3.14 | 1.25 | 1.89 | 1.00 |
|  | 1943 or earlier; age 80+ | 3.08 | 2.12 | 2.17 | 1.64 |
| Gender | (Ref: Male) |  |  |  |  |
|  | Female | 1.29 | 1.00 | 1.36 | 1.05 |
|  | Other | 539091.54 | 131544.48 | 747873.88 | 181698.75 |
| Religious affiliation | (Ref: No religion/Atheist/Agnostic) |  |  |  |  |
|  | Buddhism | 2.11 | 1.66 | 1.53 | 1.13 |
|  | Chinese folk/traditional religion | 2.28 | 1.76 | 1.14 | 1.00 |
|  | Christianity | 1.74 | 1.28 | 1.47 | 1.03 |
|  | Collapsed affiliations with prevalence<3% | 2.19 | 1.67 | 1.48 | 1.00 |
| Race/ethnicity | (Ref: Plurality group) |  |  |  |  |
|  | Non-plurality groups | 1.28 | 1.00 | 1.24 | 1.00 |

***Table S7a. Nationally representative descriptive statistics for India***

| **Characteristic** | **N = 12,765**^1^ |
| --- | --- |
| **Relationship with mother** |  |
| Very good | 11,465 (90%) |
| Somewhat good | 788 (6.2%) |
| Somewhat bad | 88 (0.7%) |
| Very bad | 73 (0.6%) |
| Does not apply | 269 (2.1%) |
| (Missing) | 82 (0.6%) |
| **Relationship with father** |  |
| Very good | 10,923 (86%) |
| Somewhat good | 995 (7.8%) |
| Somewhat bad | 126 (1.0%) |
| Very bad | 100 (0.8%) |
| Does not apply | 481 (3.8%) |
| (Missing) | 141 (1.1%) |
| **Parent marital status** |  |
| Parents married | 5,578 (44%) |
| Divorced | 236 (1.8%) |
| Parents were never married | 1,055 (8.3%) |
| One or both parents had died | 940 (7.4%) |
| (Missing) | 4,956 (39%) |
| **Subjective financial status of family growing up** |  |
| Lived comfortably | 4,946 (39%) |
| Got by | 3,010 (24%) |
| Found it difficult | 2,703 (21%) |
| Found it very difficult | 2,035 (16%) |
| (Missing) | 70 (0.5%) |
| **Abuse** |  |
| Yes | 1,468 (11%) |
| No | 10,526 (82%) |
| (Missing) | 771 (6.0%) |
| **Outsider growing up** |  |
| Yes | 1,926 (15%) |
| No | 10,780 (84%) |
| (Missing) | 59 (0.5%) |
| **Self-rated health growing up** |  |
| Excellent | 2,182 (17%) |
| Very good | 3,882 (30%) |
| Good | 4,028 (32%) |
| Fair | 2,202 (17%) |
| Poor | 424 (3.3%) |
| (Missing) | 47 (0.4%) |
| **Immigration status** |  |
| Born in this country | 12,629 (99%) |
| Born in another country | 110 (0.9%) |
| (Missing) | 26 (0.2%) |
| **Age 12 religious service attendance** |  |
| At least 1/week | 5,288 (41%) |
| 1-3/month | 2,959 (23%) |
| <1/month | 2,719 (21%) |
| Never | 1,478 (12%) |
| (Missing) | 321 (2.5%) |
| **Year of birth** |  |
| 1998-2005; age 18-24 | 2,543 (20%) |
| 1993-1998; age 25-29 | 1,640 (13%) |
| 1983-1993; age 30-39 | 3,109 (24%) |
| 1973-1983; age 40-49 | 2,275 (18%) |
| 1963-1973; age 50-59 | 1,574 (12%) |
| 1953-1963; age 60-69 | 1,188 (9.3%) |
| 1943-1953; age 70-79 | 370 (2.9%) |
| 1943 or earlier; age 80+ | 67 (0.5%) |
| (Missing) | 0 (0%) |
| **Gender** |  |
| Male | 6,473 (51%) |
| Female | 6,292 (49%) |
| Other | 0 (0%) |
| (Missing) | 0 (0%) |
| **Religious affiliation** |  |
| Christianity | 254 (2.0%) |
| Islam | 1,550 (12%) |
| Hinduism | 10,417 (82%) |
| Buddhism | 180 (1.4%) |
| Judaism | 0 (0%) |
| Sikhism | 126 (1.0%) |
| Baha'i | 0 (0%) |
| Jainism | 9 (<0.1%) |
| Shinto | 4 (<0.1%) |
| Taoism | 0 (0%) |
| Confucianism | 0 (0%) |
| Primal, Animist, or Folk religion | 27 (0.2%) |
| Spiritism | 0 (0%) |
| Umbanda, Candomble, and other African-derived religions | 0 (0%) |
| Chinese folk/traditional religion | 0 (0%) |
| Some other religion | 59 (0.5%) |
| No religion/Atheist/Agnostic | 7 (<0.1%) |
| (Missing) | 131 (1.0%) |
| **Race/Ethnicity** |  |
| General | 3,538 (28%) |
| Other backward caste | 4,177 (33%) |
| Schedule caste | 3,599 (28%) |
| Schedule tribe | 1,185 (9.3%) |
| (Missing) | 267 (2.1%) |
| ^1^n (%) | |

***Table S7b. Childhood predictors regression analysis results for India***

|  | | Charitable giving | | | | Helping | | | |
| --- | --- | --- | --- | --- | --- | --- | --- | --- | --- |
| Variable | Category | Risk-Ratio | RR 95% CI | log(RR) SE | Global p-value | Risk-Ratio | RR 95% CI | log(RR) SE | Global p-value |
| Relationship with mother | (Ref: Very bad/somewhat bad) |  |  |  | 0.228 |  |  |  | 0.432 |
|  | Very good/somewhat good | 1.14 | (0.92,1.41) | 0.11 |  | 1.07 | (0.90,1.28) | 0.09 |  |
| Relationship with father | (Ref: Very bad/somewhat bad) |  |  |  | 0.620 |  |  |  | 0.554 |
|  | Very good/somewhat good | 1.04 | (0.86,1.27) | 0.10 |  | 0.96 | (0.84,1.10) | 0.07 |  |
| Parent marital status | (Ref: Parents married) |  |  |  | 0.028 |  |  |  | 0.755 |
|  | Divorced | 1.20 | (1.02,1.40) | 0.08 |  | 1.00 | (0.84,1.17) | 0.08 |  |
|  | Parents were never married | 1.04 | (0.95,1.15) | 0.05 |  | 1.00 | (0.93,1.07) | 0.04 |  |
|  | One or both parents had died | 0.95 | (0.85,1.05) | 0.05 |  | 0.99 | (0.92,1.07) | 0.04 |  |
| Subjective financial status of family growing up | (Ref: Got by) |  |  |  | 0.037 |  |  |  | 0.414 |
|  | Lived comfortably | 1.01 | (0.93,1.09) | 0.04 |  | 1.05 | (0.99,1.11) | 0.03 |  |
|  | Found it difficult | 0.93 | (0.85,1.02) | 0.05 |  | 1.01 | (0.94,1.08) | 0.04 |  |
|  | Found it very difficult | 0.87 | (0.77,0.98) | 0.06 |  | 1.04 | (0.96,1.12) | 0.04 |  |
| Abuse | (Ref: No) |  |  |  | <.001 |  |  |  | 0.003 |
|  | Yes | 1.15 | (1.07,1.24) | 0.04 |  | 1.08 | (1.03,1.13) | 0.03 |  |
| Outsider growing up | (Ref: No) |  |  |  | <.001 |  |  |  | <.001 |
|  | Yes | 1.40 | (1.29,1.53) | 0.04 |  | 1.18 | (1.11,1.25) | 0.03 |  |
| Self-rated health growing up | (Ref: Good) |  |  |  | <.001 |  |  |  | 0.007 |
|  | Excellent | 1.15 | (1.03,1.27) | 0.05 |  | 1.12 | (1.05,1.20) | 0.04 |  |
|  | Very good | 1.25 | (1.15,1.36) | 0.04 |  | 1.03 | (0.98,1.10) | 0.03 |  |
|  | Fair | 1.13 | (1.02,1.25) | 0.05 |  | 0.97 | (0.91,1.04) | 0.04 |  |
|  | Poor | 1.45 | (1.26,1.68) | 0.07 |  | 1.08 | (0.98,1.19) | 0.05 |  |
| Immigration status | (Ref: Born in this country) |  |  |  | 0.475 |  |  |  | 0.054 |
|  | Born in another country | 1.14 | (0.80,1.61) | 0.18 |  | 1.20 | (0.99,1.46) | 0.10 |  |
| Age 12 religious service attendance | (Ref: Never) |  |  |  | <.001 |  |  |  | <.001 |
|  | At least 1/week | 1.43 | (1.27,1.62) | 0.06 |  | 1.27 | (1.17,1.37) | 0.04 |  |
|  | 1-3/month | 1.38 | (1.21,1.58) | 0.07 |  | 1.19 | (1.09,1.31) | 0.05 |  |
|  | < 1/month | 1.22 | (1.08,1.38) | 0.06 |  | 1.15 | (1.05,1.25) | 0.04 |  |
| Year of birth | (Ref: 1998-2005; current age: 18-24) |  |  |  | 0.079 |  |  |  | 0.042 |
|  | 1993-1998; age 25-29 | 1.08 | (0.98,1.20) | 0.05 |  | 1.01 | (0.94,1.08) | 0.03 |  |
|  | 1983-1993; age 30-39 | 1.05 | (0.96,1.14) | 0.04 |  | 1.02 | (0.96,1.08) | 0.03 |  |
|  | 1973-1983; age 40-49 | 1.12 | (1.02,1.23) | 0.05 |  | 1.01 | (0.95,1.08) | 0.03 |  |
|  | 1963-1973; age 50-59 | 1.08 | (0.97,1.20) | 0.05 |  | 0.97 | (0.89,1.05) | 0.04 |  |
|  | 1953-1963; age 60-69 | 1.15 | (1.01,1.30) | 0.06 |  | 0.90 | (0.82,0.99) | 0.05 |  |
|  | 1943-1953; age 70-79 | 1.08 | (0.90,1.30) | 0.09 |  | 0.87 | (0.75,1.00) | 0.07 |  |
|  | 1943 or earlier; age 80+ | 1.47 | (1.08,2.01) | 0.16 |  | 0.83 | (0.59,1.16) | 0.17 |  |
| Gender | (Ref: Male) |  |  |  | 0.675 |  |  |  | <.001 |
|  | Female | 1.01 | (0.96,1.07) | 0.03 |  | 0.88 | (0.85,0.92) | 0.02 |  |
| Religious affiliation | (Ref: Hinduism) |  |  |  | <.001 |  |  |  | 0.006 |
|  | Islam | 1.31 | (1.18,1.45) | 0.05 |  | 1.13 | (1.04,1.23) | 0.04 |  |
|  | Collapsed affiliations with prevalence<3% | 1.04 | (0.88,1.23) | 0.09 |  | 1.06 | (0.97,1.17) | 0.05 |  |
| Race/ethnicity | (Ref: Plurality group) |  |  |  | 0.303 |  |  |  | 0.007 |
|  | Non-plurality groups | 1.04 | (0.96,1.13) | 0.04 |  | 1.08 | (1.02,1.14) | 0.03 |  |

***Table S7c. Sensitivity to unmeasured confounding of childhood predictors in India***

|  | | Charitable giving | | Helping | |
| --- | --- | --- | --- | --- | --- |
| Variable | Category | E-value for Estimate | E-value for 95% CI | E-value for Estimate | E-value for 95% CI |
| Relationship with mother | (Ref: Very bad/somewhat bad) |  |  |  |  |
|  | Very good/somewhat good | 1.53 | 1.00 | 1.35 | 1.00 |
| Relationship with father | (Ref: Very bad/somewhat bad) |  |  |  |  |
|  | Very good/somewhat good | 1.26 | 1.00 | 1.25 | 1.00 |
| Parent marital status | (Ref: Parents married) |  |  |  |  |
|  | Divorced | 1.68 | 1.18 | 1.07 | 1.00 |
|  | Parents were never married | 1.25 | 1.00 | 1.04 | 1.00 |
|  | One or both parents had died | 1.30 | 1.00 | 1.10 | 1.00 |
| Subjective financial status of family growing up | (Ref: Got by) |  |  |  |  |
|  | Lived comfortably | 1.08 | 1.00 | 1.26 | 1.00 |
|  | Found it difficult | 1.36 | 1.00 | 1.11 | 1.00 |
|  | Found it very difficult | 1.57 | 1.19 | 1.23 | 1.00 |
| Abuse | (Ref: No) |  |  |  |  |
|  | Yes | 1.57 | 1.33 | 1.37 | 1.19 |
| Outsider growing up | (Ref: No) |  |  |  |  |
|  | Yes | 2.16 | 1.90 | 1.63 | 1.46 |
| Self-rated health growing up | (Ref: Good) |  |  |  |  |
|  | Excellent | 1.55 | 1.20 | 1.49 | 1.26 |
|  | Very good | 1.81 | 1.57 | 1.22 | 1.00 |
|  | Fair | 1.52 | 1.18 | 1.21 | 1.00 |
|  | Poor | 2.26 | 1.83 | 1.38 | 1.00 |
| Immigration status | (Ref: Born in this country) |  |  |  |  |
|  | Born in another country | 1.53 | 1.00 | 1.69 | 1.00 |
| Age 12 religious service attendance | (Ref: Never) |  |  |  |  |
|  | At least 1/week | 2.22 | 1.86 | 1.85 | 1.62 |
|  | 1-3/month | 2.11 | 1.73 | 1.68 | 1.40 |
|  | < 1/month | 1.74 | 1.37 | 1.56 | 1.29 |
| Year of birth | (Ref: 1998-2005; current age: 18-24) |  |  |  |  |
|  | 1993-1998; age 25-29 | 1.39 | 1.00 | 1.09 | 1.00 |
|  | 1983-1993; age 30-39 | 1.27 | 1.00 | 1.17 | 1.00 |
|  | 1973-1983; age 40-49 | 1.49 | 1.16 | 1.12 | 1.00 |
|  | 1963-1973; age 50-59 | 1.37 | 1.00 | 1.21 | 1.00 |
|  | 1953-1963; age 60-69 | 1.56 | 1.12 | 1.47 | 1.12 |
|  | 1943-1953; age 70-79 | 1.38 | 1.00 | 1.58 | 1.00 |
|  | 1943 or earlier; age 80+ | 2.30 | 1.37 | 1.71 | 1.00 |
| Gender | (Ref: Male) |  |  |  |  |
|  | Female | 1.12 | 1.00 | 1.52 | 1.40 |
| Religious affiliation | (Ref: Hinduism) |  |  |  |  |
|  | Islam | 1.94 | 1.63 | 1.52 | 1.26 |
|  | Collapsed affiliations with prevalence<3% | 1.24 | 1.00 | 1.32 | 1.00 |
| Race/ethnicity | (Ref: Plurality group) |  |  |  |  |
|  | Non-plurality groups | 1.25 | 1.00 | 1.36 | 1.15 |

***Table S8a. Nationally representative descriptive statistics for Indonesia***

| **Characteristic** | **N = 6,992**^1^ |
| --- | --- |
| **Relationship with mother** |  |
| Very good | 6,238 (89%) |
| Somewhat good | 583 (8.3%) |
| Somewhat bad | 50 (0.7%) |
| Very bad | 26 (0.4%) |
| Does not apply | 68 (1.0%) |
| (Missing) | 27 (0.4%) |
| **Relationship with father** |  |
| Very good | 6,067 (87%) |
| Somewhat good | 628 (9.0%) |
| Somewhat bad | 68 (1.0%) |
| Very bad | 52 (0.7%) |
| Does not apply | 115 (1.6%) |
| (Missing) | 61 (0.9%) |
| **Parent marital status** |  |
| Parents married | 5,557 (79%) |
| Divorced | 448 (6.4%) |
| Parents were never married | 47 (0.7%) |
| One or both parents had died | 735 (11%) |
| (Missing) | 205 (2.9%) |
| **Subjective financial status of family growing up** |  |
| Lived comfortably | 3,408 (49%) |
| Got by | 2,955 (42%) |
| Found it difficult | 439 (6.3%) |
| Found it very difficult | 181 (2.6%) |
| (Missing) | 9 (0.1%) |
| **Abuse** |  |
| Yes | 486 (6.9%) |
| No | 6,427 (92%) |
| (Missing) | 79 (1.1%) |
| **Outsider growing up** |  |
| Yes | 343 (4.9%) |
| No | 6,639 (95%) |
| (Missing) | 10 (0.1%) |
| **Self-rated health growing up** |  |
| Excellent | 1,246 (18%) |
| Very good | 1,968 (28%) |
| Good | 2,490 (36%) |
| Fair | 1,233 (18%) |
| Poor | 55 (0.8%) |
| (Missing) | 1 (<0.1%) |
| **Immigration status** |  |
| Born in this country | 6,958 (100%) |
| Born in another country | 34 (0.5%) |
| (Missing) | 0 (0%) |
| **Age 12 religious service attendance** |  |
| At least 1/week | 5,363 (77%) |
| 1-3/month | 973 (14%) |
| <1/month | 329 (4.7%) |
| Never | 275 (3.9%) |
| (Missing) | 51 (0.7%) |
| **Year of birth** |  |
| 1998-2005; age 18-24 | 1,216 (17%) |
| 1993-1998; age 25-29 | 849 (12%) |
| 1983-1993; age 30-39 | 1,591 (23%) |
| 1973-1983; age 40-49 | 1,576 (23%) |
| 1963-1973; age 50-59 | 1,169 (17%) |
| 1953-1963; age 60-69 | 490 (7.0%) |
| 1943-1953; age 70-79 | 83 (1.2%) |
| 1943 or earlier; age 80+ | 17 (0.2%) |
| (Missing) | 0 (0%) |
| **Gender** |  |
| Male | 3,461 (50%) |
| Female | 3,513 (50%) |
| Other | 7 (<0.1%) |
| (Missing) | 11 (0.2%) |
| **Religious affiliation** |  |
| Christianity | 528 (7.6%) |
| Islam | 6,373 (91%) |
| Hinduism | 75 (1.1%) |
| Buddhism | 5 (<0.1%) |
| Judaism | 0 (0%) |
| Sikhism | 0 (0%) |
| Baha'i | 0 (0%) |
| Jainism | 1 (<0.1%) |
| Shinto | 0 (0%) |
| Taoism | 0 (<0.1%) |
| Confucianism | 1 (<0.1%) |
| Primal, Animist, or Folk religion | 1 (<0.1%) |
| Spiritism | 0 (0%) |
| Umbanda, Candomble, and other African-derived religions | 0 (0%) |
| Chinese folk/traditional religion | 0 (0%) |
| Some other religion | 0 (0%) |
| No religion/Atheist/Agnostic | 2 (<0.1%) |
| (Missing) | 8 (0.1%) |
| **Race/Ethnicity** |  |
| Bali | 69 (1.0%) |
| Banjar/Melayu Banjar | 320 (4.6%) |
| Batak | 165 (2.4%) |
| Betawi | 251 (3.6%) |
| Bugis | 243 (3.5%) |
| Jawa | 2,846 (41%) |
| Madura | 262 (3.7%) |
| Makasar | 91 (1.3%) |
| Minangkabau | 273 (3.9%) |
| Other | 1,262 (18%) |
| Sunda/Parahyangan | 1,172 (17%) |
| (Missing) | 38 (0.5%) |
| ^1^n (%) | |

***Table S8b. Childhood predictors regression analysis results for Indonesia***

|  | | Charitable giving | | | | Helping | | | |
| --- | --- | --- | --- | --- | --- | --- | --- | --- | --- |
| Variable | Category | Risk-Ratio | RR 95% CI | log(RR) SE | Global p-value | Risk-Ratio | RR 95% CI | log(RR) SE | Global p-value |
| Relationship with mother | (Ref: Very bad/somewhat bad) |  |  |  | 0.688 |  |  |  | 0.649 |
|  | Very good/somewhat good | 1.03 | (0.89,1.20) | 0.08 |  | 0.95 | (0.70,1.28) | 0.15 |  |
| Relationship with father | (Ref: Very bad/somewhat bad) |  |  |  | 0.037 |  |  |  | 0.619 |
|  | Very good/somewhat good | 1.12 | (1.00,1.25) | 0.06 |  | 0.96 | (0.79,1.16) | 0.10 |  |
| Parent marital status | (Ref: Parents married) |  |  |  | 0.270 |  |  |  | 0.936 |
|  | Divorced | 1.05 | (0.99,1.12) | 0.03 |  | 0.98 | (0.86,1.12) | 0.06 |  |
|  | Parents were never married | 0.89 | (0.67,1.18) | 0.14 |  | 1.00 | (0.72,1.38) | 0.17 |  |
|  | One or both parents had died | 1.02 | (0.97,1.07) | 0.03 |  | 0.97 | (0.86,1.08) | 0.06 |  |
| Subjective financial status of family growing up | (Ref: Got by) |  |  |  | 0.148 |  |  |  | 0.045 |
|  | Lived comfortably | 1.03 | (1.00,1.06) | 0.01 |  | 1.05 | (0.99,1.12) | 0.03 |  |
|  | Found it difficult | 0.98 | (0.91,1.06) | 0.04 |  | 1.04 | (0.90,1.21) | 0.08 |  |
|  | Found it very difficult | 1.02 | (0.91,1.14) | 0.06 |  | 0.76 | (0.56,1.02) | 0.15 |  |
| Abuse | (Ref: No) |  |  |  | 0.335 |  |  |  | 0.008 |
|  | Yes | 0.97 | (0.90,1.04) | 0.04 |  | 1.15 | (1.04,1.27) | 0.05 |  |
| Outsider growing up | (Ref: No) |  |  |  | 0.766 |  |  |  | 0.021 |
|  | Yes | 1.01 | (0.94,1.09) | 0.04 |  | 1.16 | (1.02,1.32) | 0.07 |  |
| Self-rated health growing up | (Ref: Good) |  |  |  | <.001 |  |  |  | 0.009 |
|  | Excellent | 1.07 | (1.03,1.12) | 0.02 |  | 1.14 | (1.05,1.25) | 0.04 |  |
|  | Very good | 1.04 | (1.00,1.08) | 0.02 |  | 1.08 | (1.00,1.16) | 0.04 |  |
|  | Fair | 0.96 | (0.92,1.01) | 0.03 |  | 1.00 | (0.91,1.10) | 0.05 |  |
|  | Poor | 0.90 | (0.70,1.17) | 0.13 |  | 1.13 | (0.80,1.60) | 0.18 |  |
| Immigration status | (Ref: Born in this country) |  |  |  | 0.766 |  |  |  | 0.768 |
|  | Born in another country | 1.06 | (0.74,1.51) | 0.18 |  | 0.95 | (0.70,1.30) | 0.16 |  |
| Age 12 religious service attendance | (Ref: Never) |  |  |  | <.001 |  |  |  | 0.289 |
|  | At least 1/week | 1.20 | (1.08,1.32) | 0.05 |  | 1.12 | (0.95,1.32) | 0.08 |  |
|  | 1-3/month | 1.17 | (1.05,1.30) | 0.05 |  | 1.11 | (0.92,1.33) | 0.09 |  |
|  | < 1/month | 1.06 | (0.93,1.20) | 0.06 |  | 0.99 | (0.81,1.22) | 0.10 |  |
| Year of birth | (Ref: 1998-2005; current age: 18-24) |  |  |  | <.001 |  |  |  | <.001 |
|  | 1993-1998; age 25-29 | 1.08 | (1.02,1.15) | 0.03 |  | 0.95 | (0.86,1.04) | 0.05 |  |
|  | 1983-1993; age 30-39 | 1.09 | (1.04,1.14) | 0.02 |  | 0.92 | (0.85,1.00) | 0.04 |  |
|  | 1973-1983; age 40-49 | 1.11 | (1.06,1.17) | 0.02 |  | 0.88 | (0.81,0.96) | 0.04 |  |
|  | 1963-1973; age 50-59 | 1.11 | (1.05,1.18) | 0.03 |  | 0.75 | (0.67,0.84) | 0.06 |  |
|  | 1953-1963; age 60-69 | 1.14 | (1.05,1.24) | 0.04 |  | 0.67 | (0.55,0.81) | 0.10 |  |
|  | 1943-1953; age 70-79 | 1.10 | (0.90,1.34) | 0.10 |  | 0.45 | (0.25,0.82) | 0.30 |  |
|  | 1943 or earlier; age 80+ | 1.47 | (1.33,1.62) | 0.05 |  | 1.11 | (0.47,2.64) | 0.44 |  |
| Gender | (Ref: Male) |  |  |  | 0.009 |  |  |  | <.001 |
|  | Female | 1.06 | (1.02,1.09) | 0.02 |  | 0.88 | (0.83,0.93) | 0.03 |  |
|  | Other | 1.09 | (0.84,1.42) | 0.13 |  | 0.67 | (0.22,2.09) | 0.58 |  |
| Religious affiliation | (Ref: Islam) |  |  |  | <.001 |  |  |  | 0.269 |
|  | Christianity | 0.69 | (0.64,0.75) | 0.04 |  | 1.00 | (0.87,1.15) | 0.07 |  |
|  | Collapsed affiliations with prevalence<3% | 0.68 | (0.49,0.94) | 0.17 |  | 0.79 | (0.59,1.05) | 0.15 |  |
| Race/ethnicity | (Ref: Plurality group) |  |  |  | 0.569 |  |  |  | 0.015 |
|  | Non-plurality groups | 1.01 | (0.97,1.05) | 0.02 |  | 0.92 | (0.86,0.99) | 0.03 |  |

***Table S8c. Sensitivity to unmeasured confounding of childhood predictors in Indonesia***

|  | | Charitable giving | | Helping | |
| --- | --- | --- | --- | --- | --- |
| Variable | Category | E-value for Estimate | E-value for 95% CI | E-value for Estimate | E-value for 95% CI |
| Relationship with mother | (Ref: Very bad/somewhat bad) |  |  |  |  |
|  | Very good/somewhat good | 1.21 | 1.00 | 1.30 | 1.00 |
| Relationship with father | (Ref: Very bad/somewhat bad) |  |  |  |  |
|  | Very good/somewhat good | 1.49 | 1.02 | 1.26 | 1.00 |
| Parent marital status | (Ref: Parents married) |  |  |  |  |
|  | Divorced | 1.29 | 1.00 | 1.15 | 1.00 |
|  | Parents were never married | 1.50 | 1.00 | 1.06 | 1.00 |
|  | One or both parents had died | 1.15 | 1.00 | 1.23 | 1.00 |
| Subjective financial status of family growing up | (Ref: Got by) |  |  |  |  |
|  | Lived comfortably | 1.21 | 1.04 | 1.29 | 1.00 |
|  | Found it difficult | 1.17 | 1.00 | 1.25 | 1.00 |
|  | Found it very difficult | 1.15 | 1.00 | 1.97 | 1.00 |
| Abuse | (Ref: No) |  |  |  |  |
|  | Yes | 1.22 | 1.00 | 1.55 | 1.23 |
| Outsider growing up | (Ref: No) |  |  |  |  |
|  | Yes | 1.12 | 1.00 | 1.59 | 1.16 |
| Self-rated health growing up | (Ref: Good) |  |  |  |  |
|  | Excellent | 1.35 | 1.20 | 1.55 | 1.28 |
|  | Very good | 1.24 | 1.00 | 1.37 | 1.01 |
|  | Fair | 1.23 | 1.00 | 1.04 | 1.00 |
|  | Poor | 1.45 | 1.00 | 1.52 | 1.00 |
| Immigration status | (Ref: Born in this country) |  |  |  |  |
|  | Born in another country | 1.30 | 1.00 | 1.27 | 1.00 |
| Age 12 religious service attendance | (Ref: Never) |  |  |  |  |
|  | At least 1/week | 1.68 | 1.38 | 1.48 | 1.00 |
|  | 1-3/month | 1.62 | 1.28 | 1.45 | 1.00 |
|  | < 1/month | 1.31 | 1.00 | 1.09 | 1.00 |
| Year of birth | (Ref: 1998-2005; current age: 18-24) |  |  |  |  |
|  | 1993-1998; age 25-29 | 1.39 | 1.18 | 1.30 | 1.00 |
|  | 1983-1993; age 30-39 | 1.40 | 1.23 | 1.39 | 1.00 |
|  | 1973-1983; age 40-49 | 1.47 | 1.31 | 1.52 | 1.23 |
|  | 1963-1973; age 50-59 | 1.46 | 1.27 | 2.01 | 1.67 |
|  | 1953-1963; age 60-69 | 1.54 | 1.29 | 2.37 | 1.77 |
|  | 1943-1953; age 70-79 | 1.44 | 1.00 | 3.84 | 1.74 |
|  | 1943 or earlier; age 80+ | 2.30 | 2.00 | 1.47 | 1.00 |
| Gender | (Ref: Male) |  |  |  |  |
|  | Female | 1.30 | 1.16 | 1.54 | 1.35 |
|  | Other | 1.42 | 1.00 | 2.33 | 1.00 |
| Religious affiliation | (Ref: Islam) |  |  |  |  |
|  | Christianity | 2.26 | 2.01 | 1.04 | 1.00 |
|  | Collapsed affiliations with prevalence<3% | 2.32 | 1.34 | 1.85 | 1.00 |
| Race/ethnicity | (Ref: Plurality group) |  |  |  |  |
|  | Non-plurality groups | 1.11 | 1.00 | 1.38 | 1.13 |

***Table S9a. Nationally representative descriptive statistics for Israel***

| **Characteristic** | **N = 3,669**^1^ |
| --- | --- |
| **Relationship with mother** |  |
| Very good | 2,686 (73%) |
| Somewhat good | 793 (22%) |
| Somewhat bad | 110 (3.0%) |
| Very bad | 18 (0.5%) |
| Does not apply | 45 (1.2%) |
| (Missing) | 17 (0.5%) |
| **Relationship with father** |  |
| Very good | 2,290 (62%) |
| Somewhat good | 912 (25%) |
| Somewhat bad | 234 (6.4%) |
| Very bad | 37 (1.0%) |
| Does not apply | 171 (4.7%) |
| (Missing) | 25 (0.7%) |
| **Parent marital status** |  |
| Parents married | 3,172 (86%) |
| Divorced | 284 (7.8%) |
| Parents were never married | 36 (1.0%) |
| One or both parents had died | 130 (3.5%) |
| (Missing) | 47 (1.3%) |
| **Subjective financial status of family growing up** |  |
| Lived comfortably | 923 (25%) |
| Got by | 1,822 (50%) |
| Found it difficult | 667 (18%) |
| Found it very difficult | 239 (6.5%) |
| (Missing) | 17 (0.5%) |
| **Abuse** |  |
| Yes | 0 (0%) |
| No | 0 (0%) |
| (Missing) | 3,669 (100%) |
| **Outsider growing up** |  |
| Yes | 371 (10%) |
| No | 3,228 (88%) |
| (Missing) | 70 (1.9%) |
| **Self-rated health growing up** |  |
| Excellent | 1,785 (49%) |
| Very good | 1,284 (35%) |
| Good | 480 (13%) |
| Fair | 105 (2.9%) |
| Poor | 6 (0.2%) |
| (Missing) | 8 (0.2%) |
| **Immigration status** |  |
| Born in this country | 2,796 (76%) |
| Born in another country | 868 (24%) |
| (Missing) | 5 (0.1%) |
| **Age 12 religious service attendance** |  |
| At least 1/week | 867 (24%) |
| 1-3/month | 435 (12%) |
| <1/month | 810 (22%) |
| Never | 1,539 (42%) |
| (Missing) | 17 (0.5%) |
| **Year of birth** |  |
| 1998-2005; age 18-24 | 553 (15%) |
| 1993-1998; age 25-29 | 407 (11%) |
| 1983-1993; age 30-39 | 666 (18%) |
| 1973-1983; age 40-49 | 616 (17%) |
| 1963-1973; age 50-59 | 542 (15%) |
| 1953-1963; age 60-69 | 469 (13%) |
| 1943-1953; age 70-79 | 336 (9.2%) |
| 1943 or earlier; age 80+ | 79 (2.2%) |
| (Missing) | 0 (0%) |
| **Gender** |  |
| Male | 1,791 (49%) |
| Female | 1,872 (51%) |
| Other | 0 (<0.1%) |
| (Missing) | 6 (0.2%) |
| **Religious affiliation** |  |
| Christianity | 60 (1.6%) |
| Islam | 647 (18%) |
| Hinduism | 0 (0%) |
| Buddhism | 0 (0%) |
| Judaism | 2,873 (78%) |
| Sikhism | 1 (<0.1%) |
| Baha'i | 1 (<0.1%) |
| Jainism | 0 (0%) |
| Shinto | 0 (0%) |
| Taoism | 0 (0%) |
| Confucianism | 0 (0%) |
| Primal, Animist, or Folk religion | 3 (<0.1%) |
| Spiritism | 0 (0%) |
| Umbanda, Candomble, and other African-derived religions | 0 (0%) |
| Chinese folk/traditional religion | 0 (0%) |
| Some other religion | 5 (0.1%) |
| No religion/Atheist/Agnostic | 69 (1.9%) |
| (Missing) | 10 (0.3%) |
| **Race/Ethnicity** |  |
| Arab | 674 (18%) |
| Jewish | 2,926 (80%) |
| Other | 39 (1.1%) |
| (Missing) | 30 (0.8%) |
| ^1^n (%) | |

***Table S9b. Childhood predictors regression analysis results for Israel***

|  | | Charitable giving | | | | Helping | | | |
| --- | --- | --- | --- | --- | --- | --- | --- | --- | --- |
| Variable | Category | Risk-Ratio | RR 95% CI | log(RR) SE | Global p-value | Risk-Ratio | RR 95% CI | log(RR) SE | Global p-value |
| Relationship with mother | (Ref: Very bad/somewhat bad) |  |  |  | 0.003 |  |  |  | 0.752 |
|  | Very good/somewhat good | 0.79 | (0.67,0.93) | 0.08 |  | 0.98 | (0.83,1.15) | 0.08 |  |
| Relationship with father | (Ref: Very bad/somewhat bad) |  |  |  | 0.084 |  |  |  | 0.236 |
|  | Very good/somewhat good | 1.13 | (0.98,1.30) | 0.07 |  | 1.08 | (0.95,1.22) | 0.06 |  |
| Parent marital status | (Ref: Parents married) |  |  |  | 0.018 |  |  |  | 0.189 |
|  | Divorced | 0.80 | (0.67,0.96) | 0.09 |  | 1.10 | (1.00,1.21) | 0.05 |  |
|  | Parents were never married | 0.59 | (0.31,1.11) | 0.32 |  | 0.99 | (0.73,1.32) | 0.15 |  |
|  | One or both parents had died | 1.05 | (0.90,1.24) | 0.08 |  | 1.07 | (0.92,1.24) | 0.08 |  |
| Subjective financial status of family growing up | (Ref: Got by) |  |  |  | 0.275 |  |  |  | 0.014 |
|  | Lived comfortably | 1.01 | (0.93,1.09) | 0.04 |  | 1.06 | (0.99,1.13) | 0.04 |  |
|  | Found it difficult | 0.90 | (0.82,1.00) | 0.05 |  | 0.94 | (0.87,1.00) | 0.04 |  |
|  | Found it very difficult | 0.98 | (0.80,1.19) | 0.10 |  | 1.03 | (0.88,1.22) | 0.08 |  |
| Outsider growing up | (Ref: No) |  |  |  | 0.654 |  |  |  | 0.329 |
|  | Yes | 0.98 | (0.87,1.10) | 0.06 |  | 1.05 | (0.95,1.16) | 0.05 |  |
| Self-rated health growing up | (Ref: Good) |  |  |  | 0.002 |  |  |  | <.001 |
|  | Excellent | 1.29 | (1.09,1.52) | 0.08 |  | 1.19 | (1.04,1.36) | 0.07 |  |
|  | Very good | 1.25 | (1.08,1.45) | 0.07 |  | 1.30 | (1.16,1.47) | 0.06 |  |
|  | Fair | 1.04 | (0.83,1.30) | 0.11 |  | 1.13 | (0.90,1.41) | 0.11 |  |
|  | Poor | 0.32 | (0.12,0.83) | 0.49 |  | 0.59 | (0.17,2.03) | 0.63 |  |
| Immigration status | (Ref: Born in this country) |  |  |  | 0.001 |  |  |  | <.001 |
|  | Born in another country | 0.83 | (0.74,0.93) | 0.06 |  | 0.79 | (0.72,0.86) | 0.04 |  |
| Age 12 religious service attendance | (Ref: Never) |  |  |  | <.001 |  |  |  | <.001 |
|  | At least 1/week | 2.31 | (2.08,2.58) | 0.06 |  | 1.40 | (1.27,1.53) | 0.05 |  |
|  | 1-3/month | 2.11 | (1.88,2.38) | 0.06 |  | 1.38 | (1.25,1.53) | 0.05 |  |
|  | < 1/month | 1.69 | (1.50,1.90) | 0.06 |  | 1.22 | (1.12,1.34) | 0.05 |  |
| Year of birth | (Ref: 1998-2005; current age: 18-24) |  |  |  | <.001 |  |  |  | <.001 |
|  | 1993-1998; age 25-29 | 1.21 | (1.08,1.35) | 0.06 |  | 0.90 | (0.81,1.00) | 0.05 |  |
|  | 1983-1993; age 30-39 | 1.26 | (1.12,1.42) | 0.06 |  | 1.05 | (0.96,1.13) | 0.04 |  |
|  | 1973-1983; age 40-49 | 1.27 | (1.12,1.43) | 0.06 |  | 1.00 | (0.91,1.11) | 0.05 |  |
|  | 1963-1973; age 50-59 | 1.30 | (1.16,1.47) | 0.06 |  | 1.10 | (1.01,1.21) | 0.05 |  |
|  | 1953-1963; age 60-69 | 1.56 | (1.39,1.76) | 0.06 |  | 0.96 | (0.85,1.08) | 0.06 |  |
|  | 1943-1953; age 70-79 | 1.32 | (1.13,1.55) | 0.08 |  | 0.86 | (0.75,1.00) | 0.07 |  |
|  | 1943 or earlier; age 80+ | 1.15 | (0.87,1.51) | 0.14 |  | 0.52 | (0.33,0.81) | 0.23 |  |
| Gender | (Ref: Male) |  |  |  | <.001 |  |  |  | <.001 |
|  | Female | 1.09 | (1.02,1.17) | 0.03 |  | 0.99 | (0.94,1.04) | 0.03 |  |
|  | Other | 0.00 | (0.00,0.00) | 1.00 |  | 0.02 | (0.00,5787369.54) | 6.81 |  |
| Religious affiliation | (Ref: Judaism) |  |  |  | 0.149 |  |  |  | 0.642 |
|  | Islam | 1.06 | (0.57,1.97) | 0.31 |  | 0.95 | (0.64,1.42) | 0.20 |  |
|  | Collapsed affiliations with prevalence<3% | 0.73 | (0.49,1.09) | 0.20 |  | 0.88 | (0.66,1.19) | 0.15 |  |
| Race/ethnicity | (Ref: Plurality group) |  |  |  | 0.315 |  |  |  | 0.526 |
|  | Non-plurality groups | 0.81 | (0.42,1.54) | 0.32 |  | 0.90 | (0.59,1.36) | 0.21 |  |
| Abuse | (Ref: No) |  |  |  |  |  |  |  |  |

***Table S9c. Sensitivity to unmeasured confounding of childhood predictors in Israel***

|  | | Charitable giving | | Helping | |
| --- | --- | --- | --- | --- | --- |
| Variable | Category | E-value for Estimate | E-value for 95% CI | E-value for Estimate | E-value for 95% CI |
| Relationship with mother | (Ref: Very bad/somewhat bad) |  |  |  |  |
|  | Very good/somewhat good | 1.85 | 1.37 | 1.17 | 1.00 |
| Relationship with father | (Ref: Very bad/somewhat bad) |  |  |  |  |
|  | Very good/somewhat good | 1.51 | 1.00 | 1.36 | 1.00 |
| Parent marital status | (Ref: Parents married) |  |  |  |  |
|  | Divorced | 1.80 | 1.27 | 1.43 | 1.05 |
|  | Parents were never married | 2.77 | 1.00 | 1.14 | 1.00 |
|  | One or both parents had died | 1.29 | 1.00 | 1.34 | 1.00 |
| Subjective financial status of family growing up | (Ref: Got by) |  |  |  |  |
|  | Lived comfortably | 1.08 | 1.00 | 1.31 | 1.00 |
|  | Found it difficult | 1.45 | 1.00 | 1.34 | 1.00 |
|  | Found it very difficult | 1.19 | 1.00 | 1.22 | 1.00 |
| Outsider growing up | (Ref: No) |  |  |  |  |
|  | Yes | 1.18 | 1.00 | 1.28 | 1.00 |
| Self-rated health growing up | (Ref: Good) |  |  |  |  |
|  | Excellent | 1.90 | 1.41 | 1.65 | 1.23 |
|  | Very good | 1.82 | 1.39 | 1.93 | 1.58 |
|  | Fair | 1.25 | 1.00 | 1.51 | 1.00 |
|  | Poor | 5.73 | 1.72 | 2.79 | 1.00 |
| Immigration status | (Ref: Born in this country) |  |  |  |  |
|  | Born in another country | 1.70 | 1.37 | 1.86 | 1.61 |
| Age 12 religious service attendance | (Ref: Never) |  |  |  |  |
|  | At least 1/week | 4.06 | 3.57 | 2.14 | 1.85 |
|  | 1-3/month | 3.65 | 3.16 | 2.11 | 1.82 |
|  | < 1/month | 2.77 | 2.37 | 1.74 | 1.48 |
| Year of birth | (Ref: 1998-2005; current age: 18-24) |  |  |  |  |
|  | 1993-1998; age 25-29 | 1.71 | 1.38 | 1.45 | 1.00 |
|  | 1983-1993; age 30-39 | 1.84 | 1.49 | 1.26 | 1.00 |
|  | 1973-1983; age 40-49 | 1.85 | 1.49 | 1.06 | 1.00 |
|  | 1963-1973; age 50-59 | 1.93 | 1.59 | 1.44 | 1.09 |
|  | 1953-1963; age 60-69 | 2.50 | 2.13 | 1.26 | 1.00 |
|  | 1943-1953; age 70-79 | 1.97 | 1.51 | 1.59 | 1.06 |
|  | 1943 or earlier; age 80+ | 1.56 | 1.00 | 3.28 | 1.79 |
| Gender | (Ref: Male) |  |  |  |  |
|  | Female | 1.41 | 1.16 | 1.11 | 1.00 |
|  | Other | 65090.46 | 9202.95 | 124.63 | 1.00 |
| Religious affiliation | (Ref: Judaism) |  |  |  |  |
|  | Islam | 1.30 | 1.00 | 1.27 | 1.00 |
|  | Collapsed affiliations with prevalence<3% | 2.08 | 1.00 | 1.52 | 1.00 |
| Race/ethnicity | (Ref: Plurality group) |  |  |  |  |
|  | Non-plurality groups | 1.79 | 1.00 | 1.46 | 1.00 |
| Abuse | (Ref: No) |  |  |  |  |

***Table S10a. Nationally representative descriptive statistics for Japan***

| **Characteristic** | **N = 20,543**^1^ |
| --- | --- |
| **Relationship with mother** |  |
| Very good | 5,630 (27%) |
| Somewhat good | 9,461 (46%) |
| Somewhat bad | 2,750 (13%) |
| Very bad | 799 (3.9%) |
| Does not apply | 1,838 (8.9%) |
| (Missing) | 66 (0.3%) |
| **Relationship with father** |  |
| Very good | 4,156 (20%) |
| Somewhat good | 9,081 (44%) |
| Somewhat bad | 3,446 (17%) |
| Very bad | 1,223 (6.0%) |
| Does not apply | 2,580 (13%) |
| (Missing) | 57 (0.3%) |
| **Parent marital status** |  |
| Parents married | 17,713 (86%) |
| Divorced | 1,127 (5.5%) |
| Parents were never married | 591 (2.9%) |
| One or both parents had died | 754 (3.7%) |
| (Missing) | 359 (1.7%) |
| **Subjective financial status of family growing up** |  |
| Lived comfortably | 8,320 (41%) |
| Got by | 8,799 (43%) |
| Found it difficult | 2,398 (12%) |
| Found it very difficult | 973 (4.7%) |
| (Missing) | 52 (0.3%) |
| **Abuse** |  |
| Yes | 1,482 (7.2%) |
| No | 18,964 (92%) |
| (Missing) | 96 (0.5%) |
| **Outsider growing up** |  |
| Yes | 1,963 (9.6%) |
| No | 17,136 (83%) |
| (Missing) | 1,444 (7.0%) |
| **Self-rated health growing up** |  |
| Excellent | 2,711 (13%) |
| Very good | 7,106 (35%) |
| Good | 6,689 (33%) |
| Fair | 3,199 (16%) |
| Poor | 758 (3.7%) |
| (Missing) | 80 (0.4%) |
| **Immigration status** |  |
| Born in this country | 19,548 (95%) |
| Born in another country | 158 (0.8%) |
| (Missing) | 837 (4.1%) |
| **Age 12 religious service attendance** |  |
| At least 1/week | 398 (1.9%) |
| 1-3/month | 883 (4.3%) |
| <1/month | 5,023 (24%) |
| Never | 14,117 (69%) |
| (Missing) | 123 (0.6%) |
| **Year of birth** |  |
| 1998-2005; age 18-24 | 1,589 (7.7%) |
| 1993-1998; age 25-29 | 806 (3.9%) |
| 1983-1993; age 30-39 | 2,851 (14%) |
| 1973-1983; age 40-49 | 3,363 (16%) |
| 1963-1973; age 50-59 | 3,770 (18%) |
| 1953-1963; age 60-69 | 4,118 (20%) |
| 1943-1953; age 70-79 | 3,554 (17%) |
| 1943 or earlier; age 80+ | 493 (2.4%) |
| (Missing) | 0 (0%) |
| **Gender** |  |
| Male | 9,847 (48%) |
| Female | 10,602 (52%) |
| Other | 28 (0.1%) |
| (Missing) | 66 (0.3%) |
| **Religious affiliation** |  |
| Christianity | 343 (1.7%) |
| Islam | 7 (<0.1%) |
| Hinduism | 4 (<0.1%) |
| Buddhism | 6,536 (32%) |
| Judaism | 0 (0%) |
| Sikhism | 0 (0%) |
| Baha'i | 7 (<0.1%) |
| Jainism | 1 (<0.1%) |
| Shinto | 382 (1.9%) |
| Taoism | 14 (<0.1%) |
| Confucianism | 25 (0.1%) |
| Primal, Animist, or Folk religion | 13 (<0.1%) |
| Spiritism | 0 (0%) |
| Umbanda, Candomble, and other African-derived religions | 0 (0%) |
| Chinese folk/traditional religion | 0 (0%) |
| Some other religion | 46 (0.2%) |
| No religion/Atheist/Agnostic | 12,950 (63%) |
| (Missing) | 215 (1.0%) |
| ^1^n (%) | |

***Table S10b. Childhood predictors regression analysis results for Japan***

|  | | Charitable giving | | | | Helping | | | |
| --- | --- | --- | --- | --- | --- | --- | --- | --- | --- |
| Variable | Category | Risk-Ratio | RR 95% CI | log(RR) SE | Global p-value | Risk-Ratio | RR 95% CI | log(RR) SE | Global p-value |
| Relationship with mother | (Ref: Very bad/somewhat bad) |  |  |  | 0.001 |  |  |  | 0.178 |
|  | Very good/somewhat good | 1.30 | (1.10,1.54) | 0.09 |  | 1.10 | (0.96,1.26) | 0.07 |  |
| Relationship with father | (Ref: Very bad/somewhat bad) |  |  |  | 0.142 |  |  |  | 0.201 |
|  | Very good/somewhat good | 1.11 | (0.96,1.28) | 0.07 |  | 1.08 | (0.96,1.23) | 0.06 |  |
| Parent marital status | (Ref: Parents married) |  |  |  | 0.526 |  |  |  | 0.450 |
|  | Divorced | 1.01 | (0.78,1.30) | 0.13 |  | 0.99 | (0.82,1.21) | 0.10 |  |
|  | Parents were never married | 1.19 | (0.92,1.55) | 0.13 |  | 1.16 | (0.94,1.43) | 0.11 |  |
|  | One or both parents had died | 1.09 | (0.83,1.42) | 0.14 |  | 1.10 | (0.86,1.42) | 0.13 |  |
| Subjective financial status of family growing up | (Ref: Got by) |  |  |  | 0.146 |  |  |  | 0.075 |
|  | Lived comfortably | 1.12 | (1.00,1.25) | 0.06 |  | 1.13 | (1.02,1.25) | 0.05 |  |
|  | Found it difficult | 0.98 | (0.83,1.16) | 0.09 |  | 1.12 | (0.96,1.30) | 0.08 |  |
|  | Found it very difficult | 1.12 | (0.87,1.44) | 0.13 |  | 0.97 | (0.77,1.22) | 0.12 |  |
| Abuse | (Ref: No) |  |  |  | <.001 |  |  |  | <.001 |
|  | Yes | 1.58 | (1.33,1.87) | 0.09 |  | 1.59 | (1.38,1.83) | 0.07 |  |
| Outsider growing up | (Ref: No) |  |  |  | <.001 |  |  |  | <.001 |
|  | Yes | 1.46 | (1.24,1.72) | 0.08 |  | 1.29 | (1.13,1.49) | 0.07 |  |
| Self-rated health growing up | (Ref: Good) |  |  |  | 0.086 |  |  |  | 0.034 |
|  | Excellent | 1.20 | (1.02,1.41) | 0.08 |  | 1.20 | (1.03,1.39) | 0.08 |  |
|  | Very good | 1.06 | (0.93,1.19) | 0.06 |  | 1.16 | (1.03,1.30) | 0.06 |  |
|  | Fair | 0.98 | (0.83,1.16) | 0.08 |  | 1.12 | (0.98,1.29) | 0.07 |  |
|  | Poor | 1.23 | (0.95,1.59) | 0.13 |  | 1.28 | (1.03,1.59) | 0.11 |  |
| Immigration status | (Ref: Born in this country) |  |  |  | 0.061 |  |  |  | 0.135 |
|  | Born in another country | 1.51 | (0.97,2.37) | 0.23 |  | 1.37 | (0.90,2.10) | 0.22 |  |
| Age 12 religious service attendance | (Ref: Never) |  |  |  | <.001 |  |  |  | <.001 |
|  | At least 1/week | 2.20 | (1.72,2.80) | 0.12 |  | 2.33 | (1.88,2.89) | 0.11 |  |
|  | 1-3/month | 2.56 | (2.16,3.04) | 0.09 |  | 2.52 | (2.16,2.94) | 0.08 |  |
|  | < 1/month | 1.73 | (1.55,1.94) | 0.06 |  | 1.61 | (1.45,1.79) | 0.05 |  |
| Year of birth | (Ref: 1998-2005; current age: 18-24) |  |  |  | <.001 |  |  |  | <.001 |
|  | 1993-1998; age 25-29 | 0.93 | (0.67,1.28) | 0.17 |  | 0.70 | (0.55,0.88) | 0.12 |  |
|  | 1983-1993; age 30-39 | 0.93 | (0.72,1.18) | 0.12 |  | 0.77 | (0.65,0.92) | 0.09 |  |
|  | 1973-1983; age 40-49 | 0.78 | (0.61,1.00) | 0.12 |  | 0.72 | (0.61,0.84) | 0.08 |  |
|  | 1963-1973; age 50-59 | 0.80 | (0.63,1.01) | 0.12 |  | 0.61 | (0.52,0.72) | 0.09 |  |
|  | 1953-1963; age 60-69 | 1.16 | (0.93,1.45) | 0.11 |  | 0.56 | (0.47,0.66) | 0.08 |  |
|  | 1943-1953; age 70-79 | 1.76 | (1.42,2.19) | 0.11 |  | 0.56 | (0.47,0.67) | 0.09 |  |
|  | 1943 or earlier; age 80+ | 1.78 | (1.31,2.41) | 0.15 |  | 0.50 | (0.35,0.72) | 0.18 |  |
| Gender | (Ref: Male) |  |  |  | 0.093 |  |  |  | 0.742 |
|  | Female | 0.91 | (0.82,1.00) | 0.05 |  | 1.02 | (0.94,1.12) | 0.05 |  |
|  | Other | 1.63 | (0.61,4.35) | 0.50 |  | 0.78 | (0.32,1.90) | 0.46 |  |
| Religious affiliation | (Ref: No religion/Atheist/Agnostic) |  |  |  | <.001 |  |  |  | <.001 |
|  | Buddhism | 1.25 | (1.12,1.40) | 0.06 |  | 1.10 | (0.99,1.22) | 0.05 |  |
|  | Collapsed affiliations with prevalence<3% | 1.83 | (1.51,2.22) | 0.10 |  | 1.57 | (1.33,1.84) | 0.08 |  |
| Race/ethnicity | (Ref: Plurality group) |  |  |  |  |  |  |  |  |

***Table S10c. Sensitivity to unmeasured confounding of childhood predictors in Japan***

|  | | Charitable giving | | Helping | |
| --- | --- | --- | --- | --- | --- |
| Variable | Category | E-value for Estimate | E-value for 95% CI | E-value for Estimate | E-value for 95% CI |
| Relationship with mother | (Ref: Very bad/somewhat bad) |  |  |  |  |
|  | Very good/somewhat good | 1.93 | 1.43 | 1.42 | 1.00 |
| Relationship with father | (Ref: Very bad/somewhat bad) |  |  |  |  |
|  | Very good/somewhat good | 1.46 | 1.00 | 1.38 | 1.00 |
| Parent marital status | (Ref: Parents married) |  |  |  |  |
|  | Divorced | 1.08 | 1.00 | 1.09 | 1.00 |
|  | Parents were never married | 1.67 | 1.00 | 1.60 | 1.00 |
|  | One or both parents had died | 1.39 | 1.00 | 1.44 | 1.00 |
| Subjective financial status of family growing up | (Ref: Got by) |  |  |  |  |
|  | Lived comfortably | 1.49 | 1.06 | 1.52 | 1.17 |
|  | Found it difficult | 1.17 | 1.00 | 1.47 | 1.00 |
|  | Found it very difficult | 1.49 | 1.00 | 1.21 | 1.00 |
| Abuse | (Ref: No) |  |  |  |  |
|  | Yes | 2.53 | 1.99 | 2.55 | 2.10 |
| Outsider growing up | (Ref: No) |  |  |  |  |
|  | Yes | 2.28 | 1.78 | 1.91 | 1.50 |
| Self-rated health growing up | (Ref: Good) |  |  |  |  |
|  | Excellent | 1.70 | 1.18 | 1.68 | 1.21 |
|  | Very good | 1.30 | 1.00 | 1.58 | 1.20 |
|  | Fair | 1.14 | 1.00 | 1.49 | 1.00 |
|  | Poor | 1.76 | 1.00 | 1.89 | 1.22 |
| Immigration status | (Ref: Born in this country) |  |  |  |  |
|  | Born in another country | 2.39 | 1.00 | 2.09 | 1.00 |
| Age 12 religious service attendance | (Ref: Never) |  |  |  |  |
|  | At least 1/week | 3.82 | 2.83 | 4.09 | 3.17 |
|  | 1-3/month | 4.57 | 3.75 | 4.48 | 3.74 |
|  | < 1/month | 2.86 | 2.47 | 2.61 | 2.27 |
| Year of birth | (Ref: 1998-2005; current age: 18-24) |  |  |  |  |
|  | 1993-1998; age 25-29 | 1.38 | 1.00 | 2.22 | 1.52 |
|  | 1983-1993; age 30-39 | 1.38 | 1.00 | 1.91 | 1.41 |
|  | 1973-1983; age 40-49 | 1.88 | 1.07 | 2.14 | 1.65 |
|  | 1963-1973; age 50-59 | 1.81 | 1.00 | 2.65 | 2.11 |
|  | 1953-1963; age 60-69 | 1.59 | 1.00 | 2.97 | 2.39 |
|  | 1943-1953; age 70-79 | 2.92 | 2.19 | 2.97 | 2.36 |
|  | 1943 or earlier; age 80+ | 2.95 | 1.95 | 3.39 | 2.14 |
| Gender | (Ref: Male) |  |  |  |  |
|  | Female | 1.43 | 1.00 | 1.18 | 1.00 |
|  | Other | 2.64 | 1.00 | 1.90 | 1.00 |
| Religious affiliation | (Ref: No religion/Atheist/Agnostic) |  |  |  |  |
|  | Buddhism | 1.81 | 1.48 | 1.44 | 1.00 |
|  | Collapsed affiliations with prevalence<3% | 3.06 | 2.38 | 2.51 | 1.99 |
| Race/ethnicity | (Ref: Plurality group) |  |  |  |  |

***Table S11a. Nationally representative descriptive statistics for Kenya***

| **Characteristic** | **N = 11,389**^1^ |
| --- | --- |
| **Relationship with mother** |  |
| Very good | 9,418 (83%) |
| Somewhat good | 1,435 (13%) |
| Somewhat bad | 130 (1.1%) |
| Very bad | 100 (0.9%) |
| Does not apply | 240 (2.1%) |
| (Missing) | 66 (0.6%) |
| **Relationship with father** |  |
| Very good | 7,958 (70%) |
| Somewhat good | 1,896 (17%) |
| Somewhat bad | 216 (1.9%) |
| Very bad | 220 (1.9%) |
| Does not apply | 967 (8.5%) |
| (Missing) | 132 (1.2%) |
| **Parent marital status** |  |
| Parents married | 9,238 (81%) |
| Divorced | 697 (6.1%) |
| Parents were never married | 681 (6.0%) |
| One or both parents had died | 471 (4.1%) |
| (Missing) | 301 (2.6%) |
| **Subjective financial status of family growing up** |  |
| Lived comfortably | 3,026 (27%) |
| Got by | 3,279 (29%) |
| Found it difficult | 4,071 (36%) |
| Found it very difficult | 994 (8.7%) |
| (Missing) | 19 (0.2%) |
| **Abuse** |  |
| Yes | 1,300 (11%) |
| No | 10,039 (88%) |
| (Missing) | 49 (0.4%) |
| **Outsider growing up** |  |
| Yes | 1,223 (11%) |
| No | 10,114 (89%) |
| (Missing) | 52 (0.5%) |
| **Self-rated health growing up** |  |
| Excellent | 4,449 (39%) |
| Very good | 2,598 (23%) |
| Good | 2,582 (23%) |
| Fair | 1,384 (12%) |
| Poor | 349 (3.1%) |
| (Missing) | 26 (0.2%) |
| **Immigration status** |  |
| Born in this country | 11,270 (99%) |
| Born in another country | 117 (1.0%) |
| (Missing) | 2 (<0.1%) |
| **Age 12 religious service attendance** |  |
| At least 1/week | 9,189 (81%) |
| 1-3/month | 1,687 (15%) |
| <1/month | 236 (2.1%) |
| Never | 198 (1.7%) |
| (Missing) | 79 (0.7%) |
| **Year of birth** |  |
| 1998-2005; age 18-24 | 2,868 (25%) |
| 1993-1998; age 25-29 | 2,035 (18%) |
| 1983-1993; age 30-39 | 2,564 (23%) |
| 1973-1983; age 40-49 | 1,708 (15%) |
| 1963-1973; age 50-59 | 1,072 (9.4%) |
| 1953-1963; age 60-69 | 710 (6.2%) |
| 1943-1953; age 70-79 | 360 (3.2%) |
| 1943 or earlier; age 80+ | 67 (0.6%) |
| (Missing) | 5 (<0.1%) |
| **Gender** |  |
| Male | 5,567 (49%) |
| Female | 5,813 (51%) |
| Other | 2 (<0.1%) |
| (Missing) | 7 (<0.1%) |
| **Religious affiliation** |  |
| Christianity | 10,369 (91%) |
| Islam | 916 (8.0%) |
| Hinduism | 0 (0%) |
| Buddhism | 5 (<0.1%) |
| Judaism | 6 (<0.1%) |
| Sikhism | 0 (<0.1%) |
| Baha'i | 3 (<0.1%) |
| Jainism | 1 (<0.1%) |
| Shinto | 0 (0%) |
| Taoism | 0 (0%) |
| Confucianism | 0 (0%) |
| Primal, Animist, or Folk religion | 13 (0.1%) |
| Spiritism | 0 (0%) |
| Umbanda, Candomble, and other African-derived religions | 0 (0%) |
| Chinese folk/traditional religion | 0 (0%) |
| Some other religion | 0 (<0.1%) |
| No religion/Atheist/Agnostic | 67 (0.6%) |
| (Missing) | 9 (<0.1%) |
| **Race/Ethnicity** |  |
| Embu | 197 (1.7%) |
| Kalenjin | 1,377 (12%) |
| Kamba | 1,299 (11%) |
| Kenyan Somali/Somali | 396 (3.5%) |
| Kikuyu | 2,119 (19%) |
| Kisii | 789 (6.9%) |
| Luhya | 1,943 (17%) |
| Luo | 1,120 (9.8%) |
| Maasai | 237 (2.1%) |
| Meru | 630 (5.5%) |
| Miji Kenda tribes | 708 (6.2%) |
| Other | 548 (4.8%) |
| (Missing) | 27 (0.2%) |
| ^1^n (%) | |

***Table S11b. Childhood predictors regression analysis results for Kenya***

|  | | Charitable giving | | | | Helping | | | |
| --- | --- | --- | --- | --- | --- | --- | --- | --- | --- |
| Variable | Category | Risk-Ratio | RR 95% CI | log(RR) SE | Global p-value | Risk-Ratio | RR 95% CI | log(RR) SE | Global p-value |
| Relationship with mother | (Ref: Very bad/somewhat bad) |  |  |  | 0.810 |  |  |  | 0.026 |
|  | Very good/somewhat good | 0.98 | (0.74,1.28) | 0.14 |  | 1.13 | (1.01,1.27) | 0.06 |  |
| Relationship with father | (Ref: Very bad/somewhat bad) |  |  |  | 0.682 |  |  |  | 0.791 |
|  | Very good/somewhat good | 0.97 | (0.83,1.14) | 0.08 |  | 0.99 | (0.93,1.07) | 0.04 |  |
| Parent marital status | (Ref: Parents married) |  |  |  | 0.867 |  |  |  | 0.647 |
|  | Divorced | 1.02 | (0.88,1.19) | 0.08 |  | 1.03 | (0.97,1.09) | 0.03 |  |
|  | Parents were never married | 0.96 | (0.83,1.11) | 0.07 |  | 1.02 | (0.95,1.09) | 0.03 |  |
|  | One or both parents had died | 0.94 | (0.77,1.15) | 0.10 |  | 1.02 | (0.95,1.11) | 0.04 |  |
| Subjective financial status of family growing up | (Ref: Got by) |  |  |  | 0.003 |  |  |  | 0.233 |
|  | Lived comfortably | 1.18 | (1.07,1.31) | 0.05 |  | 1.00 | (0.96,1.04) | 0.02 |  |
|  | Found it difficult | 1.02 | (0.92,1.12) | 0.05 |  | 0.97 | (0.93,1.01) | 0.02 |  |
|  | Found it very difficult | 1.05 | (0.90,1.23) | 0.08 |  | 1.02 | (0.95,1.08) | 0.03 |  |
| Abuse | (Ref: No) |  |  |  | 0.116 |  |  |  | 0.002 |
|  | Yes | 1.10 | (0.98,1.23) | 0.06 |  | 1.07 | (1.02,1.12) | 0.02 |  |
| Outsider growing up | (Ref: No) |  |  |  | 0.014 |  |  |  | 0.215 |
|  | Yes | 1.15 | (1.03,1.29) | 0.06 |  | 1.04 | (0.98,1.11) | 0.03 |  |
| Self-rated health growing up | (Ref: Good) |  |  |  | <.001 |  |  |  | 0.657 |
|  | Excellent | 0.96 | (0.85,1.07) | 0.06 |  | 1.01 | (0.96,1.06) | 0.02 |  |
|  | Very good | 1.07 | (0.96,1.20) | 0.06 |  | 0.98 | (0.93,1.03) | 0.03 |  |
|  | Fair | 0.77 | (0.67,0.89) | 0.07 |  | 0.98 | (0.92,1.04) | 0.03 |  |
|  | Poor | 1.07 | (0.84,1.36) | 0.12 |  | 1.01 | (0.91,1.12) | 0.05 |  |
| Immigration status | (Ref: Born in this country) |  |  |  | 0.492 |  |  |  | 0.949 |
|  | Born in another country | 0.86 | (0.56,1.32) | 0.22 |  | 1.00 | (0.86,1.15) | 0.07 |  |
| Age 12 religious service attendance | (Ref: Never) |  |  |  | 0.803 |  |  |  | 0.050 |
|  | At least 1/week | 1.01 | (0.73,1.38) | 0.16 |  | 1.02 | (0.89,1.17) | 0.07 |  |
|  | 1-3/month | 0.95 | (0.70,1.31) | 0.16 |  | 0.97 | (0.84,1.12) | 0.08 |  |
|  | < 1/month | 0.97 | (0.66,1.42) | 0.19 |  | 0.90 | (0.75,1.07) | 0.09 |  |
| Year of birth | (Ref: 1998-2005; current age: 18-24) |  |  |  | <.001 |  |  |  | <.001 |
|  | 1993-1998; age 25-29 | 1.11 | (1.02,1.21) | 0.04 |  | 1.11 | (1.06,1.16) | 0.02 |  |
|  | 1983-1993; age 30-39 | 0.99 | (0.90,1.09) | 0.05 |  | 1.17 | (1.11,1.22) | 0.02 |  |
|  | 1973-1983; age 40-49 | 0.81 | (0.70,0.93) | 0.07 |  | 1.17 | (1.11,1.24) | 0.03 |  |
|  | 1963-1973; age 50-59 | 0.86 | (0.73,1.02) | 0.09 |  | 1.24 | (1.17,1.32) | 0.03 |  |
|  | 1953-1963; age 60-69 | 0.78 | (0.61,0.98) | 0.12 |  | 1.06 | (0.94,1.18) | 0.06 |  |
|  | 1943-1953; age 70-79 | 0.75 | (0.55,1.03) | 0.16 |  | 1.15 | (1.00,1.33) | 0.07 |  |
|  | 1943 or earlier; age 80+ | 0.69 | (0.34,1.39) | 0.36 |  | 0.98 | (0.69,1.41) | 0.18 |  |
| Gender | (Ref: Male) |  |  |  | <.001 |  |  |  | <.001 |
|  | Female | 0.88 | (0.82,0.95) | 0.04 |  | 0.91 | (0.88,0.94) | 0.02 |  |
|  | Other | 0.00 | (0.00,0.00) | 0.82 |  | 1.17 | (0.67,2.05) | 0.29 |  |
| Religious affiliation | (Ref: Christianity) |  |  |  | 0.155 |  |  |  | 0.905 |
|  | Islam | 1.21 | (1.00,1.47) | 0.10 |  | 0.99 | (0.90,1.10) | 0.05 |  |
|  | Collapsed affiliations with prevalence<3% | 1.10 | (0.71,1.68) | 0.22 |  | 1.05 | (0.85,1.29) | 0.11 |  |
| Race/ethnicity | (Ref: Plurality group) |  |  |  | 0.895 |  |  |  | 0.042 |
|  | Non-plurality groups | 1.00 | (0.87,1.14) | 0.07 |  | 0.95 | (0.91,1.00) | 0.02 |  |

***Table S11c. Sensitivity to unmeasured confounding of childhood predictors in Kenya***

|  | | Charitable giving | | Helping | |
| --- | --- | --- | --- | --- | --- |
| Variable | Category | E-value for Estimate | E-value for 95% CI | E-value for Estimate | E-value for 95% CI |
| Relationship with mother | (Ref: Very bad/somewhat bad) |  |  |  |  |
|  | Very good/somewhat good | 1.18 | 1.00 | 1.52 | 1.13 |
| Relationship with father | (Ref: Very bad/somewhat bad) |  |  |  |  |
|  | Very good/somewhat good | 1.19 | 1.00 | 1.09 | 1.00 |
| Parent marital status | (Ref: Parents married) |  |  |  |  |
|  | Divorced | 1.18 | 1.00 | 1.21 | 1.00 |
|  | Parents were never married | 1.25 | 1.00 | 1.17 | 1.00 |
|  | One or both parents had died | 1.31 | 1.00 | 1.18 | 1.00 |
| Subjective financial status of family growing up | (Ref: Got by) |  |  |  |  |
|  | Lived comfortably | 1.65 | 1.33 | 1.04 | 1.00 |
|  | Found it difficult | 1.14 | 1.00 | 1.21 | 1.00 |
|  | Found it very difficult | 1.29 | 1.00 | 1.14 | 1.00 |
| Abuse | (Ref: No) |  |  |  |  |
|  | Yes | 1.43 | 1.00 | 1.35 | 1.18 |
| Outsider growing up | (Ref: No) |  |  |  |  |
|  | Yes | 1.57 | 1.20 | 1.24 | 1.00 |
| Self-rated health growing up | (Ref: Good) |  |  |  |  |
|  | Excellent | 1.26 | 1.00 | 1.10 | 1.00 |
|  | Very good | 1.36 | 1.00 | 1.16 | 1.00 |
|  | Fair | 1.90 | 1.48 | 1.18 | 1.00 |
|  | Poor | 1.34 | 1.00 | 1.12 | 1.00 |
| Immigration status | (Ref: Born in this country) |  |  |  |  |
|  | Born in another country | 1.60 | 1.00 | 1.07 | 1.00 |
| Age 12 religious service attendance | (Ref: Never) |  |  |  |  |
|  | At least 1/week | 1.09 | 1.00 | 1.16 | 1.00 |
|  | 1-3/month | 1.27 | 1.00 | 1.22 | 1.00 |
|  | < 1/month | 1.22 | 1.00 | 1.47 | 1.00 |
| Year of birth | (Ref: 1998-2005; current age: 18-24) |  |  |  |  |
|  | 1993-1998; age 25-29 | 1.46 | 1.16 | 1.46 | 1.31 |
|  | 1983-1993; age 30-39 | 1.12 | 1.00 | 1.60 | 1.47 |
|  | 1973-1983; age 40-49 | 1.78 | 1.37 | 1.62 | 1.45 |
|  | 1963-1973; age 50-59 | 1.60 | 1.00 | 1.79 | 1.61 |
|  | 1953-1963; age 60-69 | 1.90 | 1.16 | 1.30 | 1.00 |
|  | 1943-1953; age 70-79 | 2.00 | 1.00 | 1.57 | 1.00 |
|  | 1943 or earlier; age 80+ | 2.27 | 1.00 | 1.14 | 1.00 |
| Gender | (Ref: Male) |  |  |  |  |
|  | Female | 1.51 | 1.27 | 1.43 | 1.33 |
|  | Other | 131248.19 | 26454.06 | 1.62 | 1.00 |
| Religious affiliation | (Ref: Christianity) |  |  |  |  |
|  | Islam | 1.71 | 1.00 | 1.09 | 1.00 |
|  | Collapsed affiliations with prevalence<3% | 1.42 | 1.00 | 1.26 | 1.00 |
| Race/ethnicity | (Ref: Plurality group) |  |  |  |  |
|  | Non-plurality groups | 1.05 | 1.00 | 1.28 | 1.04 |

***Table S12a. Nationally representative descriptive statistics for Mexico***

| **Characteristic** | **N = 5,776**^1^ |
| --- | --- |
| **Relationship with mother** |  |
| Very good | 3,912 (68%) |
| Somewhat good | 1,340 (23%) |
| Somewhat bad | 177 (3.1%) |
| Very bad | 90 (1.6%) |
| Does not apply | 177 (3.1%) |
| (Missing) | 80 (1.4%) |
| **Relationship with father** |  |
| Very good | 3,089 (53%) |
| Somewhat good | 1,556 (27%) |
| Somewhat bad | 335 (5.8%) |
| Very bad | 267 (4.6%) |
| Does not apply | 470 (8.1%) |
| (Missing) | 60 (1.0%) |
| **Parent marital status** |  |
| Parents married | 3,999 (69%) |
| Divorced | 341 (5.9%) |
| Parents were never married | 827 (14%) |
| One or both parents had died | 176 (3.0%) |
| (Missing) | 432 (7.5%) |
| **Subjective financial status of family growing up** |  |
| Lived comfortably | 1,775 (31%) |
| Got by | 1,872 (32%) |
| Found it difficult | 1,712 (30%) |
| Found it very difficult | 369 (6.4%) |
| (Missing) | 48 (0.8%) |
| **Abuse** |  |
| Yes | 905 (16%) |
| No | 4,604 (80%) |
| (Missing) | 267 (4.6%) |
| **Outsider growing up** |  |
| Yes | 772 (13%) |
| No | 4,897 (85%) |
| (Missing) | 107 (1.9%) |
| **Self-rated health growing up** |  |
| Excellent | 1,860 (32%) |
| Very good | 1,350 (23%) |
| Good | 1,677 (29%) |
| Fair | 743 (13%) |
| Poor | 133 (2.3%) |
| (Missing) | 14 (0.2%) |
| **Immigration status** |  |
| Born in this country | 5,517 (96%) |
| Born in another country | 108 (1.9%) |
| (Missing) | 151 (2.6%) |
| **Age 12 religious service attendance** |  |
| At least 1/week | 2,514 (44%) |
| 1-3/month | 1,162 (20%) |
| <1/month | 1,087 (19%) |
| Never | 944 (16%) |
| (Missing) | 69 (1.2%) |
| **Year of birth** |  |
| 1998-2005; age 18-24 | 986 (17%) |
| 1993-1998; age 25-29 | 623 (11%) |
| 1983-1993; age 30-39 | 1,312 (23%) |
| 1973-1983; age 40-49 | 1,027 (18%) |
| 1963-1973; age 50-59 | 873 (15%) |
| 1953-1963; age 60-69 | 611 (11%) |
| 1943-1953; age 70-79 | 277 (4.8%) |
| 1943 or earlier; age 80+ | 68 (1.2%) |
| (Missing) | 0 (0%) |
| **Gender** |  |
| Male | 2,755 (48%) |
| Female | 2,997 (52%) |
| Other | 3 (<0.1%) |
| (Missing) | 21 (0.4%) |
| **Religious affiliation** |  |
| Christianity | 5,337 (92%) |
| Islam | 6 (<0.1%) |
| Hinduism | 1 (<0.1%) |
| Buddhism | 1 (<0.1%) |
| Judaism | 8 (0.1%) |
| Sikhism | 4 (<0.1%) |
| Baha'i | 1 (<0.1%) |
| Jainism | 0 (0%) |
| Shinto | 2 (<0.1%) |
| Taoism | 5 (<0.1%) |
| Confucianism | 0 (0%) |
| Primal, Animist, or Folk religion | 2 (<0.1%) |
| Spiritism | 0 (0%) |
| Umbanda, Candomble, and other African-derived religions | 0 (0%) |
| Chinese folk/traditional religion | 0 (0%) |
| Some other religion | 7 (0.1%) |
| No religion/Atheist/Agnostic | 328 (5.7%) |
| (Missing) | 74 (1.3%) |
| **Race/Ethnicity** |  |
| Black | 108 (1.9%) |
| Indigenous | 594 (10%) |
| Mestizo | 2,762 (48%) |
| Mulatto | 63 (1.1%) |
| Other | 339 (5.9%) |
| White | 1,116 (19%) |
| (Missing) | 794 (14%) |
| ^1^n (%) | |

***Table S12b. Childhood predictors regression analysis results for Mexico***

|  | | Charitable giving | | | | Helping | | | |
| --- | --- | --- | --- | --- | --- | --- | --- | --- | --- |
| Variable | Category | Risk-Ratio | RR 95% CI | log(RR) SE | Global p-value | Risk-Ratio | RR 95% CI | log(RR) SE | Global p-value |
| Relationship with mother | (Ref: Very bad/somewhat bad) |  |  |  | 0.707 |  |  |  | 0.831 |
|  | Very good/somewhat good | 1.06 | (0.78,1.42) | 0.15 |  | 1.00 | (0.89,1.12) | 0.06 |  |
| Relationship with father | (Ref: Very bad/somewhat bad) |  |  |  | 0.487 |  |  |  | 0.407 |
|  | Very good/somewhat good | 0.92 | (0.74,1.16) | 0.11 |  | 0.97 | (0.89,1.05) | 0.04 |  |
| Parent marital status | (Ref: Parents married) |  |  |  | 0.596 |  |  |  | 0.042 |
|  | Divorced | 0.95 | (0.70,1.27) | 0.15 |  | 1.13 | (1.02,1.24) | 0.05 |  |
|  | Parents were never married | 0.88 | (0.71,1.09) | 0.11 |  | 1.00 | (0.92,1.08) | 0.04 |  |
|  | One or both parents had died | 1.02 | (0.70,1.48) | 0.19 |  | 1.02 | (0.88,1.19) | 0.08 |  |
| Subjective financial status of family growing up | (Ref: Got by) |  |  |  | 0.019 |  |  |  | 0.474 |
|  | Lived comfortably | 1.11 | (0.93,1.32) | 0.09 |  | 1.00 | (0.93,1.06) | 0.03 |  |
|  | Found it difficult | 0.96 | (0.80,1.14) | 0.09 |  | 0.96 | (0.90,1.02) | 0.03 |  |
|  | Found it very difficult | 1.37 | (1.06,1.77) | 0.13 |  | 1.03 | (0.92,1.15) | 0.06 |  |
| Abuse | (Ref: No) |  |  |  | 0.556 |  |  |  | 0.003 |
|  | Yes | 1.05 | (0.87,1.27) | 0.10 |  | 1.10 | (1.03,1.18) | 0.03 |  |
| Outsider growing up | (Ref: No) |  |  |  | 0.703 |  |  |  | 0.004 |
|  | Yes | 1.03 | (0.86,1.25) | 0.10 |  | 1.10 | (1.03,1.19) | 0.04 |  |
| Self-rated health growing up | (Ref: Good) |  |  |  | 0.633 |  |  |  | 0.004 |
|  | Excellent | 1.10 | (0.92,1.30) | 0.09 |  | 1.10 | (1.02,1.18) | 0.04 |  |
|  | Very good | 1.02 | (0.84,1.24) | 0.10 |  | 1.07 | (0.99,1.16) | 0.04 |  |
|  | Fair | 1.16 | (0.93,1.45) | 0.11 |  | 1.01 | (0.91,1.11) | 0.05 |  |
|  | Poor | 1.03 | (0.68,1.55) | 0.21 |  | 1.26 | (1.10,1.44) | 0.07 |  |
| Immigration status | (Ref: Born in this country) |  |  |  | 0.745 |  |  |  | 0.843 |
|  | Born in another country | 0.92 | (0.55,1.55) | 0.26 |  | 1.00 | (0.82,1.22) | 0.10 |  |
| Age 12 religious service attendance | (Ref: Never) |  |  |  | <.001 |  |  |  | <.001 |
|  | At least 1/week | 1.54 | (1.24,1.91) | 0.11 |  | 1.18 | (1.09,1.29) | 0.04 |  |
|  | 1-3/month | 1.41 | (1.11,1.79) | 0.12 |  | 1.11 | (1.01,1.22) | 0.05 |  |
|  | < 1/month | 1.16 | (0.90,1.51) | 0.13 |  | 1.11 | (1.01,1.21) | 0.05 |  |
| Year of birth | (Ref: 1998-2005; current age: 18-24) |  |  |  | 0.054 |  |  |  | 0.476 |
|  | 1993-1998; age 25-29 | 1.01 | (0.79,1.30) | 0.12 |  | 1.03 | (0.94,1.12) | 0.05 |  |
|  | 1983-1993; age 30-39 | 1.03 | (0.83,1.29) | 0.11 |  | 0.98 | (0.90,1.06) | 0.04 |  |
|  | 1973-1983; age 40-49 | 1.06 | (0.84,1.33) | 0.12 |  | 0.99 | (0.91,1.08) | 0.04 |  |
|  | 1963-1973; age 50-59 | 1.15 | (0.90,1.46) | 0.12 |  | 1.01 | (0.92,1.11) | 0.05 |  |
|  | 1953-1963; age 60-69 | 1.29 | (0.99,1.69) | 0.14 |  | 0.94 | (0.83,1.05) | 0.06 |  |
|  | 1943-1953; age 70-79 | 1.32 | (0.92,1.89) | 0.18 |  | 0.85 | (0.71,1.01) | 0.09 |  |
|  | 1943 or earlier; age 80+ | 1.86 | (1.20,2.90) | 0.23 |  | 1.01 | (0.76,1.34) | 0.15 |  |
| Gender | (Ref: Male) |  |  |  | 0.058 |  |  |  | <.001 |
|  | Female | 0.86 | (0.75,0.98) | 0.07 |  | 0.89 | (0.84,0.94) | 0.03 |  |
|  | Other | 0.46 | (0.09,2.25) | 0.81 |  | 1.28 | (0.80,2.03) | 0.24 |  |
| Religious affiliation | (Ref: No religion/Atheist/Agnostic) |  |  |  | <.001 |  |  |  | 0.451 |
|  | Christianity | 1.22 | (0.87,1.70) | 0.17 |  | 1.02 | (0.91,1.14) | 0.06 |  |
|  | Collapsed affiliations with prevalence<3% | 2.48 | (1.52,4.06) | 0.25 |  | 0.83 | (0.59,1.17) | 0.17 |  |
| Race/ethnicity | (Ref: Plurality group) |  |  |  | 0.354 |  |  |  | 0.725 |
|  | Non-plurality groups | 1.06 | (0.93,1.21) | 0.07 |  | 1.00 | (0.94,1.06) | 0.03 |  |

***Table S12c. Sensitivity to unmeasured confounding of childhood predictors in Mexico***

|  | | Charitable giving | | Helping | |
| --- | --- | --- | --- | --- | --- |
| Variable | Category | E-value for Estimate | E-value for 95% CI | E-value for Estimate | E-value for 95% CI |
| Relationship with mother | (Ref: Very bad/somewhat bad) |  |  |  |  |
|  | Very good/somewhat good | 1.30 | 1.00 | 1.02 | 1.00 |
| Relationship with father | (Ref: Very bad/somewhat bad) |  |  |  |  |
|  | Very good/somewhat good | 1.38 | 1.00 | 1.22 | 1.00 |
| Parent marital status | (Ref: Parents married) |  |  |  |  |
|  | Divorced | 1.30 | 1.00 | 1.50 | 1.18 |
|  | Parents were never married | 1.54 | 1.00 | 1.07 | 1.00 |
|  | One or both parents had died | 1.15 | 1.00 | 1.16 | 1.00 |
| Subjective financial status of family growing up | (Ref: Got by) |  |  |  |  |
|  | Lived comfortably | 1.46 | 1.00 | 1.06 | 1.00 |
|  | Found it difficult | 1.26 | 1.00 | 1.26 | 1.00 |
|  | Found it very difficult | 2.08 | 1.30 | 1.19 | 1.00 |
| Abuse | (Ref: No) |  |  |  |  |
|  | Yes | 1.29 | 1.00 | 1.45 | 1.22 |
| Outsider growing up | (Ref: No) |  |  |  |  |
|  | Yes | 1.23 | 1.00 | 1.44 | 1.20 |
| Self-rated health growing up | (Ref: Good) |  |  |  |  |
|  | Excellent | 1.42 | 1.00 | 1.42 | 1.17 |
|  | Very good | 1.16 | 1.00 | 1.35 | 1.00 |
|  | Fair | 1.60 | 1.00 | 1.08 | 1.00 |
|  | Poor | 1.21 | 1.00 | 1.83 | 1.42 |
| Immigration status | (Ref: Born in this country) |  |  |  |  |
|  | Born in another country | 1.38 | 1.00 | 1.05 | 1.00 |
| Age 12 religious service attendance | (Ref: Never) |  |  |  |  |
|  | At least 1/week | 2.44 | 1.78 | 1.65 | 1.40 |
|  | 1-3/month | 2.18 | 1.47 | 1.45 | 1.09 |
|  | < 1/month | 1.60 | 1.00 | 1.45 | 1.10 |
| Year of birth | (Ref: 1998-2005; current age: 18-24) |  |  |  |  |
|  | 1993-1998; age 25-29 | 1.13 | 1.00 | 1.20 | 1.00 |
|  | 1983-1993; age 30-39 | 1.22 | 1.00 | 1.18 | 1.00 |
|  | 1973-1983; age 40-49 | 1.30 | 1.00 | 1.11 | 1.00 |
|  | 1963-1973; age 50-59 | 1.55 | 1.00 | 1.11 | 1.00 |
|  | 1953-1963; age 60-69 | 1.91 | 1.00 | 1.34 | 1.00 |
|  | 1943-1953; age 70-79 | 1.97 | 1.00 | 1.64 | 1.00 |
|  | 1943 or earlier; age 80+ | 3.13 | 1.68 | 1.09 | 1.00 |
| Gender | (Ref: Male) |  |  |  |  |
|  | Female | 1.60 | 1.16 | 1.50 | 1.33 |
|  | Other | 3.78 | 1.00 | 1.87 | 1.00 |
| Religious affiliation | (Ref: No religion/Atheist/Agnostic) |  |  |  |  |
|  | Christianity | 1.73 | 1.00 | 1.16 | 1.00 |
|  | Collapsed affiliations with prevalence<3% | 4.40 | 2.41 | 1.69 | 1.00 |
| Race/ethnicity | (Ref: Plurality group) |  |  |  |  |
|  | Non-plurality groups | 1.32 | 1.00 | 1.01 | 1.00 |

***Table S13a. Nationally representative descriptive statistics for Nigeria***

| **Characteristic** | **N = 6,827**^1^ |
| --- | --- |
| **Relationship with mother** |  |
| Very good | 5,986 (88%) |
| Somewhat good | 648 (9.5%) |
| Somewhat bad | 62 (0.9%) |
| Very bad | 18 (0.3%) |
| Does not apply | 104 (1.5%) |
| (Missing) | 9 (0.1%) |
| **Relationship with father** |  |
| Very good | 5,578 (82%) |
| Somewhat good | 924 (14%) |
| Somewhat bad | 76 (1.1%) |
| Very bad | 43 (0.6%) |
| Does not apply | 177 (2.6%) |
| (Missing) | 29 (0.4%) |
| **Parent marital status** |  |
| Parents married | 5,568 (82%) |
| Divorced | 307 (4.5%) |
| Parents were never married | 335 (4.9%) |
| One or both parents had died | 462 (6.8%) |
| (Missing) | 154 (2.3%) |
| **Subjective financial status of family growing up** |  |
| Lived comfortably | 2,192 (32%) |
| Got by | 2,381 (35%) |
| Found it difficult | 1,661 (24%) |
| Found it very difficult | 563 (8.3%) |
| (Missing) | 29 (0.4%) |
| **Abuse** |  |
| Yes | 880 (13%) |
| No | 5,851 (86%) |
| (Missing) | 96 (1.4%) |
| **Outsider growing up** |  |
| Yes | 669 (9.8%) |
| No | 6,059 (89%) |
| (Missing) | 99 (1.5%) |
| **Self-rated health growing up** |  |
| Excellent | 2,644 (39%) |
| Very good | 2,613 (38%) |
| Good | 1,152 (17%) |
| Fair | 306 (4.5%) |
| Poor | 98 (1.4%) |
| (Missing) | 14 (0.2%) |
| **Immigration status** |  |
| Born in this country | 6,779 (99%) |
| Born in another country | 47 (0.7%) |
| (Missing) | 1 (<0.1%) |
| **Age 12 religious service attendance** |  |
| At least 1/week | 5,907 (87%) |
| 1-3/month | 600 (8.8%) |
| <1/month | 136 (2.0%) |
| Never | 138 (2.0%) |
| (Missing) | 45 (0.7%) |
| **Year of birth** |  |
| 1998-2005; age 18-24 | 1,533 (22%) |
| 1993-1998; age 25-29 | 1,193 (17%) |
| 1983-1993; age 30-39 | 1,943 (28%) |
| 1973-1983; age 40-49 | 1,059 (16%) |
| 1963-1973; age 50-59 | 619 (9.1%) |
| 1953-1963; age 60-69 | 296 (4.3%) |
| 1943-1953; age 70-79 | 133 (2.0%) |
| 1943 or earlier; age 80+ | 50 (0.7%) |
| (Missing) | 0 (0%) |
| **Gender** |  |
| Male | 3,371 (49%) |
| Female | 3,456 (51%) |
| Other | 0 (<0.1%) |
| (Missing) | 0 (0%) |
| **Religious affiliation** |  |
| Christianity | 3,463 (51%) |
| Islam | 3,314 (49%) |
| Hinduism | 0 (0%) |
| Buddhism | 0 (<0.1%) |
| Judaism | 0 (0%) |
| Sikhism | 0 (0%) |
| Baha'i | 0 (0%) |
| Jainism | 0 (0%) |
| Shinto | 0 (0%) |
| Taoism | 0 (0%) |
| Confucianism | 0 (<0.1%) |
| Primal, Animist, or Folk religion | 17 (0.3%) |
| Spiritism | 0 (0%) |
| Umbanda, Candomble, and other African-derived religions | 0 (0%) |
| Chinese folk/traditional religion | 0 (0%) |
| Some other religion | 0 (0%) |
| No religion/Atheist/Agnostic | 19 (0.3%) |
| (Missing) | 14 (0.2%) |
| **Race/Ethnicity** |  |
| Edo | 116 (1.7%) |
| Efik | 48 (0.7%) |
| Fulani | 266 (3.9%) |
| Hausa | 2,342 (34%) |
| Ibibio | 180 (2.6%) |
| Idoma | 61 (0.9%) |
| Igala | 77 (1.1%) |
| Igbo (Ibo) | 1,111 (16%) |
| Ijaw | 110 (1.6%) |
| Kanuri | 31 (0.5%) |
| Other | 1,014 (15%) |
| Tiv | 198 (2.9%) |
| Urhobo | 38 (0.6%) |
| Yoruba | 1,230 (18%) |
| (Missing) | 4 (<0.1%) |
| ^1^n (%) | |

***Table S13b. Childhood predictors regression analysis results for Nigeria***

|  | | Charitable giving | | | | Helping | | | |
| --- | --- | --- | --- | --- | --- | --- | --- | --- | --- |
| Variable | Category | Risk-Ratio | RR 95% CI | log(RR) SE | Global p-value | Risk-Ratio | RR 95% CI | log(RR) SE | Global p-value |
| Relationship with mother | (Ref: Very bad/somewhat bad) |  |  |  | 0.347 |  |  |  | 0.068 |
|  | Very good/somewhat good | 1.16 | (0.85,1.57) | 0.15 |  | 1.13 | (0.99,1.29) | 0.07 |  |
| Relationship with father | (Ref: Very bad/somewhat bad) |  |  |  | 0.041 |  |  |  | 0.011 |
|  | Very good/somewhat good | 1.29 | (1.01,1.65) | 0.12 |  | 1.16 | (1.04,1.30) | 0.06 |  |
| Parent marital status | (Ref: Parents married) |  |  |  | 0.084 |  |  |  | 0.343 |
|  | Divorced | 1.00 | (0.84,1.20) | 0.09 |  | 0.98 | (0.90,1.07) | 0.04 |  |
|  | Parents were never married | 1.07 | (0.92,1.24) | 0.08 |  | 1.04 | (0.98,1.10) | 0.03 |  |
|  | One or both parents had died | 1.16 | (1.03,1.31) | 0.06 |  | 1.03 | (0.98,1.08) | 0.02 |  |
| Subjective financial status of family growing up | (Ref: Got by) |  |  |  | <.001 |  |  |  | 0.012 |
|  | Lived comfortably | 1.24 | (1.13,1.36) | 0.05 |  | 1.08 | (1.03,1.13) | 0.02 |  |
|  | Found it difficult | 1.09 | (0.98,1.21) | 0.05 |  | 1.04 | (0.99,1.09) | 0.03 |  |
|  | Found it very difficult | 0.98 | (0.81,1.19) | 0.10 |  | 1.06 | (1.00,1.12) | 0.03 |  |
| Abuse | (Ref: No) |  |  |  | 0.606 |  |  |  | 0.152 |
|  | Yes | 1.03 | (0.92,1.15) | 0.06 |  | 1.03 | (0.99,1.07) | 0.02 |  |
| Outsider growing up | (Ref: No) |  |  |  | 0.219 |  |  |  | 0.250 |
|  | Yes | 1.08 | (0.95,1.22) | 0.06 |  | 1.03 | (0.98,1.08) | 0.03 |  |
| Self-rated health growing up | (Ref: Good) |  |  |  | 0.127 |  |  |  | 0.074 |
|  | Excellent | 1.16 | (1.04,1.31) | 0.06 |  | 1.05 | (0.99,1.12) | 0.03 |  |
|  | Very good | 1.11 | (0.99,1.25) | 0.06 |  | 1.07 | (1.01,1.13) | 0.03 |  |
|  | Fair | 1.17 | (0.95,1.44) | 0.11 |  | 1.12 | (1.03,1.22) | 0.04 |  |
|  | Poor | 1.17 | (0.84,1.64) | 0.17 |  | 0.98 | (0.84,1.14) | 0.08 |  |
| Immigration status | (Ref: Born in this country) |  |  |  | 0.649 |  |  |  | 0.381 |
|  | Born in another country | 0.90 | (0.58,1.40) | 0.22 |  | 0.88 | (0.67,1.16) | 0.14 |  |
| Age 12 religious service attendance | (Ref: Never) |  |  |  | 0.001 |  |  |  | 0.039 |
|  | At least 1/week | 0.82 | (0.62,1.09) | 0.14 |  | 1.09 | (0.93,1.29) | 0.08 |  |
|  | 1-3/month | 0.69 | (0.50,0.95) | 0.17 |  | 1.00 | (0.84,1.19) | 0.09 |  |
|  | < 1/month | 0.47 | (0.30,0.73) | 0.22 |  | 0.99 | (0.79,1.26) | 0.12 |  |
| Year of birth | (Ref: 1998-2005; current age: 18-24) |  |  |  | <.001 |  |  |  | <.001 |
|  | 1993-1998; age 25-29 | 1.17 | (1.04,1.31) | 0.06 |  | 1.13 | (1.08,1.19) | 0.02 |  |
|  | 1983-1993; age 30-39 | 1.24 | (1.12,1.38) | 0.05 |  | 1.13 | (1.08,1.19) | 0.02 |  |
|  | 1973-1983; age 40-49 | 1.37 | (1.21,1.56) | 0.06 |  | 1.13 | (1.07,1.19) | 0.03 |  |
|  | 1963-1973; age 50-59 | 1.46 | (1.26,1.69) | 0.08 |  | 1.19 | (1.11,1.27) | 0.04 |  |
|  | 1953-1963; age 60-69 | 1.07 | (0.83,1.38) | 0.13 |  | 1.16 | (1.04,1.30) | 0.06 |  |
|  | 1943-1953; age 70-79 | 1.53 | (1.15,2.04) | 0.15 |  | 1.12 | (0.98,1.28) | 0.07 |  |
|  | 1943 or earlier; age 80+ | 1.28 | (0.83,1.98) | 0.22 |  | 0.97 | (0.71,1.31) | 0.16 |  |
| Gender | (Ref: Male) |  |  |  | <.001 |  |  |  | <.001 |
|  | Female | 0.85 | (0.78,0.92) | 0.04 |  | 0.97 | (0.94,1.01) | 0.02 |  |
|  | Other | 2.19 | (1.89,2.53) | 0.07 |  | 1.17 | (1.11,1.24) | 0.03 |  |
| Religious affiliation | (Ref: Christianity) |  |  |  | 0.033 |  |  |  | 0.044 |
|  | Islam | 1.12 | (0.98,1.28) | 0.07 |  | 1.06 | (1.01,1.11) | 0.02 |  |
|  | Collapsed affiliations with prevalence<3% | 1.49 | (1.03,2.16) | 0.19 |  | 1.13 | (0.91,1.41) | 0.11 |  |
| Race/ethnicity | (Ref: Plurality group) |  |  |  | 0.537 |  |  |  | 0.374 |
|  | Non-plurality groups | 0.96 | (0.85,1.09) | 0.06 |  | 1.02 | (0.97,1.08) | 0.03 |  |

***Table S13c. Sensitivity to unmeasured confounding of childhood predictors in Nigeria***

|  | | Charitable giving | | Helping | |
| --- | --- | --- | --- | --- | --- |
| Variable | Category | E-value for Estimate | E-value for 95% CI | E-value for Estimate | E-value for 95% CI |
| Relationship with mother | (Ref: Very bad/somewhat bad) |  |  |  |  |
|  | Very good/somewhat good | 1.58 | 1.00 | 1.51 | 1.00 |
| Relationship with father | (Ref: Very bad/somewhat bad) |  |  |  |  |
|  | Very good/somewhat good | 1.90 | 1.10 | 1.60 | 1.23 |
| Parent marital status | (Ref: Parents married) |  |  |  |  |
|  | Divorced | 1.06 | 1.00 | 1.16 | 1.00 |
|  | Parents were never married | 1.34 | 1.00 | 1.24 | 1.00 |
|  | One or both parents had died | 1.60 | 1.22 | 1.19 | 1.00 |
| Subjective financial status of family growing up | (Ref: Got by) |  |  |  |  |
|  | Lived comfortably | 1.79 | 1.52 | 1.36 | 1.21 |
|  | Found it difficult | 1.39 | 1.00 | 1.24 | 1.00 |
|  | Found it very difficult | 1.15 | 1.00 | 1.30 | 1.00 |
| Abuse | (Ref: No) |  |  |  |  |
|  | Yes | 1.21 | 1.00 | 1.20 | 1.00 |
| Outsider growing up | (Ref: No) |  |  |  |  |
|  | Yes | 1.37 | 1.00 | 1.20 | 1.00 |
| Self-rated health growing up | (Ref: Good) |  |  |  |  |
|  | Excellent | 1.60 | 1.23 | 1.29 | 1.00 |
|  | Very good | 1.47 | 1.00 | 1.34 | 1.12 |
|  | Fair | 1.62 | 1.00 | 1.48 | 1.19 |
|  | Poor | 1.62 | 1.00 | 1.17 | 1.00 |
| Immigration status | (Ref: Born in this country) |  |  |  |  |
|  | Born in another country | 1.45 | 1.00 | 1.51 | 1.00 |
| Age 12 religious service attendance | (Ref: Never) |  |  |  |  |
|  | At least 1/week | 1.73 | 1.00 | 1.42 | 1.00 |
|  | 1-3/month | 2.27 | 1.29 | 1.01 | 1.00 |
|  | < 1/month | 3.66 | 2.09 | 1.09 | 1.00 |
| Year of birth | (Ref: 1998-2005; current age: 18-24) |  |  |  |  |
|  | 1993-1998; age 25-29 | 1.61 | 1.25 | 1.52 | 1.37 |
|  | 1983-1993; age 30-39 | 1.79 | 1.49 | 1.52 | 1.37 |
|  | 1973-1983; age 40-49 | 2.09 | 1.72 | 1.51 | 1.34 |
|  | 1963-1973; age 50-59 | 2.28 | 1.83 | 1.66 | 1.45 |
|  | 1953-1963; age 60-69 | 1.34 | 1.00 | 1.59 | 1.25 |
|  | 1943-1953; age 70-79 | 2.44 | 1.56 | 1.48 | 1.00 |
|  | 1943 or earlier; age 80+ | 1.89 | 1.00 | 1.22 | 1.00 |
| Gender | (Ref: Male) |  |  |  |  |
|  | Female | 1.64 | 1.39 | 1.20 | 1.00 |
|  | Other | 3.80 | 3.19 | 1.62 | 1.45 |
| Religious affiliation | (Ref: Christianity) |  |  |  |  |
|  | Islam | 1.49 | 1.00 | 1.30 | 1.08 |
|  | Collapsed affiliations with prevalence<3% | 2.34 | 1.20 | 1.52 | 1.00 |
| Race/ethnicity | (Ref: Plurality group) |  |  |  |  |
|  | Non-plurality groups | 1.24 | 1.00 | 1.17 | 1.00 |

***Table S14a. Nationally representative descriptive statistics for Philippines***

| **Characteristic** | **N = 5,292**^1^ |
| --- | --- |
| **Relationship with mother** |  |
| Very good | 3,333 (63%) |
| Somewhat good | 1,703 (32%) |
| Somewhat bad | 124 (2.3%) |
| Very bad | 39 (0.7%) |
| Does not apply | 59 (1.1%) |
| (Missing) | 35 (0.7%) |
| **Relationship with father** |  |
| Very good | 3,443 (65%) |
| Somewhat good | 1,429 (27%) |
| Somewhat bad | 159 (3.0%) |
| Very bad | 58 (1.1%) |
| Does not apply | 108 (2.0%) |
| (Missing) | 95 (1.8%) |
| **Parent marital status** |  |
| Parents married | 4,575 (86%) |
| Divorced | 64 (1.2%) |
| Parents were never married | 517 (9.8%) |
| One or both parents had died | 51 (1.0%) |
| (Missing) | 85 (1.6%) |
| **Subjective financial status of family growing up** |  |
| Lived comfortably | 937 (18%) |
| Got by | 3,006 (57%) |
| Found it difficult | 1,055 (20%) |
| Found it very difficult | 291 (5.5%) |
| (Missing) | 3 (<0.1%) |
| **Abuse** |  |
| Yes | 420 (7.9%) |
| No | 4,837 (91%) |
| (Missing) | 35 (0.7%) |
| **Outsider growing up** |  |
| Yes | 395 (7.5%) |
| No | 4,884 (92%) |
| (Missing) | 13 (0.2%) |
| **Self-rated health growing up** |  |
| Excellent | 1,041 (20%) |
| Very good | 559 (11%) |
| Good | 2,174 (41%) |
| Fair | 1,246 (24%) |
| Poor | 272 (5.1%) |
| (Missing) | 0 (<0.1%) |
| **Immigration status** |  |
| Born in this country | 5,284 (100%) |
| Born in another country | 8 (0.1%) |
| (Missing) | 0 (0%) |
| **Age 12 religious service attendance** |  |
| At least 1/week | 2,453 (46%) |
| 1-3/month | 1,699 (32%) |
| <1/month | 892 (17%) |
| Never | 201 (3.8%) |
| (Missing) | 47 (0.9%) |
| **Year of birth** |  |
| 1998-2005; age 18-24 | 1,073 (20%) |
| 1993-1998; age 25-29 | 695 (13%) |
| 1983-1993; age 30-39 | 1,160 (22%) |
| 1973-1983; age 40-49 | 972 (18%) |
| 1963-1973; age 50-59 | 732 (14%) |
| 1953-1963; age 60-69 | 495 (9.4%) |
| 1943-1953; age 70-79 | 143 (2.7%) |
| 1943 or earlier; age 80+ | 23 (0.4%) |
| (Missing) | 0 (0%) |
| **Gender** |  |
| Male | 2,625 (50%) |
| Female | 2,643 (50%) |
| Other | 13 (0.2%) |
| (Missing) | 11 (0.2%) |
| **Religious affiliation** |  |
| Christianity | 4,968 (94%) |
| Islam | 276 (5.2%) |
| Hinduism | 0 (0%) |
| Buddhism | 1 (<0.1%) |
| Judaism | 0 (0%) |
| Sikhism | 4 (<0.1%) |
| Baha'i | 1 (<0.1%) |
| Jainism | 0 (0%) |
| Shinto | 0 (0%) |
| Taoism | 0 (0%) |
| Confucianism | 0 (0%) |
| Primal, Animist, or Folk religion | 14 (0.3%) |
| Spiritism | 0 (0%) |
| Umbanda, Candomble, and other African-derived religions | 0 (0%) |
| Chinese folk/traditional religion | 0 (0%) |
| Some other religion | 9 (0.2%) |
| No religion/Atheist/Agnostic | 9 (0.2%) |
| (Missing) | 11 (0.2%) |
| **Race/Ethnicity** |  |
| Aeta | 1 (<0.1%) |
| Badjao | 2 (<0.1%) |
| Bicolano/Bikolano | 300 (5.7%) |
| Cebuano | 656 (12%) |
| Chinese-Filipino | 3 (<0.1%) |
| Igorot | 42 (0.8%) |
| Ilocano/Ilokano | 429 (8.1%) |
| Ilonggo/Hiligaynon | 428 (8.1%) |
| Kapampangan | 107 (2.0%) |
| Maguindanaoan | 84 (1.6%) |
| Mangyan | 2 (<0.1%) |
| Maranao | 39 (0.7%) |
| Masbateno | 54 (1.0%) |
| Other | 244 (4.6%) |
| Pangasinense | 107 (2.0%) |
| Tagalog | 1,691 (32%) |
| Tausug | 94 (1.8%) |
| Visayan/Bisaya | 739 (14%) |
| Waray | 216 (4.1%) |
| Zamboangueno | 51 (1.0%) |
| (Missing) | 3 (<0.1%) |
| ^1^n (%) | |

***Table S14b. Childhood predictors regression analysis results for Philippines***

|  | | Charitable giving | | | | Helping | | | |
| --- | --- | --- | --- | --- | --- | --- | --- | --- | --- |
| Variable | Category | Risk-Ratio | RR 95% CI | log(RR) SE | Global p-value | Risk-Ratio | RR 95% CI | log(RR) SE | Global p-value |
| Relationship with mother | (Ref: Very bad/somewhat bad) |  |  |  | 0.404 |  |  |  | 0.763 |
|  | Very good/somewhat good | 1.22 | (0.76,1.96) | 0.24 |  | 1.02 | (0.89,1.16) | 0.07 |  |
| Relationship with father | (Ref: Very bad/somewhat bad) |  |  |  | 0.781 |  |  |  | 0.452 |
|  | Very good/somewhat good | 0.99 | (0.65,1.50) | 0.21 |  | 0.96 | (0.86,1.07) | 0.06 |  |
| Parent marital status | (Ref: Parents married) |  |  |  | 0.815 |  |  |  | 0.639 |
|  | Divorced | 0.87 | (0.39,1.96) | 0.41 |  | 0.93 | (0.75,1.16) | 0.11 |  |
|  | Parents were never married | 1.08 | (0.83,1.40) | 0.13 |  | 1.03 | (0.95,1.12) | 0.04 |  |
|  | One or both parents had died | 1.23 | (0.67,2.28) | 0.31 |  | 1.09 | (0.86,1.38) | 0.12 |  |
| Subjective financial status of family growing up | (Ref: Got by) |  |  |  | 0.021 |  |  |  | 0.172 |
|  | Lived comfortably | 1.39 | (1.13,1.73) | 0.11 |  | 1.08 | (1.01,1.15) | 0.03 |  |
|  | Found it difficult | 1.09 | (0.89,1.35) | 0.11 |  | 1.03 | (0.96,1.11) | 0.04 |  |
|  | Found it very difficult | 1.21 | (0.89,1.65) | 0.16 |  | 1.00 | (0.88,1.13) | 0.07 |  |
| Abuse | (Ref: No) |  |  |  | 0.833 |  |  |  | 0.464 |
|  | Yes | 1.03 | (0.76,1.40) | 0.15 |  | 1.04 | (0.94,1.14) | 0.05 |  |
| Outsider growing up | (Ref: No) |  |  |  | 0.022 |  |  |  | 0.681 |
|  | Yes | 1.38 | (1.05,1.82) | 0.14 |  | 1.02 | (0.92,1.13) | 0.05 |  |
| Self-rated health growing up | (Ref: Good) |  |  |  | 0.584 |  |  |  | 0.103 |
|  | Excellent | 1.10 | (0.88,1.39) | 0.12 |  | 1.10 | (1.02,1.17) | 0.04 |  |
|  | Very good | 1.00 | (0.75,1.33) | 0.15 |  | 1.02 | (0.93,1.11) | 0.05 |  |
|  | Fair | 0.93 | (0.75,1.15) | 0.11 |  | 1.00 | (0.94,1.07) | 0.03 |  |
|  | Poor | 0.76 | (0.49,1.16) | 0.22 |  | 1.00 | (0.88,1.13) | 0.06 |  |
| Immigration status | (Ref: Born in this country) |  |  |  | <.001 |  |  |  | 0.138 |
|  | Born in another country | 0.00 | (0.00,0.00) | 0.45 |  | 0.40 | (0.12,1.35) | 0.63 |  |
| Age 12 religious service attendance | (Ref: Never) |  |  |  | 0.013 |  |  |  | <.001 |
|  | At least 1/week | 1.48 | (0.88,2.51) | 0.27 |  | 1.24 | (1.04,1.47) | 0.09 |  |
|  | 1-3/month | 1.34 | (0.80,2.23) | 0.26 |  | 1.22 | (1.03,1.44) | 0.09 |  |
|  | < 1/month | 0.95 | (0.54,1.70) | 0.29 |  | 1.02 | (0.85,1.22) | 0.09 |  |
| Year of birth | (Ref: 1998-2005; current age: 18-24) |  |  |  | <.001 |  |  |  | 0.008 |
|  | 1993-1998; age 25-29 | 2.13 | (1.48,3.08) | 0.19 |  | 0.95 | (0.87,1.05) | 0.05 |  |
|  | 1983-1993; age 30-39 | 2.06 | (1.49,2.84) | 0.16 |  | 1.02 | (0.95,1.10) | 0.04 |  |
|  | 1973-1983; age 40-49 | 2.12 | (1.52,2.94) | 0.17 |  | 1.00 | (0.93,1.07) | 0.04 |  |
|  | 1963-1973; age 50-59 | 1.84 | (1.27,2.67) | 0.19 |  | 0.92 | (0.84,1.01) | 0.05 |  |
|  | 1953-1963; age 60-69 | 2.21 | (1.46,3.36) | 0.21 |  | 0.89 | (0.79,0.99) | 0.06 |  |
|  | 1943-1953; age 70-79 | 2.98 | (1.91,4.65) | 0.23 |  | 0.71 | (0.55,0.92) | 0.13 |  |
|  | 1943 or earlier; age 80+ | 2.20 | (0.72,6.71) | 0.57 |  | 0.85 | (0.55,1.32) | 0.22 |  |
| Gender | (Ref: Male) |  |  |  | 0.004 |  |  |  | 0.002 |
|  | Female | 0.80 | (0.69,0.94) | 0.08 |  | 0.93 | (0.89,0.98) | 0.03 |  |
|  | Other | 1.97 | (0.90,4.30) | 0.40 |  | 1.21 | (0.97,1.52) | 0.12 |  |
| Religious affiliation | (Ref: Christianity) |  |  |  | 0.778 |  |  |  | 0.384 |
|  | Islam | 1.02 | (0.75,1.39) | 0.16 |  | 1.05 | (0.97,1.15) | 0.04 |  |
|  | Collapsed affiliations with prevalence<3% | 0.78 | (0.38,1.60) | 0.37 |  | 1.12 | (0.85,1.48) | 0.14 |  |
| Race/ethnicity | (Ref: Plurality group) |  |  |  | 0.082 |  |  |  | 0.396 |
|  | Non-plurality groups | 0.85 | (0.70,1.03) | 0.10 |  | 0.98 | (0.93,1.03) | 0.03 |  |

***Table S14c. Sensitivity to unmeasured confounding of childhood predictors in Philippines***

|  | | Charitable giving | | Helping | |
| --- | --- | --- | --- | --- | --- |
| Variable | Category | E-value for Estimate | E-value for 95% CI | E-value for Estimate | E-value for 95% CI |
| Relationship with mother | (Ref: Very bad/somewhat bad) |  |  |  |  |
|  | Very good/somewhat good | 1.74 | 1.00 | 1.15 | 1.00 |
| Relationship with father | (Ref: Very bad/somewhat bad) |  |  |  |  |
|  | Very good/somewhat good | 1.13 | 1.00 | 1.25 | 1.00 |
| Parent marital status | (Ref: Parents married) |  |  |  |  |
|  | Divorced | 1.56 | 1.00 | 1.36 | 1.00 |
|  | Parents were never married | 1.37 | 1.00 | 1.22 | 1.00 |
|  | One or both parents had died | 1.77 | 1.00 | 1.40 | 1.00 |
| Subjective financial status of family growing up | (Ref: Got by) |  |  |  |  |
|  | Lived comfortably | 2.13 | 1.50 | 1.37 | 1.09 |
|  | Found it difficult | 1.41 | 1.00 | 1.21 | 1.00 |
|  | Found it very difficult | 1.71 | 1.00 | 1.06 | 1.00 |
| Abuse | (Ref: No) |  |  |  |  |
|  | Yes | 1.21 | 1.00 | 1.23 | 1.00 |
| Outsider growing up | (Ref: No) |  |  |  |  |
|  | Yes | 2.10 | 1.26 | 1.17 | 1.00 |
| Self-rated health growing up | (Ref: Good) |  |  |  |  |
|  | Excellent | 1.44 | 1.00 | 1.42 | 1.17 |
|  | Very good | 1.06 | 1.00 | 1.15 | 1.00 |
|  | Fair | 1.36 | 1.00 | 1.03 | 1.00 |
|  | Poor | 1.98 | 1.00 | 1.04 | 1.00 |
| Immigration status | (Ref: Born in this country) |  |  |  |  |
|  | Born in another country | 452573.84 | 188900.53 | 4.49 | 1.00 |
| Age 12 religious service attendance | (Ref: Never) |  |  |  |  |
|  | At least 1/week | 2.33 | 1.00 | 1.78 | 1.24 |
|  | 1-3/month | 2.01 | 1.00 | 1.73 | 1.19 |
|  | < 1/month | 1.27 | 1.00 | 1.16 | 1.00 |
| Year of birth | (Ref: 1998-2005; current age: 18-24) |  |  |  |  |
|  | 1993-1998; age 25-29 | 3.69 | 2.32 | 1.27 | 1.00 |
|  | 1983-1993; age 30-39 | 3.53 | 2.35 | 1.17 | 1.00 |
|  | 1973-1983; age 40-49 | 3.66 | 2.42 | 1.04 | 1.00 |
|  | 1963-1973; age 50-59 | 3.09 | 1.86 | 1.40 | 1.00 |
|  | 1953-1963; age 60-69 | 3.86 | 2.28 | 1.51 | 1.12 |
|  | 1943-1953; age 70-79 | 5.41 | 3.23 | 2.16 | 1.41 |
|  | 1943 or earlier; age 80+ | 3.83 | 1.00 | 1.62 | 1.00 |
| Gender | (Ref: Male) |  |  |  |  |
|  | Female | 1.80 | 1.33 | 1.35 | 1.17 |
|  | Other | 3.35 | 1.00 | 1.72 | 1.00 |
| Religious affiliation | (Ref: Christianity) |  |  |  |  |
|  | Islam | 1.17 | 1.00 | 1.29 | 1.00 |
|  | Collapsed affiliations with prevalence<3% | 1.88 | 1.00 | 1.48 | 1.00 |
| Race/ethnicity | (Ref: Plurality group) |  |  |  |  |
|  | Non-plurality groups | 1.63 | 1.00 | 1.17 | 1.00 |

***Table S15a. Nationally representative descriptive statistics for Poland***

| **Characteristic** | **N = 10,389**^1^ |
| --- | --- |
| **Relationship with mother** |  |
| Very good | 4,879 (47%) |
| Somewhat good | 4,973 (48%) |
| Somewhat bad | 285 (2.7%) |
| Very bad | 58 (0.6%) |
| Does not apply | 80 (0.8%) |
| (Missing) | 112 (1.1%) |
| **Relationship with father** |  |
| Very good | 4,231 (41%) |
| Somewhat good | 4,984 (48%) |
| Somewhat bad | 516 (5.0%) |
| Very bad | 78 (0.7%) |
| Does not apply | 407 (3.9%) |
| (Missing) | 173 (1.7%) |
| **Parent marital status** |  |
| Parents married | 8,972 (86%) |
| Divorced | 587 (5.7%) |
| Parents were never married | 193 (1.9%) |
| One or both parents had died | 313 (3.0%) |
| (Missing) | 324 (3.1%) |
| **Subjective financial status of family growing up** |  |
| Lived comfortably | 1,384 (13%) |
| Got by | 6,257 (60%) |
| Found it difficult | 2,133 (21%) |
| Found it very difficult | 509 (4.9%) |
| (Missing) | 106 (1.0%) |
| **Abuse** |  |
| Yes | 325 (3.1%) |
| No | 10,009 (96%) |
| (Missing) | 55 (0.5%) |
| **Outsider growing up** |  |
| Yes | 490 (4.7%) |
| No | 9,615 (93%) |
| (Missing) | 284 (2.7%) |
| **Self-rated health growing up** |  |
| Excellent | 2,676 (26%) |
| Very good | 5,371 (52%) |
| Good | 1,779 (17%) |
| Fair | 406 (3.9%) |
| Poor | 123 (1.2%) |
| (Missing) | 34 (0.3%) |
| **Immigration status** |  |
| Born in this country | 10,258 (99%) |
| Born in another country | 108 (1.0%) |
| (Missing) | 23 (0.2%) |
| **Age 12 religious service attendance** |  |
| At least 1/week | 4,751 (46%) |
| 1-3/month | 2,689 (26%) |
| <1/month | 2,161 (21%) |
| Never | 354 (3.4%) |
| (Missing) | 434 (4.2%) |
| **Year of birth** |  |
| 1998-2005; age 18-24 | 955 (9.2%) |
| 1993-1998; age 25-29 | 761 (7.3%) |
| 1983-1993; age 30-39 | 2,159 (21%) |
| 1973-1983; age 40-49 | 1,956 (19%) |
| 1963-1973; age 50-59 | 1,670 (16%) |
| 1953-1963; age 60-69 | 1,909 (18%) |
| 1943-1953; age 70-79 | 833 (8.0%) |
| 1943 or earlier; age 80+ | 145 (1.4%) |
| (Missing) | 1 (<0.1%) |
| **Gender** |  |
| Male | 4,974 (48%) |
| Female | 5,387 (52%) |
| Other | 3 (<0.1%) |
| (Missing) | 26 (0.2%) |
| **Religious affiliation** |  |
| Christianity | 9,861 (95%) |
| Islam | 3 (<0.1%) |
| Hinduism | 0 (0%) |
| Buddhism | 2 (<0.1%) |
| Judaism | 0 (0%) |
| Sikhism | 1 (<0.1%) |
| Baha'i | 0 (0%) |
| Jainism | 0 (0%) |
| Shinto | 0 (0%) |
| Taoism | 0 (0%) |
| Confucianism | 0 (0%) |
| Primal, Animist, or Folk religion | 5 (<0.1%) |
| Spiritism | 0 (0%) |
| Umbanda, Candomble, and other African-derived religions | 0 (0%) |
| Chinese folk/traditional religion | 0 (0%) |
| Some other religion | 0 (0%) |
| No religion/Atheist/Agnostic | 482 (4.6%) |
| (Missing) | 35 (0.3%) |
| **Race/Ethnicity** |  |
| Belarussian | 2 (<0.1%) |
| German | 4 (<0.1%) |
| Kashubians | 3 (<0.1%) |
| Other | 4 (<0.1%) |
| Polish | 10,309 (99%) |
| Silesia | 14 (0.1%) |
| Ukrainian | 38 (0.4%) |
| (Missing) | 14 (0.1%) |
| ^1^n (%) | |

***Table S15b. Childhood predictors regression analysis results for Poland***

|  | | Charitable giving | | | | Helping | | | |
| --- | --- | --- | --- | --- | --- | --- | --- | --- | --- |
| Variable | Category | Risk-Ratio | RR 95% CI | log(RR) SE | Global p-value | Risk-Ratio | RR 95% CI | log(RR) SE | Global p-value |
| Relationship with mother | (Ref: Very bad/somewhat bad) |  |  |  | 0.070 |  |  |  | 0.606 |
|  | Very good/somewhat good | 0.74 | (0.53,1.04) | 0.17 |  | 0.93 | (0.68,1.27) | 0.16 |  |
| Relationship with father | (Ref: Very bad/somewhat bad) |  |  |  | 0.744 |  |  |  | 0.591 |
|  | Very good/somewhat good | 0.96 | (0.73,1.27) | 0.14 |  | 0.95 | (0.74,1.22) | 0.13 |  |
| Parent marital status | (Ref: Parents married) |  |  |  | 0.176 |  |  |  | 0.064 |
|  | Divorced | 1.05 | (0.86,1.29) | 0.10 |  | 1.06 | (0.87,1.30) | 0.10 |  |
|  | Parents were never married | 0.85 | (0.46,1.59) | 0.29 |  | 1.03 | (0.77,1.38) | 0.15 |  |
|  | One or both parents had died | 1.39 | (1.00,1.93) | 0.17 |  | 1.39 | (1.08,1.78) | 0.13 |  |
| Subjective financial status of family growing up | (Ref: Got by) |  |  |  | 0.031 |  |  |  | 0.253 |
|  | Lived comfortably | 1.21 | (1.03,1.42) | 0.08 |  | 1.13 | (0.98,1.31) | 0.07 |  |
|  | Found it difficult | 1.14 | (0.99,1.33) | 0.07 |  | 0.96 | (0.83,1.10) | 0.07 |  |
|  | Found it very difficult | 1.30 | (0.95,1.80) | 0.16 |  | 0.91 | (0.66,1.25) | 0.16 |  |
| Abuse | (Ref: No) |  |  |  | 0.695 |  |  |  | 0.167 |
|  | Yes | 1.06 | (0.79,1.43) | 0.15 |  | 1.20 | (0.91,1.59) | 0.14 |  |
| Outsider growing up | (Ref: No) |  |  |  | 0.030 |  |  |  | 0.067 |
|  | Yes | 1.33 | (1.01,1.74) | 0.14 |  | 1.26 | (0.96,1.65) | 0.14 |  |
| Self-rated health growing up | (Ref: Good) |  |  |  | 0.139 |  |  |  | 0.001 |
|  | Excellent | 1.12 | (0.89,1.40) | 0.12 |  | 0.75 | (0.62,0.91) | 0.10 |  |
|  | Very good | 1.02 | (0.86,1.22) | 0.09 |  | 0.79 | (0.68,0.91) | 0.07 |  |
|  | Fair | 1.09 | (0.78,1.52) | 0.17 |  | 1.15 | (0.91,1.45) | 0.12 |  |
|  | Poor | 1.72 | (1.12,2.63) | 0.22 |  | 1.17 | (0.79,1.73) | 0.20 |  |
| Immigration status | (Ref: Born in this country) |  |  |  | 0.288 |  |  |  | 0.068 |
|  | Born in another country | 0.66 | (0.31,1.42) | 0.39 |  | 1.47 | (0.96,2.24) | 0.22 |  |
| Age 12 religious service attendance | (Ref: Never) |  |  |  | 0.002 |  |  |  | 0.004 |
|  | At least 1/week | 1.17 | (0.78,1.75) | 0.20 |  | 0.75 | (0.56,1.00) | 0.15 |  |
|  | 1-3/month | 1.02 | (0.67,1.53) | 0.21 |  | 0.91 | (0.68,1.22) | 0.15 |  |
|  | < 1/month | 0.82 | (0.55,1.22) | 0.20 |  | 0.95 | (0.71,1.28) | 0.15 |  |
| Year of birth | (Ref: 1998-2005; current age: 18-24) |  |  |  | <.001 |  |  |  | 0.097 |
|  | 1993-1998; age 25-29 | 1.61 | (1.21,2.15) | 0.15 |  | 1.00 | (0.81,1.24) | 0.11 |  |
|  | 1983-1993; age 30-39 | 1.98 | (1.51,2.62) | 0.14 |  | 1.19 | (0.97,1.45) | 0.10 |  |
|  | 1973-1983; age 40-49 | 1.66 | (1.25,2.22) | 0.15 |  | 1.21 | (1.00,1.47) | 0.10 |  |
|  | 1963-1973; age 50-59 | 1.60 | (1.21,2.11) | 0.14 |  | 1.10 | (0.91,1.33) | 0.10 |  |
|  | 1953-1963; age 60-69 | 1.82 | (1.34,2.47) | 0.16 |  | 1.23 | (0.99,1.52) | 0.11 |  |
|  | 1943-1953; age 70-79 | 2.10 | (1.46,3.03) | 0.19 |  | 1.38 | (1.04,1.84) | 0.15 |  |
|  | 1943 or earlier; age 80+ | 2.04 | (1.14,3.64) | 0.30 |  | 1.30 | (0.73,2.29) | 0.29 |  |
| Gender | (Ref: Male) |  |  |  | <.001 |  |  |  | <.001 |
|  | Female | 1.14 | (1.01,1.28) | 0.06 |  | 1.11 | (1.01,1.22) | 0.05 |  |
|  | Other | 0.00 | (0.00,0.00) | 0.84 |  | 0.00 | (0.00,0.00) | 0.65 |  |
| Religious affiliation | (Ref: No religion/Atheist/Agnostic) |  |  |  | 0.217 |  |  |  | 0.094 |
|  | Christianity | 1.20 | (0.88,1.65) | 0.16 |  | 1.31 | (1.02,1.69) | 0.13 |  |
|  | Collapsed affiliations with prevalence<3% | 0.33 | (0.04,2.49) | 1.02 |  | 1.34 | (0.69,2.59) | 0.34 |  |
| Race/ethnicity | (Ref: Plurality group) |  |  |  | 0.665 |  |  |  | 0.798 |
|  | Non-plurality groups | 1.16 | (0.59,2.30) | 0.35 |  | 1.06 | (0.60,1.87) | 0.29 |  |

***Table S15c. Sensitivity to unmeasured confounding of childhood predictors in Poland***

|  | | Charitable giving | | Helping | |
| --- | --- | --- | --- | --- | --- |
| Variable | Category | E-value for Estimate | E-value for 95% CI | E-value for Estimate | E-value for 95% CI |
| Relationship with mother | (Ref: Very bad/somewhat bad) |  |  |  |  |
|  | Very good/somewhat good | 2.03 | 1.00 | 1.38 | 1.00 |
| Relationship with father | (Ref: Very bad/somewhat bad) |  |  |  |  |
|  | Very good/somewhat good | 1.23 | 1.00 | 1.28 | 1.00 |
| Parent marital status | (Ref: Parents married) |  |  |  |  |
|  | Divorced | 1.28 | 1.00 | 1.32 | 1.00 |
|  | Parents were never married | 1.62 | 1.00 | 1.21 | 1.00 |
|  | One or both parents had died | 2.12 | 1.00 | 2.12 | 1.38 |
| Subjective financial status of family growing up | (Ref: Got by) |  |  |  |  |
|  | Lived comfortably | 1.72 | 1.21 | 1.52 | 1.00 |
|  | Found it difficult | 1.55 | 1.00 | 1.25 | 1.00 |
|  | Found it very difficult | 1.93 | 1.00 | 1.44 | 1.00 |
| Abuse | (Ref: No) |  |  |  |  |
|  | Yes | 1.31 | 1.00 | 1.70 | 1.00 |
| Outsider growing up | (Ref: No) |  |  |  |  |
|  | Yes | 1.99 | 1.13 | 1.83 | 1.00 |
| Self-rated health growing up | (Ref: Good) |  |  |  |  |
|  | Excellent | 1.48 | 1.00 | 1.99 | 1.41 |
|  | Very good | 1.17 | 1.00 | 1.86 | 1.43 |
|  | Fair | 1.40 | 1.00 | 1.56 | 1.00 |
|  | Poor | 2.82 | 1.49 | 1.61 | 1.00 |
| Immigration status | (Ref: Born in this country) |  |  |  |  |
|  | Born in another country | 2.40 | 1.00 | 2.30 | 1.00 |
| Age 12 religious service attendance | (Ref: Never) |  |  |  |  |
|  | At least 1/week | 1.61 | 1.00 | 2.00 | 1.00 |
|  | 1-3/month | 1.15 | 1.00 | 1.42 | 1.00 |
|  | < 1/month | 1.74 | 1.00 | 1.28 | 1.00 |
| Year of birth | (Ref: 1998-2005; current age: 18-24) |  |  |  |  |
|  | 1993-1998; age 25-29 | 2.61 | 1.71 | 1.03 | 1.00 |
|  | 1983-1993; age 30-39 | 3.38 | 2.38 | 1.66 | 1.00 |
|  | 1973-1983; age 40-49 | 2.72 | 1.81 | 1.72 | 1.06 |
|  | 1963-1973; age 50-59 | 2.58 | 1.72 | 1.43 | 1.00 |
|  | 1953-1963; age 60-69 | 3.04 | 2.01 | 1.75 | 1.00 |
|  | 1943-1953; age 70-79 | 3.62 | 2.27 | 2.11 | 1.23 |
|  | 1943 or earlier; age 80+ | 3.50 | 1.54 | 1.92 | 1.00 |
| Gender | (Ref: Male) |  |  |  |  |
|  | Female | 1.53 | 1.11 | 1.46 | 1.10 |
|  | Other | 65140.66 | 11309.52 | 110716.05 | 30813.69 |
| Religious affiliation | (Ref: No religion/Atheist/Agnostic) |  |  |  |  |
|  | Christianity | 1.70 | 1.00 | 1.96 | 1.18 |
|  | Collapsed affiliations with prevalence<3% | 5.49 | 1.00 | 2.01 | 1.00 |
| Race/ethnicity | (Ref: Plurality group) |  |  |  |  |
|  | Non-plurality groups | 1.60 | 1.00 | 1.32 | 1.00 |

***Table S16a. Nationally representative descriptive statistics for South Africa***

| **Characteristic** | **N = 2,651**^1^ |
| --- | --- |
| **Relationship with mother** |  |
| Very good | 2,186 (82%) |
| Somewhat good | 263 (9.9%) |
| Somewhat bad | 51 (1.9%) |
| Very bad | 39 (1.5%) |
| Does not apply | 90 (3.4%) |
| (Missing) | 21 (0.8%) |
| **Relationship with father** |  |
| Very good | 1,656 (62%) |
| Somewhat good | 333 (13%) |
| Somewhat bad | 86 (3.3%) |
| Very bad | 159 (6.0%) |
| Does not apply | 331 (12%) |
| (Missing) | 85 (3.2%) |
| **Parent marital status** |  |
| Parents married | 1,321 (50%) |
| Divorced | 131 (5.0%) |
| Parents were never married | 904 (34%) |
| One or both parents had died | 140 (5.3%) |
| (Missing) | 155 (5.8%) |
| **Subjective financial status of family growing up** |  |
| Lived comfortably | 1,050 (40%) |
| Got by | 875 (33%) |
| Found it difficult | 432 (16%) |
| Found it very difficult | 289 (11%) |
| (Missing) | 5 (0.2%) |
| **Abuse** |  |
| Yes | 450 (17%) |
| No | 2,149 (81%) |
| (Missing) | 52 (2.0%) |
| **Outsider growing up** |  |
| Yes | 434 (16%) |
| No | 2,211 (83%) |
| (Missing) | 6 (0.2%) |
| **Self-rated health growing up** |  |
| Excellent | 1,225 (46%) |
| Very good | 590 (22%) |
| Good | 370 (14%) |
| Fair | 266 (10%) |
| Poor | 183 (6.9%) |
| (Missing) | 17 (0.6%) |
| **Immigration status** |  |
| Born in this country | 2,511 (95%) |
| Born in another country | 139 (5.2%) |
| (Missing) | 1 (<0.1%) |
| **Age 12 religious service attendance** |  |
| At least 1/week | 1,681 (63%) |
| 1-3/month | 552 (21%) |
| <1/month | 175 (6.6%) |
| Never | 217 (8.2%) |
| (Missing) | 26 (1.0%) |
| **Year of birth** |  |
| 1998-2005; age 18-24 | 461 (17%) |
| 1993-1998; age 25-29 | 364 (14%) |
| 1983-1993; age 30-39 | 655 (25%) |
| 1973-1983; age 40-49 | 522 (20%) |
| 1963-1973; age 50-59 | 309 (12%) |
| 1953-1963; age 60-69 | 195 (7.4%) |
| 1943-1953; age 70-79 | 120 (4.5%) |
| 1943 or earlier; age 80+ | 17 (0.6%) |
| (Missing) | 9 (0.3%) |
| **Gender** |  |
| Male | 1,288 (49%) |
| Female | 1,356 (51%) |
| Other | 2 (<0.1%) |
| (Missing) | 4 (0.2%) |
| **Religious affiliation** |  |
| Christianity | 2,323 (88%) |
| Islam | 52 (2.0%) |
| Hinduism | 2 (<0.1%) |
| Buddhism | 11 (0.4%) |
| Judaism | 0 (0%) |
| Sikhism | 0 (0%) |
| Baha'i | 0 (0%) |
| Jainism | 0 (0%) |
| Shinto | 2 (<0.1%) |
| Taoism | 1 (<0.1%) |
| Confucianism | 0 (0%) |
| Primal, Animist, or Folk religion | 117 (4.4%) |
| Spiritism | 0 (0%) |
| Umbanda, Candomble, and other African-derived religions | 0 (0%) |
| Chinese folk/traditional religion | 0 (0%) |
| Some other religion | 7 (0.3%) |
| No religion/Atheist/Agnostic | 107 (4.1%) |
| (Missing) | 27 (1.0%) |
| **Race/Ethnicity** |  |
| Asian/Indian | 6 (0.2%) |
| Black | 2,381 (90%) |
| Colored | 252 (9.5%) |
| Other | 1 (<0.1%) |
| White | 8 (0.3%) |
| (Missing) | 3 (0.1%) |
| ^1^n (%) | |

***Table S16b. Childhood predictors regression analysis results for South Africa***

|  | | Charitable giving | | | | Helping | | | |
| --- | --- | --- | --- | --- | --- | --- | --- | --- | --- |
| Variable | Category | Risk-Ratio | RR 95% CI | log(RR) SE | Global p-value | Risk-Ratio | RR 95% CI | log(RR) SE | Global p-value |
| Relationship with mother | (Ref: Very bad/somewhat bad) |  |  |  | 0.928 |  |  |  | 0.273 |
|  | Very good/somewhat good | 1.02 | (0.64,1.62) | 0.24 |  | 0.89 | (0.72,1.11) | 0.11 |  |
| Relationship with father | (Ref: Very bad/somewhat bad) |  |  |  | 0.198 |  |  |  | 0.372 |
|  | Very good/somewhat good | 0.83 | (0.62,1.11) | 0.15 |  | 0.95 | (0.84,1.07) | 0.06 |  |
| Parent marital status | (Ref: Parents married) |  |  |  | 0.152 |  |  |  | 0.917 |
|  | Divorced | 1.46 | (0.97,2.18) | 0.21 |  | 1.04 | (0.86,1.24) | 0.09 |  |
|  | Parents were never married | 0.93 | (0.72,1.20) | 0.13 |  | 1.01 | (0.92,1.12) | 0.05 |  |
|  | One or both parents had died | 0.94 | (0.57,1.55) | 0.26 |  | 1.00 | (0.80,1.24) | 0.11 |  |
| Subjective financial status of family growing up | (Ref: Got by) |  |  |  | 0.618 |  |  |  | 0.249 |
|  | Lived comfortably | 1.15 | (0.92,1.43) | 0.11 |  | 1.06 | (0.96,1.16) | 0.05 |  |
|  | Found it difficult | 0.99 | (0.72,1.34) | 0.16 |  | 0.97 | (0.85,1.10) | 0.07 |  |
|  | Found it very difficult | 1.04 | (0.74,1.47) | 0.18 |  | 0.91 | (0.78,1.07) | 0.08 |  |
| Abuse | (Ref: No) |  |  |  | 0.008 |  |  |  | 0.279 |
|  | Yes | 1.38 | (1.09,1.76) | 0.12 |  | 1.06 | (0.95,1.17) | 0.05 |  |
| Outsider growing up | (Ref: No) |  |  |  | <.001 |  |  |  | 0.149 |
|  | Yes | 1.60 | (1.25,2.04) | 0.12 |  | 1.09 | (0.97,1.22) | 0.06 |  |
| Self-rated health growing up | (Ref: Good) |  |  |  | 0.229 |  |  |  | 0.222 |
|  | Excellent | 1.38 | (1.02,1.87) | 0.15 |  | 1.05 | (0.92,1.19) | 0.07 |  |
|  | Very good | 1.17 | (0.81,1.69) | 0.19 |  | 1.01 | (0.87,1.17) | 0.08 |  |
|  | Fair | 1.40 | (0.91,2.17) | 0.22 |  | 1.17 | (1.00,1.37) | 0.08 |  |
|  | Poor | 1.25 | (0.80,1.94) | 0.22 |  | 1.04 | (0.85,1.27) | 0.10 |  |
| Immigration status | (Ref: Born in this country) |  |  |  | 0.621 |  |  |  | 0.926 |
|  | Born in another country | 1.12 | (0.71,1.76) | 0.23 |  | 1.01 | (0.81,1.25) | 0.11 |  |
| Age 12 religious service attendance | (Ref: Never) |  |  |  | 0.329 |  |  |  | 0.020 |
|  | At least 1/week | 1.57 | (0.79,3.14) | 0.35 |  | 1.37 | (1.01,1.85) | 0.15 |  |
|  | 1-3/month | 1.33 | (0.66,2.66) | 0.35 |  | 1.27 | (0.93,1.72) | 0.16 |  |
|  | < 1/month | 1.34 | (0.57,3.13) | 0.43 |  | 1.09 | (0.76,1.57) | 0.18 |  |
| Year of birth | (Ref: 1998-2005; current age: 18-24) |  |  |  | 0.709 |  |  |  | 0.447 |
|  | 1993-1998; age 25-29 | 1.09 | (0.80,1.48) | 0.16 |  | 1.11 | (0.97,1.28) | 0.07 |  |
|  | 1983-1993; age 30-39 | 1.07 | (0.79,1.45) | 0.15 |  | 1.10 | (0.95,1.26) | 0.07 |  |
|  | 1973-1983; age 40-49 | 1.21 | (0.88,1.67) | 0.16 |  | 1.03 | (0.88,1.21) | 0.08 |  |
|  | 1963-1973; age 50-59 | 1.10 | (0.75,1.62) | 0.19 |  | 1.19 | (1.01,1.40) | 0.08 |  |
|  | 1953-1963; age 60-69 | 1.22 | (0.72,2.07) | 0.27 |  | 1.17 | (0.91,1.49) | 0.13 |  |
|  | 1943-1953; age 70-79 | 0.85 | (0.40,1.84) | 0.39 |  | 1.16 | (0.87,1.54) | 0.15 |  |
|  | 1943 or earlier; age 80+ | 0.35 | (0.08,1.62) | 0.78 |  | 0.48 | (0.10,2.25) | 0.79 |  |
| Gender | (Ref: Male) |  |  |  | 0.387 |  |  |  | <.001 |
|  | Female | 0.87 | (0.71,1.08) | 0.11 |  | 0.82 | (0.76,0.89) | 0.04 |  |
|  | Other | 1.58 | (0.28,8.85) | 0.88 |  | 1.15 | (0.62,2.14) | 0.31 |  |
| Religious affiliation | (Ref: No religion/Atheist/Agnostic) |  |  |  | 0.641 |  |  |  | 0.732 |
|  | Primal, Animist, or Folk religion | 0.66 | (0.24,1.82) | 0.51 |  | 1.15 | (0.73,1.82) | 0.23 |  |
|  | Christianity | 0.84 | (0.34,2.10) | 0.47 |  | 1.20 | (0.80,1.82) | 0.21 |  |
|  | Collapsed affiliations with prevalence<3% | 0.62 | (0.20,1.88) | 0.57 |  | 1.07 | (0.64,1.80) | 0.26 |  |
| Race/ethnicity | (Ref: Plurality group) |  |  |  | 0.172 |  |  |  | 0.715 |
|  | Non-plurality groups | 0.75 | (0.50,1.13) | 0.21 |  | 0.97 | (0.80,1.17) | 0.10 |  |

***Table S16c. Sensitivity to unmeasured confounding of childhood predictors in South Africa***

|  | | Charitable giving | | Helping | |
| --- | --- | --- | --- | --- | --- |
| Variable | Category | E-value for Estimate | E-value for 95% CI | E-value for Estimate | E-value for 95% CI |
| Relationship with mother | (Ref: Very bad/somewhat bad) |  |  |  |  |
|  | Very good/somewhat good | 1.15 | 1.00 | 1.48 | 1.00 |
| Relationship with father | (Ref: Very bad/somewhat bad) |  |  |  |  |
|  | Very good/somewhat good | 1.70 | 1.00 | 1.30 | 1.00 |
| Parent marital status | (Ref: Parents married) |  |  |  |  |
|  | Divorced | 2.27 | 1.00 | 1.23 | 1.00 |
|  | Parents were never married | 1.35 | 1.00 | 1.14 | 1.00 |
|  | One or both parents had died | 1.32 | 1.00 | 1.06 | 1.00 |
| Subjective financial status of family growing up | (Ref: Got by) |  |  |  |  |
|  | Lived comfortably | 1.56 | 1.00 | 1.30 | 1.00 |
|  | Found it difficult | 1.14 | 1.00 | 1.23 | 1.00 |
|  | Found it very difficult | 1.24 | 1.00 | 1.42 | 1.00 |
| Abuse | (Ref: No) |  |  |  |  |
|  | Yes | 2.11 | 1.41 | 1.31 | 1.00 |
| Outsider growing up | (Ref: No) |  |  |  |  |
|  | Yes | 2.57 | 1.81 | 1.39 | 1.00 |
| Self-rated health growing up | (Ref: Good) |  |  |  |  |
|  | Excellent | 2.10 | 1.15 | 1.26 | 1.00 |
|  | Very good | 1.61 | 1.00 | 1.09 | 1.00 |
|  | Fair | 2.16 | 1.00 | 1.62 | 1.02 |
|  | Poor | 1.80 | 1.00 | 1.23 | 1.00 |
| Immigration status | (Ref: Born in this country) |  |  |  |  |
|  | Born in another country | 1.49 | 1.00 | 1.09 | 1.00 |
| Age 12 religious service attendance | (Ref: Never) |  |  |  |  |
|  | At least 1/week | 2.53 | 1.00 | 2.08 | 1.13 |
|  | 1-3/month | 1.98 | 1.00 | 1.85 | 1.00 |
|  | < 1/month | 2.01 | 1.00 | 1.42 | 1.00 |
| Year of birth | (Ref: 1998-2005; current age: 18-24) |  |  |  |  |
|  | 1993-1998; age 25-29 | 1.40 | 1.00 | 1.47 | 1.00 |
|  | 1983-1993; age 30-39 | 1.34 | 1.00 | 1.42 | 1.00 |
|  | 1973-1983; age 40-49 | 1.71 | 1.00 | 1.20 | 1.00 |
|  | 1963-1973; age 50-59 | 1.44 | 1.00 | 1.66 | 1.08 |
|  | 1953-1963; age 60-69 | 1.75 | 1.00 | 1.61 | 1.00 |
|  | 1943-1953; age 70-79 | 1.62 | 1.00 | 1.59 | 1.00 |
|  | 1943 or earlier; age 80+ | 5.13 | 1.00 | 3.63 | 1.00 |
| Gender | (Ref: Male) |  |  |  |  |
|  | Female | 1.56 | 1.00 | 1.72 | 1.48 |
|  | Other | 2.53 | 1.00 | 1.58 | 1.00 |
| Religious affiliation | (Ref: No religion/Atheist/Agnostic) |  |  |  |  |
|  | Primal, Animist, or Folk religion | 2.38 | 1.00 | 1.57 | 1.00 |
|  | Christianity | 1.66 | 1.00 | 1.70 | 1.00 |
|  | Collapsed affiliations with prevalence<3% | 2.63 | 1.00 | 1.35 | 1.00 |
| Race/ethnicity | (Ref: Plurality group) |  |  |  |  |
|  | Non-plurality groups | 1.98 | 1.00 | 1.23 | 1.00 |

***Table S17a. Nationally representative descriptive statistics for Spain***

| **Characteristic** | **N = 6,290**^1^ |
| --- | --- |
| **Relationship with mother** |  |
| Very good | 4,557 (72%) |
| Somewhat good | 1,258 (20%) |
| Somewhat bad | 248 (3.9%) |
| Very bad | 92 (1.5%) |
| Does not apply | 107 (1.7%) |
| (Missing) | 28 (0.4%) |
| **Relationship with father** |  |
| Very good | 4,131 (66%) |
| Somewhat good | 1,397 (22%) |
| Somewhat bad | 309 (4.9%) |
| Very bad | 178 (2.8%) |
| Does not apply | 243 (3.9%) |
| (Missing) | 33 (0.5%) |
| **Parent marital status** |  |
| Parents married | 5,285 (84%) |
| Divorced | 378 (6.0%) |
| Parents were never married | 312 (5.0%) |
| One or both parents had died | 126 (2.0%) |
| (Missing) | 188 (3.0%) |
| **Subjective financial status of family growing up** |  |
| Lived comfortably | 2,041 (32%) |
| Got by | 2,956 (47%) |
| Found it difficult | 1,154 (18%) |
| Found it very difficult | 110 (1.7%) |
| (Missing) | 29 (0.5%) |
| **Abuse** |  |
| Yes | 659 (10%) |
| No | 5,510 (88%) |
| (Missing) | 122 (1.9%) |
| **Outsider growing up** |  |
| Yes | 579 (9.2%) |
| No | 5,637 (90%) |
| (Missing) | 75 (1.2%) |
| **Self-rated health growing up** |  |
| Excellent | 2,450 (39%) |
| Very good | 2,286 (36%) |
| Good | 1,235 (20%) |
| Fair | 164 (2.6%) |
| Poor | 135 (2.1%) |
| (Missing) | 20 (0.3%) |
| **Immigration status** |  |
| Born in this country | 5,479 (87%) |
| Born in another country | 788 (13%) |
| (Missing) | 23 (0.4%) |
| **Age 12 religious service attendance** |  |
| At least 1/week | 2,391 (38%) |
| 1-3/month | 1,132 (18%) |
| <1/month | 1,287 (20%) |
| Never | 1,445 (23%) |
| (Missing) | 36 (0.6%) |
| **Year of birth** |  |
| 1998-2005; age 18-24 | 594 (9.4%) |
| 1993-1998; age 25-29 | 450 (7.2%) |
| 1983-1993; age 30-39 | 1,111 (18%) |
| 1973-1983; age 40-49 | 1,396 (22%) |
| 1963-1973; age 50-59 | 1,252 (20%) |
| 1953-1963; age 60-69 | 977 (16%) |
| 1943-1953; age 70-79 | 467 (7.4%) |
| 1943 or earlier; age 80+ | 43 (0.7%) |
| (Missing) | 0 (0%) |
| **Gender** |  |
| Male | 3,142 (50%) |
| Female | 3,119 (50%) |
| Other | 6 (0.1%) |
| (Missing) | 22 (0.4%) |
| **Religious affiliation** |  |
| Christianity | 5,119 (81%) |
| Islam | 132 (2.1%) |
| Hinduism | 5 (<0.1%) |
| Buddhism | 8 (0.1%) |
| Judaism | 5 (<0.1%) |
| Sikhism | 2 (<0.1%) |
| Baha'i | 0 (0%) |
| Jainism | 0 (0%) |
| Shinto | 0 (0%) |
| Taoism | 0 (0%) |
| Confucianism | 1 (<0.1%) |
| Primal, Animist, or Folk religion | 4 (<0.1%) |
| Spiritism | 0 (0%) |
| Umbanda, Candomble, and other African-derived religions | 0 (0%) |
| Chinese folk/traditional religion | 0 (0%) |
| Some other religion | 13 (0.2%) |
| No religion/Atheist/Agnostic | 972 (15%) |
| (Missing) | 29 (0.5%) |
| ^1^n (%) | |

***Table S17b. Childhood predictors regression analysis results for Spain***

|  | | Charitable giving | | | | Helping | | | |
| --- | --- | --- | --- | --- | --- | --- | --- | --- | --- |
| Variable | Category | Risk-Ratio | RR 95% CI | log(RR) SE | Global p-value | Risk-Ratio | RR 95% CI | log(RR) SE | Global p-value |
| Relationship with mother | (Ref: Very bad/somewhat bad) |  |  |  | 0.270 |  |  |  | 0.939 |
|  | Very good/somewhat good | 1.13 | (0.91,1.42) | 0.11 |  | 1.00 | (0.89,1.14) | 0.06 |  |
| Relationship with father | (Ref: Very bad/somewhat bad) |  |  |  | 0.077 |  |  |  | 0.148 |
|  | Very good/somewhat good | 1.17 | (0.98,1.39) | 0.09 |  | 1.08 | (0.97,1.20) | 0.05 |  |
| Parent marital status | (Ref: Parents married) |  |  |  | 0.316 |  |  |  | 0.005 |
|  | Divorced | 0.95 | (0.77,1.17) | 0.11 |  | 1.14 | (1.02,1.27) | 0.06 |  |
|  | Parents were never married | 1.15 | (0.97,1.37) | 0.09 |  | 1.14 | (1.03,1.26) | 0.05 |  |
|  | One or both parents had died | 0.93 | (0.66,1.31) | 0.17 |  | 1.19 | (1.00,1.42) | 0.09 |  |
| Subjective financial status of family growing up | (Ref: Got by) |  |  |  | 0.162 |  |  |  | 0.483 |
|  | Lived comfortably | 1.06 | (0.95,1.18) | 0.05 |  | 1.04 | (0.97,1.12) | 0.04 |  |
|  | Found it difficult | 0.90 | (0.79,1.03) | 0.07 |  | 1.00 | (0.91,1.09) | 0.05 |  |
|  | Found it very difficult | 0.97 | (0.68,1.38) | 0.18 |  | 1.12 | (0.92,1.35) | 0.10 |  |
| Abuse | (Ref: No) |  |  |  | 0.010 |  |  |  | 0.017 |
|  | Yes | 1.19 | (1.04,1.36) | 0.07 |  | 1.11 | (1.02,1.22) | 0.05 |  |
| Outsider growing up | (Ref: No) |  |  |  | 0.376 |  |  |  | 0.018 |
|  | Yes | 1.06 | (0.93,1.22) | 0.07 |  | 1.11 | (1.02,1.21) | 0.04 |  |
| Self-rated health growing up | (Ref: Good) |  |  |  | 0.006 |  |  |  | 0.588 |
|  | Excellent | 0.79 | (0.70,0.90) | 0.07 |  | 1.02 | (0.93,1.12) | 0.05 |  |
|  | Very good | 0.84 | (0.74,0.95) | 0.06 |  | 1.07 | (0.97,1.17) | 0.05 |  |
|  | Fair | 0.94 | (0.71,1.25) | 0.15 |  | 1.09 | (0.90,1.33) | 0.10 |  |
|  | Poor | 0.79 | (0.54,1.16) | 0.19 |  | 1.04 | (0.83,1.29) | 0.11 |  |
| Immigration status | (Ref: Born in this country) |  |  |  | <.001 |  |  |  | <.001 |
|  | Born in another country | 0.71 | (0.61,0.83) | 0.08 |  | 1.14 | (1.06,1.23) | 0.04 |  |
| Age 12 religious service attendance | (Ref: Never) |  |  |  | 0.001 |  |  |  | 0.399 |
|  | At least 1/week | 1.16 | (1.02,1.32) | 0.06 |  | 0.99 | (0.91,1.08) | 0.04 |  |
|  | 1-3/month | 0.99 | (0.85,1.14) | 0.07 |  | 1.01 | (0.92,1.10) | 0.05 |  |
|  | < 1/month | 0.94 | (0.81,1.08) | 0.07 |  | 0.94 | (0.86,1.03) | 0.05 |  |
| Year of birth | (Ref: 1998-2005; current age: 18-24) |  |  |  | 0.001 |  |  |  | <.001 |
|  | 1993-1998; age 25-29 | 1.51 | (1.18,1.94) | 0.13 |  | 0.91 | (0.80,1.02) | 0.06 |  |
|  | 1983-1993; age 30-39 | 1.50 | (1.22,1.86) | 0.11 |  | 0.85 | (0.77,0.93) | 0.05 |  |
|  | 1973-1983; age 40-49 | 1.50 | (1.22,1.84) | 0.11 |  | 0.85 | (0.77,0.93) | 0.05 |  |
|  | 1963-1973; age 50-59 | 1.52 | (1.23,1.88) | 0.11 |  | 0.77 | (0.69,0.86) | 0.05 |  |
|  | 1953-1963; age 60-69 | 1.46 | (1.16,1.85) | 0.12 |  | 0.60 | (0.52,0.70) | 0.07 |  |
|  | 1943-1953; age 70-79 | 1.24 | (0.92,1.66) | 0.15 |  | 0.54 | (0.43,0.67) | 0.11 |  |
|  | 1943 or earlier; age 80+ | 2.08 | (1.43,3.00) | 0.19 |  | 0.61 | (0.36,1.04) | 0.27 |  |
| Gender | (Ref: Male) |  |  |  | <.001 |  |  |  | <.001 |
|  | Female | 0.74 | (0.68,0.81) | 0.05 |  | 0.77 | (0.72,0.82) | 0.03 |  |
|  | Other | 0.00 | (0.00,0.00) | 0.34 |  | 1.18 | (0.88,1.58) | 0.15 |  |
| Religious affiliation | (Ref: No religion/Atheist/Agnostic) |  |  |  | <.001 |  |  |  | <.001 |
|  | Christianity | 1.73 | (1.45,2.07) | 0.09 |  | 1.74 | (1.57,1.94) | 0.05 |  |
|  | Collapsed affiliations with prevalence<3% | 1.43 | (1.00,2.06) | 0.19 |  | 1.33 | (1.08,1.64) | 0.11 |  |
| Race/ethnicity | (Ref: Plurality group) |  |  |  |  |  |  |  |  |

***Table S17c. Sensitivity to unmeasured confounding of childhood predictors in Spain***

|  | | Charitable giving | | Helping | |
| --- | --- | --- | --- | --- | --- |
| Variable | Category | E-value for Estimate | E-value for 95% CI | E-value for Estimate | E-value for 95% CI |
| Relationship with mother | (Ref: Very bad/somewhat bad) |  |  |  |  |
|  | Very good/somewhat good | 1.52 | 1.00 | 1.07 | 1.00 |
| Relationship with father | (Ref: Very bad/somewhat bad) |  |  |  |  |
|  | Very good/somewhat good | 1.61 | 1.00 | 1.37 | 1.00 |
| Parent marital status | (Ref: Parents married) |  |  |  |  |
|  | Divorced | 1.30 | 1.00 | 1.53 | 1.16 |
|  | Parents were never married | 1.57 | 1.00 | 1.54 | 1.19 |
|  | One or both parents had died | 1.36 | 1.00 | 1.67 | 1.00 |
| Subjective financial status of family growing up | (Ref: Got by) |  |  |  |  |
|  | Lived comfortably | 1.32 | 1.00 | 1.24 | 1.00 |
|  | Found it difficult | 1.47 | 1.00 | 1.05 | 1.00 |
|  | Found it very difficult | 1.21 | 1.00 | 1.48 | 1.00 |
| Abuse | (Ref: No) |  |  |  |  |
|  | Yes | 1.67 | 1.25 | 1.47 | 1.15 |
| Outsider growing up | (Ref: No) |  |  |  |  |
|  | Yes | 1.33 | 1.00 | 1.46 | 1.15 |
| Self-rated health growing up | (Ref: Good) |  |  |  |  |
|  | Excellent | 1.83 | 1.46 | 1.16 | 1.00 |
|  | Very good | 1.67 | 1.30 | 1.33 | 1.00 |
|  | Fair | 1.32 | 1.00 | 1.41 | 1.00 |
|  | Poor | 1.83 | 1.00 | 1.24 | 1.00 |
| Immigration status | (Ref: Born in this country) |  |  |  |  |
|  | Born in another country | 2.17 | 1.71 | 1.55 | 1.32 |
| Age 12 religious service attendance | (Ref: Never) |  |  |  |  |
|  | At least 1/week | 1.60 | 1.18 | 1.09 | 1.00 |
|  | 1-3/month | 1.14 | 1.00 | 1.10 | 1.00 |
|  | < 1/month | 1.34 | 1.00 | 1.32 | 1.00 |
| Year of birth | (Ref: 1998-2005; current age: 18-24) |  |  |  |  |
|  | 1993-1998; age 25-29 | 2.40 | 1.65 | 1.44 | 1.00 |
|  | 1983-1993; age 30-39 | 2.37 | 1.73 | 1.64 | 1.35 |
|  | 1973-1983; age 40-49 | 2.36 | 1.73 | 1.64 | 1.36 |
|  | 1963-1973; age 50-59 | 2.41 | 1.76 | 1.92 | 1.61 |
|  | 1953-1963; age 60-69 | 2.29 | 1.59 | 2.71 | 2.23 |
|  | 1943-1953; age 70-79 | 1.78 | 1.00 | 3.14 | 2.35 |
|  | 1943 or earlier; age 80+ | 3.57 | 2.22 | 2.66 | 1.00 |
| Gender | (Ref: Male) |  |  |  |  |
|  | Female | 2.03 | 1.76 | 1.92 | 1.74 |
|  | Other | 510850.48 | 260242.68 | 1.64 | 1.00 |
| Religious affiliation | (Ref: No religion/Atheist/Agnostic) |  |  |  |  |
|  | Christianity | 2.86 | 2.26 | 2.88 | 2.51 |
|  | Collapsed affiliations with prevalence<3% | 2.22 | 1.00 | 1.99 | 1.37 |
| Race/ethnicity | (Ref: Plurality group) |  |  |  |  |

***Table S18a. Nationally representative descriptive statistics for Sweden***

| **Characteristic** | **N = 15,068**^1^ |
| --- | --- |
| **Relationship with mother** |  |
| Very good | 8,743 (58%) |
| Somewhat good | 4,513 (30%) |
| Somewhat bad | 1,194 (7.9%) |
| Very bad | 371 (2.5%) |
| Does not apply | 216 (1.4%) |
| (Missing) | 30 (0.2%) |
| **Relationship with father** |  |
| Very good | 7,134 (47%) |
| Somewhat good | 4,885 (32%) |
| Somewhat bad | 1,588 (11%) |
| Very bad | 725 (4.8%) |
| Does not apply | 720 (4.8%) |
| (Missing) | 16 (0.1%) |
| **Parent marital status** |  |
| Parents married | 10,887 (72%) |
| Divorced | 1,927 (13%) |
| Parents were never married | 1,747 (12%) |
| One or both parents had died | 362 (2.4%) |
| (Missing) | 145 (1.0%) |
| **Subjective financial status of family growing up** |  |
| Lived comfortably | 5,951 (39%) |
| Got by | 7,717 (51%) |
| Found it difficult | 1,238 (8.2%) |
| Found it very difficult | 140 (0.9%) |
| (Missing) | 22 (0.1%) |
| **Abuse** |  |
| Yes | 2,288 (15%) |
| No | 12,735 (85%) |
| (Missing) | 45 (0.3%) |
| **Outsider growing up** |  |
| Yes | 1,867 (12%) |
| No | 13,034 (86%) |
| (Missing) | 168 (1.1%) |
| **Self-rated health growing up** |  |
| Excellent | 5,733 (38%) |
| Very good | 5,124 (34%) |
| Good | 2,669 (18%) |
| Fair | 1,108 (7.4%) |
| Poor | 397 (2.6%) |
| (Missing) | 38 (0.2%) |
| **Immigration status** |  |
| Born in this country | 13,922 (92%) |
| Born in another country | 1,052 (7.0%) |
| (Missing) | 94 (0.6%) |
| **Age 12 religious service attendance** |  |
| At least 1/week | 955 (6.3%) |
| 1-3/month | 1,362 (9.0%) |
| <1/month | 6,224 (41%) |
| Never | 6,472 (43%) |
| (Missing) | 54 (0.4%) |
| **Year of birth** |  |
| 1998-2005; age 18-24 | 1,515 (10%) |
| 1993-1998; age 25-29 | 1,399 (9.3%) |
| 1983-1993; age 30-39 | 2,398 (16%) |
| 1973-1983; age 40-49 | 2,221 (15%) |
| 1963-1973; age 50-59 | 2,493 (17%) |
| 1953-1963; age 60-69 | 2,168 (14%) |
| 1943-1953; age 70-79 | 2,253 (15%) |
| 1943 or earlier; age 80+ | 621 (4.1%) |
| (Missing) | 0 (0%) |
| **Gender** |  |
| Male | 7,536 (50%) |
| Female | 7,493 (50%) |
| Other | 27 (0.2%) |
| (Missing) | 12 (<0.1%) |
| **Religious affiliation** |  |
| Christianity | 10,617 (70%) |
| Islam | 462 (3.1%) |
| Hinduism | 16 (0.1%) |
| Buddhism | 41 (0.3%) |
| Judaism | 51 (0.3%) |
| Sikhism | 9 (<0.1%) |
| Baha'i | 3 (<0.1%) |
| Jainism | 0 (0%) |
| Shinto | 1 (<0.1%) |
| Taoism | 0 (0%) |
| Confucianism | 4 (<0.1%) |
| Primal, Animist, or Folk religion | 31 (0.2%) |
| Spiritism | 0 (0%) |
| Umbanda, Candomble, and other African-derived religions | 0 (0%) |
| Chinese folk/traditional religion | 0 (0%) |
| Some other religion | 69 (0.5%) |
| No religion/Atheist/Agnostic | 3,738 (25%) |
| (Missing) | 26 (0.2%) |
| ^1^n (%) | |

***Table S18b. Childhood predictors regression analysis results for Sweden***

|  | | Charitable giving | | | | Helping | | | |
| --- | --- | --- | --- | --- | --- | --- | --- | --- | --- |
| Variable | Category | Risk-Ratio | RR 95% CI | log(RR) SE | Global p-value | Risk-Ratio | RR 95% CI | log(RR) SE | Global p-value |
| Relationship with mother | (Ref: Very bad/somewhat bad) |  |  |  | 0.627 |  |  |  | 0.714 |
|  | Very good/somewhat good | 1.02 | (0.95,1.09) | 0.03 |  | 1.01 | (0.94,1.09) | 0.04 |  |
| Relationship with father | (Ref: Very bad/somewhat bad) |  |  |  | 0.002 |  |  |  | 0.734 |
|  | Very good/somewhat good | 1.10 | (1.03,1.16) | 0.03 |  | 1.01 | (0.95,1.07) | 0.03 |  |
| Parent marital status | (Ref: Parents married) |  |  |  | 0.558 |  |  |  | 0.703 |
|  | Divorced | 0.97 | (0.91,1.04) | 0.03 |  | 1.03 | (0.97,1.10) | 0.03 |  |
|  | Parents were never married | 0.95 | (0.88,1.03) | 0.04 |  | 1.00 | (0.94,1.06) | 0.03 |  |
|  | One or both parents had died | 1.01 | (0.89,1.14) | 0.06 |  | 1.00 | (0.85,1.18) | 0.08 |  |
| Subjective financial status of family growing up | (Ref: Got by) |  |  |  | 0.328 |  |  |  | 0.510 |
|  | Lived comfortably | 1.03 | (0.99,1.07) | 0.02 |  | 0.97 | (0.92,1.01) | 0.02 |  |
|  | Found it difficult | 1.05 | (0.98,1.13) | 0.04 |  | 1.01 | (0.94,1.09) | 0.04 |  |
|  | Found it very difficult | 1.04 | (0.85,1.26) | 0.10 |  | 1.02 | (0.86,1.20) | 0.08 |  |
| Abuse | (Ref: No) |  |  |  | 0.313 |  |  |  | <.001 |
|  | Yes | 1.03 | (0.97,1.08) | 0.03 |  | 1.20 | (1.14,1.27) | 0.03 |  |
| Outsider growing up | (Ref: No) |  |  |  | 0.186 |  |  |  | 0.367 |
|  | Yes | 1.04 | (0.98,1.12) | 0.03 |  | 1.03 | (0.97,1.09) | 0.03 |  |
| Self-rated health growing up | (Ref: Good) |  |  |  | 0.095 |  |  |  | 0.478 |
|  | Excellent | 1.07 | (1.01,1.13) | 0.03 |  | 0.99 | (0.93,1.05) | 0.03 |  |
|  | Very good | 1.04 | (0.98,1.10) | 0.03 |  | 1.00 | (0.95,1.07) | 0.03 |  |
|  | Fair | 1.01 | (0.93,1.10) | 0.04 |  | 1.06 | (0.98,1.15) | 0.04 |  |
|  | Poor | 0.93 | (0.80,1.07) | 0.07 |  | 1.01 | (0.89,1.13) | 0.06 |  |
| Immigration status | (Ref: Born in this country) |  |  |  | 0.046 |  |  |  | 0.293 |
|  | Born in another country | 0.92 | (0.86,1.00) | 0.04 |  | 1.04 | (0.97,1.12) | 0.04 |  |
| Age 12 religious service attendance | (Ref: Never) |  |  |  | <.001 |  |  |  | <.001 |
|  | At least 1/week | 1.43 | (1.34,1.52) | 0.03 |  | 1.35 | (1.24,1.47) | 0.04 |  |
|  | 1-3/month | 1.39 | (1.31,1.47) | 0.03 |  | 1.34 | (1.24,1.44) | 0.04 |  |
|  | < 1/month | 1.16 | (1.12,1.22) | 0.02 |  | 1.20 | (1.14,1.26) | 0.02 |  |
| Year of birth | (Ref: 1998-2005; current age: 18-24) |  |  |  | <.001 |  |  |  | <.001 |
|  | 1993-1998; age 25-29 | 1.23 | (1.10,1.38) | 0.06 |  | 0.91 | (0.85,0.96) | 0.03 |  |
|  | 1983-1993; age 30-39 | 1.42 | (1.29,1.57) | 0.05 |  | 0.79 | (0.74,0.83) | 0.03 |  |
|  | 1973-1983; age 40-49 | 1.56 | (1.41,1.72) | 0.05 |  | 0.70 | (0.66,0.75) | 0.04 |  |
|  | 1963-1973; age 50-59 | 1.58 | (1.44,1.75) | 0.05 |  | 0.62 | (0.58,0.67) | 0.04 |  |
|  | 1953-1963; age 60-69 | 1.75 | (1.59,1.93) | 0.05 |  | 0.43 | (0.39,0.47) | 0.05 |  |
|  | 1943-1953; age 70-79 | 2.01 | (1.82,2.21) | 0.05 |  | 0.31 | (0.28,0.35) | 0.06 |  |
|  | 1943 or earlier; age 80+ | 2.10 | (1.89,2.34) | 0.05 |  | 0.24 | (0.19,0.30) | 0.12 |  |
| Gender | (Ref: Male) |  |  |  | <.001 |  |  |  | 0.075 |
|  | Female | 1.16 | (1.12,1.21) | 0.02 |  | 1.04 | (1.00,1.09) | 0.02 |  |
|  | Other | 1.41 | (0.93,2.16) | 0.22 |  | 0.75 | (0.49,1.14) | 0.21 |  |
| Religious affiliation | (Ref: No religion/Atheist/Agnostic) |  |  |  | <.001 |  |  |  | <.001 |
|  | Islam | 1.49 | (1.31,1.69) | 0.06 |  | 1.19 | (1.08,1.32) | 0.05 |  |
|  | Christianity | 1.08 | (1.03,1.15) | 0.03 |  | 1.00 | (0.95,1.05) | 0.03 |  |
|  | Collapsed affiliations with prevalence<3% | 1.14 | (0.94,1.39) | 0.10 |  | 1.21 | (1.05,1.40) | 0.07 |  |
| Race/ethnicity | (Ref: Plurality group) |  |  |  |  |  |  |  |  |

***Table S18c. Sensitivity to unmeasured confounding of childhood predictors in Sweden***

|  | | Charitable giving | | Helping | |
| --- | --- | --- | --- | --- | --- |
| Variable | Category | E-value for Estimate | E-value for 95% CI | E-value for Estimate | E-value for 95% CI |
| Relationship with mother | (Ref: Very bad/somewhat bad) |  |  |  |  |
|  | Very good/somewhat good | 1.15 | 1.00 | 1.13 | 1.00 |
| Relationship with father | (Ref: Very bad/somewhat bad) |  |  |  |  |
|  | Very good/somewhat good | 1.42 | 1.22 | 1.11 | 1.00 |
| Parent marital status | (Ref: Parents married) |  |  |  |  |
|  | Divorced | 1.19 | 1.00 | 1.22 | 1.00 |
|  | Parents were never married | 1.28 | 1.00 | 1.04 | 1.00 |
|  | One or both parents had died | 1.08 | 1.00 | 1.05 | 1.00 |
| Subjective financial status of family growing up | (Ref: Got by) |  |  |  |  |
|  | Lived comfortably | 1.21 | 1.00 | 1.22 | 1.00 |
|  | Found it difficult | 1.28 | 1.00 | 1.10 | 1.00 |
|  | Found it very difficult | 1.23 | 1.00 | 1.16 | 1.00 |
| Abuse | (Ref: No) |  |  |  |  |
|  | Yes | 1.20 | 1.00 | 1.70 | 1.54 |
| Outsider growing up | (Ref: No) |  |  |  |  |
|  | Yes | 1.26 | 1.00 | 1.20 | 1.00 |
| Self-rated health growing up | (Ref: Good) |  |  |  |  |
|  | Excellent | 1.33 | 1.10 | 1.14 | 1.00 |
|  | Very good | 1.25 | 1.00 | 1.07 | 1.00 |
|  | Fair | 1.09 | 1.00 | 1.32 | 1.00 |
|  | Poor | 1.37 | 1.00 | 1.08 | 1.00 |
| Immigration status | (Ref: Born in this country) |  |  |  |  |
|  | Born in another country | 1.38 | 1.03 | 1.25 | 1.00 |
| Age 12 religious service attendance | (Ref: Never) |  |  |  |  |
|  | At least 1/week | 2.21 | 2.01 | 2.04 | 1.80 |
|  | 1-3/month | 2.13 | 1.95 | 2.01 | 1.79 |
|  | < 1/month | 1.60 | 1.47 | 1.69 | 1.55 |
| Year of birth | (Ref: 1998-2005; current age: 18-24) |  |  |  |  |
|  | 1993-1998; age 25-29 | 1.77 | 1.44 | 1.44 | 1.24 |
|  | 1983-1993; age 30-39 | 2.20 | 1.90 | 1.86 | 1.69 |
|  | 1973-1983; age 40-49 | 2.49 | 2.17 | 2.19 | 1.98 |
|  | 1963-1973; age 50-59 | 2.55 | 2.23 | 2.59 | 2.35 |
|  | 1953-1963; age 60-69 | 2.90 | 2.56 | 4.12 | 3.71 |
|  | 1943-1953; age 70-79 | 3.43 | 3.05 | 5.94 | 5.24 |
|  | 1943 or earlier; age 80+ | 3.62 | 3.19 | 7.76 | 6.06 |
| Gender | (Ref: Male) |  |  |  |  |
|  | Female | 1.60 | 1.49 | 1.24 | 1.00 |
|  | Other | 2.18 | 1.00 | 2.00 | 1.00 |
| Religious affiliation | (Ref: No religion/Atheist/Agnostic) |  |  |  |  |
|  | Islam | 2.34 | 1.95 | 1.68 | 1.38 |
|  | Christianity | 1.39 | 1.19 | 1.03 | 1.00 |
|  | Collapsed affiliations with prevalence<3% | 1.55 | 1.00 | 1.72 | 1.27 |
| Race/ethnicity | (Ref: Plurality group) |  |  |  |  |

***Table S19a. Nationally representative descriptive statistics for Tanzania***

| **Characteristic** | **N = 9,075**^1^ |
| --- | --- |
| **Relationship with mother** |  |
| Very good | 7,739 (85%) |
| Somewhat good | 796 (8.8%) |
| Somewhat bad | 84 (0.9%) |
| Very bad | 84 (0.9%) |
| Does not apply | 303 (3.3%) |
| (Missing) | 70 (0.8%) |
| **Relationship with father** |  |
| Very good | 6,831 (75%) |
| Somewhat good | 1,101 (12%) |
| Somewhat bad | 203 (2.2%) |
| Very bad | 247 (2.7%) |
| Does not apply | 550 (6.1%) |
| (Missing) | 142 (1.6%) |
| **Parent marital status** |  |
| Parents married | 6,929 (76%) |
| Divorced | 678 (7.5%) |
| Parents were never married | 751 (8.3%) |
| One or both parents had died | 313 (3.4%) |
| (Missing) | 404 (4.4%) |
| **Subjective financial status of family growing up** |  |
| Lived comfortably | 2,611 (29%) |
| Got by | 2,909 (32%) |
| Found it difficult | 2,679 (30%) |
| Found it very difficult | 814 (9.0%) |
| (Missing) | 61 (0.7%) |
| **Abuse** |  |
| Yes | 716 (7.9%) |
| No | 8,328 (92%) |
| (Missing) | 32 (0.3%) |
| **Outsider growing up** |  |
| Yes | 734 (8.1%) |
| No | 8,320 (92%) |
| (Missing) | 22 (0.2%) |
| **Self-rated health growing up** |  |
| Excellent | 2,406 (27%) |
| Very good | 2,036 (22%) |
| Good | 2,946 (32%) |
| Fair | 1,177 (13%) |
| Poor | 456 (5.0%) |
| (Missing) | 54 (0.6%) |
| **Immigration status** |  |
| Born in this country | 9,048 (100%) |
| Born in another country | 25 (0.3%) |
| (Missing) | 1 (<0.1%) |
| **Age 12 religious service attendance** |  |
| At least 1/week | 5,580 (61%) |
| 1-3/month | 2,383 (26%) |
| <1/month | 333 (3.7%) |
| Never | 595 (6.6%) |
| (Missing) | 184 (2.0%) |
| **Year of birth** |  |
| 1998-2005; age 18-24 | 2,284 (25%) |
| 1993-1998; age 25-29 | 1,349 (15%) |
| 1983-1993; age 30-39 | 2,060 (23%) |
| 1973-1983; age 40-49 | 1,503 (17%) |
| 1963-1973; age 50-59 | 912 (10%) |
| 1953-1963; age 60-69 | 575 (6.3%) |
| 1943-1953; age 70-79 | 297 (3.3%) |
| 1943 or earlier; age 80+ | 93 (1.0%) |
| (Missing) | 2 (<0.1%) |
| **Gender** |  |
| Male | 4,299 (47%) |
| Female | 4,776 (53%) |
| Other | 0 (0%) |
| (Missing) | 0 (0%) |
| **Religious affiliation** |  |
| Christianity | 5,651 (62%) |
| Islam | 3,060 (34%) |
| Hinduism | 0 (0%) |
| Buddhism | 0 (0%) |
| Judaism | 0 (0%) |
| Sikhism | 0 (0%) |
| Baha'i | 1 (<0.1%) |
| Jainism | 0 (0%) |
| Shinto | 0 (0%) |
| Taoism | 0 (0%) |
| Confucianism | 0 (0%) |
| Primal, Animist, or Folk religion | 11 (0.1%) |
| Spiritism | 0 (0%) |
| Umbanda, Candomble, and other African-derived religions | 0 (0%) |
| Chinese folk/traditional religion | 0 (0%) |
| Some other religion | 0 (0%) |
| No religion/Atheist/Agnostic | 345 (3.8%) |
| (Missing) | 7 (<0.1%) |
| **Race/Ethnicity** |  |
| African | 9,060 (100%) |
| Arab | 11 (0.1%) |
| Indian | 3 (<0.1%) |
| (Missing) | 2 (<0.1%) |
| ^1^n (%) | |

***Table S19b. Childhood predictors regression analysis results for Tanzania***

|  | | Charitable giving | | | | Helping | | | |
| --- | --- | --- | --- | --- | --- | --- | --- | --- | --- |
| Variable | Category | Risk-Ratio | RR 95% CI | log(RR) SE | Global p-value | Risk-Ratio | RR 95% CI | log(RR) SE | Global p-value |
| Relationship with mother | (Ref: Very bad/somewhat bad) |  |  |  | 0.852 |  |  |  | 0.795 |
|  | Very good/somewhat good | 0.98 | (0.74,1.29) | 0.14 |  | 1.02 | (0.82,1.26) | 0.11 |  |
| Relationship with father | (Ref: Very bad/somewhat bad) |  |  |  | 0.639 |  |  |  | 0.672 |
|  | Very good/somewhat good | 1.05 | (0.86,1.27) | 0.10 |  | 1.03 | (0.88,1.21) | 0.08 |  |
| Parent marital status | (Ref: Parents married) |  |  |  | 0.961 |  |  |  | 0.591 |
|  | Divorced | 1.01 | (0.89,1.16) | 0.07 |  | 1.09 | (0.95,1.24) | 0.07 |  |
|  | Parents were never married | 0.97 | (0.82,1.16) | 0.09 |  | 1.01 | (0.90,1.14) | 0.06 |  |
|  | One or both parents had died | 1.01 | (0.79,1.28) | 0.12 |  | 1.05 | (0.86,1.27) | 0.10 |  |
| Subjective financial status of family growing up | (Ref: Got by) |  |  |  | 0.060 |  |  |  | 0.850 |
|  | Lived comfortably | 0.92 | (0.83,1.03) | 0.05 |  | 0.98 | (0.89,1.08) | 0.05 |  |
|  | Found it difficult | 0.88 | (0.79,0.98) | 0.05 |  | 1.01 | (0.93,1.11) | 0.05 |  |
|  | Found it very difficult | 0.84 | (0.71,0.99) | 0.09 |  | 0.95 | (0.81,1.11) | 0.08 |  |
| Abuse | (Ref: No) |  |  |  | 0.003 |  |  |  | 0.027 |
|  | Yes | 1.24 | (1.07,1.42) | 0.07 |  | 1.13 | (1.02,1.26) | 0.06 |  |
| Outsider growing up | (Ref: No) |  |  |  | 0.135 |  |  |  | 0.024 |
|  | Yes | 1.11 | (0.97,1.27) | 0.07 |  | 1.14 | (1.02,1.29) | 0.06 |  |
| Self-rated health growing up | (Ref: Good) |  |  |  | 0.016 |  |  |  | <.001 |
|  | Excellent | 1.19 | (1.07,1.31) | 0.05 |  | 1.21 | (1.10,1.33) | 0.05 |  |
|  | Very good | 1.06 | (0.95,1.19) | 0.06 |  | 1.16 | (1.05,1.29) | 0.05 |  |
|  | Fair | 1.07 | (0.93,1.22) | 0.07 |  | 1.19 | (1.05,1.34) | 0.06 |  |
|  | Poor | 1.13 | (0.92,1.39) | 0.11 |  | 1.29 | (1.09,1.54) | 0.09 |  |
| Immigration status | (Ref: Born in this country) |  |  |  | 0.013 |  |  |  | 0.181 |
|  | Born in another country | 1.94 | (1.16,3.26) | 0.26 |  | 1.36 | (0.87,2.13) | 0.23 |  |
| Age 12 religious service attendance | (Ref: Never) |  |  |  | 0.011 |  |  |  | 0.014 |
|  | At least 1/week | 1.39 | (1.04,1.85) | 0.15 |  | 1.40 | (1.11,1.77) | 0.12 |  |
|  | 1-3/month | 1.36 | (1.01,1.83) | 0.15 |  | 1.44 | (1.12,1.85) | 0.13 |  |
|  | < 1/month | 0.97 | (0.67,1.40) | 0.19 |  | 1.22 | (0.90,1.65) | 0.15 |  |
| Year of birth | (Ref: 1998-2005; current age: 18-24) |  |  |  | <.001 |  |  |  | 0.013 |
|  | 1993-1998; age 25-29 | 1.20 | (1.05,1.37) | 0.07 |  | 1.06 | (0.94,1.19) | 0.06 |  |
|  | 1983-1993; age 30-39 | 1.05 | (0.93,1.18) | 0.06 |  | 1.07 | (0.96,1.19) | 0.05 |  |
|  | 1973-1983; age 40-49 | 1.03 | (0.90,1.17) | 0.07 |  | 1.13 | (1.01,1.25) | 0.05 |  |
|  | 1963-1973; age 50-59 | 1.03 | (0.88,1.21) | 0.08 |  | 1.23 | (1.08,1.39) | 0.07 |  |
|  | 1953-1963; age 60-69 | 0.97 | (0.79,1.19) | 0.10 |  | 1.18 | (1.00,1.39) | 0.08 |  |
|  | 1943-1953; age 70-79 | 0.66 | (0.44,0.97) | 0.20 |  | 1.03 | (0.78,1.36) | 0.14 |  |
|  | 1943 or earlier; age 80+ | 0.45 | (0.22,0.93) | 0.37 |  | 0.42 | (0.20,0.89) | 0.38 |  |
| Gender | (Ref: Male) |  |  |  | <.001 |  |  |  | <.001 |
|  | Female | 0.63 | (0.58,0.69) | 0.04 |  | 0.83 | (0.78,0.90) | 0.04 |  |
| Religious affiliation | (Ref: No religion/Atheist/Agnostic) |  |  |  | 0.002 |  |  |  | <.001 |
|  | Islam | 0.80 | (0.56,1.14) | 0.18 |  | 0.76 | (0.58,0.99) | 0.14 |  |
|  | Christianity | 0.87 | (0.61,1.26) | 0.18 |  | 0.83 | (0.63,1.08) | 0.14 |  |
|  | Collapsed affiliations with prevalence<3% | 1.89 | (1.16,3.08) | 0.25 |  | 1.95 | (1.38,2.76) | 0.18 |  |
| Race/ethnicity | (Ref: Plurality group) |  |  |  | 0.374 |  |  |  | 0.521 |
|  | Non-plurality groups | 1.60 | (0.57,4.48) | 0.53 |  | 1.27 | (0.62,2.61) | 0.37 |  |

***Table S19c. Sensitivity to unmeasured confounding of childhood predictors in Tanzania***

|  | | Charitable giving | | Helping | |
| --- | --- | --- | --- | --- | --- |
| Variable | Category | E-value for Estimate | E-value for 95% CI | E-value for Estimate | E-value for 95% CI |
| Relationship with mother | (Ref: Very bad/somewhat bad) |  |  |  |  |
|  | Very good/somewhat good | 1.16 | 1.00 | 1.16 | 1.00 |
| Relationship with father | (Ref: Very bad/somewhat bad) |  |  |  |  |
|  | Very good/somewhat good | 1.26 | 1.00 | 1.22 | 1.00 |
| Parent marital status | (Ref: Parents married) |  |  |  |  |
|  | Divorced | 1.14 | 1.00 | 1.39 | 1.00 |
|  | Parents were never married | 1.19 | 1.00 | 1.13 | 1.00 |
|  | One or both parents had died | 1.10 | 1.00 | 1.27 | 1.00 |
| Subjective financial status of family growing up | (Ref: Got by) |  |  |  |  |
|  | Lived comfortably | 1.38 | 1.00 | 1.16 | 1.00 |
|  | Found it difficult | 1.52 | 1.17 | 1.12 | 1.00 |
|  | Found it very difficult | 1.67 | 1.08 | 1.28 | 1.00 |
| Abuse | (Ref: No) |  |  |  |  |
|  | Yes | 1.78 | 1.36 | 1.52 | 1.14 |
| Outsider growing up | (Ref: No) |  |  |  |  |
|  | Yes | 1.46 | 1.00 | 1.55 | 1.16 |
| Self-rated health growing up | (Ref: Good) |  |  |  |  |
|  | Excellent | 1.66 | 1.35 | 1.71 | 1.44 |
|  | Very good | 1.32 | 1.00 | 1.59 | 1.28 |
|  | Fair | 1.34 | 1.00 | 1.66 | 1.29 |
|  | Poor | 1.51 | 1.00 | 1.91 | 1.39 |
| Immigration status | (Ref: Born in this country) |  |  |  |  |
|  | Born in another country | 3.29 | 1.58 | 2.06 | 1.00 |
| Age 12 religious service attendance | (Ref: Never) |  |  |  |  |
|  | At least 1/week | 2.12 | 1.24 | 2.16 | 1.47 |
|  | 1-3/month | 2.05 | 1.09 | 2.24 | 1.49 |
|  | < 1/month | 1.22 | 1.00 | 1.73 | 1.00 |
| Year of birth | (Ref: 1998-2005; current age: 18-24) |  |  |  |  |
|  | 1993-1998; age 25-29 | 1.69 | 1.28 | 1.30 | 1.00 |
|  | 1983-1993; age 30-39 | 1.27 | 1.00 | 1.33 | 1.00 |
|  | 1973-1983; age 40-49 | 1.19 | 1.00 | 1.50 | 1.12 |
|  | 1963-1973; age 50-59 | 1.22 | 1.00 | 1.75 | 1.37 |
|  | 1953-1963; age 60-69 | 1.22 | 1.00 | 1.65 | 1.06 |
|  | 1943-1953; age 70-79 | 2.42 | 1.22 | 1.21 | 1.00 |
|  | 1943 or earlier; age 80+ | 3.89 | 1.35 | 4.17 | 1.50 |
| Gender | (Ref: Male) |  |  |  |  |
|  | Female | 2.55 | 2.27 | 1.68 | 1.47 |
| Religious affiliation | (Ref: No religion/Atheist/Agnostic) |  |  |  |  |
|  | Islam | 1.82 | 1.00 | 1.98 | 1.12 |
|  | Christianity | 1.55 | 1.00 | 1.72 | 1.00 |
|  | Collapsed affiliations with prevalence<3% | 3.18 | 1.58 | 3.31 | 2.10 |
| Race/ethnicity | (Ref: Plurality group) |  |  |  |  |
|  | Non-plurality groups | 2.58 | 1.00 | 1.85 | 1.00 |

***Table S20a. Nationally representative descriptive statistics for Turkey***

| **Characteristic** | **N = 1,473**^1^ |
| --- | --- |
| **Relationship with mother** |  |
| Very good | 970 (66%) |
| Somewhat good | 401 (27%) |
| Somewhat bad | 48 (3.2%) |
| Very bad | 26 (1.8%) |
| Does not apply | 21 (1.4%) |
| (Missing) | 7 (0.5%) |
| **Relationship with father** |  |
| Very good | 795 (54%) |
| Somewhat good | 425 (29%) |
| Somewhat bad | 73 (5.0%) |
| Very bad | 95 (6.5%) |
| Does not apply | 60 (4.1%) |
| (Missing) | 25 (1.7%) |
| **Parent marital status** |  |
| Parents married | 1,325 (90%) |
| Divorced | 57 (3.9%) |
| Parents were never married | 7 (0.5%) |
| One or both parents had died | 61 (4.1%) |
| (Missing) | 23 (1.5%) |
| **Subjective financial status of family growing up** |  |
| Lived comfortably | 498 (34%) |
| Got by | 647 (44%) |
| Found it difficult | 218 (15%) |
| Found it very difficult | 108 (7.3%) |
| (Missing) | 2 (0.1%) |
| **Abuse** |  |
| Yes | 158 (11%) |
| No | 1,290 (88%) |
| (Missing) | 25 (1.7%) |
| **Outsider growing up** |  |
| Yes | 157 (11%) |
| No | 1,306 (89%) |
| (Missing) | 9 (0.6%) |
| **Self-rated health growing up** |  |
| Excellent | 377 (26%) |
| Very good | 410 (28%) |
| Good | 419 (28%) |
| Fair | 220 (15%) |
| Poor | 47 (3.2%) |
| (Missing) | 0 (<0.1%) |
| **Immigration status** |  |
| Born in this country | 1,415 (96%) |
| Born in another country | 58 (4.0%) |
| (Missing) | 0 (0%) |
| **Age 12 religious service attendance** |  |
| At least 1/week | 609 (41%) |
| 1-3/month | 238 (16%) |
| <1/month | 225 (15%) |
| Never | 383 (26%) |
| (Missing) | 18 (1.2%) |
| **Year of birth** |  |
| 1998-2005; age 18-24 | 222 (15%) |
| 1993-1998; age 25-29 | 152 (10%) |
| 1983-1993; age 30-39 | 315 (21%) |
| 1973-1983; age 40-49 | 312 (21%) |
| 1963-1973; age 50-59 | 225 (15%) |
| 1953-1963; age 60-69 | 164 (11%) |
| 1943-1953; age 70-79 | 65 (4.4%) |
| 1943 or earlier; age 80+ | 18 (1.2%) |
| (Missing) | 0 (0%) |
| **Gender** |  |
| Male | 754 (51%) |
| Female | 719 (49%) |
| Other | 0 (0%) |
| (Missing) | 0 (0%) |
| **Religious affiliation** |  |
| Christianity | 1 (<0.1%) |
| Islam | 1,439 (98%) |
| Hinduism | 0 (0%) |
| Buddhism | 0 (0%) |
| Judaism | 1 (<0.1%) |
| Sikhism | 0 (0%) |
| Baha'i | 0 (0%) |
| Jainism | 0 (0%) |
| Shinto | 0 (0%) |
| Taoism | 0 (0%) |
| Confucianism | 0 (0%) |
| Primal, Animist, or Folk religion | 0 (0%) |
| Spiritism | 0 (0%) |
| Umbanda, Candomble, and other African-derived religions | 0 (0%) |
| Chinese folk/traditional religion | 0 (0%) |
| Some other religion | 0 (0%) |
| No religion/Atheist/Agnostic | 13 (0.9%) |
| (Missing) | 19 (1.3%) |
| **Race/Ethnicity** |  |
| Albanian | 8 (0.5%) |
| Arab | 51 (3.5%) |
| Armenian | 1 (<0.1%) |
| Azeri | 9 (0.6%) |
| Bosnian | 5 (0.3%) |
| Circassian | 19 (1.3%) |
| Georgian | 4 (0.3%) |
| Greek | 1 (<0.1%) |
| Kurdish/Zaza | 252 (17%) |
| Laz | 25 (1.7%) |
| Other | 58 (3.9%) |
| Turkish | 1,030 (70%) |
| Uyghur | 1 (<0.1%) |
| (Missing) | 9 (0.6%) |
| ^1^n (%) | |

***Table S20b. Childhood predictors regression analysis results for Turkey***

|  | | Charitable giving | | | | Helping | | | |
| --- | --- | --- | --- | --- | --- | --- | --- | --- | --- |
| Variable | Category | Risk-Ratio | RR 95% CI | log(RR) SE | Global p-value | Risk-Ratio | RR 95% CI | log(RR) SE | Global p-value |
| Relationship with mother | (Ref: Very bad/somewhat bad) |  |  |  | 0.004 |  |  |  | 0.444 |
|  | Very good/somewhat good | 1.95 | (1.23,3.10) | 0.24 |  | 0.93 | (0.77,1.13) | 0.10 |  |
| Relationship with father | (Ref: Very bad/somewhat bad) |  |  |  | 0.374 |  |  |  | 0.899 |
|  | Very good/somewhat good | 0.89 | (0.67,1.17) | 0.14 |  | 1.01 | (0.87,1.17) | 0.07 |  |
| Parent marital status | (Ref: Parents married) |  |  |  | 0.634 |  |  |  | 0.292 |
|  | Divorced | 0.86 | (0.51,1.46) | 0.27 |  | 1.16 | (0.93,1.43) | 0.11 |  |
|  | Parents were never married | 0.42 | (0.06,2.97) | 1.00 |  | 1.28 | (0.87,1.87) | 0.19 |  |
|  | One or both parents had died | 1.18 | (0.76,1.84) | 0.23 |  | 0.93 | (0.68,1.27) | 0.16 |  |
| Subjective financial status of family growing up | (Ref: Got by) |  |  |  | 0.513 |  |  |  | 0.548 |
|  | Lived comfortably | 1.10 | (0.90,1.34) | 0.10 |  | 0.93 | (0.82,1.05) | 0.06 |  |
|  | Found it difficult | 0.88 | (0.65,1.19) | 0.15 |  | 1.02 | (0.89,1.17) | 0.07 |  |
|  | Found it very difficult | 0.99 | (0.66,1.50) | 0.21 |  | 0.95 | (0.76,1.19) | 0.12 |  |
| Abuse | (Ref: No) |  |  |  | 0.459 |  |  |  | 0.599 |
|  | Yes | 1.12 | (0.83,1.49) | 0.15 |  | 1.04 | (0.89,1.21) | 0.08 |  |
| Outsider growing up | (Ref: No) |  |  |  | 0.832 |  |  |  | 0.771 |
|  | Yes | 1.02 | (0.74,1.40) | 0.16 |  | 1.02 | (0.88,1.19) | 0.08 |  |
| Self-rated health growing up | (Ref: Good) |  |  |  | 0.819 |  |  |  | 0.704 |
|  | Excellent | 1.10 | (0.86,1.40) | 0.12 |  | 0.94 | (0.82,1.08) | 0.07 |  |
|  | Very good | 1.16 | (0.91,1.49) | 0.13 |  | 0.92 | (0.80,1.06) | 0.07 |  |
|  | Fair | 1.05 | (0.77,1.43) | 0.16 |  | 0.99 | (0.85,1.16) | 0.08 |  |
|  | Poor | 0.99 | (0.51,1.93) | 0.34 |  | 0.87 | (0.62,1.21) | 0.17 |  |
| Immigration status | (Ref: Born in this country) |  |  |  | 0.485 |  |  |  | 0.577 |
|  | Born in another country | 0.82 | (0.47,1.44) | 0.29 |  | 1.08 | (0.83,1.39) | 0.13 |  |
| Age 12 religious service attendance | (Ref: Never) |  |  |  | 0.368 |  |  |  | 0.223 |
|  | At least 1/week | 1.16 | (0.90,1.50) | 0.13 |  | 1.09 | (0.94,1.25) | 0.07 |  |
|  | 1-3/month | 1.21 | (0.90,1.62) | 0.15 |  | 1.18 | (1.00,1.38) | 0.08 |  |
|  | < 1/month | 1.29 | (0.96,1.72) | 0.15 |  | 1.12 | (0.94,1.32) | 0.09 |  |
| Year of birth | (Ref: 1998-2005; current age: 18-24) |  |  |  | 0.025 |  |  |  | 0.663 |
|  | 1993-1998; age 25-29 | 1.54 | (1.08,2.18) | 0.18 |  | 1.08 | (0.91,1.29) | 0.09 |  |
|  | 1983-1993; age 30-39 | 1.46 | (1.08,1.98) | 0.15 |  | 1.06 | (0.92,1.23) | 0.07 |  |
|  | 1973-1983; age 40-49 | 1.35 | (0.99,1.84) | 0.16 |  | 1.08 | (0.93,1.25) | 0.07 |  |
|  | 1963-1973; age 50-59 | 1.61 | (1.16,2.24) | 0.17 |  | 1.09 | (0.92,1.29) | 0.09 |  |
|  | 1953-1963; age 60-69 | 1.24 | (0.79,1.94) | 0.23 |  | 1.01 | (0.81,1.26) | 0.11 |  |
|  | 1943-1953; age 70-79 | 2.22 | (1.42,3.49) | 0.23 |  | 0.78 | (0.49,1.24) | 0.24 |  |
|  | 1943 or earlier; age 80+ | 1.85 | (0.67,5.12) | 0.52 |  | 0.41 | (0.09,1.77) | 0.75 |  |
| Gender | (Ref: Male) |  |  |  | 0.087 |  |  |  | 0.200 |
|  | Female | 0.84 | (0.69,1.03) | 0.10 |  | 0.93 | (0.84,1.04) | 0.05 |  |
| Religious affiliation | (Ref: Islam) |  |  |  | 0.047 |  |  |  | 0.672 |
|  | Collapsed affiliations with prevalence<3% | 1.71 | (1.00,2.91) | 0.27 |  | 0.92 | (0.61,1.39) | 0.21 |  |
| Race/ethnicity | (Ref: Plurality group) |  |  |  | 0.004 |  |  |  | 0.661 |
|  | Non-plurality groups | 0.71 | (0.55,0.90) | 0.13 |  | 0.98 | (0.87,1.10) | 0.06 |  |

***Table S20c. Sensitivity to unmeasured confounding of childhood predictors in Turkey***

|  | | Charitable giving | | Helping | |
| --- | --- | --- | --- | --- | --- |
| Variable | Category | E-value for Estimate | E-value for 95% CI | E-value for Estimate | E-value for 95% CI |
| Relationship with mother | (Ref: Very bad/somewhat bad) |  |  |  |  |
|  | Very good/somewhat good | 3.32 | 1.77 | 1.35 | 1.00 |
| Relationship with father | (Ref: Very bad/somewhat bad) |  |  |  |  |
|  | Very good/somewhat good | 1.51 | 1.00 | 1.10 | 1.00 |
| Parent marital status | (Ref: Parents married) |  |  |  |  |
|  | Divorced | 1.59 | 1.00 | 1.58 | 1.00 |
|  | Parents were never married | 4.20 | 1.00 | 1.87 | 1.00 |
|  | One or both parents had died | 1.65 | 1.00 | 1.37 | 1.00 |
| Subjective financial status of family growing up | (Ref: Got by) |  |  |  |  |
|  | Lived comfortably | 1.42 | 1.00 | 1.37 | 1.00 |
|  | Found it difficult | 1.54 | 1.00 | 1.17 | 1.00 |
|  | Found it very difficult | 1.10 | 1.00 | 1.28 | 1.00 |
| Abuse | (Ref: No) |  |  |  |  |
|  | Yes | 1.47 | 1.00 | 1.24 | 1.00 |
| Outsider growing up | (Ref: No) |  |  |  |  |
|  | Yes | 1.17 | 1.00 | 1.17 | 1.00 |
| Self-rated health growing up | (Ref: Good) |  |  |  |  |
|  | Excellent | 1.43 | 1.00 | 1.32 | 1.00 |
|  | Very good | 1.59 | 1.00 | 1.40 | 1.00 |
|  | Fair | 1.28 | 1.00 | 1.08 | 1.00 |
|  | Poor | 1.11 | 1.00 | 1.57 | 1.00 |
| Immigration status | (Ref: Born in this country) |  |  |  |  |
|  | Born in another country | 1.74 | 1.00 | 1.36 | 1.00 |
| Age 12 religious service attendance | (Ref: Never) |  |  |  |  |
|  | At least 1/week | 1.59 | 1.00 | 1.40 | 1.00 |
|  | 1-3/month | 1.71 | 1.00 | 1.63 | 1.06 |
|  | < 1/month | 1.90 | 1.00 | 1.48 | 1.00 |
| Year of birth | (Ref: 1998-2005; current age: 18-24) |  |  |  |  |
|  | 1993-1998; age 25-29 | 2.44 | 1.38 | 1.39 | 1.00 |
|  | 1983-1993; age 30-39 | 2.29 | 1.37 | 1.32 | 1.00 |
|  | 1973-1983; age 40-49 | 2.04 | 1.00 | 1.37 | 1.00 |
|  | 1963-1973; age 50-59 | 2.61 | 1.60 | 1.41 | 1.00 |
|  | 1953-1963; age 60-69 | 1.79 | 1.00 | 1.09 | 1.00 |
|  | 1943-1953; age 70-79 | 3.87 | 2.18 | 1.88 | 1.00 |
|  | 1943 or earlier; age 80+ | 3.11 | 1.00 | 4.36 | 1.00 |
| Gender | (Ref: Male) |  |  |  |  |
|  | Female | 1.65 | 1.00 | 1.35 | 1.00 |
| Religious affiliation | (Ref: Islam) |  |  |  |  |
|  | Collapsed affiliations with prevalence<3% | 2.81 | 1.06 | 1.40 | 1.00 |
| Race/ethnicity | (Ref: Plurality group) |  |  |  |  |
|  | Non-plurality groups | 2.18 | 1.45 | 1.18 | 1.00 |

***Table S21a. Nationally representative descriptive statistics for United Kingdom***

| **Characteristic** | **N = 5,368**^1^ |
| --- | --- |
| **Relationship with mother** |  |
| Very good | 3,435 (64%) |
| Somewhat good | 1,338 (25%) |
| Somewhat bad | 325 (6.1%) |
| Very bad | 150 (2.8%) |
| Does not apply | 92 (1.7%) |
| (Missing) | 27 (0.5%) |
| **Relationship with father** |  |
| Very good | 2,907 (54%) |
| Somewhat good | 1,383 (26%) |
| Somewhat bad | 407 (7.6%) |
| Very bad | 321 (6.0%) |
| Does not apply | 321 (6.0%) |
| (Missing) | 29 (0.5%) |
| **Parent marital status** |  |
| Parents married | 4,343 (81%) |
| Divorced | 481 (9.0%) |
| Parents were never married | 315 (5.9%) |
| One or both parents had died | 154 (2.9%) |
| (Missing) | 75 (1.4%) |
| **Subjective financial status of family growing up** |  |
| Lived comfortably | 2,552 (48%) |
| Got by | 1,933 (36%) |
| Found it difficult | 632 (12%) |
| Found it very difficult | 230 (4.3%) |
| (Missing) | 22 (0.4%) |
| **Abuse** |  |
| Yes | 864 (16%) |
| No | 4,455 (83%) |
| (Missing) | 49 (0.9%) |
| **Outsider growing up** |  |
| Yes | 1,017 (19%) |
| No | 4,308 (80%) |
| (Missing) | 43 (0.8%) |
| **Self-rated health growing up** |  |
| Excellent | 2,154 (40%) |
| Very good | 1,736 (32%) |
| Good | 995 (19%) |
| Fair | 332 (6.2%) |
| Poor | 130 (2.4%) |
| (Missing) | 20 (0.4%) |
| **Immigration status** |  |
| Born in this country | 4,659 (87%) |
| Born in another country | 682 (13%) |
| (Missing) | 27 (0.5%) |
| **Age 12 religious service attendance** |  |
| At least 1/week | 1,732 (32%) |
| 1-3/month | 733 (14%) |
| <1/month | 903 (17%) |
| Never | 1,972 (37%) |
| (Missing) | 28 (0.5%) |
| **Year of birth** |  |
| 1998-2005; age 18-24 | 490 (9.1%) |
| 1993-1998; age 25-29 | 391 (7.3%) |
| 1983-1993; age 30-39 | 946 (18%) |
| 1973-1983; age 40-49 | 827 (15%) |
| 1963-1973; age 50-59 | 949 (18%) |
| 1953-1963; age 60-69 | 889 (17%) |
| 1943-1953; age 70-79 | 711 (13%) |
| 1943 or earlier; age 80+ | 163 (3.0%) |
| (Missing) | 1 (<0.1%) |
| **Gender** |  |
| Male | 2,557 (48%) |
| Female | 2,789 (52%) |
| Other | 14 (0.3%) |
| (Missing) | 9 (0.2%) |
| **Religious affiliation** |  |
| Christianity | 3,461 (64%) |
| Islam | 230 (4.3%) |
| Hinduism | 88 (1.6%) |
| Buddhism | 15 (0.3%) |
| Judaism | 59 (1.1%) |
| Sikhism | 30 (0.6%) |
| Baha'i | 5 (<0.1%) |
| Jainism | 0 (<0.1%) |
| Shinto | 0 (0%) |
| Taoism | 2 (<0.1%) |
| Confucianism | 3 (<0.1%) |
| Primal, Animist, or Folk religion | 22 (0.4%) |
| Spiritism | 0 (0%) |
| Umbanda, Candomble, and other African-derived religions | 0 (0%) |
| Chinese folk/traditional religion | 0 (0%) |
| Some other religion | 24 (0.5%) |
| No religion/Atheist/Agnostic | 1,409 (26%) |
| (Missing) | 21 (0.4%) |
| **Race/Ethnicity** |  |
| Asian | 426 (7.9%) |
| Black | 152 (2.8%) |
| Other | 96 (1.8%) |
| White | 4,647 (87%) |
| (Missing) | 47 (0.9%) |
| ^1^n (%) | |

***Table S21b. Childhood predictors regression analysis results for United Kingdom***

|  | | Charitable giving | | | | Helping | | | |
| --- | --- | --- | --- | --- | --- | --- | --- | --- | --- |
| Variable | Category | Risk-Ratio | RR 95% CI | log(RR) SE | Global p-value | Risk-Ratio | RR 95% CI | log(RR) SE | Global p-value |
| Relationship with mother | (Ref: Very bad/somewhat bad) |  |  |  | 0.052 |  |  |  | 0.281 |
|  | Very good/somewhat good | 1.12 | (1.00,1.26) | 0.06 |  | 1.07 | (0.95,1.20) | 0.06 |  |
| Relationship with father | (Ref: Very bad/somewhat bad) |  |  |  | 0.595 |  |  |  | 0.068 |
|  | Very good/somewhat good | 1.02 | (0.94,1.12) | 0.05 |  | 1.09 | (0.99,1.21) | 0.05 |  |
| Parent marital status | (Ref: Parents married) |  |  |  | 0.178 |  |  |  | <.001 |
|  | Divorced | 0.96 | (0.86,1.07) | 0.06 |  | 1.08 | (0.97,1.21) | 0.05 |  |
|  | Parents were never married | 0.86 | (0.72,1.02) | 0.09 |  | 1.28 | (1.15,1.42) | 0.05 |  |
|  | One or both parents had died | 0.86 | (0.70,1.07) | 0.11 |  | 0.95 | (0.76,1.18) | 0.11 |  |
| Subjective financial status of family growing up | (Ref: Got by) |  |  |  | 0.140 |  |  |  | 0.967 |
|  | Lived comfortably | 1.05 | (0.99,1.13) | 0.03 |  | 0.98 | (0.92,1.06) | 0.04 |  |
|  | Found it difficult | 1.07 | (0.97,1.18) | 0.05 |  | 1.00 | (0.90,1.11) | 0.05 |  |
|  | Found it very difficult | 0.91 | (0.77,1.08) | 0.09 |  | 0.98 | (0.82,1.17) | 0.09 |  |
| Abuse | (Ref: No) |  |  |  | 0.001 |  |  |  | 0.003 |
|  | Yes | 1.13 | (1.05,1.22) | 0.04 |  | 1.13 | (1.04,1.22) | 0.04 |  |
| Outsider growing up | (Ref: No) |  |  |  | 0.114 |  |  |  | <.001 |
|  | Yes | 1.06 | (0.98,1.15) | 0.04 |  | 1.14 | (1.06,1.23) | 0.04 |  |
| Self-rated health growing up | (Ref: Good) |  |  |  | 0.508 |  |  |  | 0.408 |
|  | Excellent | 1.04 | (0.96,1.14) | 0.05 |  | 1.04 | (0.95,1.14) | 0.05 |  |
|  | Very good | 1.07 | (0.98,1.17) | 0.05 |  | 0.98 | (0.89,1.07) | 0.05 |  |
|  | Fair | 0.97 | (0.84,1.13) | 0.08 |  | 0.97 | (0.84,1.12) | 0.07 |  |
|  | Poor | 1.06 | (0.85,1.31) | 0.11 |  | 0.89 | (0.70,1.13) | 0.12 |  |
| Immigration status | (Ref: Born in this country) |  |  |  | 0.002 |  |  |  | <.001 |
|  | Born in another country | 0.85 | (0.77,0.95) | 0.05 |  | 1.23 | (1.14,1.33) | 0.04 |  |
| Age 12 religious service attendance | (Ref: Never) |  |  |  | <.001 |  |  |  | <.001 |
|  | At least 1/week | 1.27 | (1.16,1.38) | 0.04 |  | 1.16 | (1.06,1.28) | 0.05 |  |
|  | 1-3/month | 1.31 | (1.19,1.44) | 0.05 |  | 1.27 | (1.15,1.40) | 0.05 |  |
|  | < 1/month | 1.15 | (1.05,1.27) | 0.05 |  | 1.14 | (1.04,1.26) | 0.05 |  |
| Year of birth | (Ref: 1998-2005; current age: 18-24) |  |  |  | 0.005 |  |  |  | <.001 |
|  | 1993-1998; age 25-29 | 1.09 | (0.92,1.29) | 0.09 |  | 0.85 | (0.75,0.96) | 0.06 |  |
|  | 1983-1993; age 30-39 | 1.08 | (0.93,1.26) | 0.08 |  | 0.89 | (0.80,0.98) | 0.05 |  |
|  | 1973-1983; age 40-49 | 1.18 | (1.01,1.37) | 0.08 |  | 0.84 | (0.75,0.94) | 0.06 |  |
|  | 1963-1973; age 50-59 | 1.03 | (0.88,1.20) | 0.08 |  | 0.82 | (0.73,0.92) | 0.06 |  |
|  | 1953-1963; age 60-69 | 1.07 | (0.91,1.25) | 0.08 |  | 0.64 | (0.56,0.74) | 0.07 |  |
|  | 1943-1953; age 70-79 | 1.15 | (0.98,1.35) | 0.08 |  | 0.54 | (0.46,0.63) | 0.08 |  |
|  | 1943 or earlier; age 80+ | 1.31 | (1.10,1.56) | 0.09 |  | 0.35 | (0.26,0.48) | 0.16 |  |
| Gender | (Ref: Male) |  |  |  | 0.933 |  |  |  | 0.767 |
|  | Female | 1.01 | (0.95,1.07) | 0.03 |  | 0.98 | (0.92,1.05) | 0.03 |  |
|  | Other | 0.87 | (0.38,2.01) | 0.42 |  | 1.05 | (0.78,1.43) | 0.15 |  |
| Religious affiliation | (Ref: No religion/Atheist/Agnostic) |  |  |  | 0.010 |  |  |  | 0.929 |
|  | Islam | 1.27 | (1.09,1.49) | 0.08 |  | 1.00 | (0.86,1.17) | 0.08 |  |
|  | Christianity | 1.01 | (0.93,1.10) | 0.04 |  | 1.03 | (0.94,1.12) | 0.04 |  |
|  | Collapsed affiliations with prevalence<3% | 1.04 | (0.88,1.22) | 0.08 |  | 1.01 | (0.87,1.18) | 0.08 |  |
| Race/ethnicity | (Ref: Plurality group) |  |  |  | 0.577 |  |  |  | 0.644 |
|  | Non-plurality groups | 0.97 | (0.86,1.09) | 0.06 |  | 1.02 | (0.93,1.13) | 0.05 |  |

***Table S21c. Sensitivity to unmeasured confounding of childhood predictors in United Kingdom***

|  | | Charitable giving | | Helping | |
| --- | --- | --- | --- | --- | --- |
| Variable | Category | E-value for Estimate | E-value for 95% CI | E-value for Estimate | E-value for 95% CI |
| Relationship with mother | (Ref: Very bad/somewhat bad) |  |  |  |  |
|  | Very good/somewhat good | 1.49 | 1.00 | 1.33 | 1.00 |
| Relationship with father | (Ref: Very bad/somewhat bad) |  |  |  |  |
|  | Very good/somewhat good | 1.18 | 1.00 | 1.41 | 1.00 |
| Parent marital status | (Ref: Parents married) |  |  |  |  |
|  | Divorced | 1.26 | 1.00 | 1.38 | 1.00 |
|  | Parents were never married | 1.61 | 1.00 | 1.87 | 1.56 |
|  | One or both parents had died | 1.59 | 1.00 | 1.30 | 1.00 |
| Subjective financial status of family growing up | (Ref: Got by) |  |  |  |  |
|  | Lived comfortably | 1.29 | 1.00 | 1.14 | 1.00 |
|  | Found it difficult | 1.35 | 1.00 | 1.01 | 1.00 |
|  | Found it very difficult | 1.42 | 1.00 | 1.17 | 1.00 |
| Abuse | (Ref: No) |  |  |  |  |
|  | Yes | 1.52 | 1.28 | 1.51 | 1.24 |
| Outsider growing up | (Ref: No) |  |  |  |  |
|  | Yes | 1.32 | 1.00 | 1.55 | 1.31 |
| Self-rated health growing up | (Ref: Good) |  |  |  |  |
|  | Excellent | 1.26 | 1.00 | 1.24 | 1.00 |
|  | Very good | 1.34 | 1.00 | 1.17 | 1.00 |
|  | Fair | 1.19 | 1.00 | 1.22 | 1.00 |
|  | Poor | 1.31 | 1.00 | 1.49 | 1.00 |
| Immigration status | (Ref: Born in this country) |  |  |  |  |
|  | Born in another country | 1.62 | 1.30 | 1.77 | 1.55 |
| Age 12 religious service attendance | (Ref: Never) |  |  |  |  |
|  | At least 1/week | 1.85 | 1.60 | 1.60 | 1.32 |
|  | 1-3/month | 1.95 | 1.66 | 1.85 | 1.56 |
|  | < 1/month | 1.58 | 1.27 | 1.54 | 1.23 |
| Year of birth | (Ref: 1998-2005; current age: 18-24) |  |  |  |  |
|  | 1993-1998; age 25-29 | 1.39 | 1.00 | 1.64 | 1.25 |
|  | 1983-1993; age 30-39 | 1.38 | 1.00 | 1.51 | 1.15 |
|  | 1973-1983; age 40-49 | 1.63 | 1.13 | 1.66 | 1.32 |
|  | 1963-1973; age 50-59 | 1.21 | 1.00 | 1.74 | 1.40 |
|  | 1953-1963; age 60-69 | 1.33 | 1.00 | 2.49 | 2.06 |
|  | 1943-1953; age 70-79 | 1.57 | 1.00 | 3.13 | 2.55 |
|  | 1943 or earlier; age 80+ | 1.95 | 1.43 | 5.08 | 3.55 |
| Gender | (Ref: Male) |  |  |  |  |
|  | Female | 1.08 | 1.00 | 1.16 | 1.00 |
|  | Other | 1.55 | 1.00 | 1.30 | 1.00 |
| Religious affiliation | (Ref: No religion/Atheist/Agnostic) |  |  |  |  |
|  | Islam | 1.86 | 1.41 | 1.06 | 1.00 |
|  | Christianity | 1.10 | 1.00 | 1.19 | 1.00 |
|  | Collapsed affiliations with prevalence<3% | 1.24 | 1.00 | 1.12 | 1.00 |
| Race/ethnicity | (Ref: Plurality group) |  |  |  |  |
|  | Non-plurality groups | 1.22 | 1.00 | 1.17 | 1.00 |

***Table S22a. Nationally representative descriptive statistics for United States***

| **Characteristic** | **N = 38,312**^1^ |
| --- | --- |
| **Relationship with mother** |  |
| Very good | 20,590 (54%) |
| Somewhat good | 11,525 (30%) |
| Somewhat bad | 3,523 (9.2%) |
| Very bad | 1,874 (4.9%) |
| Does not apply | 694 (1.8%) |
| (Missing) | 106 (0.3%) |
| **Relationship with father** |  |
| Very good | 15,313 (40%) |
| Somewhat good | 12,665 (33%) |
| Somewhat bad | 4,879 (13%) |
| Very bad | 2,604 (6.8%) |
| Does not apply | 2,811 (7.3%) |
| (Missing) | 38 (0.1%) |
| **Parent marital status** |  |
| Parents married | 27,415 (72%) |
| Divorced | 6,325 (17%) |
| Parents were never married | 3,048 (8.0%) |
| One or both parents had died | 1,024 (2.7%) |
| (Missing) | 500 (1.3%) |
| **Subjective financial status of family growing up** |  |
| Lived comfortably | 15,116 (39%) |
| Got by | 15,682 (41%) |
| Found it difficult | 5,152 (13%) |
| Found it very difficult | 2,342 (6.1%) |
| (Missing) | 19 (<0.1%) |
| **Abuse** |  |
| Yes | 10,026 (26%) |
| No | 28,045 (73%) |
| (Missing) | 242 (0.6%) |
| **Outsider growing up** |  |
| Yes | 10,185 (27%) |
| No | 27,714 (72%) |
| (Missing) | 413 (1.1%) |
| **Self-rated health growing up** |  |
| Excellent | 16,866 (44%) |
| Very good | 12,108 (32%) |
| Good | 6,444 (17%) |
| Fair | 2,303 (6.0%) |
| Poor | 520 (1.4%) |
| (Missing) | 71 (0.2%) |
| **Immigration status** |  |
| Born in this country | 34,865 (91%) |
| Born in another country | 3,020 (7.9%) |
| (Missing) | 427 (1.1%) |
| **Age 12 religious service attendance** |  |
| At least 1/week | 18,609 (49%) |
| 1-3/month | 6,644 (17%) |
| <1/month | 5,829 (15%) |
| Never | 7,085 (18%) |
| (Missing) | 145 (0.4%) |
| **Year of birth** |  |
| 1998-2005; age 18-24 | 2,682 (7.0%) |
| 1993-1998; age 25-29 | 3,540 (9.2%) |
| 1983-1993; age 30-39 | 7,284 (19%) |
| 1973-1983; age 40-49 | 5,649 (15%) |
| 1963-1973; age 50-59 | 6,745 (18%) |
| 1953-1963; age 60-69 | 6,832 (18%) |
| 1943-1953; age 70-79 | 4,054 (11%) |
| 1943 or earlier; age 80+ | 1,525 (4.0%) |
| (Missing) | 0 (0%) |
| **Gender** |  |
| Male | 18,222 (48%) |
| Female | 19,562 (51%) |
| Other | 392 (1.0%) |
| (Missing) | 136 (0.4%) |
| **Religious affiliation** |  |
| Christianity | 30,444 (79%) |
| Islam | 220 (0.6%) |
| Hinduism | 203 (0.5%) |
| Buddhism | 172 (0.4%) |
| Judaism | 787 (2.1%) |
| Sikhism | 47 (0.1%) |
| Baha'i | 4 (<0.1%) |
| Jainism | 18 (<0.1%) |
| Shinto | 6 (<0.1%) |
| Taoism | 17 (<0.1%) |
| Confucianism | 8 (<0.1%) |
| Primal, Animist, or Folk religion | 67 (0.2%) |
| Spiritism | 0 (0%) |
| Umbanda, Candomble, and other African-derived religions | 0 (0%) |
| Chinese folk/traditional religion | 0 (0%) |
| Some other religion | 359 (0.9%) |
| No religion/Atheist/Agnostic | 5,845 (15%) |
| (Missing) | 115 (0.3%) |
| **Race/Ethnicity** |  |
| Asian | 2,466 (6.4%) |
| Black | 4,501 (12%) |
| Hispanic | 6,724 (18%) |
| Other | 997 (2.6%) |
| White | 23,605 (62%) |
| (Missing) | 20 (<0.1%) |
| ^1^n (%) | |

***Table S22b. Childhood predictors regression analysis results for United States***

|  | | Charitable giving | | | | Helping | | | |
| --- | --- | --- | --- | --- | --- | --- | --- | --- | --- |
| Variable | Category | Risk-Ratio | RR 95% CI | log(RR) SE | Global p-value | Risk-Ratio | RR 95% CI | log(RR) SE | Global p-value |
| Relationship with mother | (Ref: Very bad/somewhat bad) |  |  |  | 0.450 |  |  |  | 0.914 |
|  | Very good/somewhat good | 1.03 | (0.95,1.13) | 0.04 |  | 1.00 | (0.94,1.06) | 0.03 |  |
| Relationship with father | (Ref: Very bad/somewhat bad) |  |  |  | 0.214 |  |  |  | 0.454 |
|  | Very good/somewhat good | 0.96 | (0.89,1.03) | 0.04 |  | 1.02 | (0.97,1.08) | 0.03 |  |
| Parent marital status | (Ref: Parents married) |  |  |  | 0.118 |  |  |  | 0.532 |
|  | Divorced | 0.92 | (0.85,0.99) | 0.04 |  | 1.04 | (0.98,1.10) | 0.03 |  |
|  | Parents were never married | 1.04 | (0.87,1.24) | 0.09 |  | 1.05 | (0.95,1.16) | 0.05 |  |
|  | One or both parents had died | 1.04 | (0.88,1.22) | 0.08 |  | 1.04 | (0.89,1.21) | 0.08 |  |
| Subjective financial status of family growing up | (Ref: Got by) |  |  |  | 0.260 |  |  |  | 0.019 |
|  | Lived comfortably | 1.00 | (0.96,1.05) | 0.02 |  | 1.00 | (0.96,1.05) | 0.02 |  |
|  | Found it difficult | 1.08 | (1.00,1.17) | 0.04 |  | 1.04 | (0.97,1.11) | 0.03 |  |
|  | Found it very difficult | 1.06 | (0.93,1.20) | 0.07 |  | 1.13 | (1.04,1.21) | 0.04 |  |
| Abuse | (Ref: No) |  |  |  | 0.142 |  |  |  | <.001 |
|  | Yes | 1.05 | (0.98,1.11) | 0.03 |  | 1.14 | (1.09,1.19) | 0.02 |  |
| Outsider growing up | (Ref: No) |  |  |  | 0.037 |  |  |  | <.001 |
|  | Yes | 0.93 | (0.86,1.00) | 0.04 |  | 1.09 | (1.04,1.15) | 0.02 |  |
| Self-rated health growing up | (Ref: Good) |  |  |  | 0.035 |  |  |  | 0.762 |
|  | Excellent | 1.11 | (1.03,1.20) | 0.04 |  | 1.03 | (0.97,1.09) | 0.03 |  |
|  | Very good | 1.09 | (1.00,1.18) | 0.04 |  | 1.00 | (0.94,1.07) | 0.03 |  |
|  | Fair | 0.93 | (0.78,1.12) | 0.09 |  | 0.99 | (0.88,1.11) | 0.06 |  |
|  | Poor | 1.09 | (0.82,1.46) | 0.15 |  | 1.02 | (0.84,1.23) | 0.10 |  |
| Immigration status | (Ref: Born in this country) |  |  |  | 0.905 |  |  |  | 0.795 |
|  | Born in another country | 1.00 | (0.89,1.11) | 0.06 |  | 0.99 | (0.91,1.07) | 0.04 |  |
| Age 12 religious service attendance | (Ref: Never) |  |  |  | <.001 |  |  |  | 0.020 |
|  | At least 1/week | 1.36 | (1.24,1.49) | 0.05 |  | 1.10 | (1.03,1.17) | 0.03 |  |
|  | 1-3/month | 1.23 | (1.11,1.36) | 0.05 |  | 1.06 | (0.99,1.15) | 0.04 |  |
|  | < 1/month | 1.19 | (1.07,1.32) | 0.06 |  | 1.04 | (0.96,1.12) | 0.04 |  |
| Year of birth | (Ref: 1998-2005; current age: 18-24) |  |  |  | <.001 |  |  |  | <.001 |
|  | 1993-1998; age 25-29 | 1.41 | (1.06,1.87) | 0.14 |  | 1.01 | (0.89,1.16) | 0.07 |  |
|  | 1983-1993; age 30-39 | 1.44 | (1.11,1.86) | 0.13 |  | 0.97 | (0.86,1.09) | 0.06 |  |
|  | 1973-1983; age 40-49 | 1.74 | (1.35,2.24) | 0.13 |  | 0.96 | (0.85,1.08) | 0.06 |  |
|  | 1963-1973; age 50-59 | 1.87 | (1.46,2.41) | 0.13 |  | 0.95 | (0.84,1.07) | 0.06 |  |
|  | 1953-1963; age 60-69 | 2.01 | (1.57,2.58) | 0.13 |  | 0.89 | (0.80,1.00) | 0.06 |  |
|  | 1943-1953; age 70-79 | 2.15 | (1.68,2.76) | 0.13 |  | 0.75 | (0.67,0.85) | 0.06 |  |
|  | 1943 or earlier; age 80+ | 2.19 | (1.69,2.82) | 0.13 |  | 0.61 | (0.52,0.72) | 0.08 |  |
| Gender | (Ref: Male) |  |  |  | 0.001 |  |  |  | 0.465 |
|  | Female | 1.09 | (1.04,1.14) | 0.02 |  | 1.02 | (0.98,1.06) | 0.02 |  |
|  | Other | 1.28 | (0.85,1.93) | 0.21 |  | 0.98 | (0.77,1.24) | 0.12 |  |
| Religious affiliation | (Ref: No religion/Atheist/Agnostic) |  |  |  | 0.005 |  |  |  | 0.686 |
|  | Christianity | 0.94 | (0.85,1.04) | 0.05 |  | 1.03 | (0.95,1.11) | 0.04 |  |
|  | Collapsed affiliations with prevalence<3% | 1.10 | (0.96,1.26) | 0.07 |  | 1.00 | (0.89,1.12) | 0.06 |  |
| Race/ethnicity | (Ref: Plurality group) |  |  |  | 0.003 |  |  |  | <.001 |
|  | Non-plurality groups | 0.92 | (0.87,0.97) | 0.03 |  | 1.09 | (1.05,1.14) | 0.02 |  |

***Table S22c. Sensitivity to unmeasured confounding of childhood predictors in United States***

|  | | Charitable giving | | Helping | |
| --- | --- | --- | --- | --- | --- |
| Variable | Category | E-value for Estimate | E-value for 95% CI | E-value for Estimate | E-value for 95% CI |
| Relationship with mother | (Ref: Very bad/somewhat bad) |  |  |  |  |
|  | Very good/somewhat good | 1.22 | 1.00 | 1.01 | 1.00 |
| Relationship with father | (Ref: Very bad/somewhat bad) |  |  |  |  |
|  | Very good/somewhat good | 1.26 | 1.00 | 1.17 | 1.00 |
| Parent marital status | (Ref: Parents married) |  |  |  |  |
|  | Divorced | 1.40 | 1.10 | 1.23 | 1.00 |
|  | Parents were never married | 1.24 | 1.00 | 1.27 | 1.00 |
|  | One or both parents had died | 1.24 | 1.00 | 1.25 | 1.00 |
| Subjective financial status of family growing up | (Ref: Got by) |  |  |  |  |
|  | Lived comfortably | 1.07 | 1.00 | 1.07 | 1.00 |
|  | Found it difficult | 1.37 | 1.00 | 1.25 | 1.00 |
|  | Found it very difficult | 1.31 | 1.00 | 1.50 | 1.26 |
| Abuse | (Ref: No) |  |  |  |  |
|  | Yes | 1.27 | 1.00 | 1.54 | 1.41 |
| Outsider growing up | (Ref: No) |  |  |  |  |
|  | Yes | 1.37 | 1.07 | 1.42 | 1.26 |
| Self-rated health growing up | (Ref: Good) |  |  |  |  |
|  | Excellent | 1.46 | 1.19 | 1.19 | 1.00 |
|  | Very good | 1.39 | 1.01 | 1.07 | 1.00 |
|  | Fair | 1.35 | 1.00 | 1.12 | 1.00 |
|  | Poor | 1.42 | 1.00 | 1.14 | 1.00 |
| Immigration status | (Ref: Born in this country) |  |  |  |  |
|  | Born in another country | 1.06 | 1.00 | 1.11 | 1.00 |
| Age 12 religious service attendance | (Ref: Never) |  |  |  |  |
|  | At least 1/week | 2.06 | 1.79 | 1.43 | 1.20 |
|  | 1-3/month | 1.75 | 1.45 | 1.32 | 1.00 |
|  | < 1/month | 1.66 | 1.33 | 1.23 | 1.00 |
| Year of birth | (Ref: 1998-2005; current age: 18-24) |  |  |  |  |
|  | 1993-1998; age 25-29 | 2.16 | 1.31 | 1.14 | 1.00 |
|  | 1983-1993; age 30-39 | 2.24 | 1.47 | 1.20 | 1.00 |
|  | 1973-1983; age 40-49 | 2.88 | 2.04 | 1.26 | 1.00 |
|  | 1963-1973; age 50-59 | 3.16 | 2.28 | 1.29 | 1.00 |
|  | 1953-1963; age 60-69 | 3.44 | 2.52 | 1.49 | 1.00 |
|  | 1943-1953; age 70-79 | 3.73 | 2.75 | 1.98 | 1.63 |
|  | 1943 or earlier; age 80+ | 3.80 | 2.78 | 2.64 | 2.11 |
| Gender | (Ref: Male) |  |  |  |  |
|  | Female | 1.39 | 1.23 | 1.18 | 1.00 |
|  | Other | 1.88 | 1.00 | 1.18 | 1.00 |
| Religious affiliation | (Ref: No religion/Atheist/Agnostic) |  |  |  |  |
|  | Christianity | 1.32 | 1.00 | 1.20 | 1.00 |
|  | Collapsed affiliations with prevalence<3% | 1.44 | 1.00 | 1.04 | 1.00 |
| Race/ethnicity | (Ref: Plurality group) |  |  |  |  |
|  | Non-plurality groups | 1.40 | 1.20 | 1.41 | 1.26 |

***Table S23a. Population weighted meta-analysis of regression results – charitable giving***

| Variable | Category | RR | 95% CI |
| --- | --- | --- | --- |
| Relationship with mother | (Ref: Very bad/somewhat bad) |  |  |
|  | Very good/somewhat good | 1.11 | (1.01,1.21) |
| Relationship with father | (Ref: Very bad/somewhat bad) |  |  |
|  | Very good/somewhat good | 1.04 | (0.96,1.13) |
| Parent marital status | (Ref: Parents married) |  |  |
|  | Divorced | 1.06 | (0.99,1.14) |
|  | Single, never married | 1.00 | (0.93,1.07) |
|  | One or both parents had died | 1.00 | (0.95,1.06) |
| Subjective financial status of family growing up | (Ref: Got by) |  |  |
|  | Lived comfortably | 1.05 | (1.02,1.09) |
|  | Found it difficult | 0.97 | (0.93,1.01) |
|  | Found it very difficult | 0.97 | (0.92,1.02) |
| Abuse | (Ref: No) |  |  |
|  | Yes | 1.12 | (1.08,1.16) |
| Outsider growing up | (Ref: No) |  |  |
|  | Yes | 1.20 | (1.15,1.25) |
| Self-rated health growing up | (Ref: Good) |  |  |
|  | Excellent | 1.11 | (1.06,1.16) |
|  | Very good | 1.13 | (1.08,1.17) |
|  | Fair | 1.05 | (0.99,1.10) |
|  | Poor | 1.19 | (1.10,1.29) |
| Immigration status | (Ref: Born in this country) |  |  |
|  | Born in another country | 0.74 | (0.63,0.86) |
| Age 12 religious service attendance | (Ref: Never) |  |  |
|  | At least 1/week | 1.36 | (1.28,1.44) |
|  | 1-3/month | 1.30 | (1.23,1.39) |
|  | < 1/month | 1.13 | (1.06,1.20) |
| Year of birth | (Ref: 1998-2005; age 18-24) |  |  |
|  | 1993-1998; age 25-29 | 1.19 | (1.12,1.26) |
|  | 1983-1993; age 30-39 | 1.19 | (1.13,1.25) |
|  | 1973-1983; age 40-49 | 1.25 | (1.19,1.32) |
|  | 1963-1973; age 50-59 | 1.25 | (1.18,1.32) |
|  | 1953-1963; age 60-69 | 1.32 | (1.24,1.41) |
|  | 1943-1953; age 70-79 | 1.39 | (1.27,1.51) |
|  | 1943 or earlier; age 80+ | 1.61 | (1.40,1.86) |
| Gender | (Ref: Male) |  |  |
|  | Female | 0.97 | (0.94,1.00) |
|  | Other | 0.42 | (0.34,0.52) |

Table S23b. Population weighted meta-analysis of regression results - helping

| Variable | Category | RR | 95% CI |
| --- | --- | --- | --- |
| Relationship with mother | (Ref: Very bad/somewhat bad) |  |  |
|  | Very good/somewhat good | 1.03 | (0.96,1.11) |
| Relationship with father | (Ref: Very bad/somewhat bad) |  |  |
|  | Very good/somewhat good | 1.00 | (0.94,1.06) |
| Parent marital status | (Ref: Parents married) |  |  |
|  | Divorced | 1.02 | (0.96,1.08) |
|  | Single, never married | 1.03 | (0.99,1.08) |
|  | One or both parents had died | 1.01 | (0.97,1.06) |
| Subjective financial status of family growing up | (Ref: Got by) |  |  |
|  | Lived comfortably | 1.04 | (1.01,1.06) |
|  | Found it difficult | 1.01 | (0.98,1.05) |
|  | Found it very difficult | 1.01 | (0.96,1.05) |
| Abuse | (Ref: No) |  |  |
|  | Yes | 1.11 | (1.08,1.14) |
| Outsider growing up | (Ref: No) |  |  |
|  | Yes | 1.13 | (1.10,1.16) |
| Self-rated health growing up | (Ref: Good) |  |  |
|  | Excellent | 1.08 | (1.05,1.12) |
|  | Very good | 1.03 | (1.01,1.06) |
|  | Fair | 1.01 | (0.97,1.04) |
|  | Poor | 1.06 | (1.00,1.12) |
| Immigration status | (Ref: Born in this country) |  |  |
|  | Born in another country | 1.07 | (0.98,1.18) |
| Age 12 religious service attendance | (Ref: Never) |  |  |
|  | At least 1/week | 1.22 | (1.17,1.27) |
|  | 1-3/month | 1.19 | (1.14,1.24) |
|  | < 1/month | 1.11 | (1.06,1.15) |
| Year of birth | (Ref: 1998-2005; age 18-24) |  |  |
|  | 1993-1998; age 25-29 | 1.00 | (0.96,1.03) |
|  | 1983-1993; age 30-39 | 1.00 | (0.97,1.03) |
|  | 1973-1983; age 40-49 | 0.99 | (0.96,1.02) |
|  | 1963-1973; age 50-59 | 0.95 | (0.91,0.98) |
|  | 1953-1963; age 60-69 | 0.88 | (0.84,0.92) |
|  | 1943-1953; age 70-79 | 0.79 | (0.73,0.86) |
|  | 1943 or earlier; age 80+ | 0.78 | (0.66,0.91) |
| Gender | (Ref: Male) |  |  |
|  | Female | 0.92 | (0.90,0.94) |
|  | Other | 0.69 | (0.56,0.85) |

***Table S24a. Population weighted meta-analysis of E-values – charitable giving***

| Variable | Category | *E*-value for Estimate | *E*-value for 95% CI |
| --- | --- | --- | --- |
| Relationship with mother | (Ref: Very bad/somewhat bad) |  |  |
|  | Very good/somewhat good | 1.45 | 1.11 |
| Relationship with father | (Ref: Very bad/somewhat bad) |  |  |
|  | Very good/somewhat good | 1.25 | 1.00 |
| Parent marital status | (Ref: Parents married) |  |  |
|  | Divorced | 1.31 | 1.00 |
|  | Single, never married | 1.07 | 1.00 |
|  | One or both parents had died | 1.05 | 1.00 |
| Subjective financial status of family growing up | (Ref: Got by) |  |  |
|  | Lived comfortably | 1.29 | 1.16 |
|  | Found it difficult | 1.22 | 1.00 |
|  | Found it very difficult | 1.22 | 1.00 |
| Abuse | (Ref: No) |  |  |
|  | Yes | 1.49 | 1.37 |
| Outsider growing up | (Ref: No) |  |  |
|  | Yes | 1.69 | 1.58 |
| Self-rated health growing up | (Ref: Good) |  |  |
|  | Excellent | 1.46 | 1.31 |
|  | Very good | 1.50 | 1.38 |
|  | Fair | 1.26 | 1.00 |
|  | Poor | 1.67 | 1.44 |
| Immigration status | (Ref: Born in this country) |  |  |
|  | Born in another country | 2.05 | 1.60 |
| Age 12 religious service attendance | (Ref: Never) |  |  |
|  | At least 1/week | 2.06 | 1.88 |
|  | 1-3/month | 1.93 | 1.75 |
|  | < 1/month | 1.51 | 1.32 |
| Year of birth | (Ref: 1998-2005; age 18-24) |  |  |
|  | 1993-1998; age 25-29 | 1.66 | 1.49 |
|  | 1983-1993; age 30-39 | 1.67 | 1.52 |
|  | 1973-1983; age 40-49 | 1.82 | 1.67 |
|  | 1963-1973; age 50-59 | 1.80 | 1.64 |
|  | 1953-1963; age 60-69 | 1.98 | 1.79 |
|  | 1943-1953; age 70-79 | 2.12 | 1.86 |
|  | 1943 or earlier; age 80+ | 2.60 | 2.15 |
| Gender | (Ref: Male) |  |  |
|  | Female | 1.21 | 1.07 |
|  | Other | 4.23 | 3.28 |

***Table S24b. Population weighted meta-analysis of E-values - helping***

| Variable | Category | *E*-value for Estimate | *E*-value for 95% CI |
| --- | --- | --- | --- |
| Relationship with mother | (Ref: Very bad/somewhat bad) |  |  |
|  | Very good/somewhat good | 1.22 | 1.00 |
| Relationship with father | (Ref: Very bad/somewhat bad) |  |  |
|  | Very good/somewhat good | 1.07 | 1.00 |
| Parent marital status | (Ref: Parents married) |  |  |
|  | Divorced | 1.16 | 1.00 |
|  | Single, never married | 1.22 | 1.00 |
|  | One or both parents had died | 1.13 | 1.00 |
| Subjective financial status of family growing up | (Ref: Got by) |  |  |
|  | Lived comfortably | 1.23 | 1.11 |
|  | Found it difficult | 1.13 | 1.00 |
|  | Found it very difficult | 1.08 | 1.00 |
| Abuse | (Ref: No) |  |  |
|  | Yes | 1.46 | 1.39 |
| Outsider growing up | (Ref: No) |  |  |
|  | Yes | 1.51 | 1.43 |
| Self-rated health growing up | (Ref: Good) |  |  |
|  | Excellent | 1.39 | 1.28 |
|  | Very good | 1.22 | 1.09 |
|  | Fair | 1.10 | 1.00 |
|  | Poor | 1.32 | 1.07 |
| Immigration status | (Ref: Born in this country) |  |  |
|  | Born in another country | 1.35 | 1.00 |
| Age 12 religious service attendance | (Ref: Never) |  |  |
|  | At least 1/week | 1.73 | 1.62 |
|  | 1-3/month | 1.66 | 1.53 |
|  | < 1/month | 1.45 | 1.31 |
| Year of birth | (Ref: 1998-2005; age 18-24) |  |  |
|  | 1993-1998; age 25-29 | 1.07 | 1.00 |
|  | 1983-1993; age 30-39 | 1.02 | 1.00 |
|  | 1973-1983; age 40-49 | 1.13 | 1.00 |
|  | 1963-1973; age 50-59 | 1.30 | 1.14 |
|  | 1953-1963; age 60-69 | 1.53 | 1.39 |
|  | 1943-1953; age 70-79 | 1.83 | 1.60 |
|  | 1943 or earlier; age 80+ | 1.90 | 1.43 |
| Gender | (Ref: Male) |  |  |
|  | Female | 1.40 | 1.34 |
|  | Other | 2.25 | 1.63 |

|  | **Charitable Giving** | **Helping** |  |
| --- | --- | --- | --- |
| ***Figure S1. Forest plot for ‘Relationship with mother’ – ‘Very good/somewhat good’ effect*** | 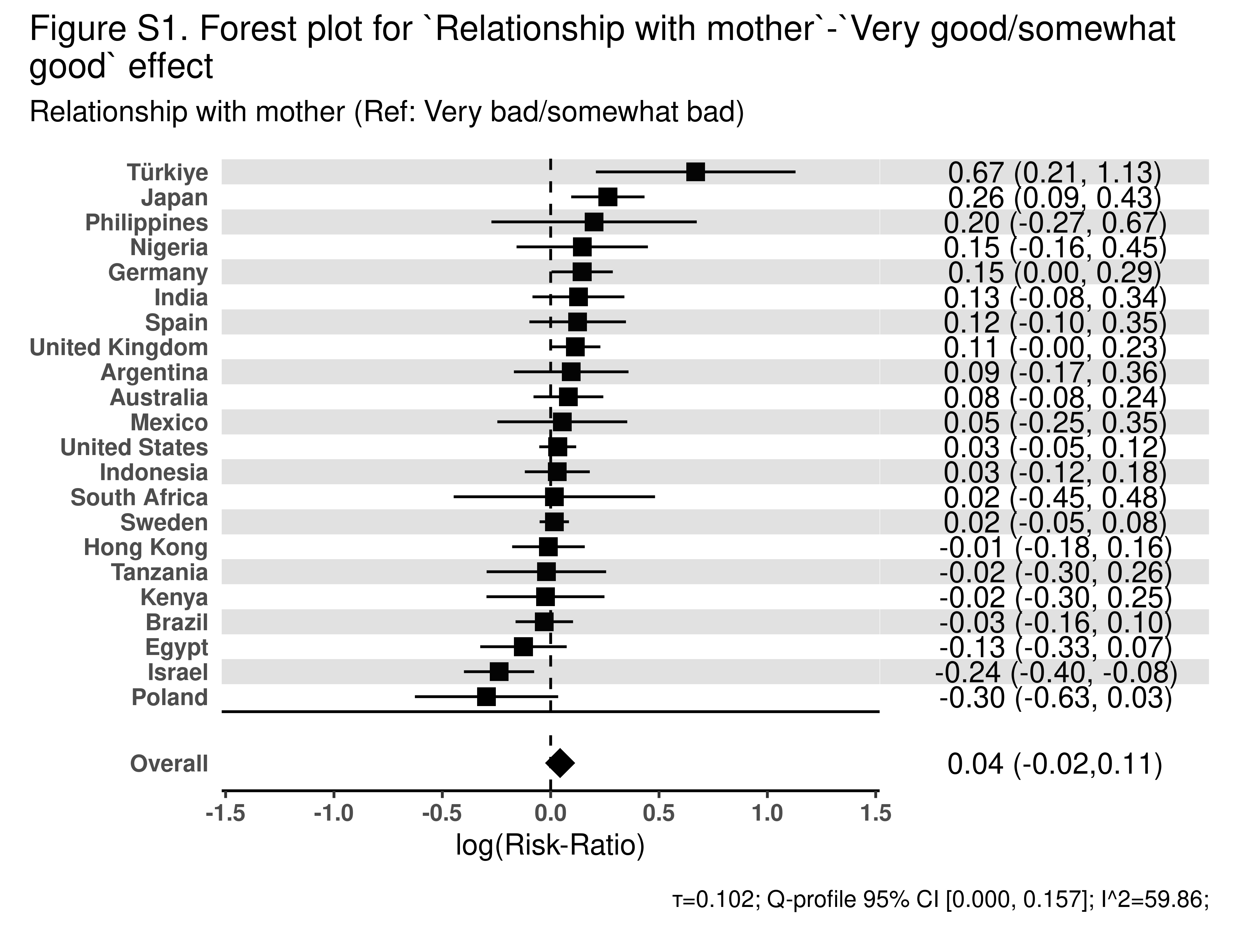 | 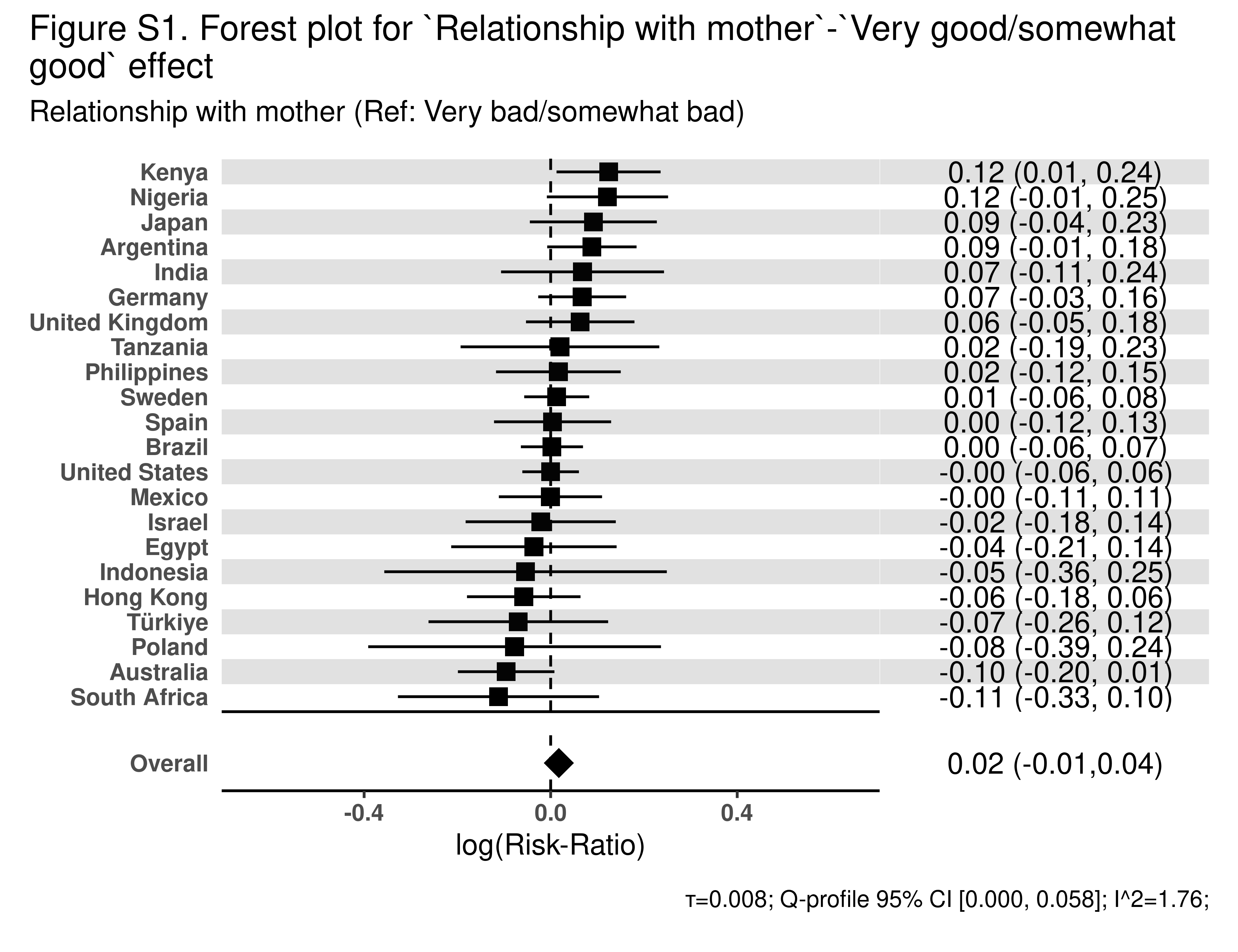 |  |
| ***Figure S2. Forest plot for ‘Relationship with father’ – ‘Very good/somewhat good’ effect*** | 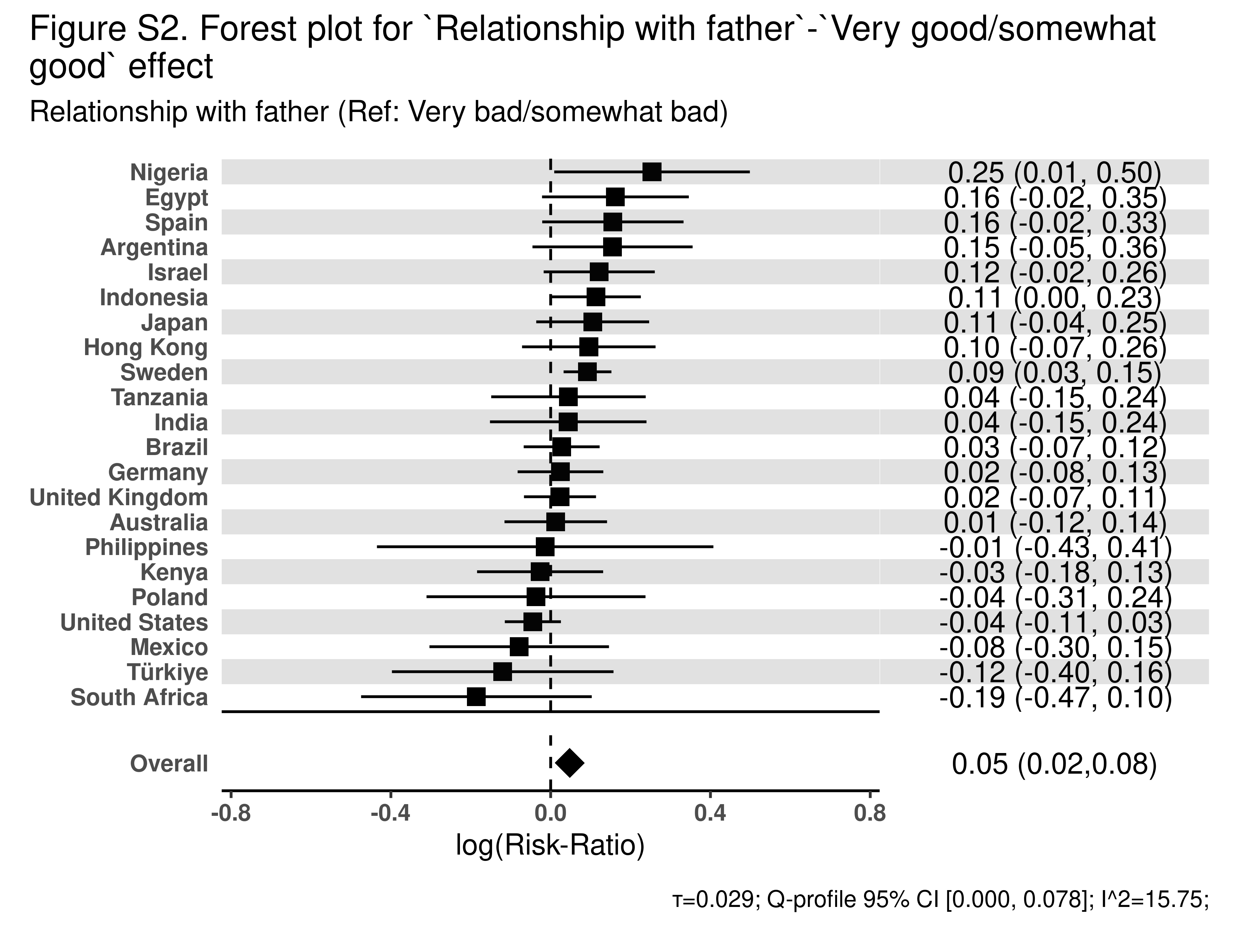 | 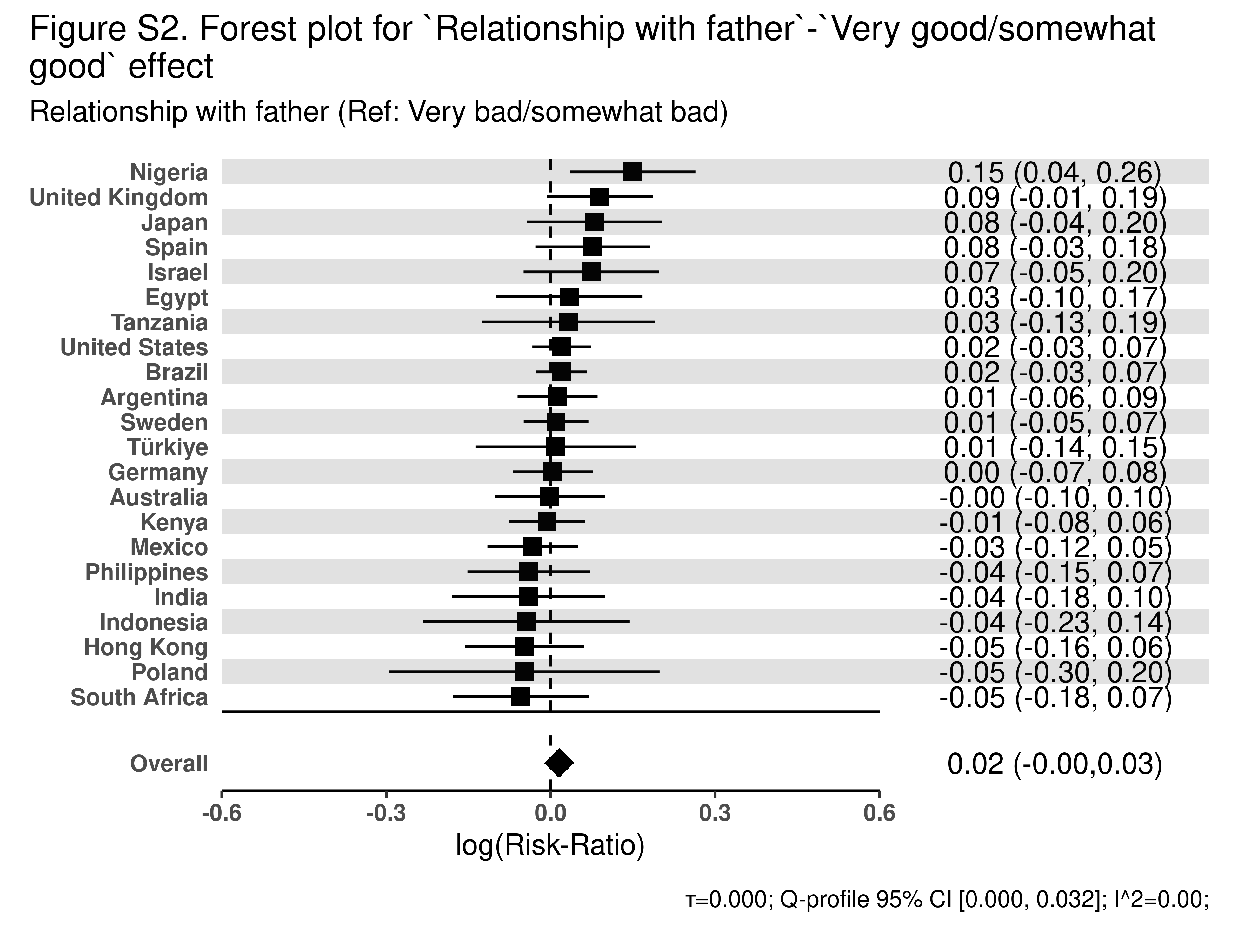 |  |
| ***Figure S3. Forest plot for ‘Parent marital status’ – ‘Divorced’ effect*** | 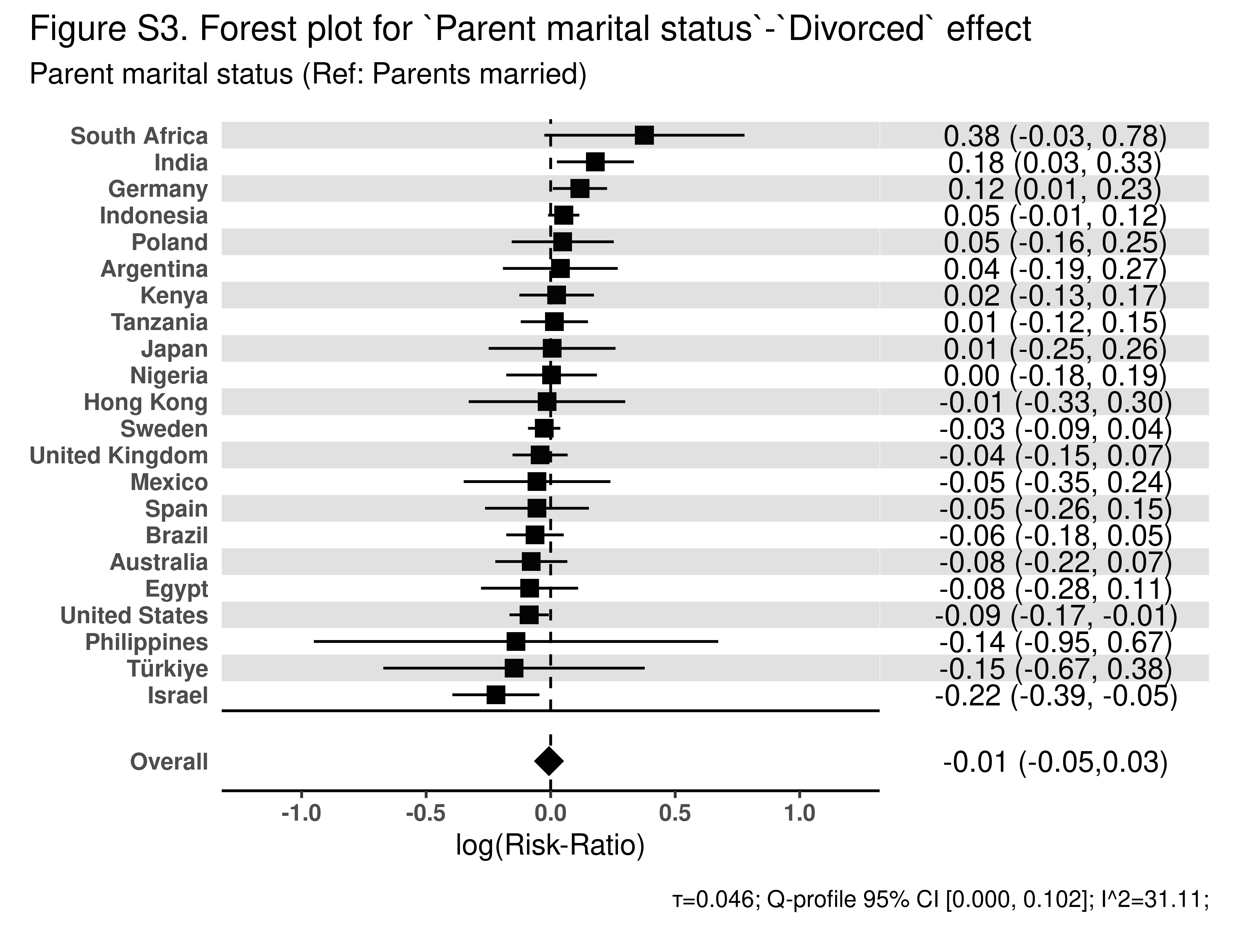 | 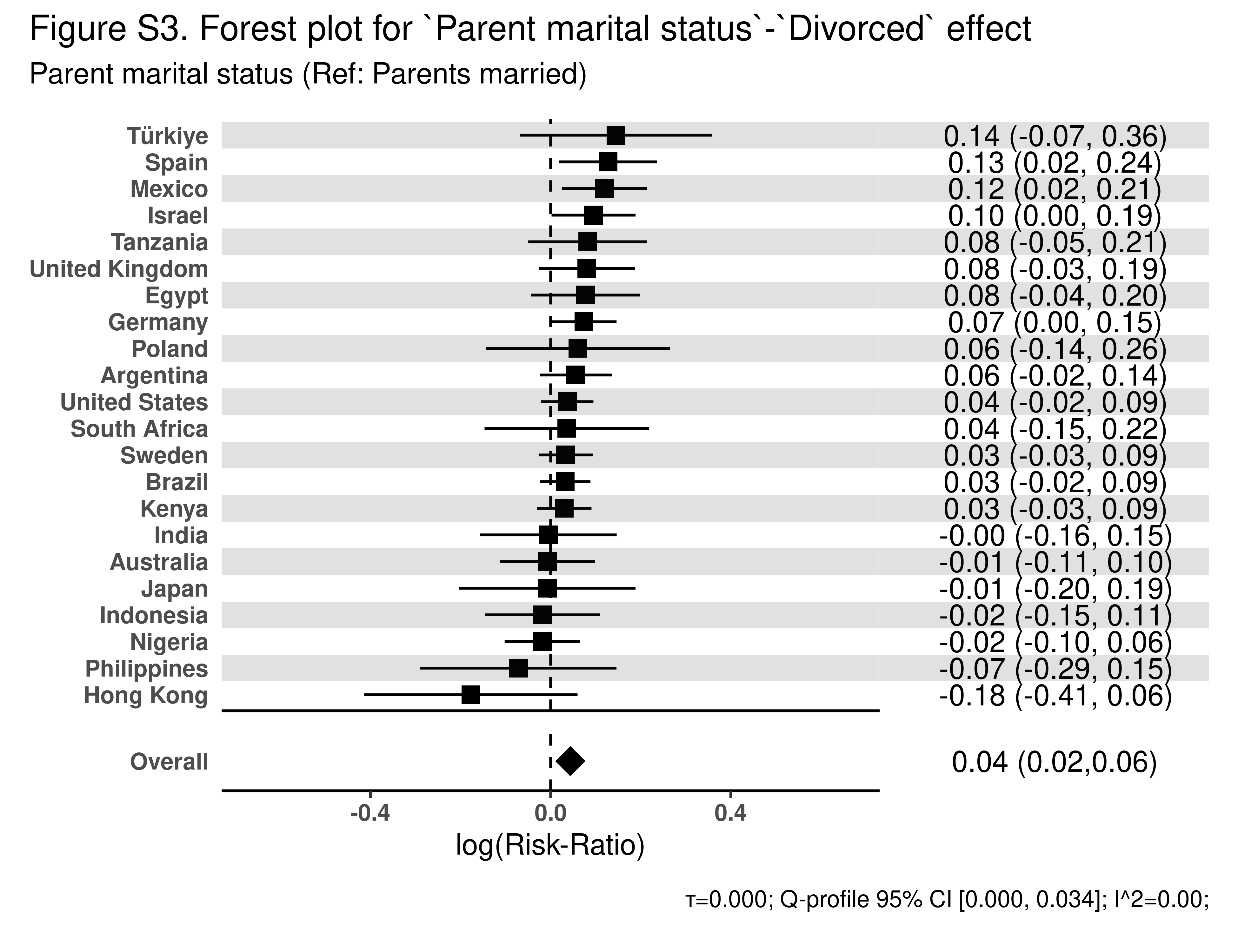 |  |
| ***Figure S4. Forest plot for ‘Parent marital status’ – ‘Single, never married’ effect*** | 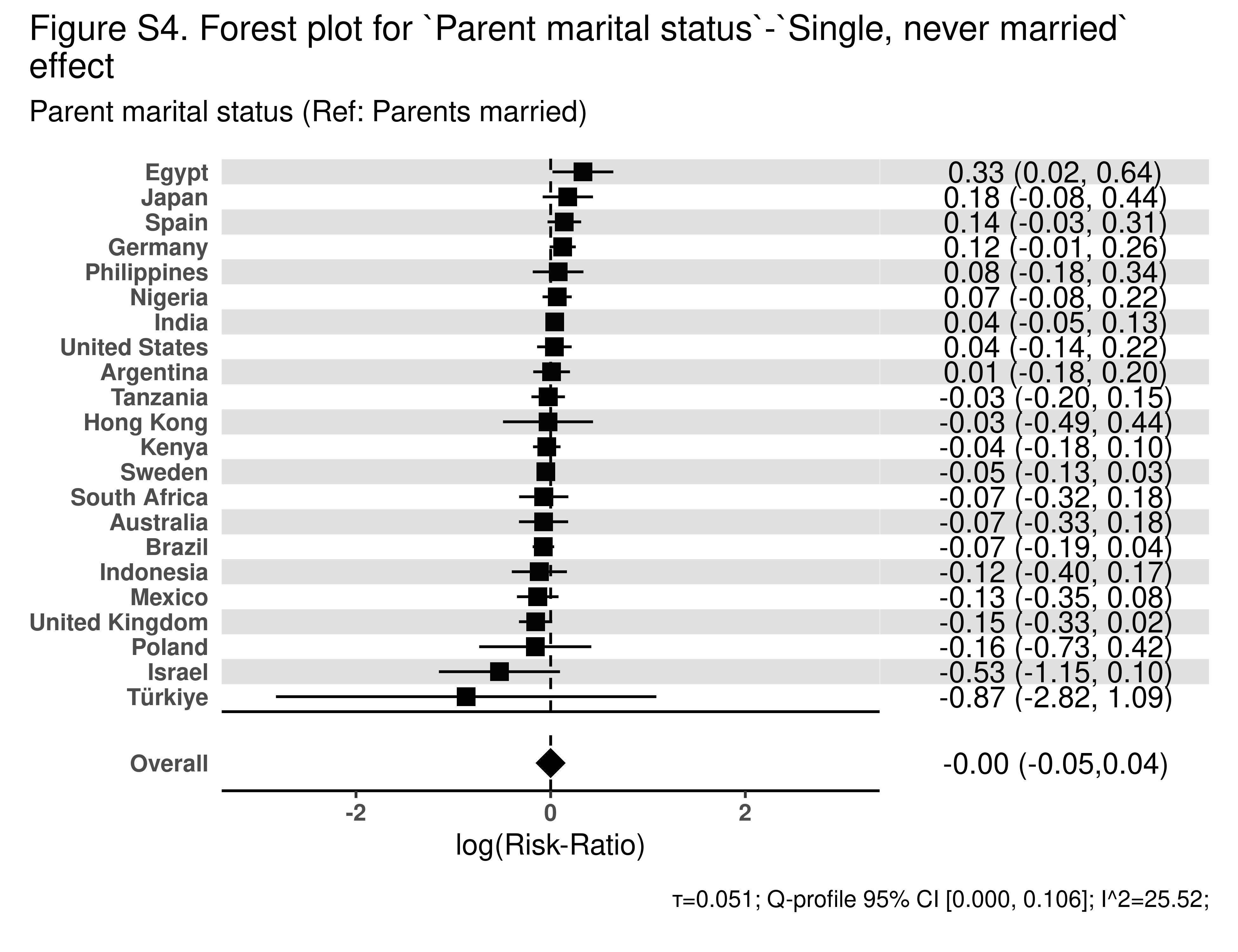 | 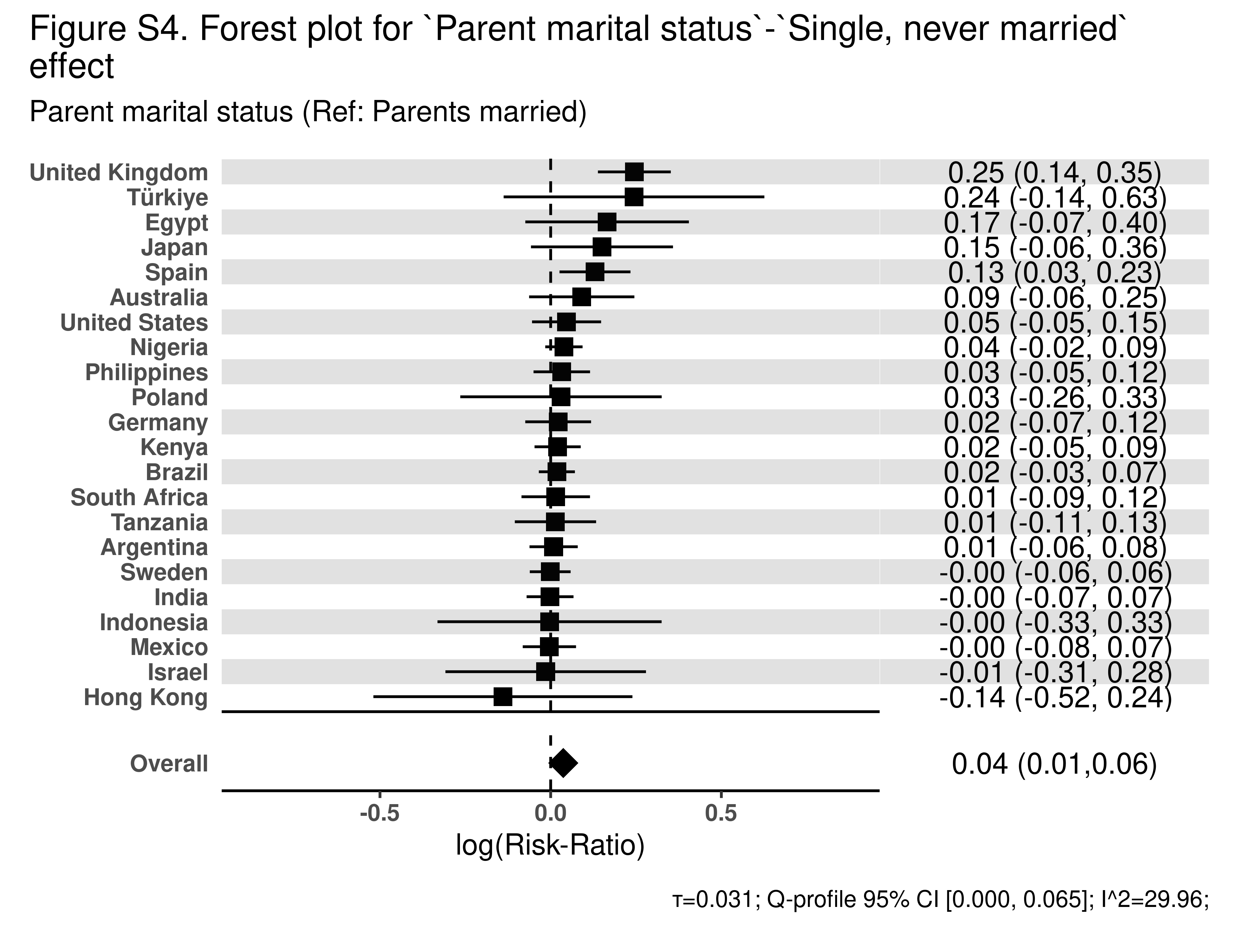 |  |
| ***Figure S5. Forest plot for ‘Parent marital status’ – ‘One or both parents had died’ effect*** | 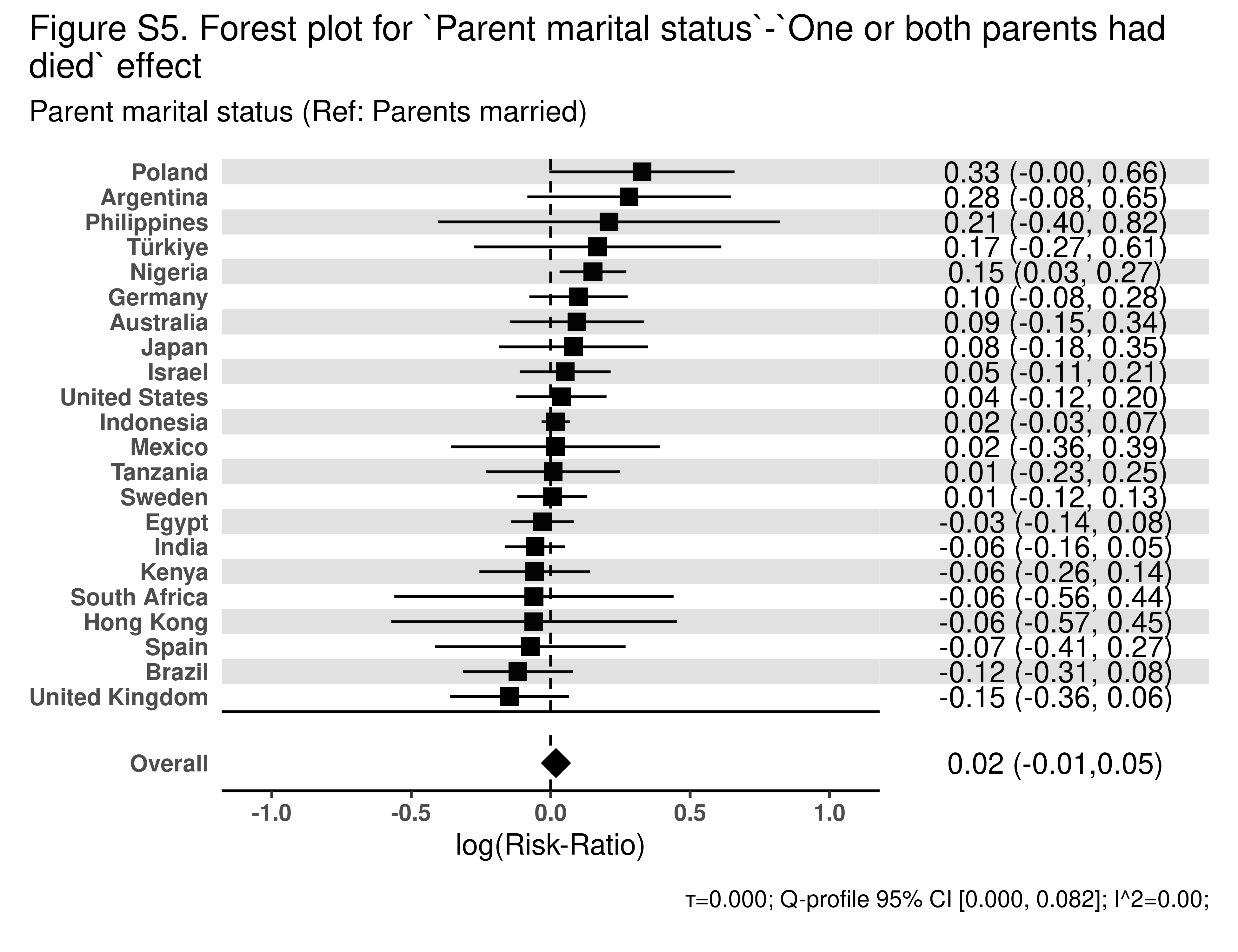 | 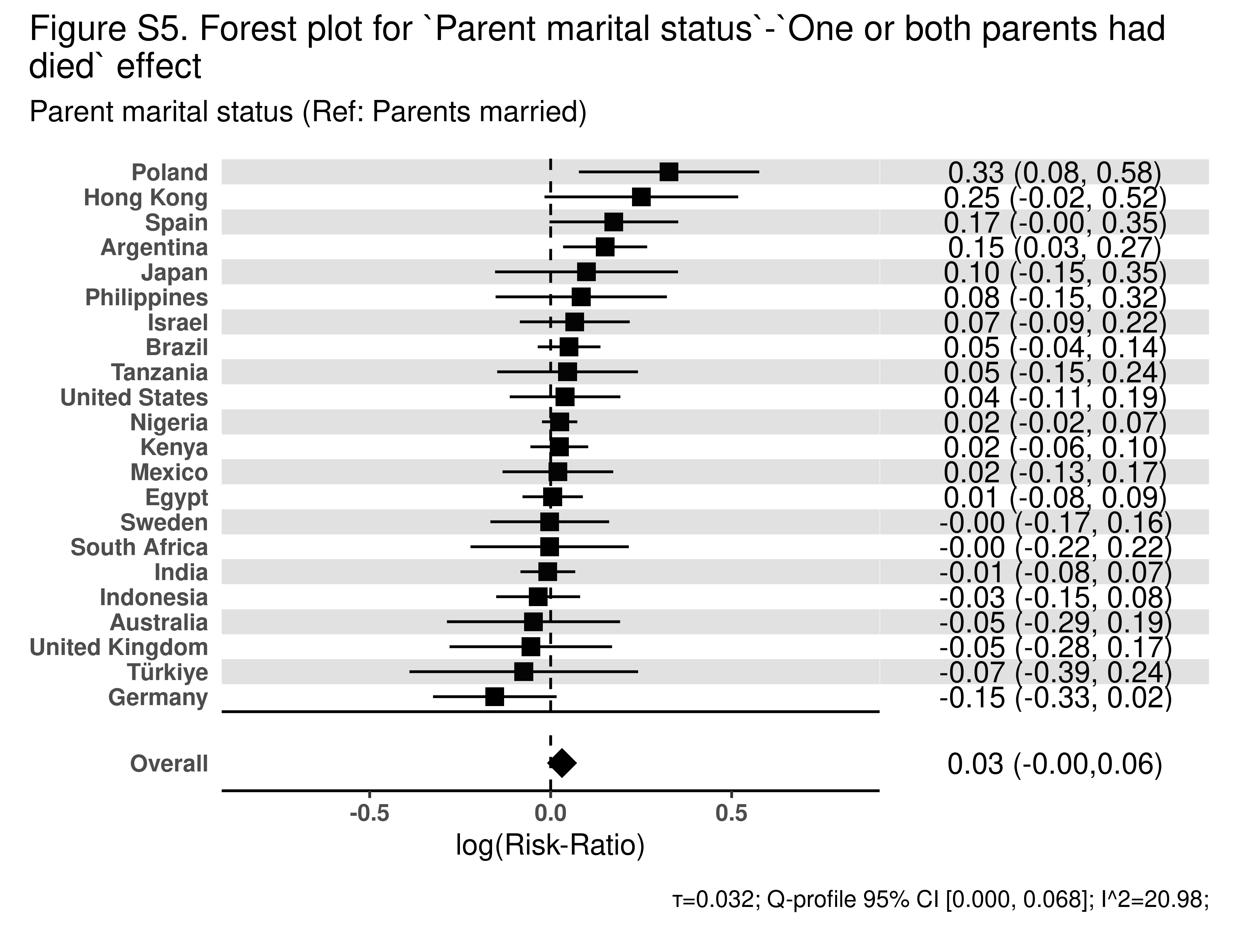 |  |
| ***Figure S6. Forest plot for ‘Subjective financial status of family growing up’ – ‘Lived comfortably’ effect*** | 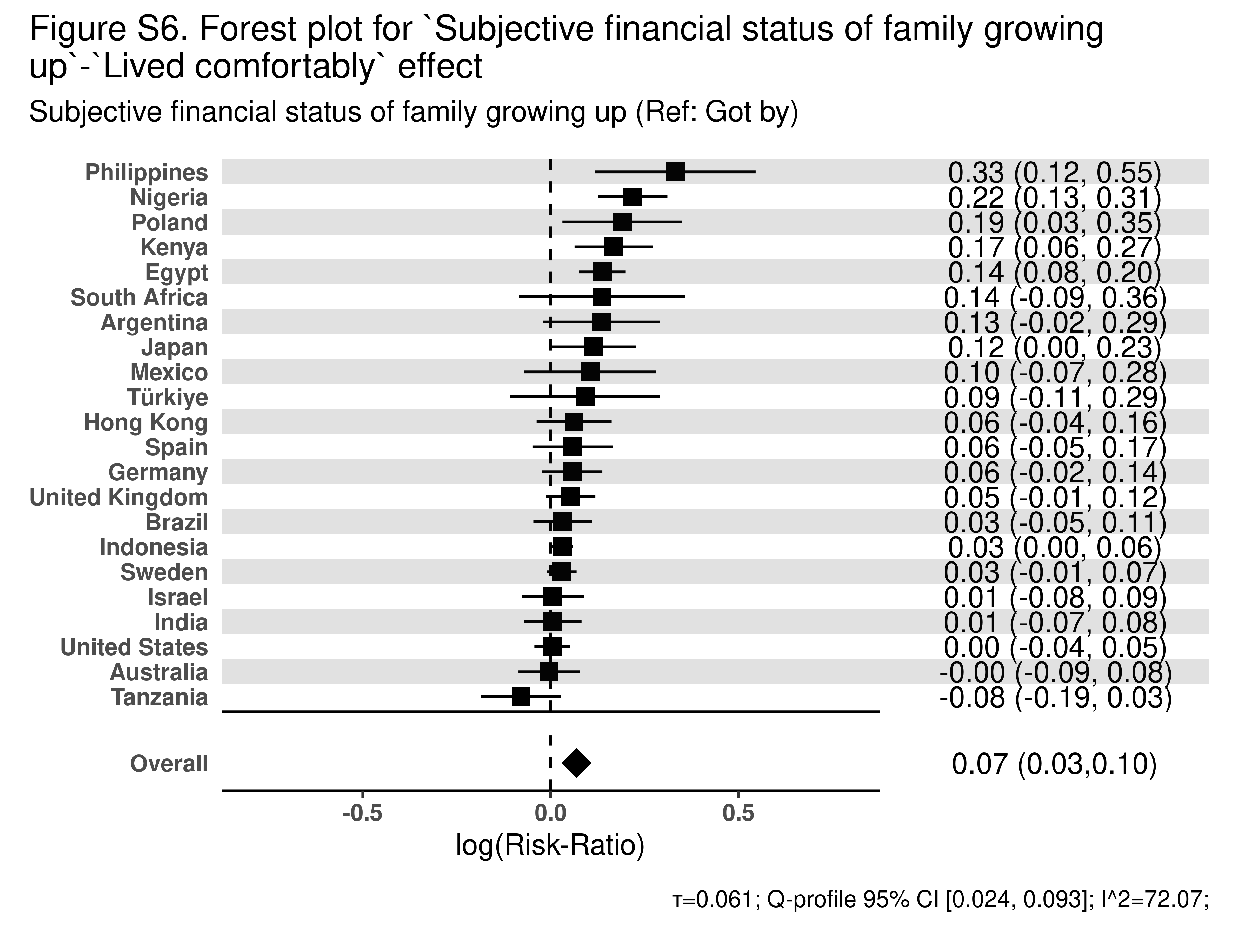 | 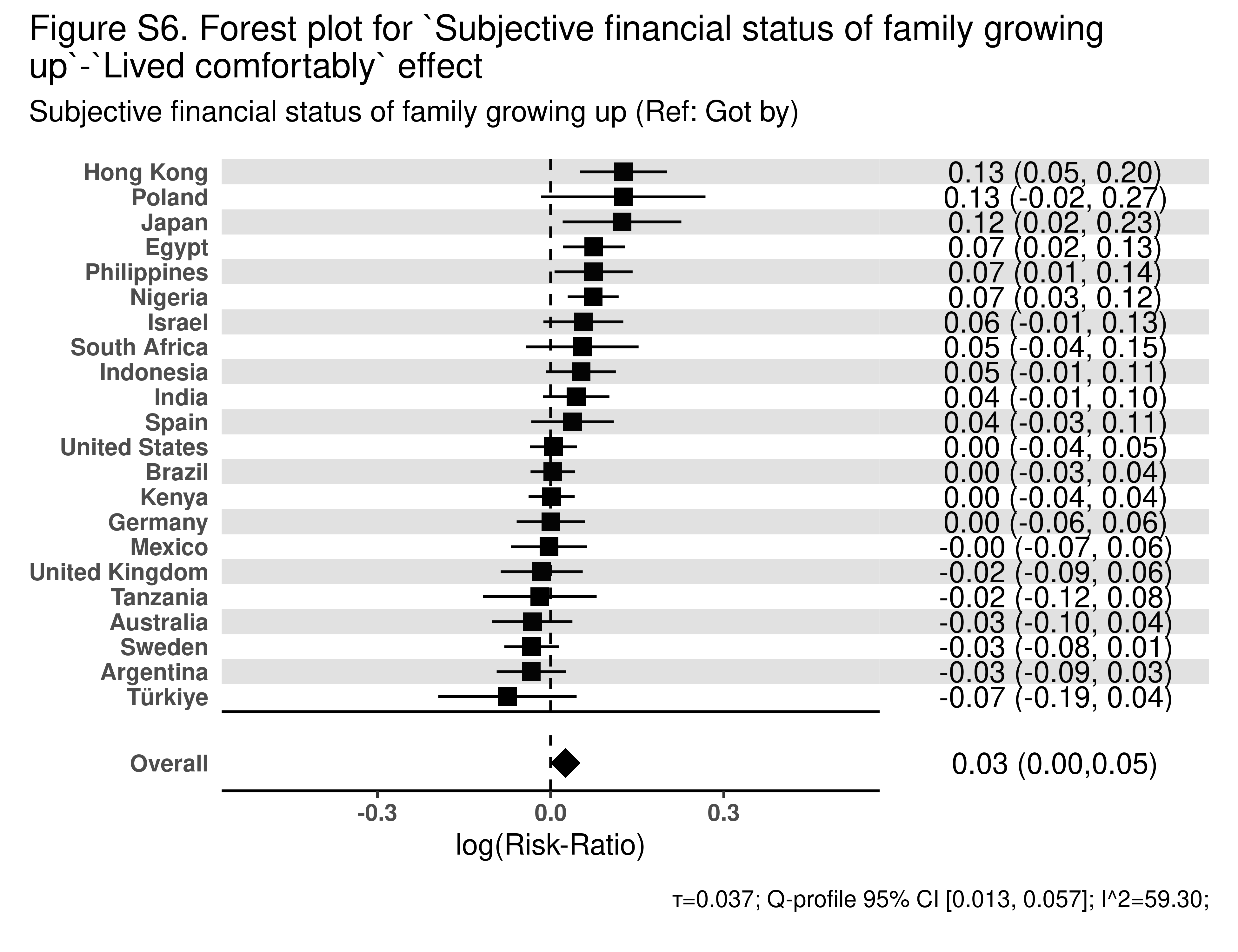 |  |
| ***Figure S7. Forest plot for ‘Subjective financial status of family growing up’ – ‘Found it difficult’ effect*** | 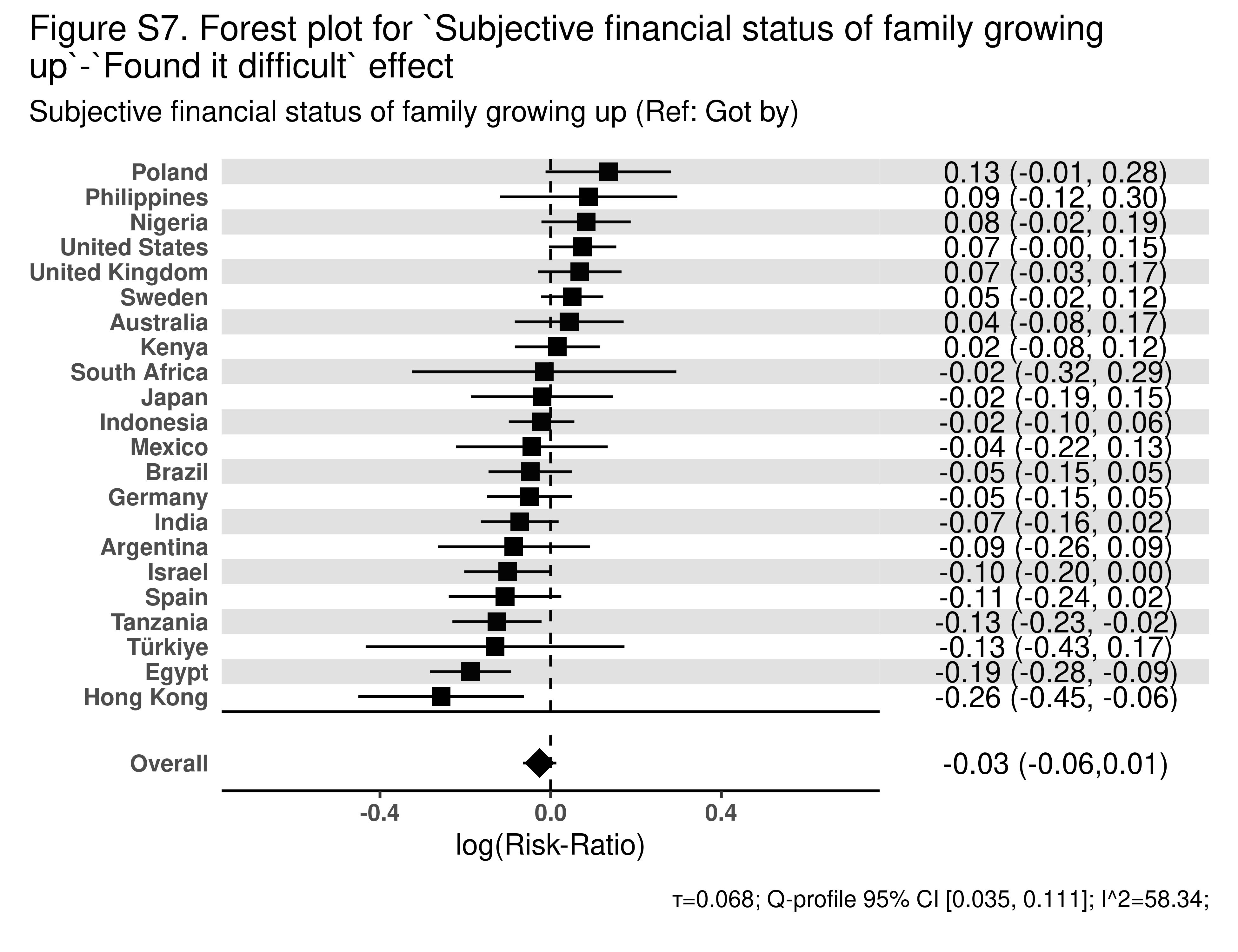 | 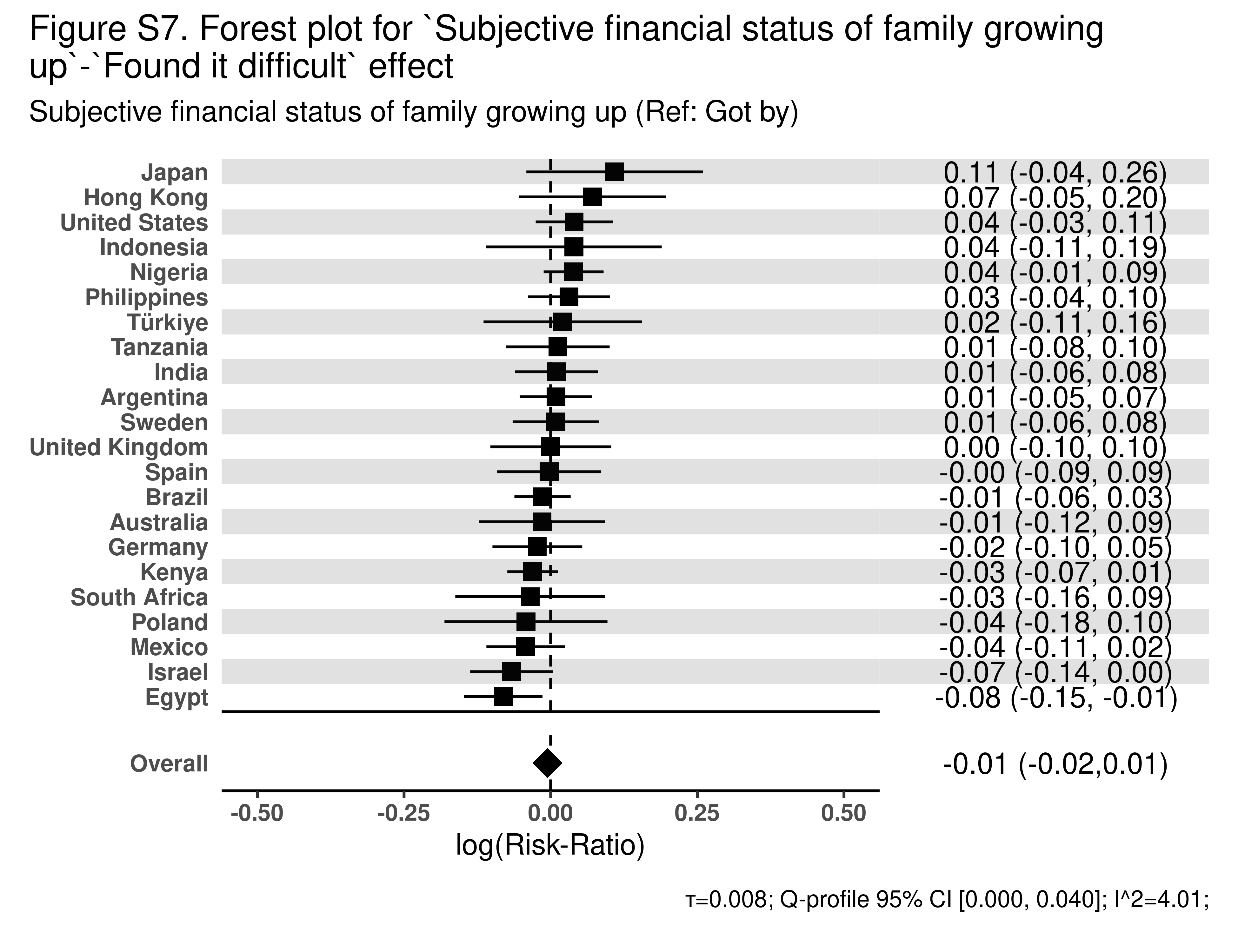 |  |
| ***Figure S8. Forest plot for ‘Subjective financial status of family growing up’ – ‘Found it very difficult’ effect*** | 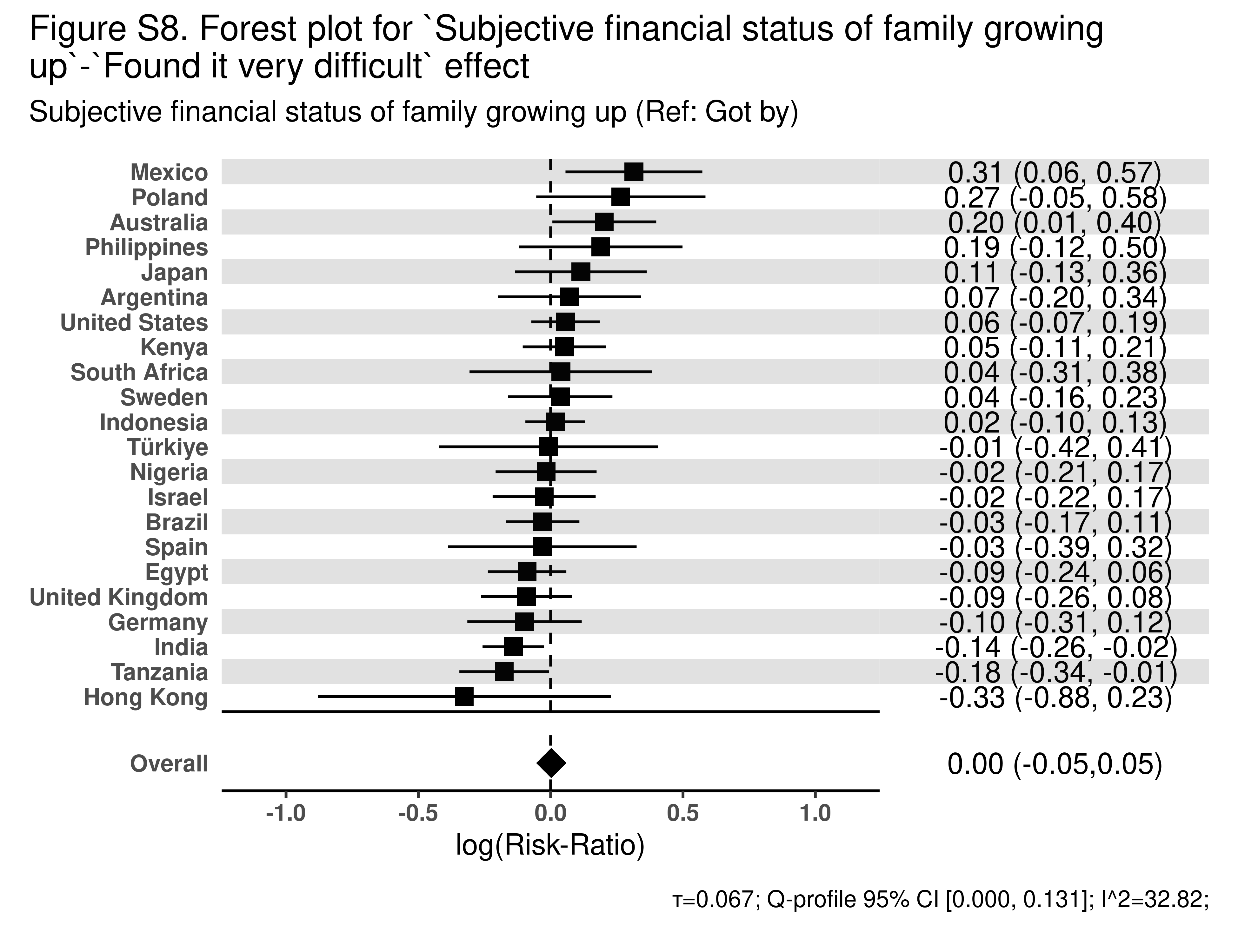 | 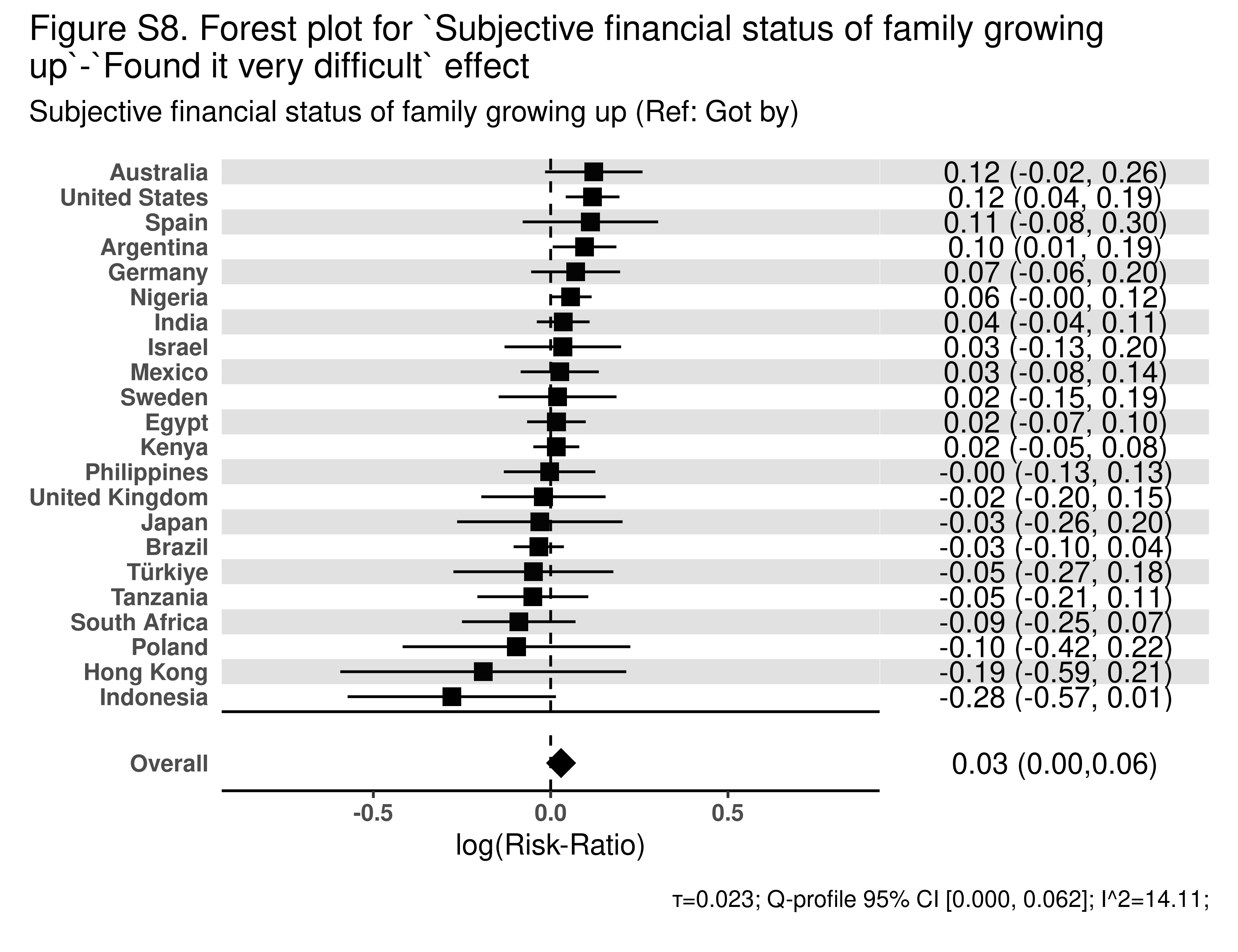 |  |
| ***Figure S9. Forest plot for ‘Abuse’ – ‘Yes’ effect*** | 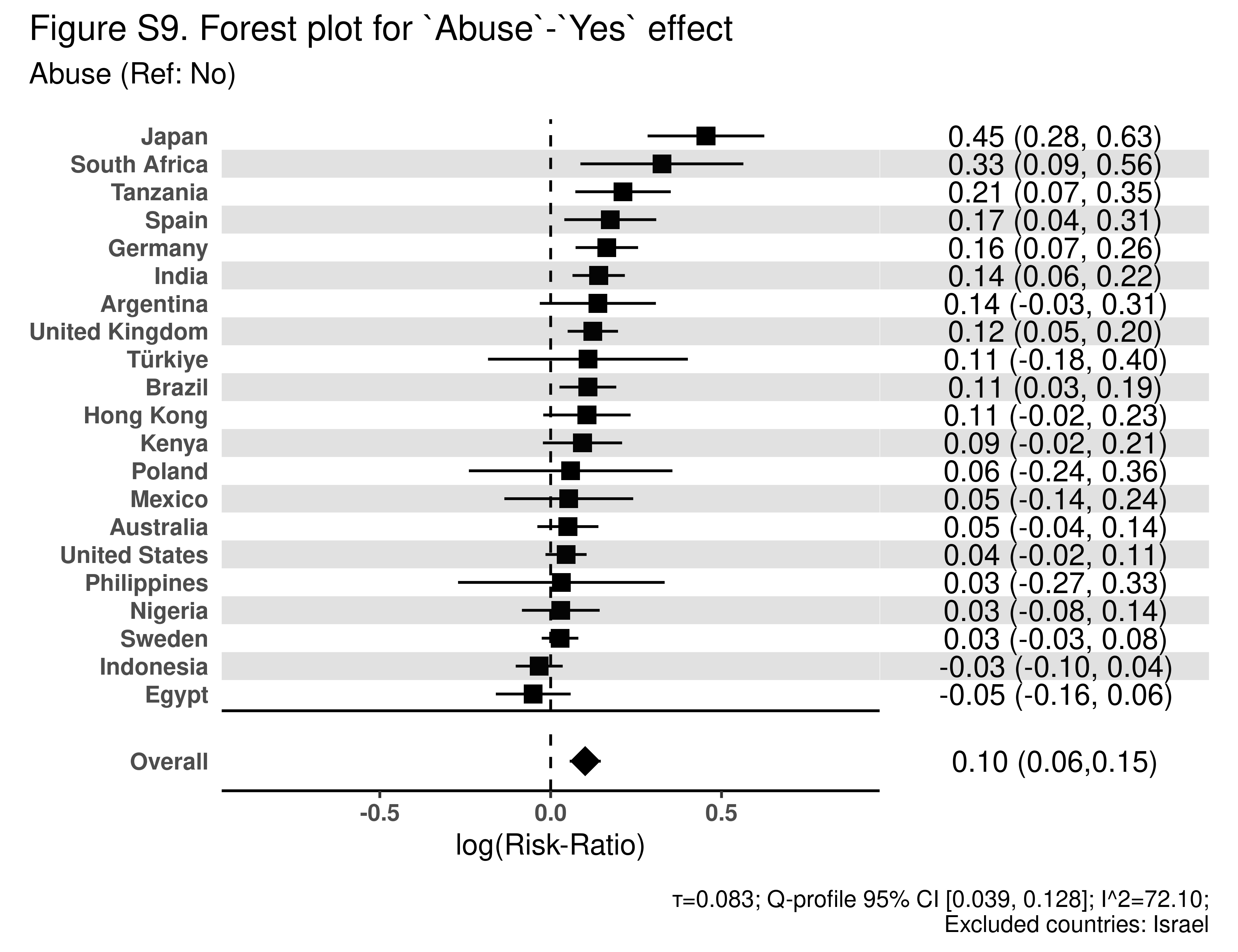 | 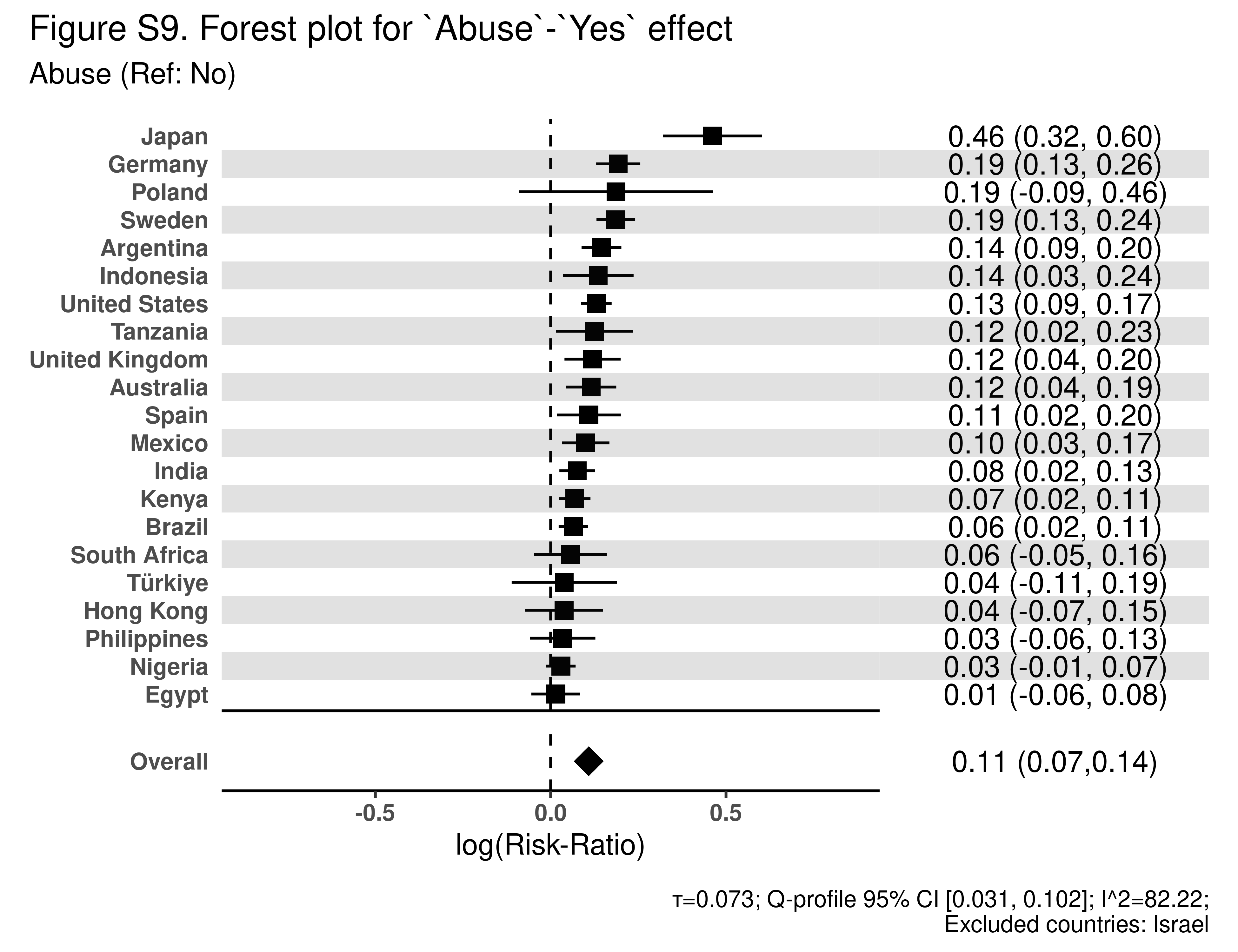 |  |
| ***Figure S10. Forest plot for ‘Outsider growing up’ – ‘Yes’ effect*** | 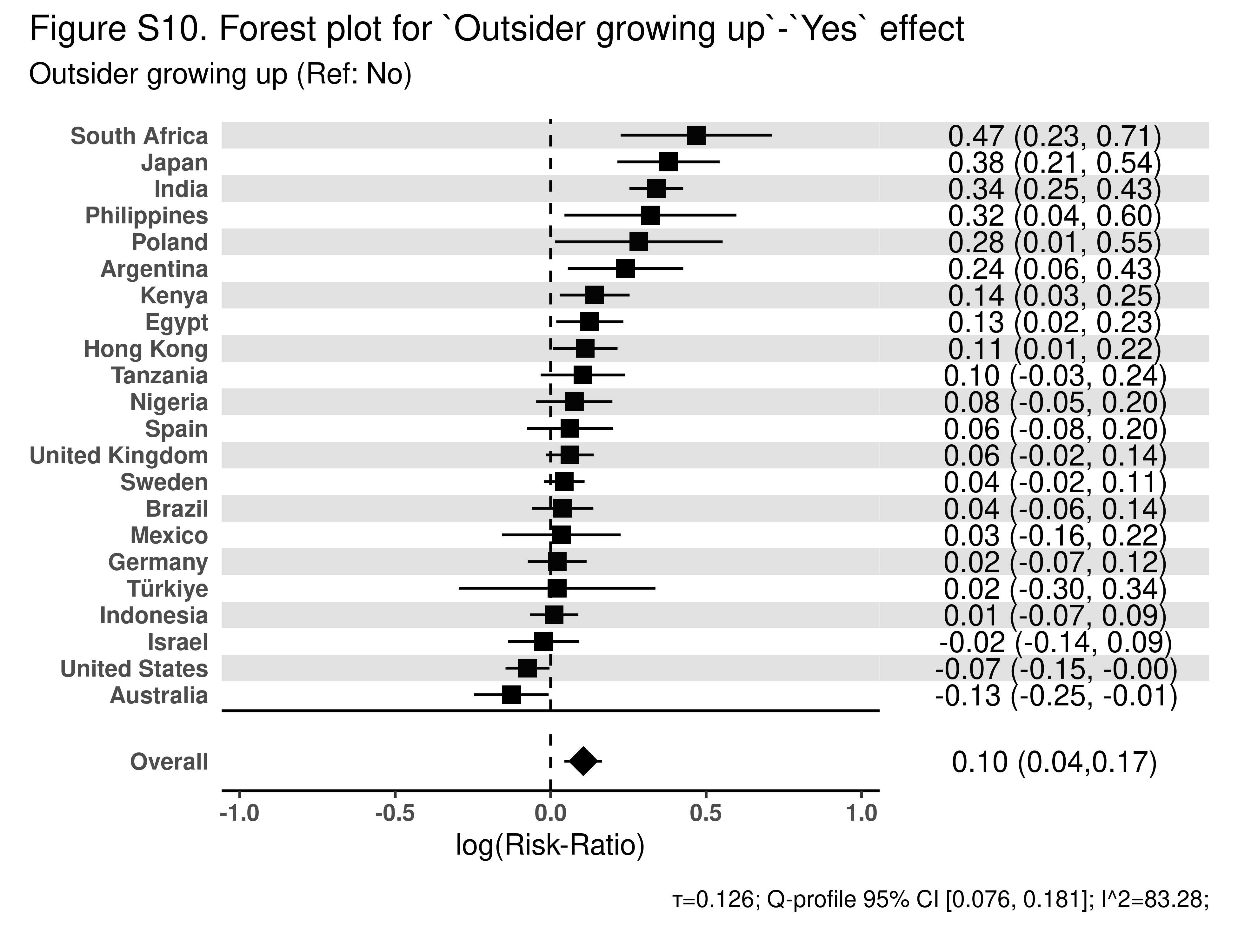 | 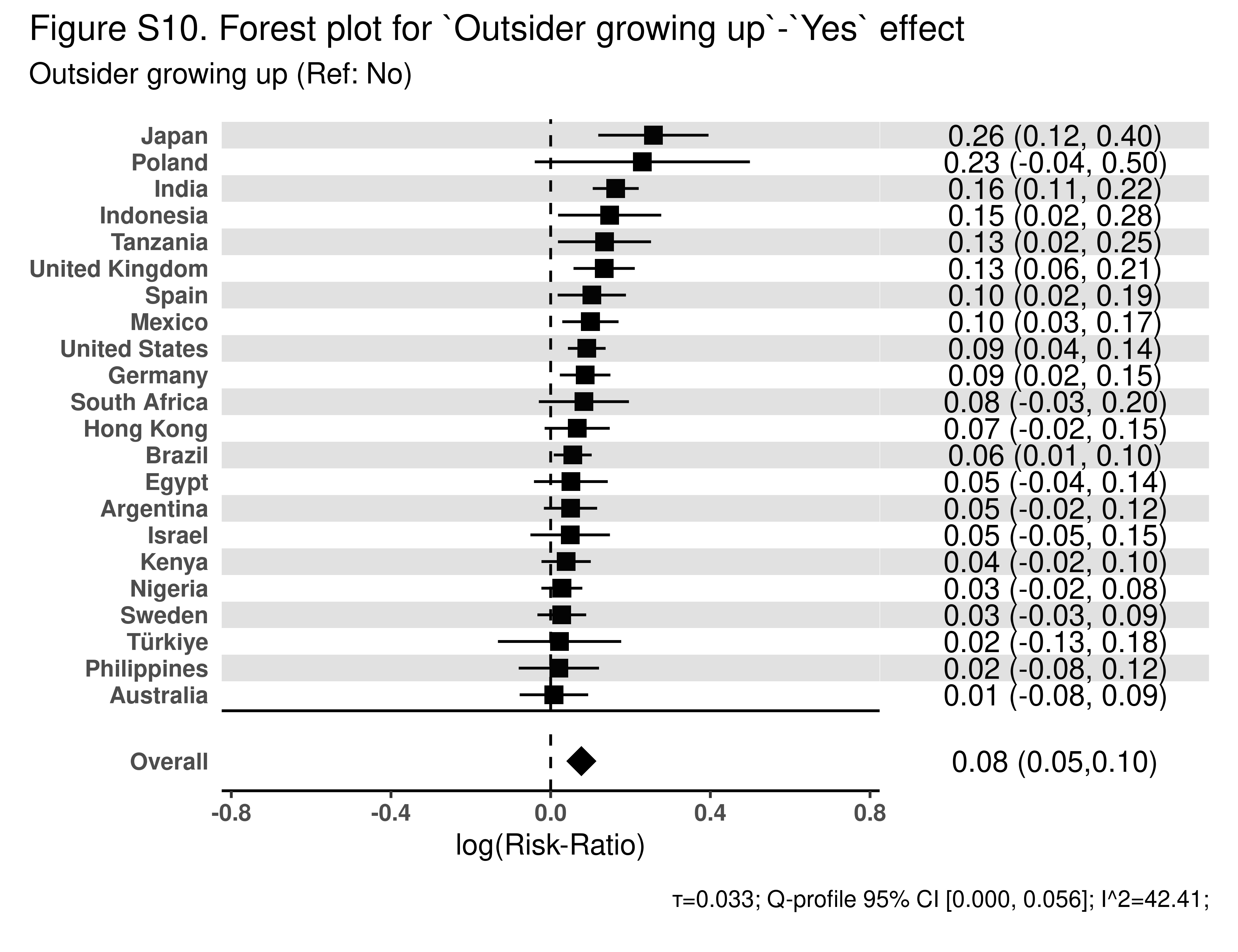 |  |
| ***Figure S11. Forest plot for ‘Self-rated health growing up’ – ‘Excellent’ effect*** | 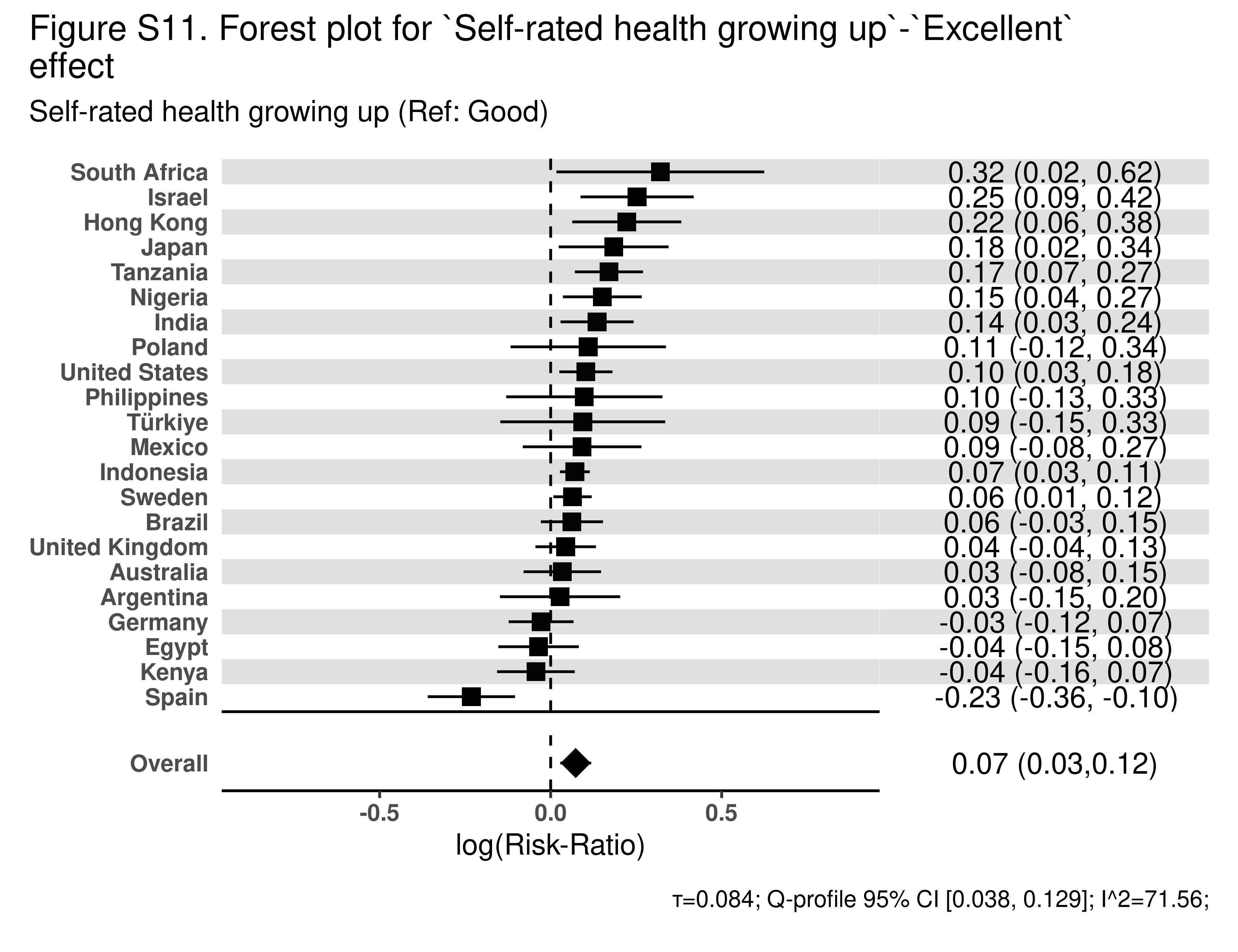 | 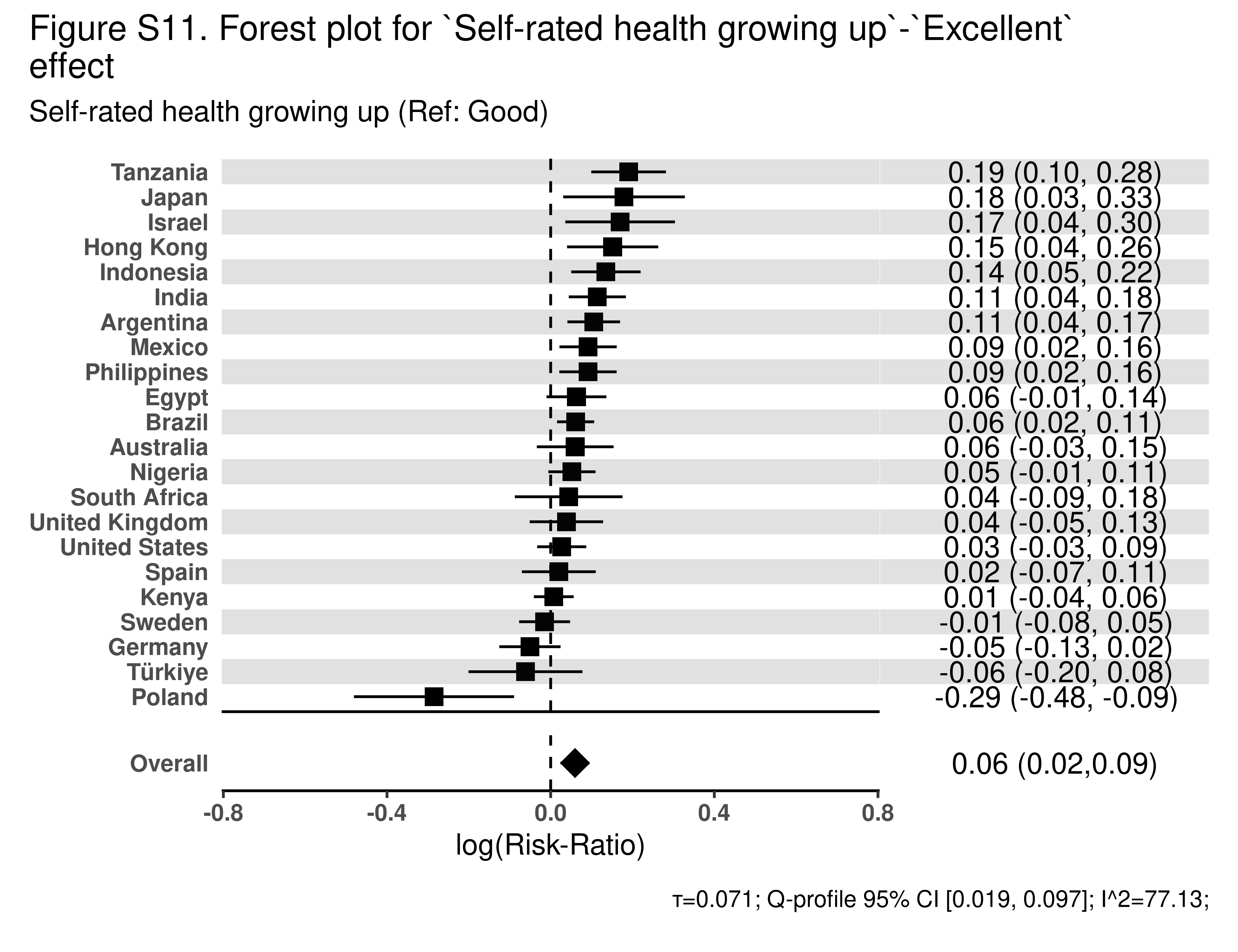 |  |
| ***Figure S12. Forest plot for ‘Self-rated health growing up’ – ‘Very good’ effect*** | 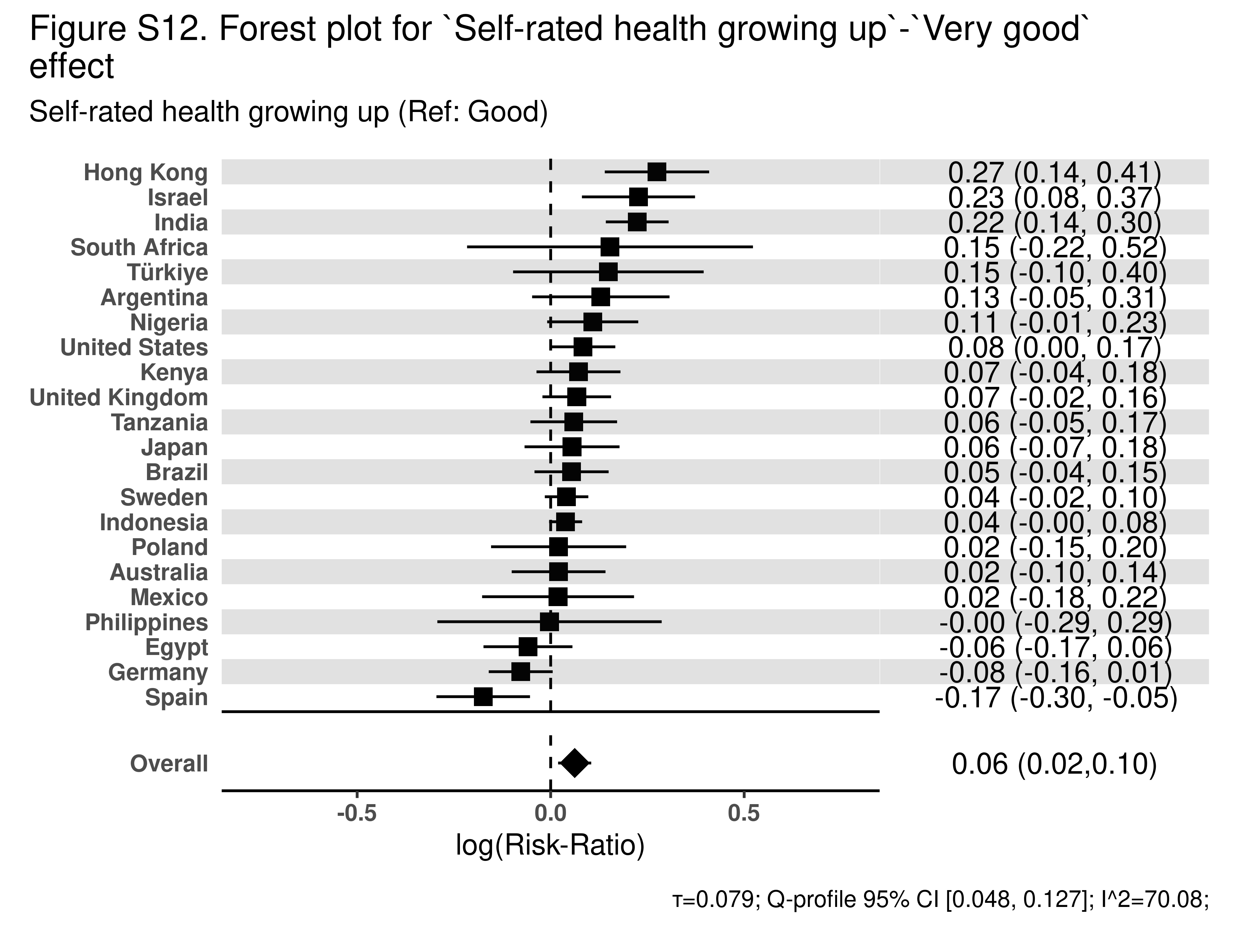 | 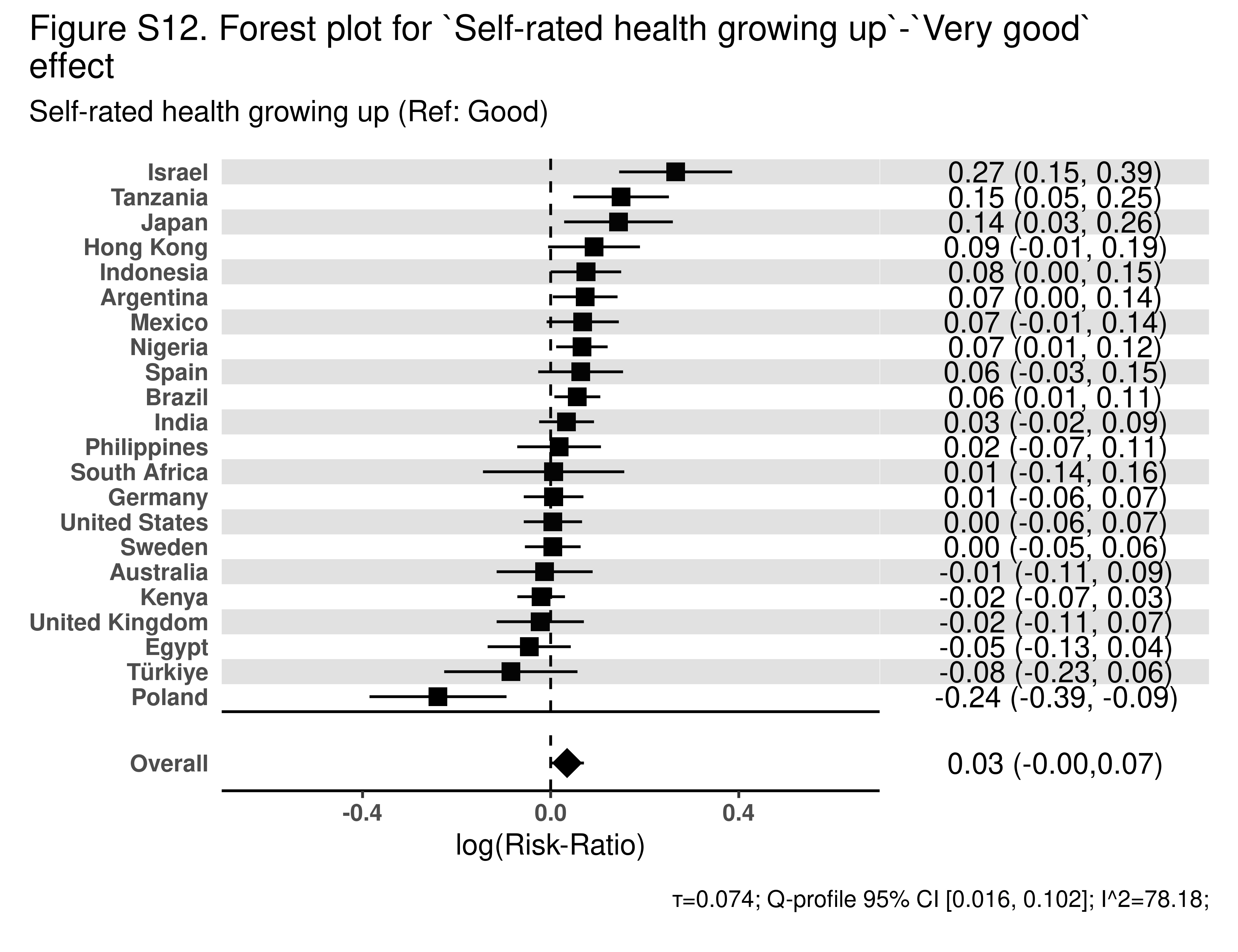 |  |
| ***Figure S13. Forest plot for ‘Self-rated health growing up’ – ‘Fair’ effect*** | 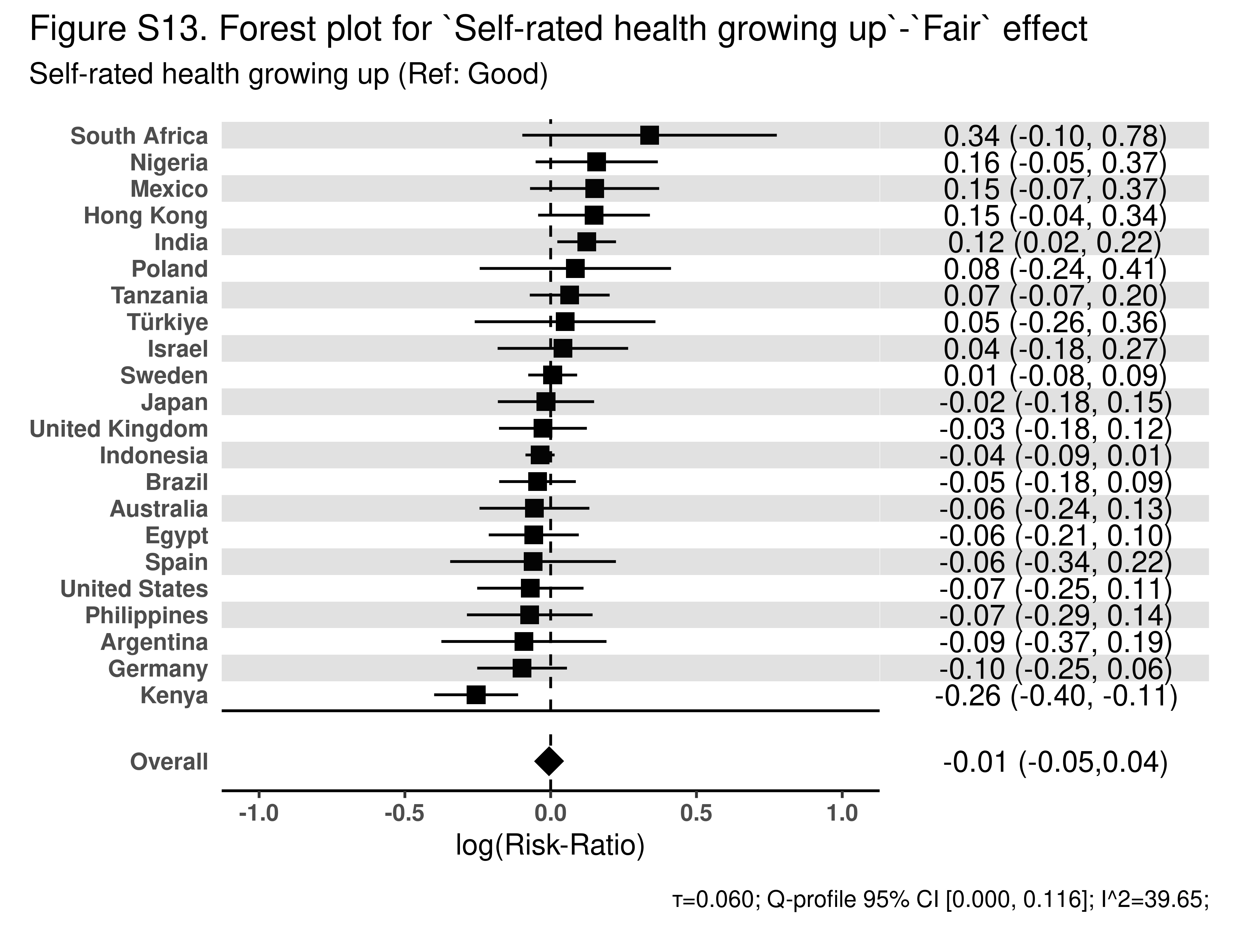 | 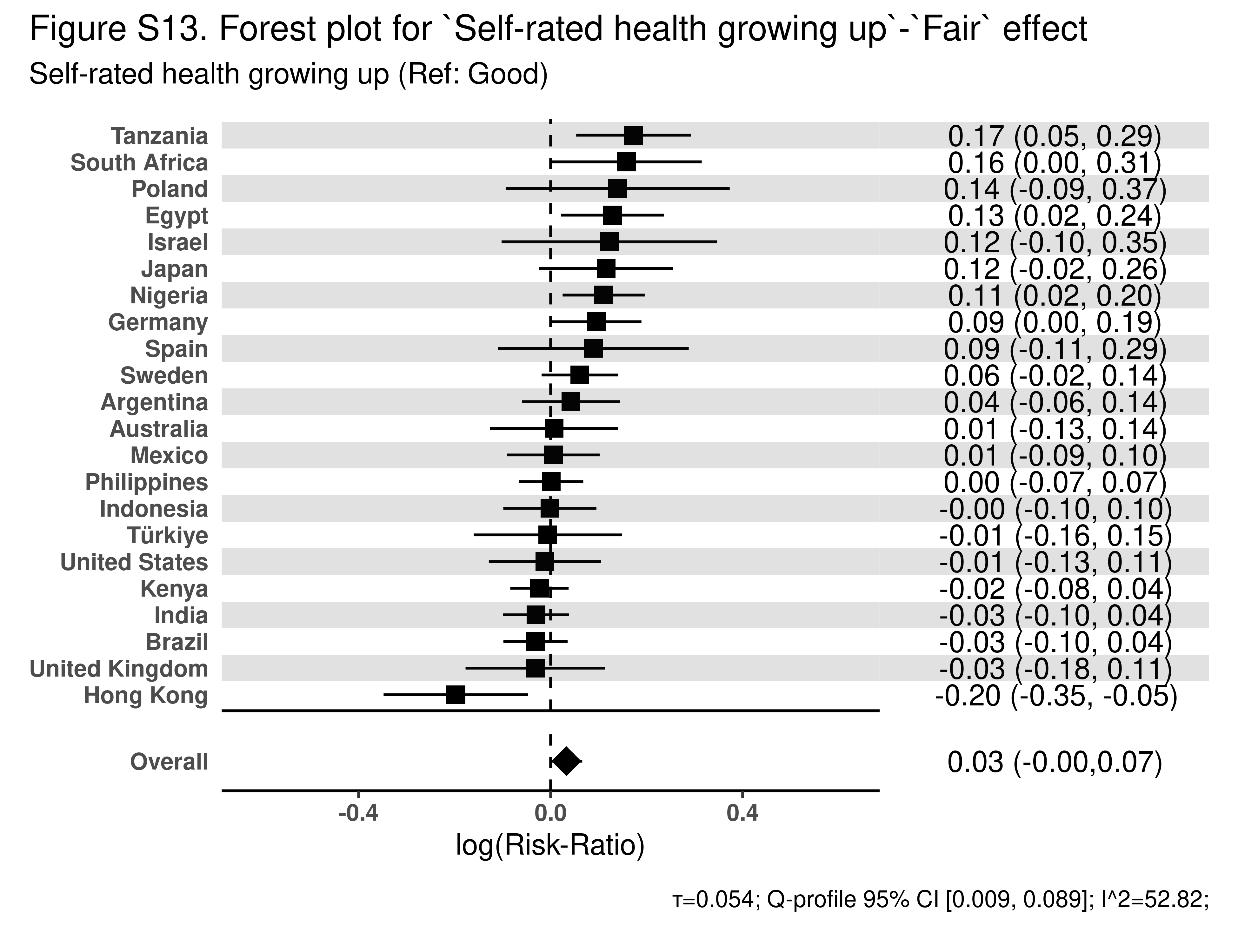 |  |
| ***Figure S14. Forest plot for ‘Self-rated health growing up’ – ‘Poor’ effect*** | 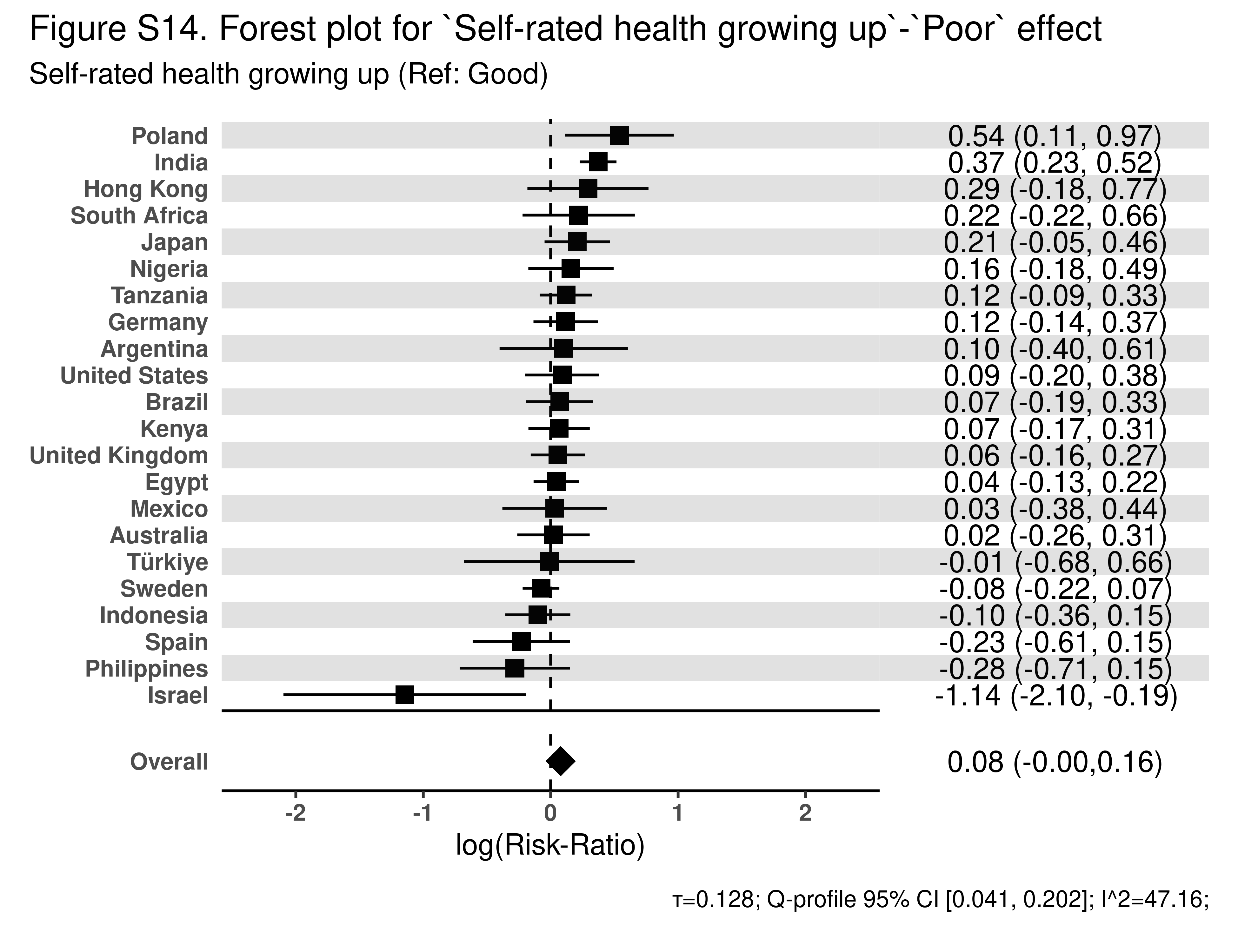 | 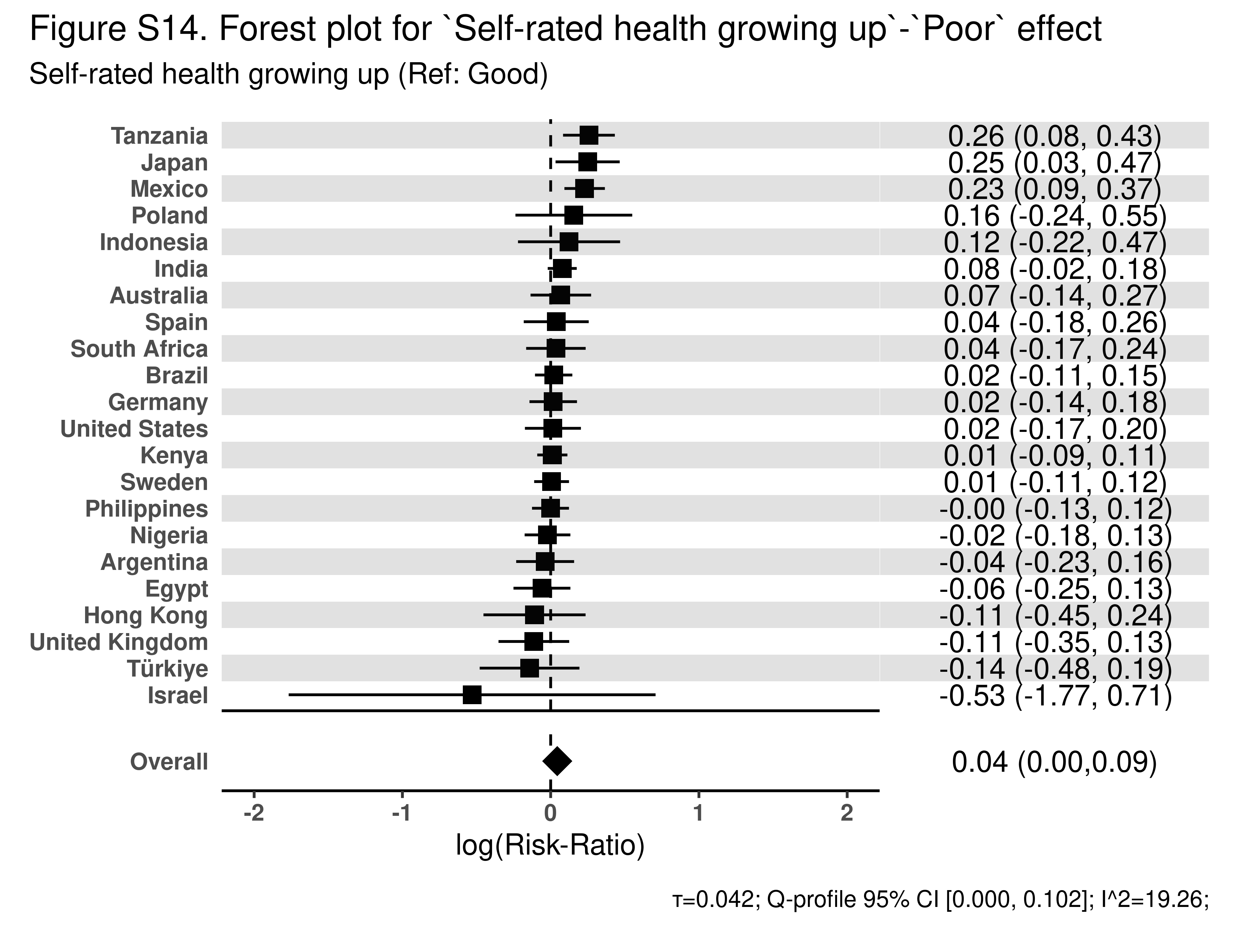 |  |
| ***Figure S15. Forest plot for ‘Immigration status’ – ‘Born in another country’ effect*** | 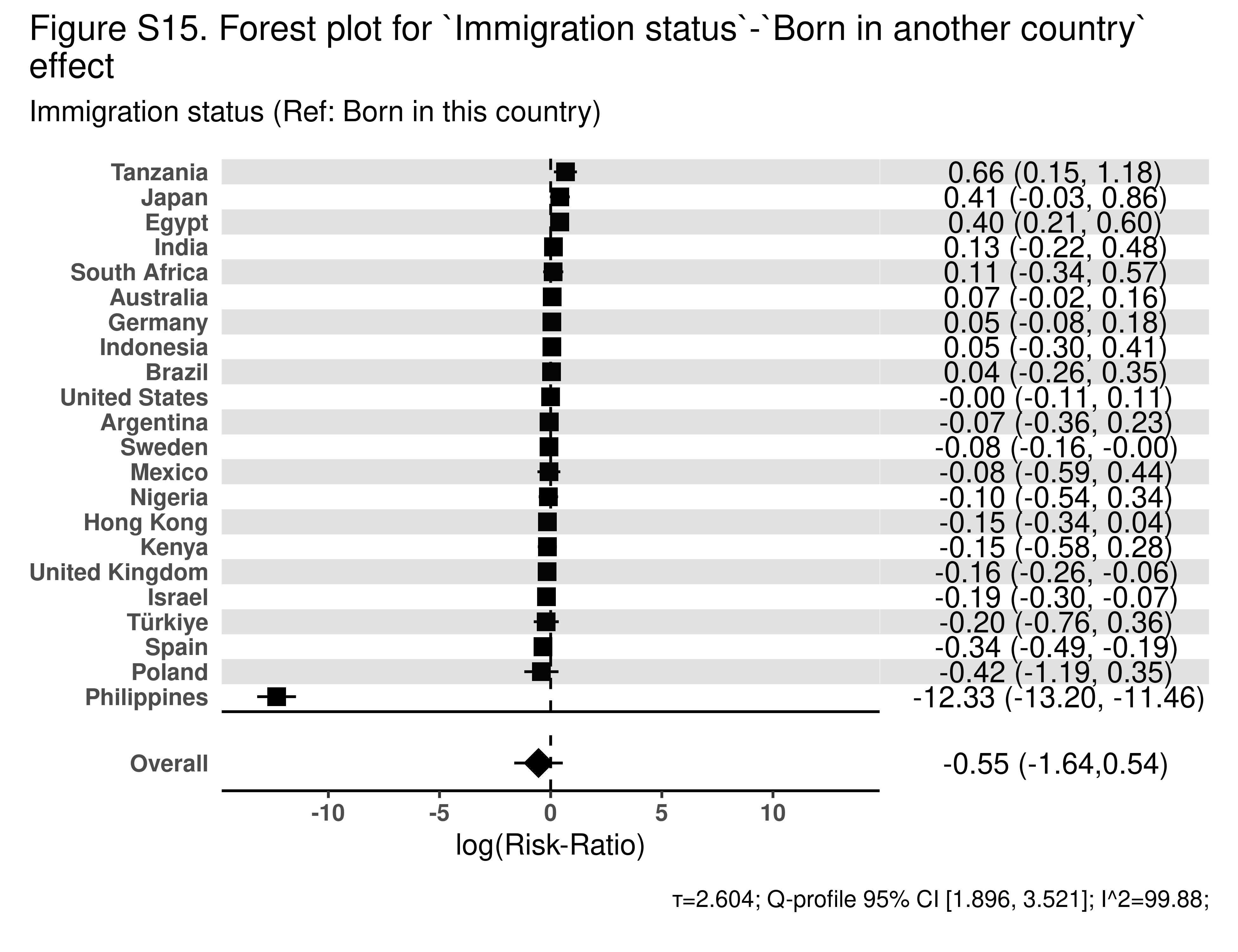 | 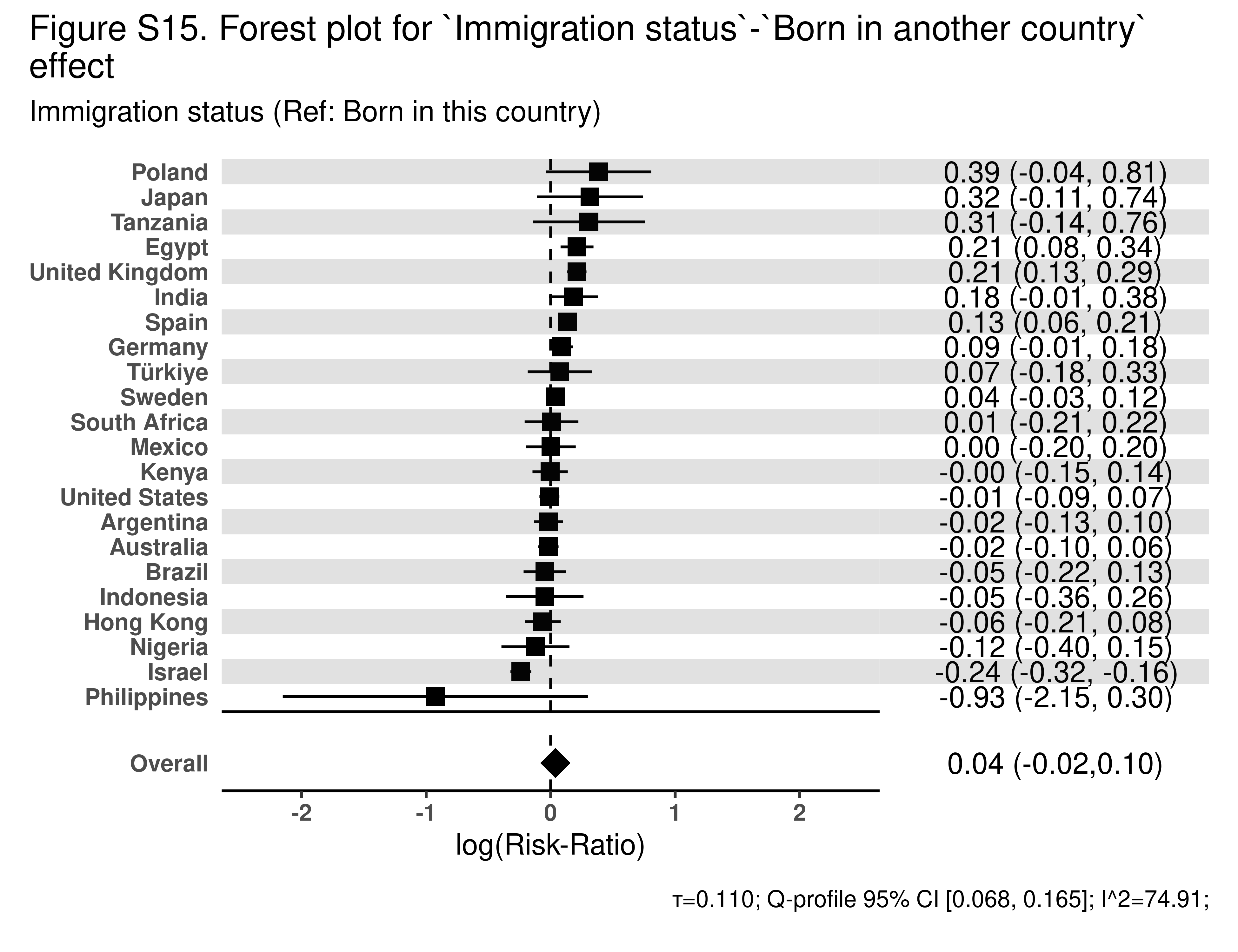 |  |
| ***Figure S16. Forest plot for ‘Age 12 religious service attendance’ – ‘At least 1/week’ effect*** | 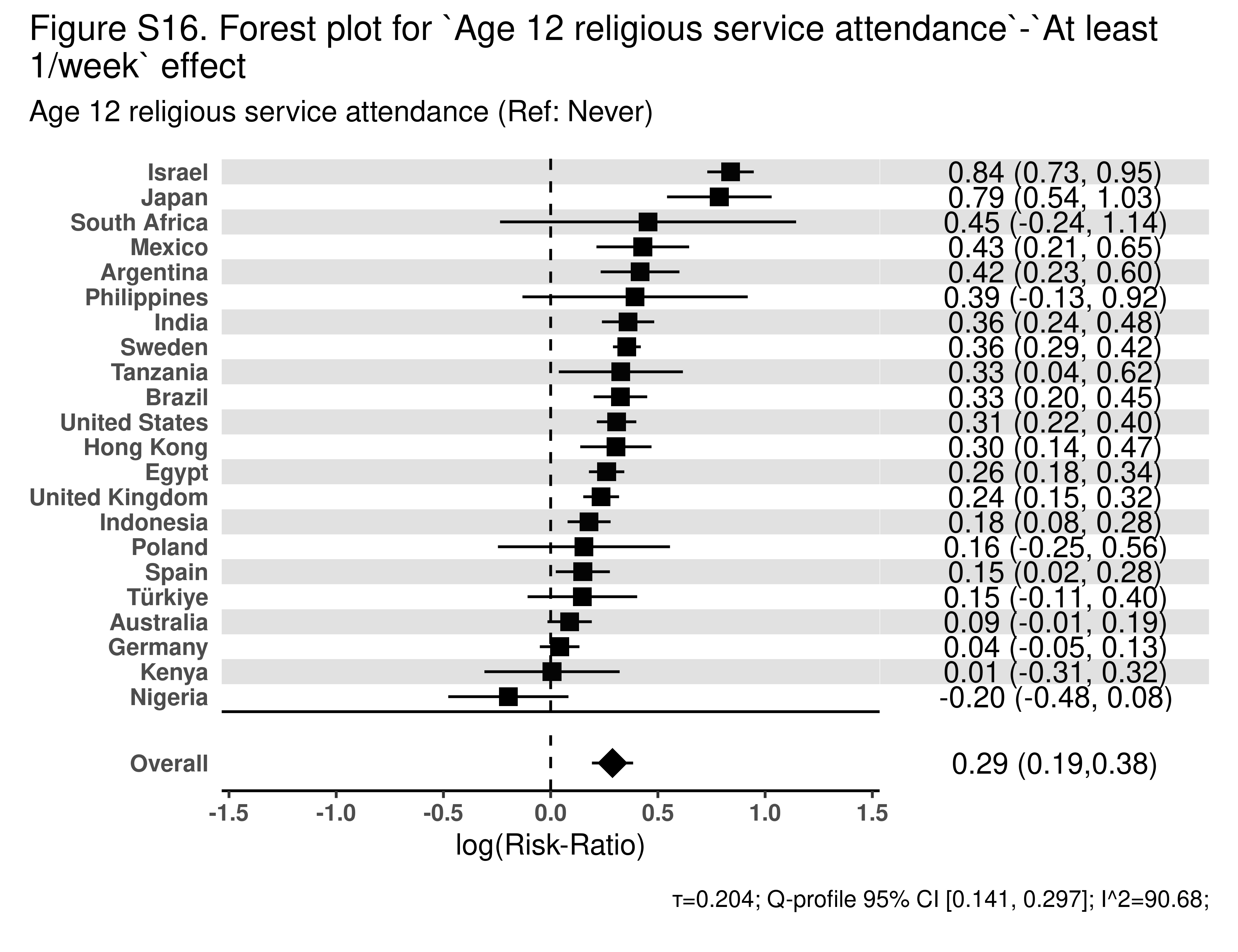 | 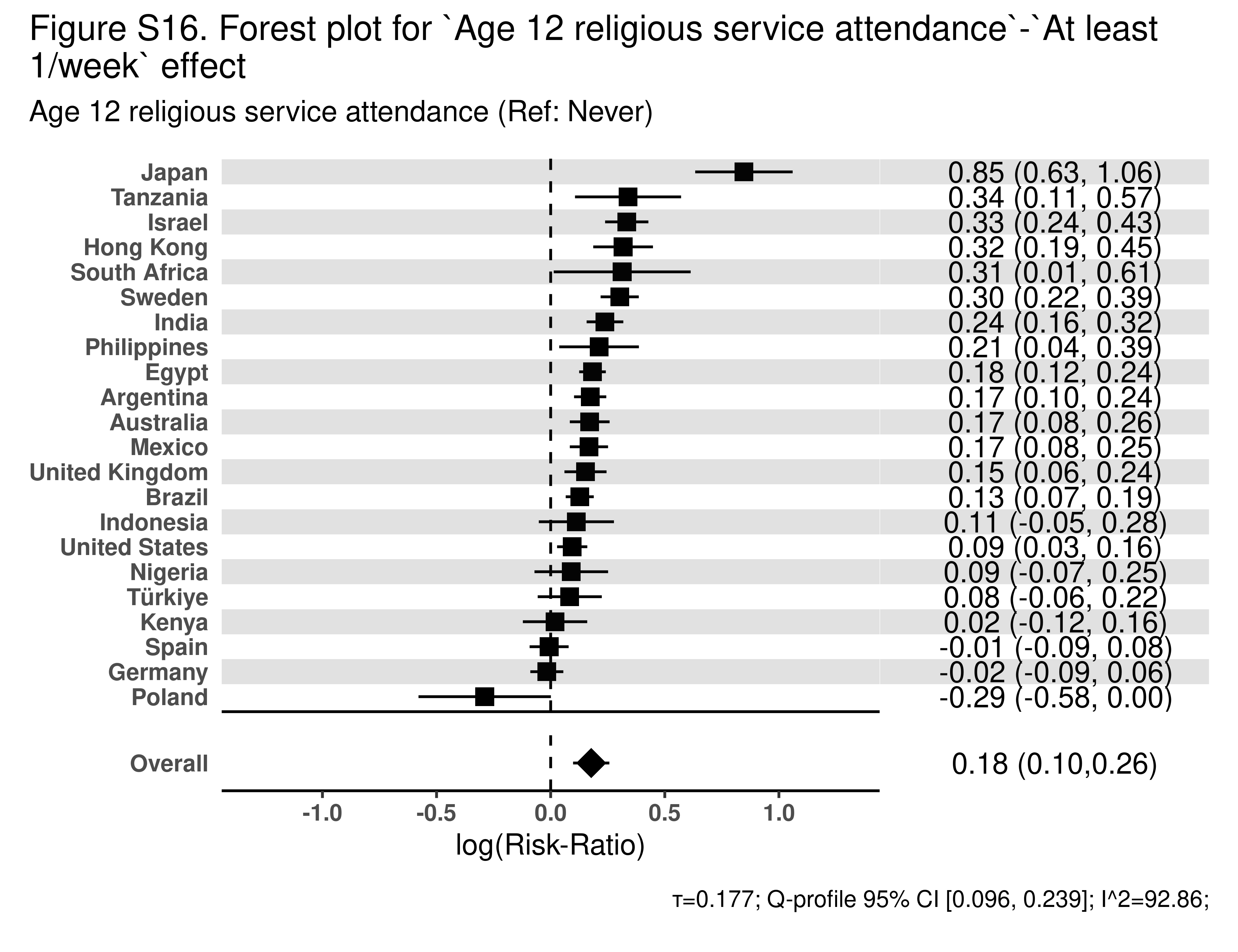 |  |
| ***Figure S17. Forest plot for ‘Age 12 religious service attendance’ – ‘1-3/month’ effect*** | 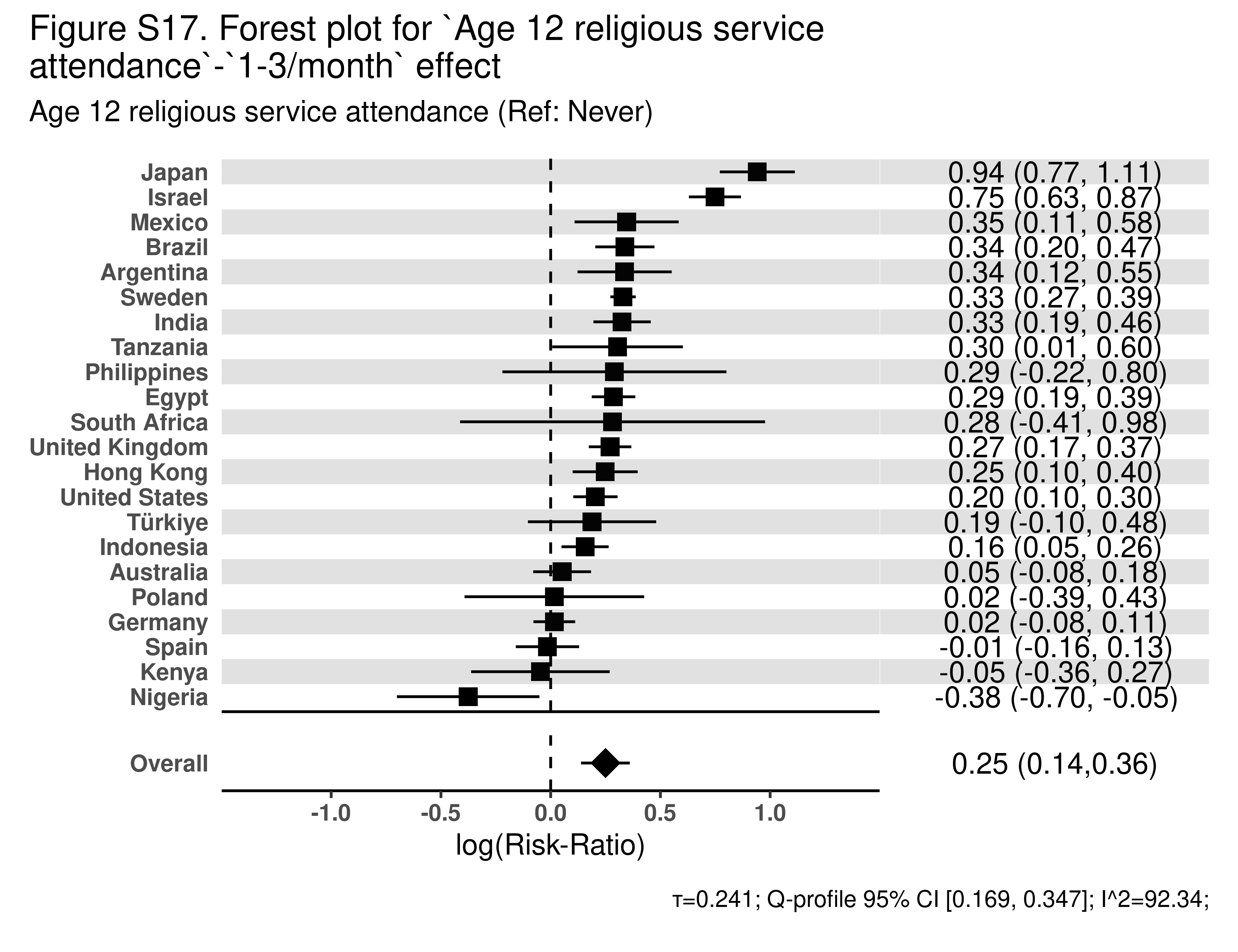 | 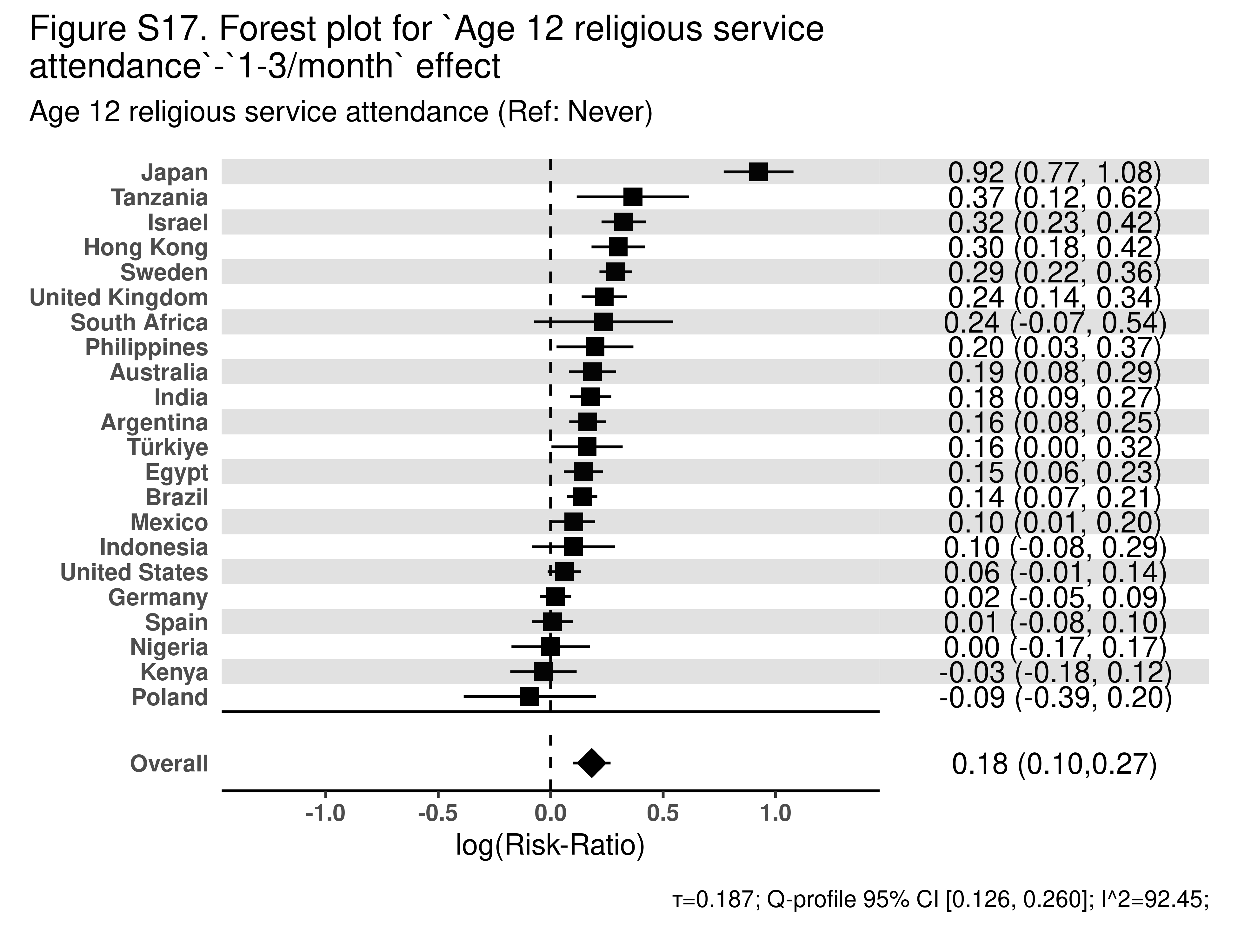 |  |
| ***Figure S18. Forest plot for ‘Age 12 religious service attendance’ – ‘<1/month’ effect*** | 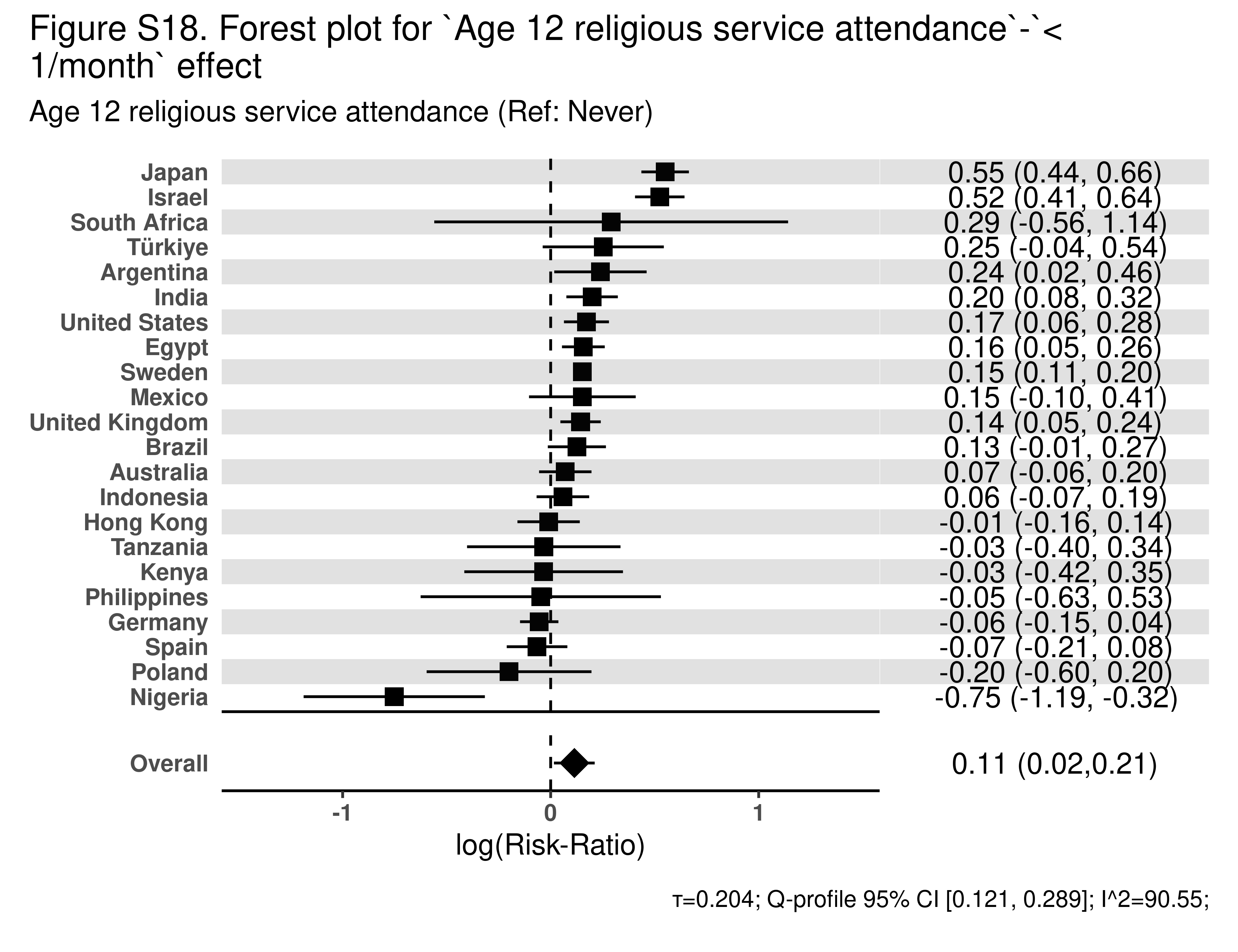 | 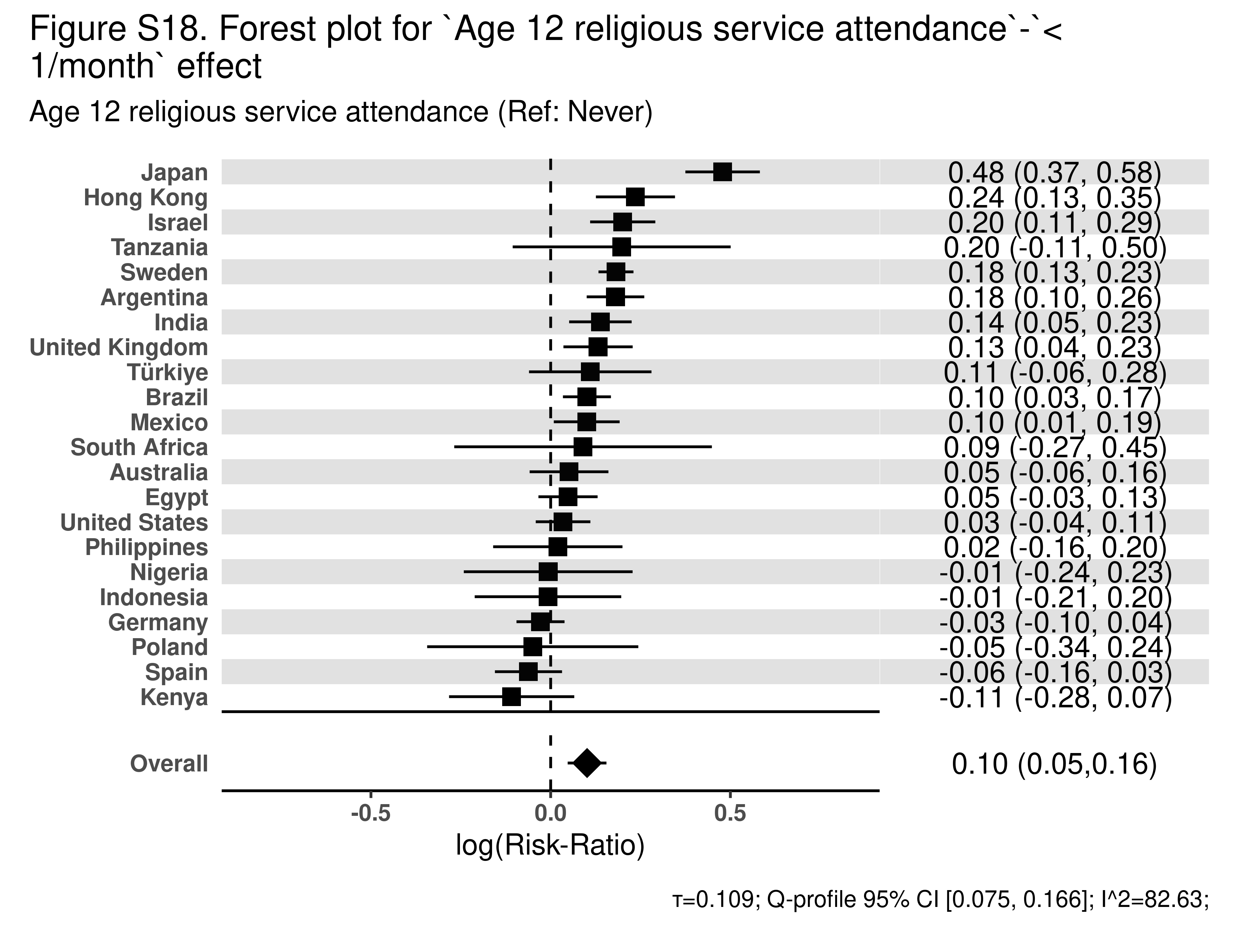 | |
| ***Figure S19. Forest plot for ‘Year of birth’ – ‘1993-1998; age 25-29’ effect*** | 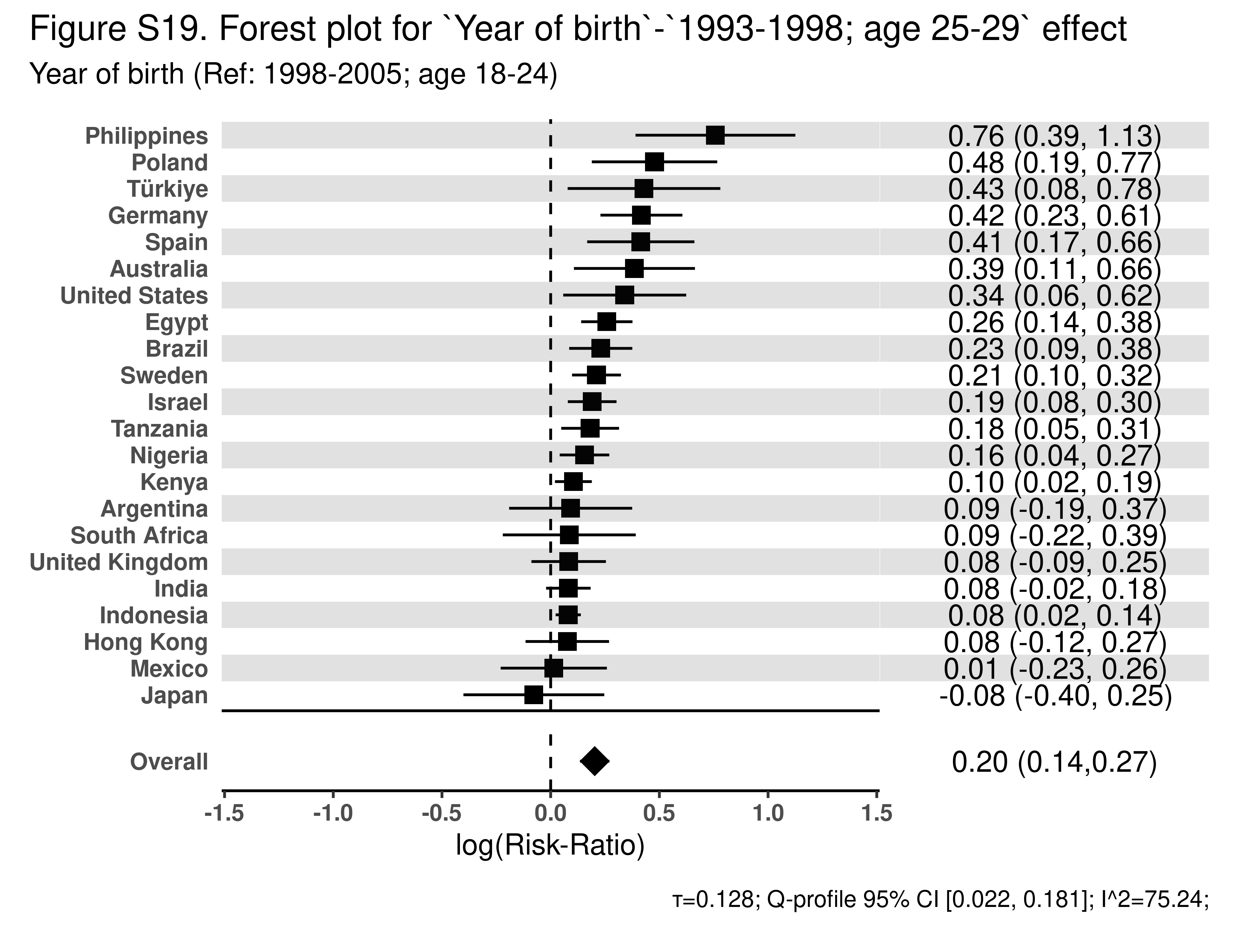 | 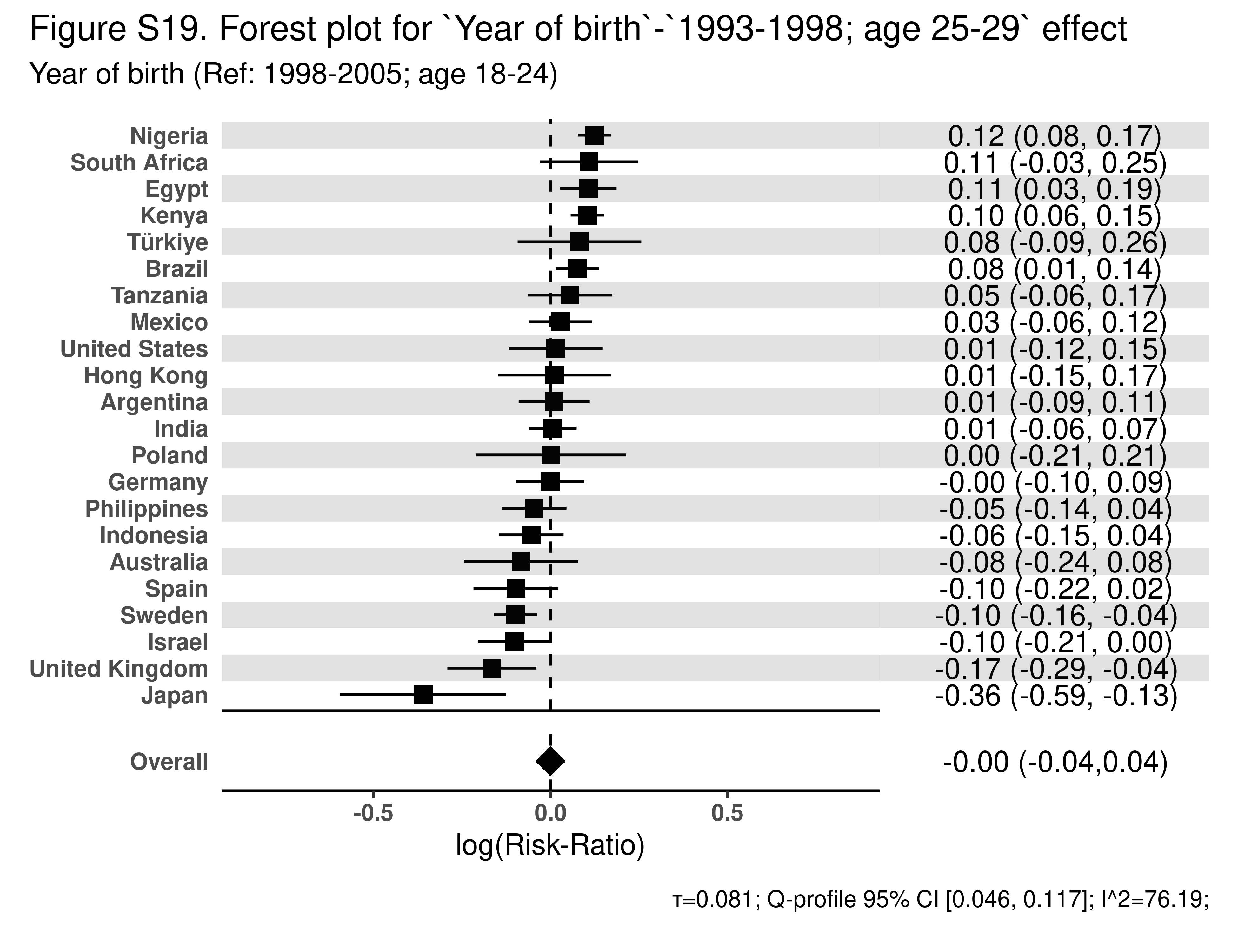 |  |
| ***Figure S20. Forest plot for ‘Year of birth’ – ‘1983-1993; age 30-39’ effect*** | 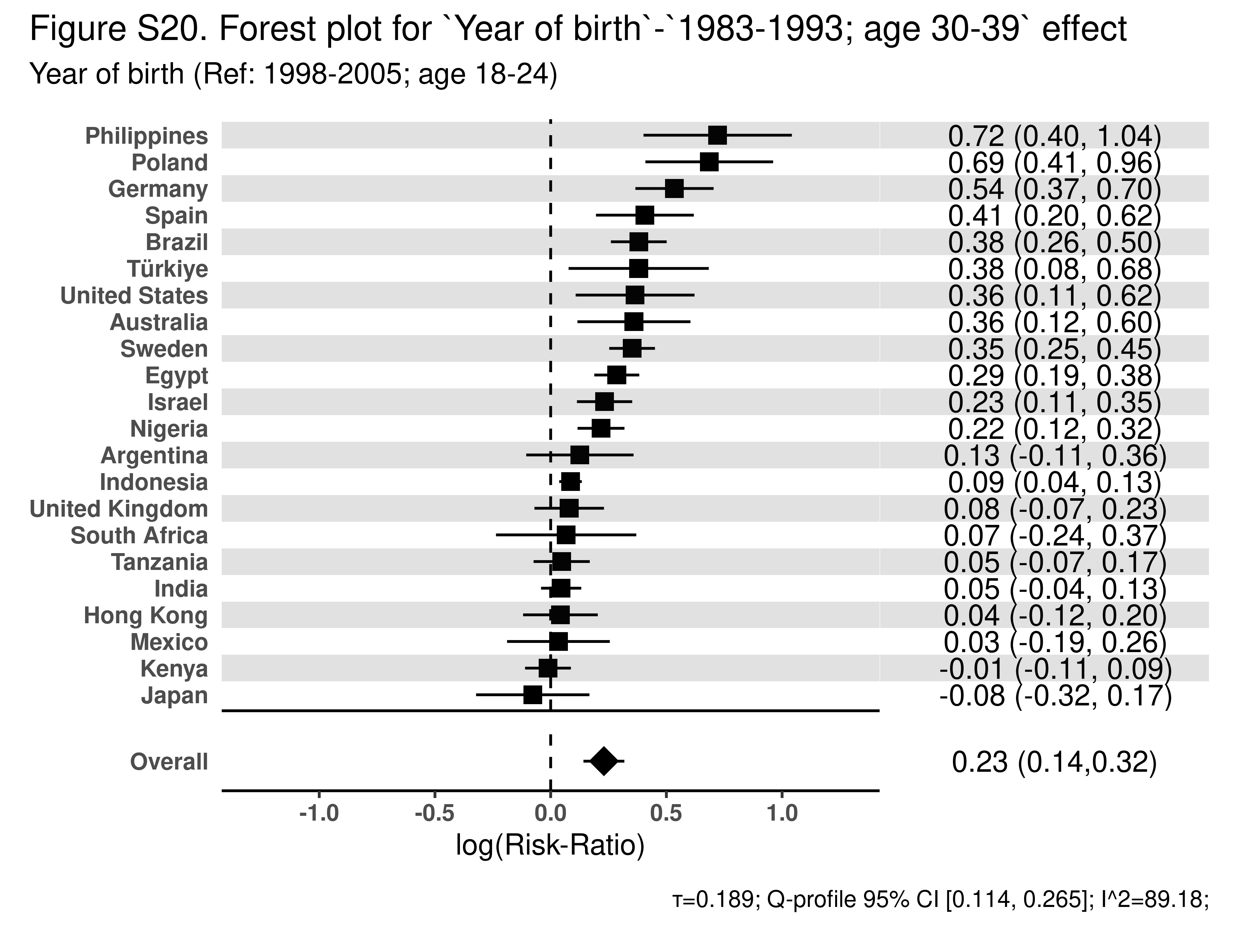 | 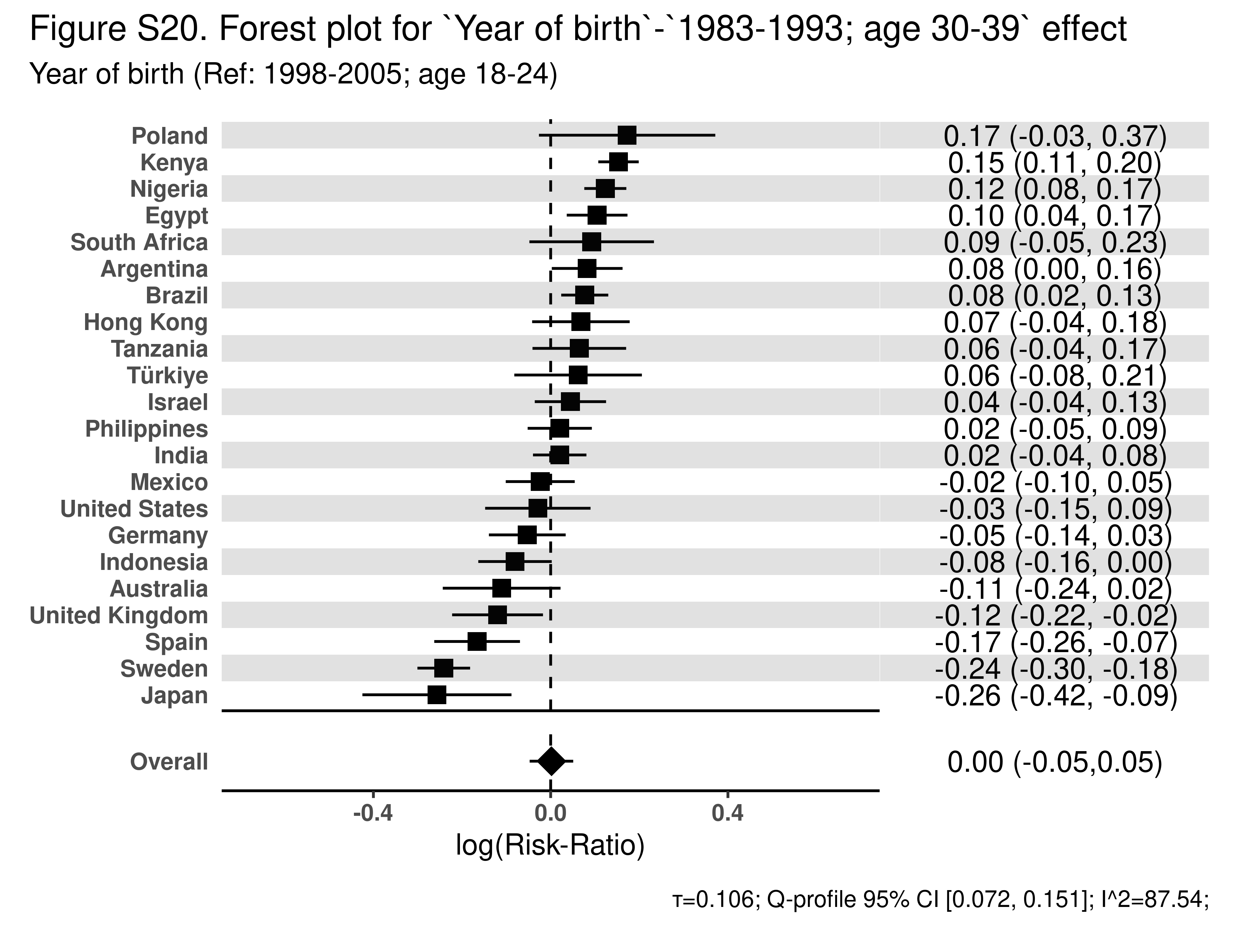 |  |
| ***Figure S21. Forest plot for ‘Year of birth’ – ‘1973-1983; age 40-49’ effect*** | 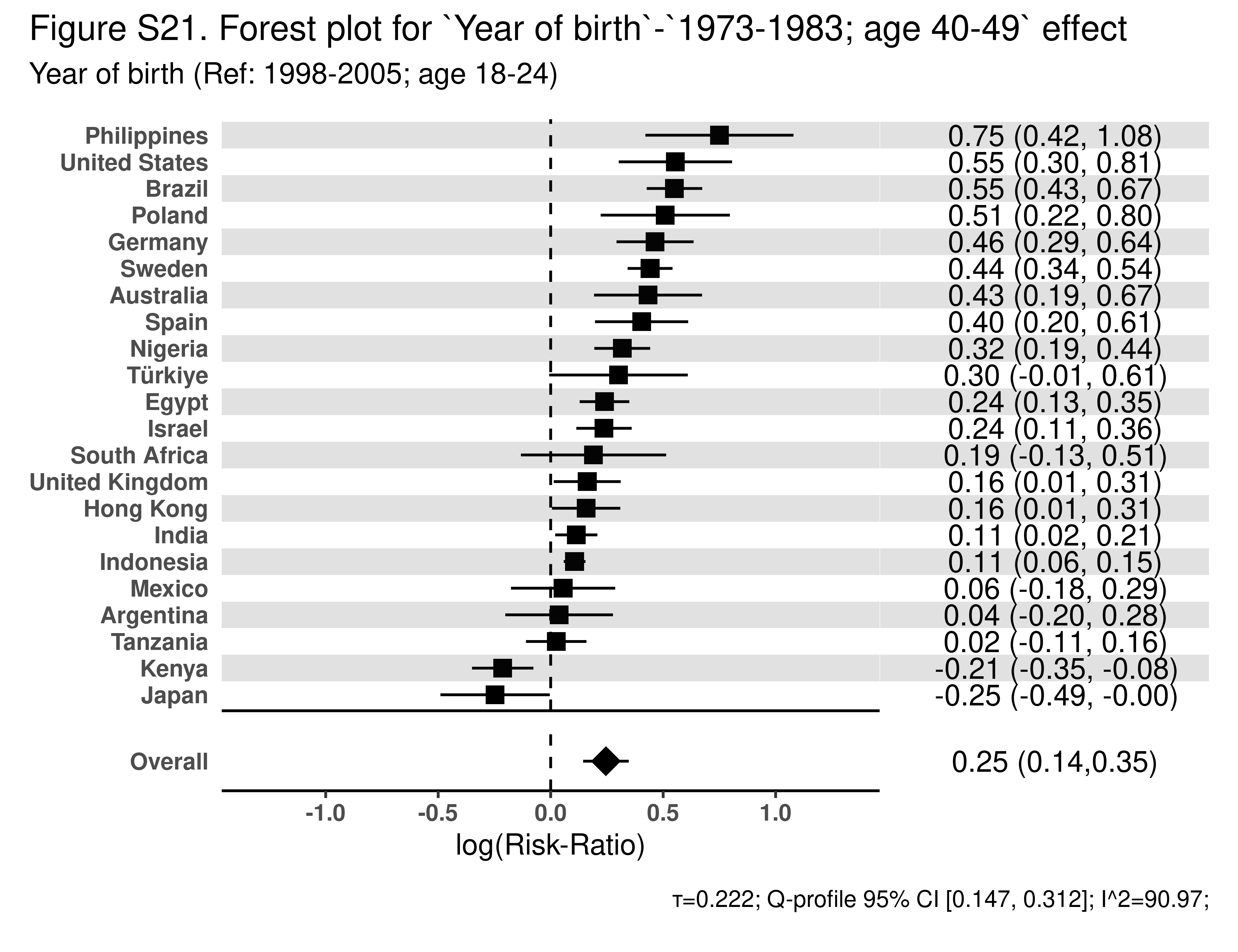 | 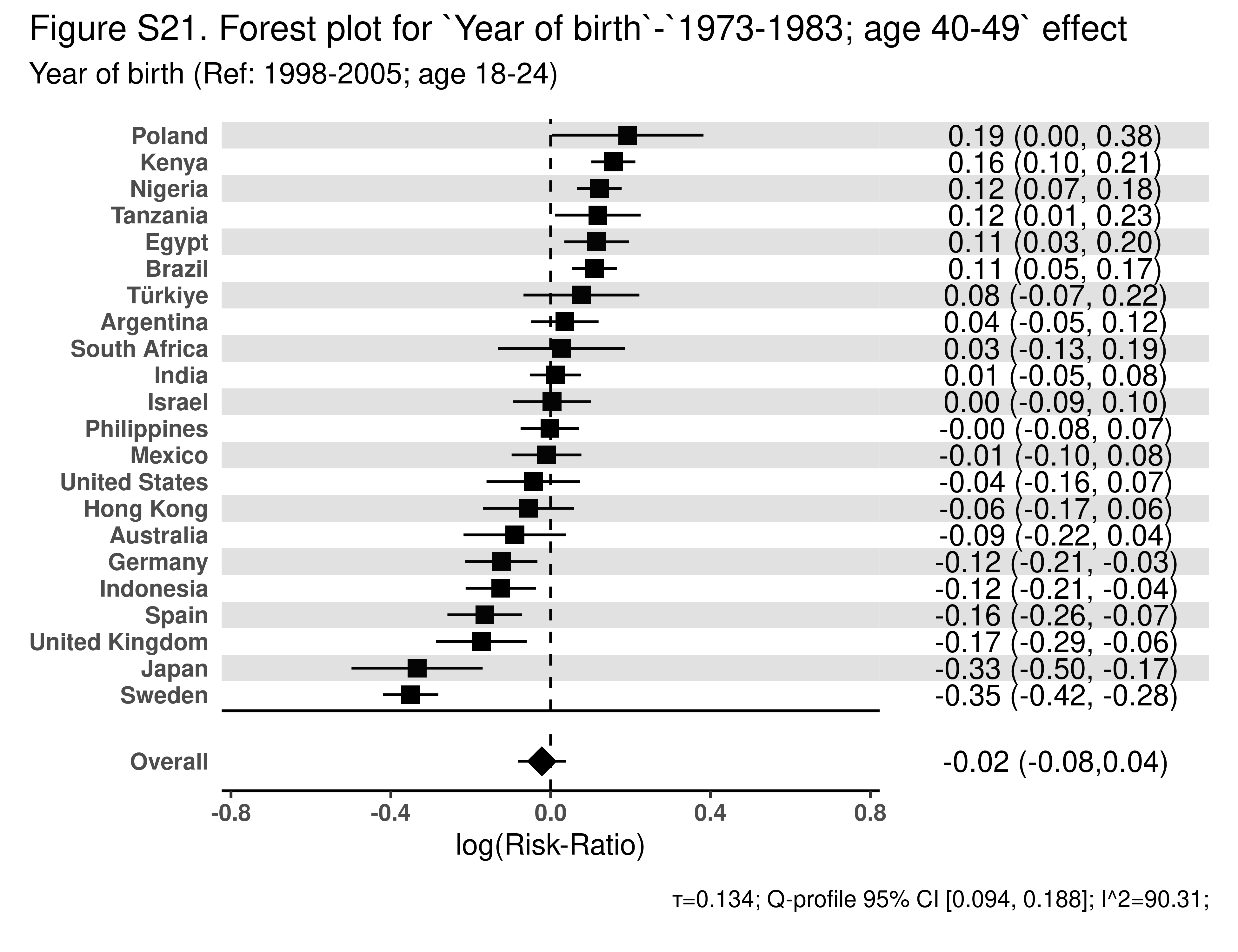 |  |
| ***Figure S22. Forest plot for ‘Year of birth’ – ‘1963-1973; age 50-59’ effect*** | 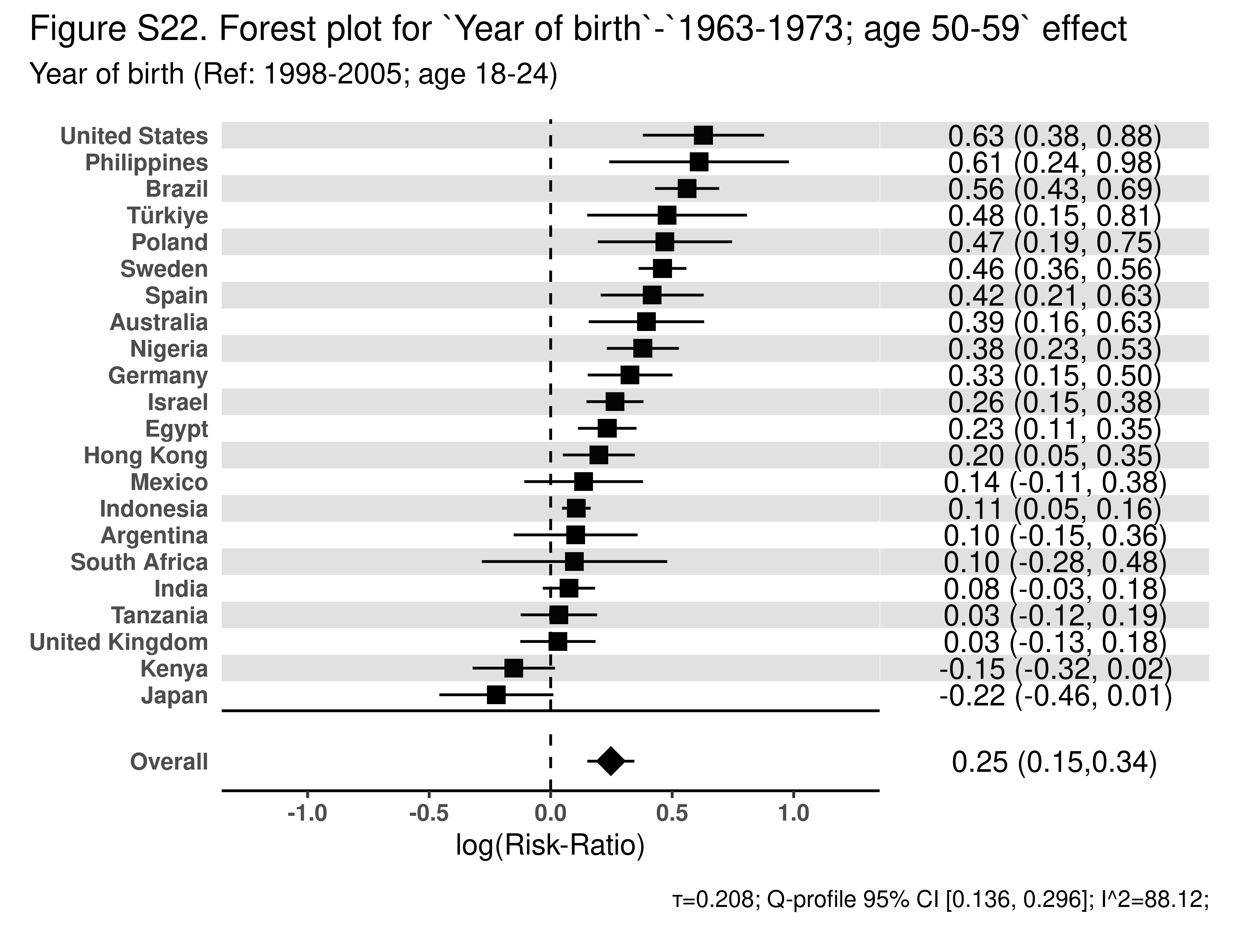 | 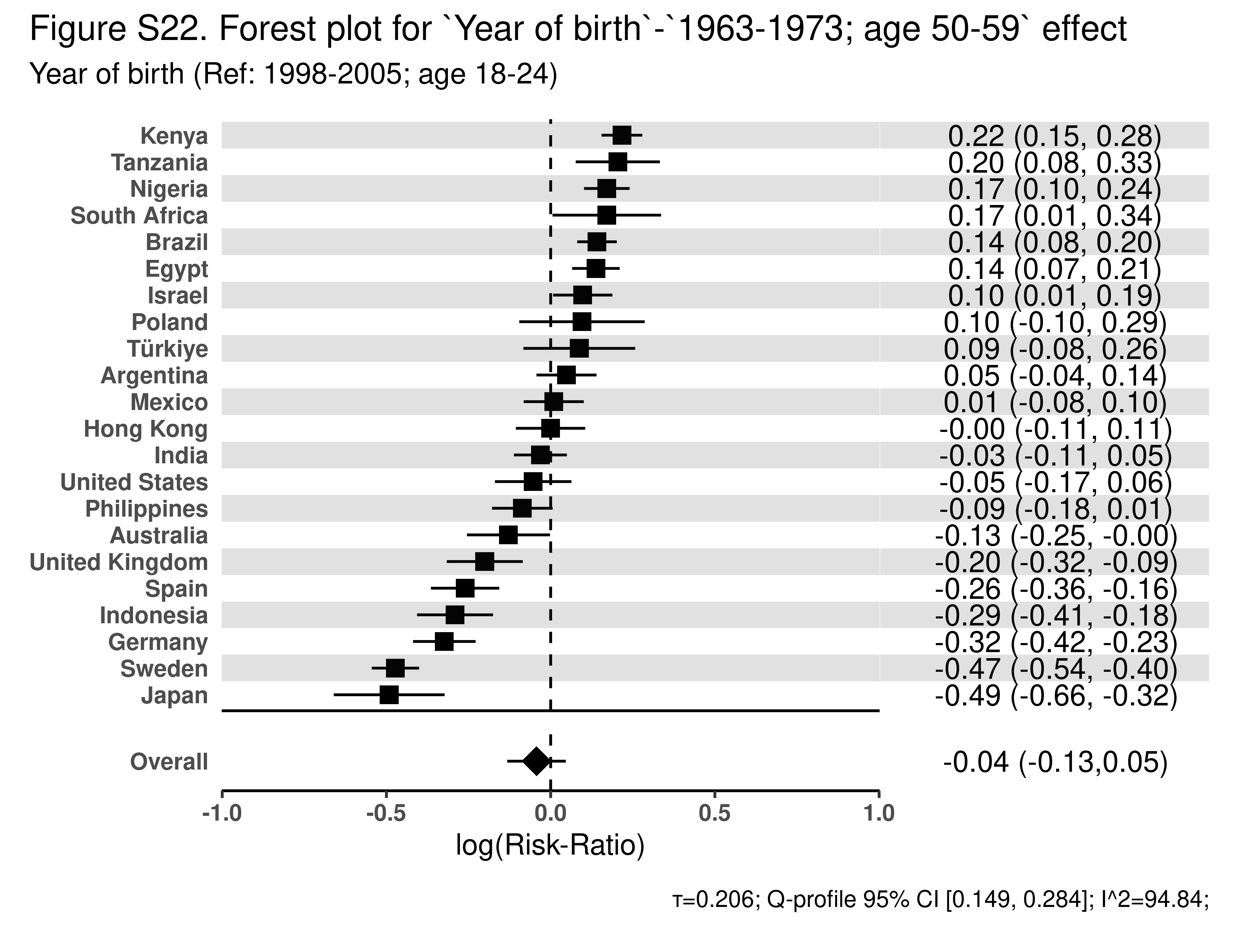 |  |
| ***Figure S23. Forest plot for ‘Year of birth’ – ‘1953-1963; age 60-69’ effect*** | 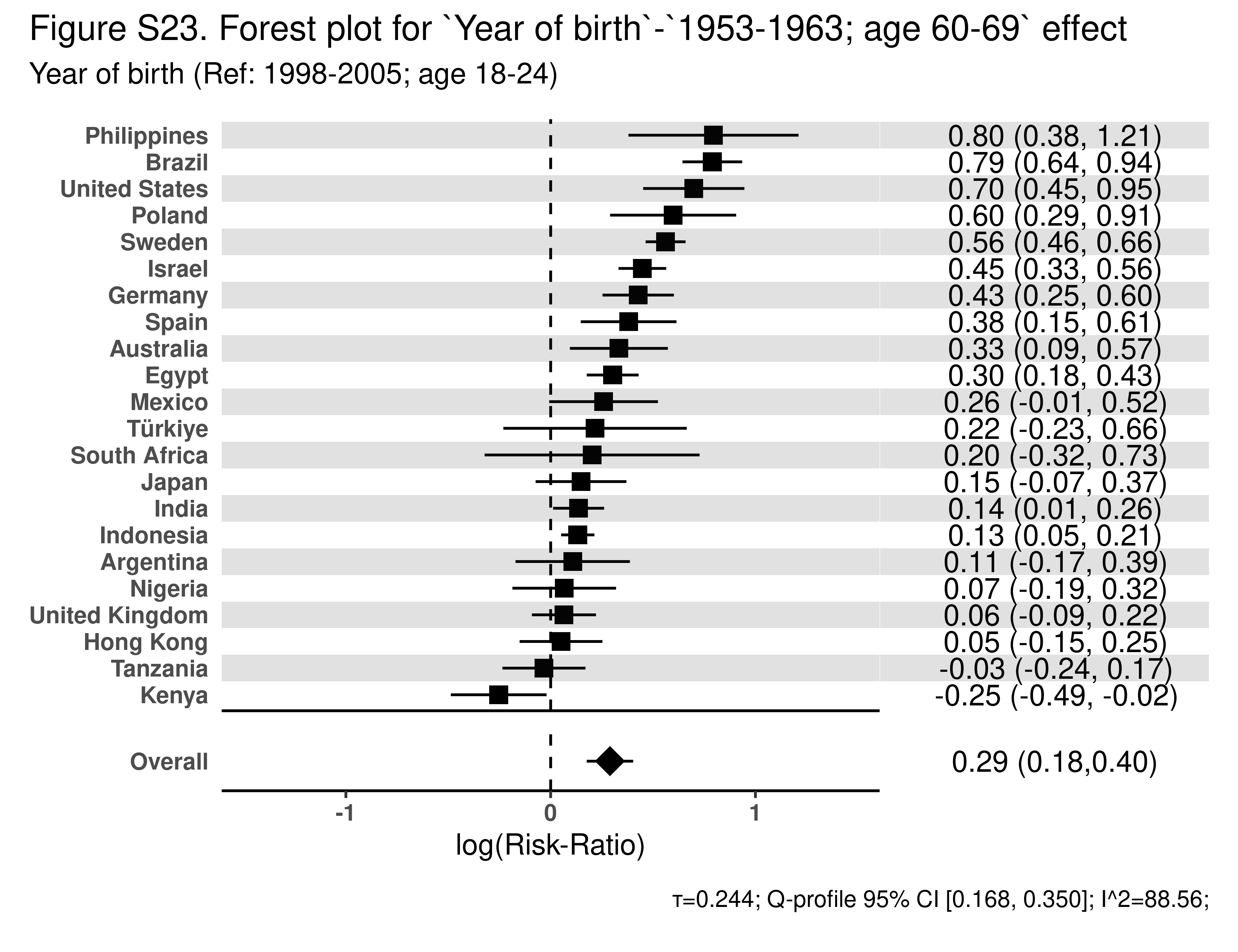 | 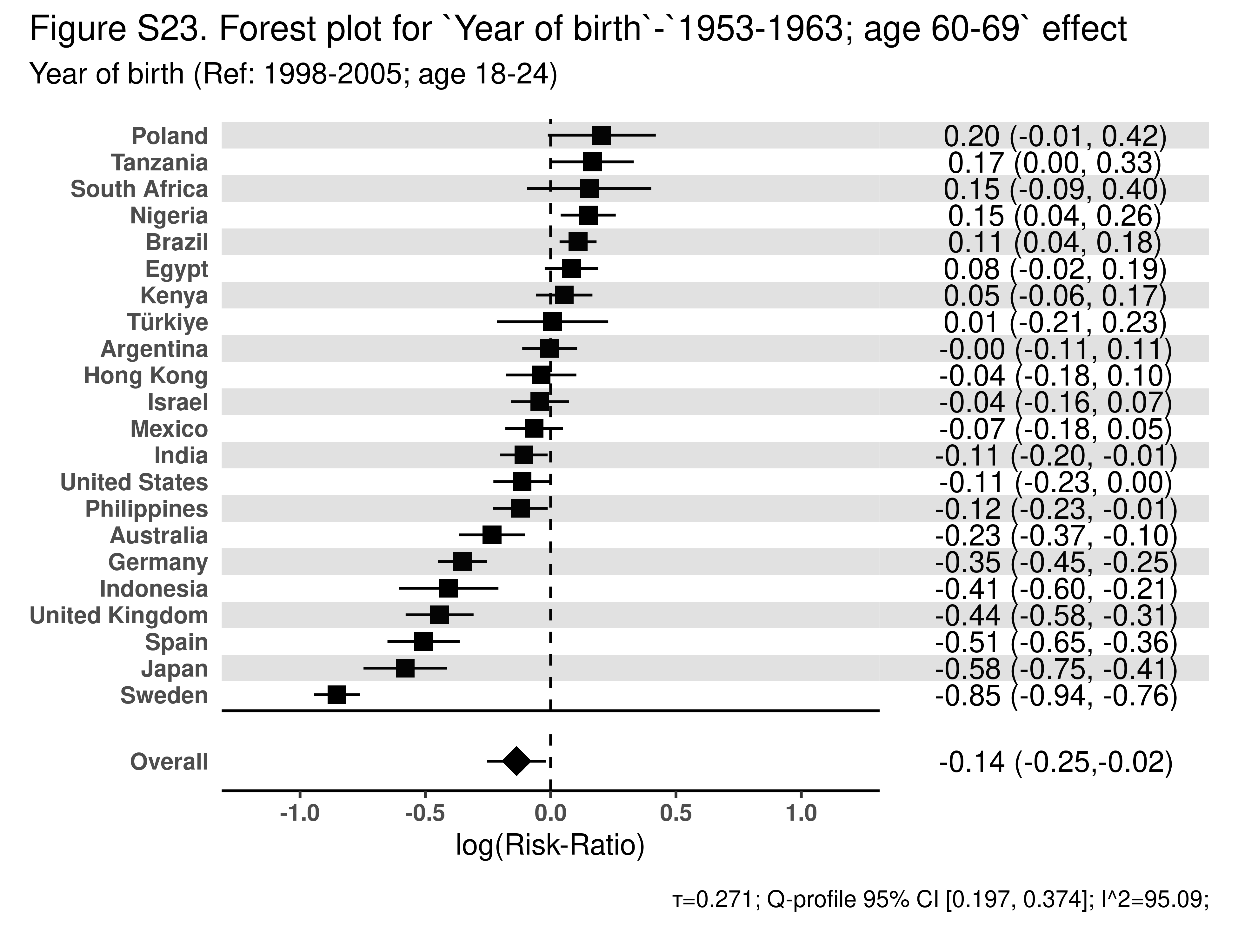 |  |
| ***Figure S24. Forest plot for ‘Year of birth’ – ‘1943-1953; age 70-79’ effect*** | 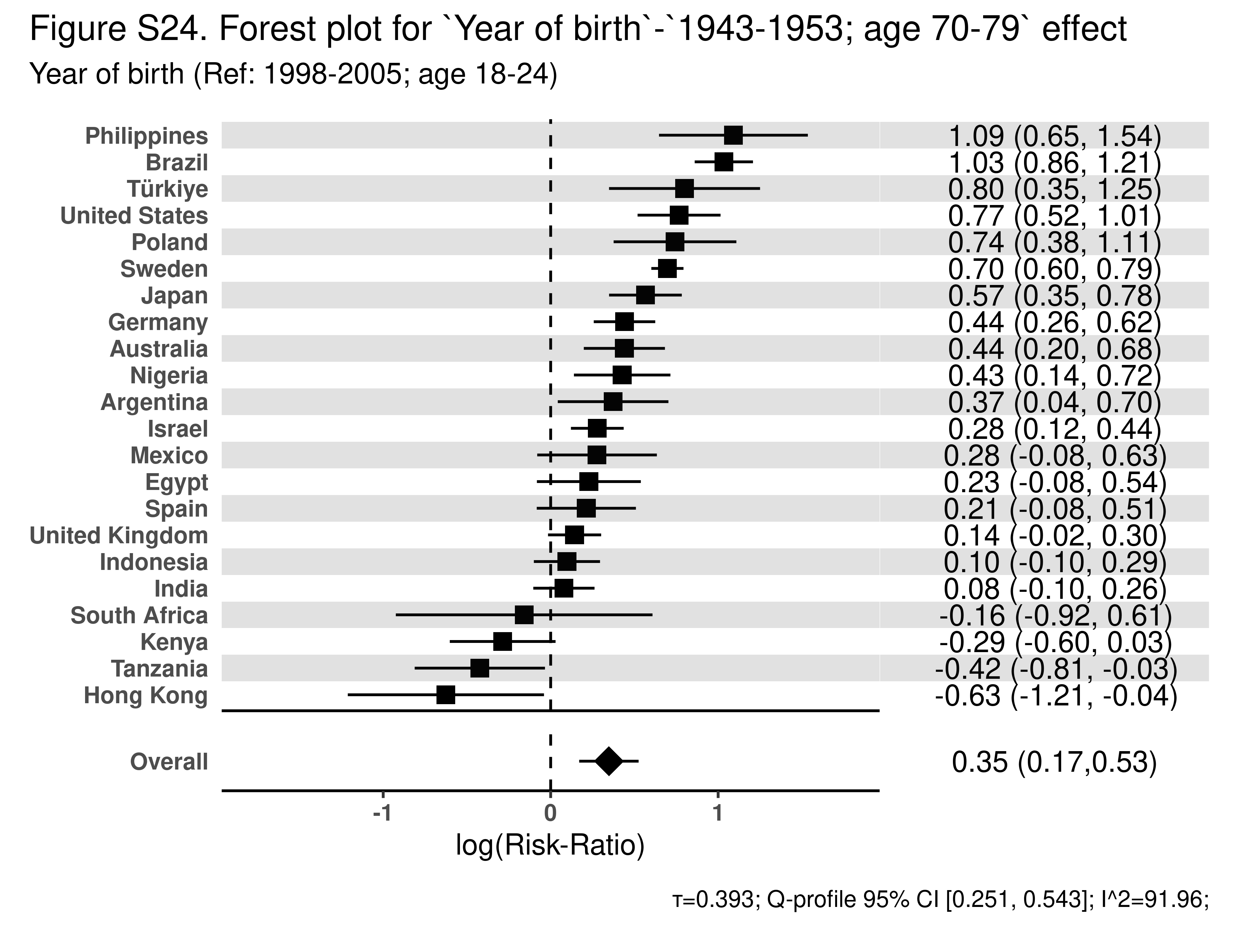 | 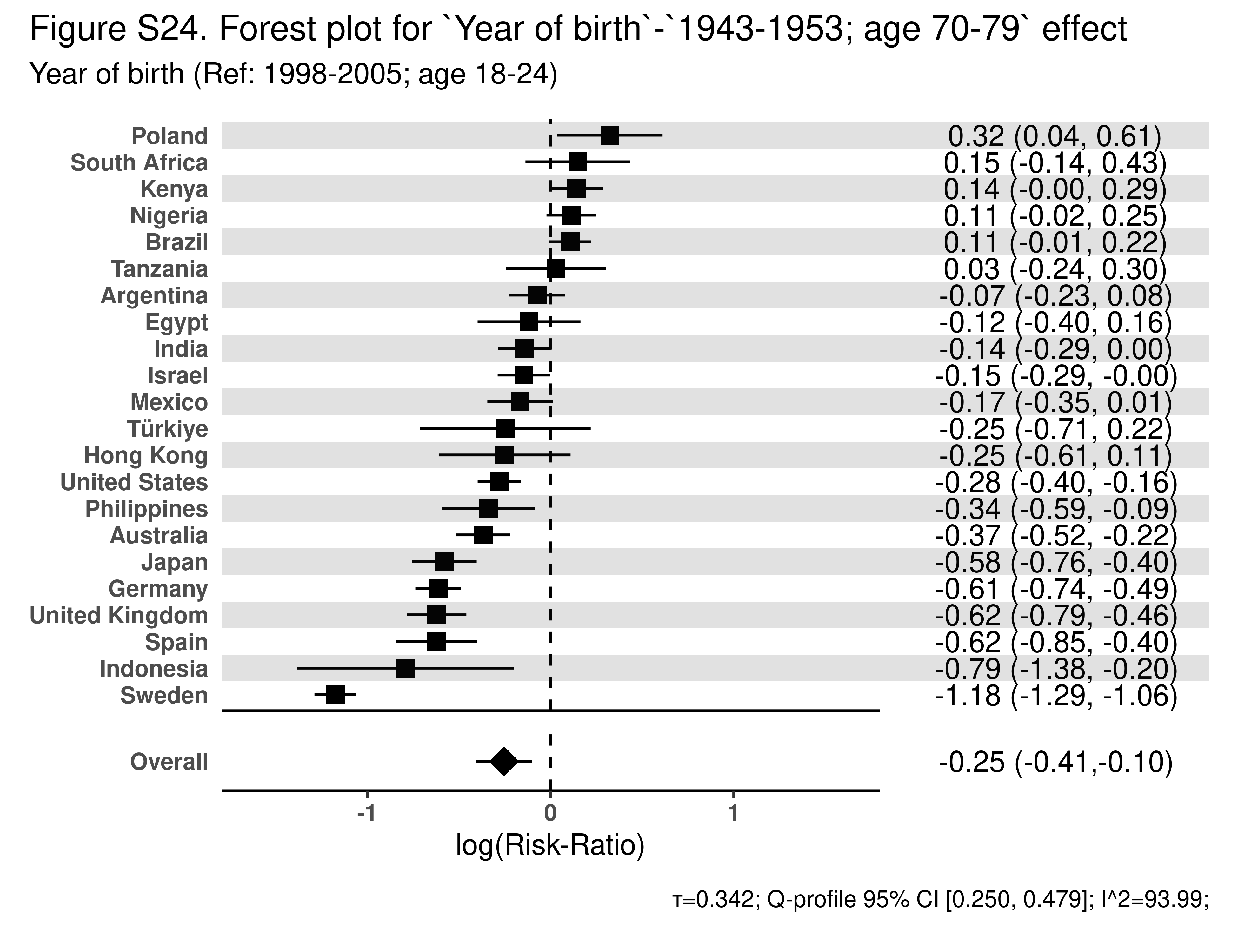 |  |
| ***Figure S25. Forest plot for ‘Year of birth’ – ‘1943 or earlier; age 80+’ effect*** | 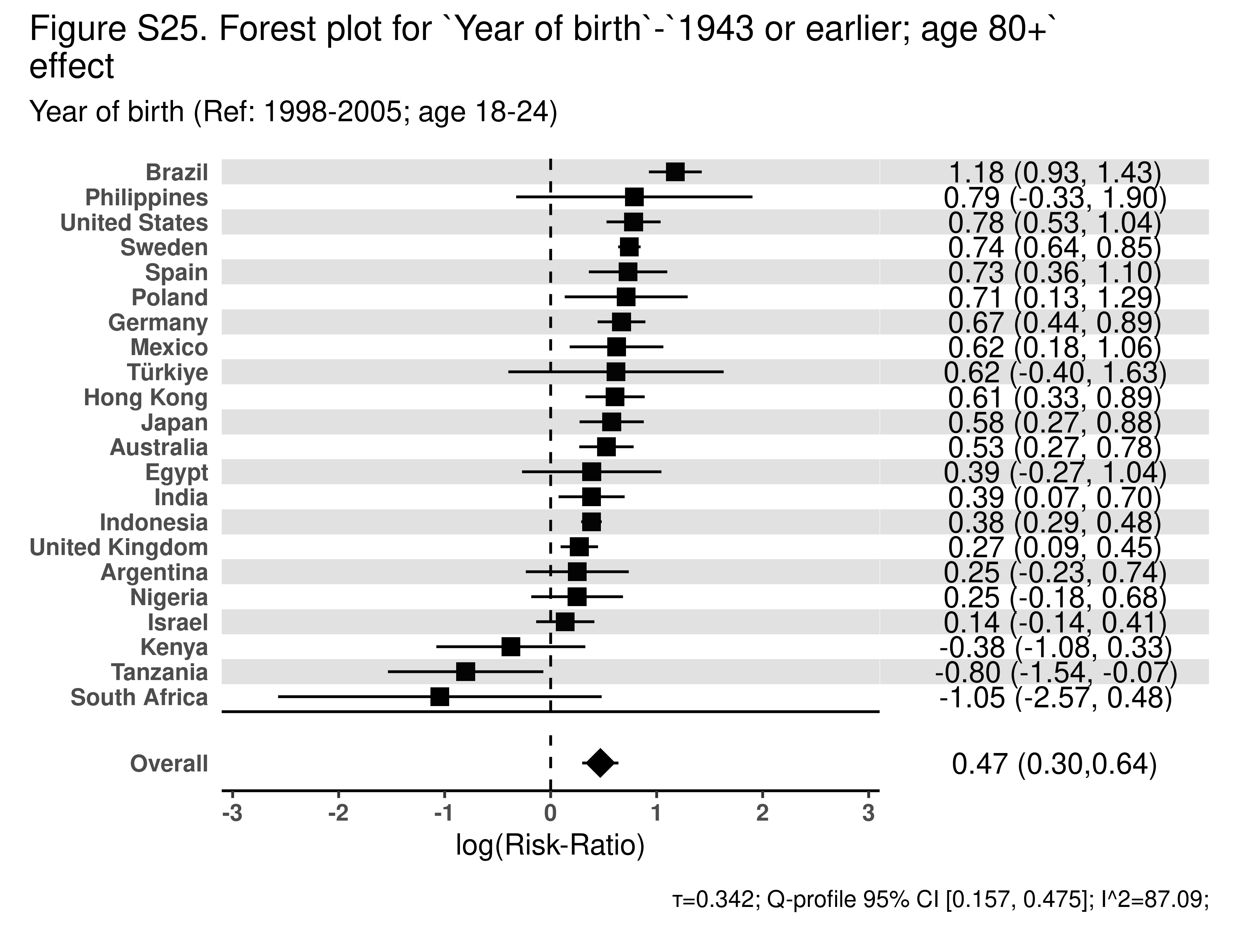 | 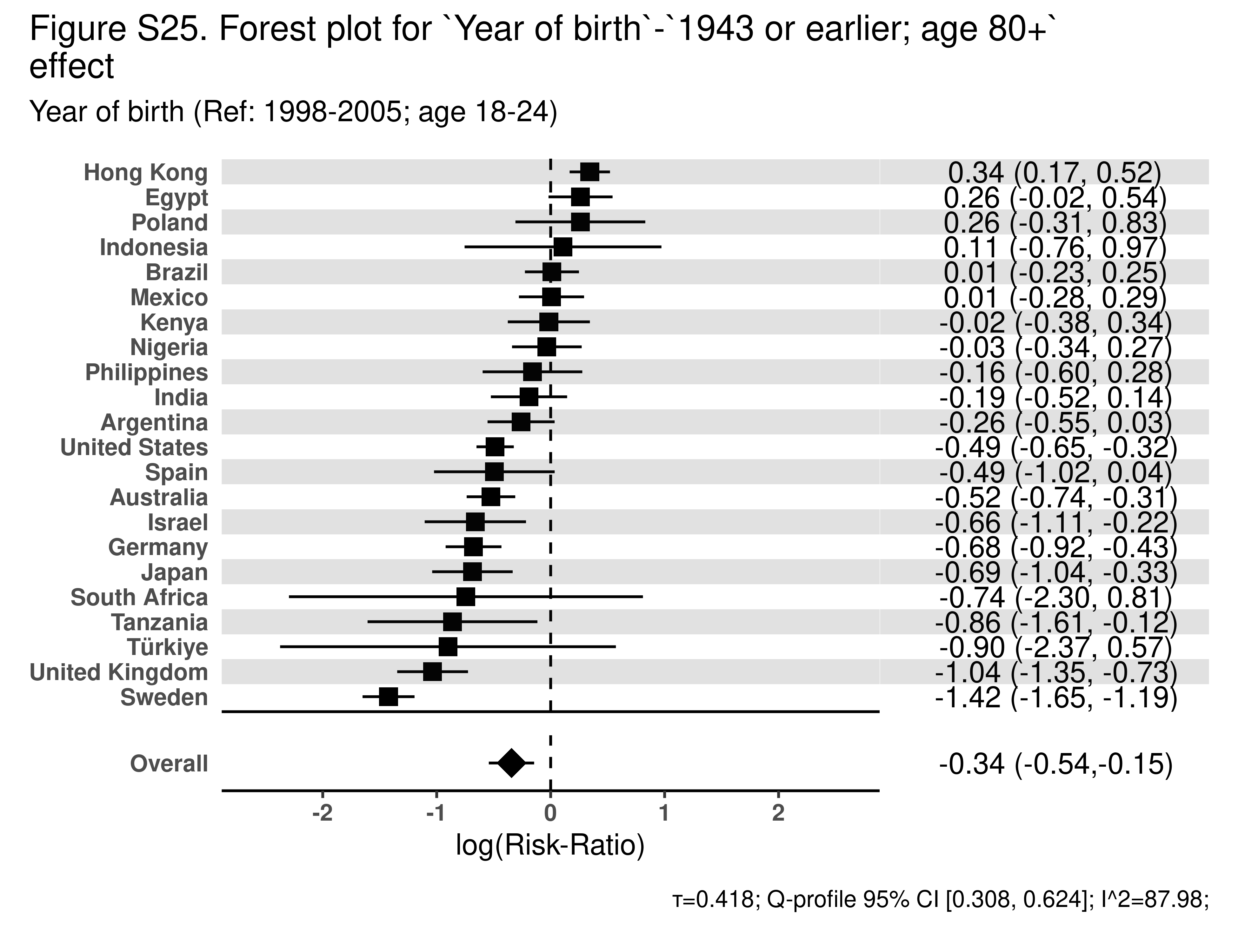 |  |
| ***Figure S26. Forest plot for ‘Gender’ – ‘Female’ effect*** | 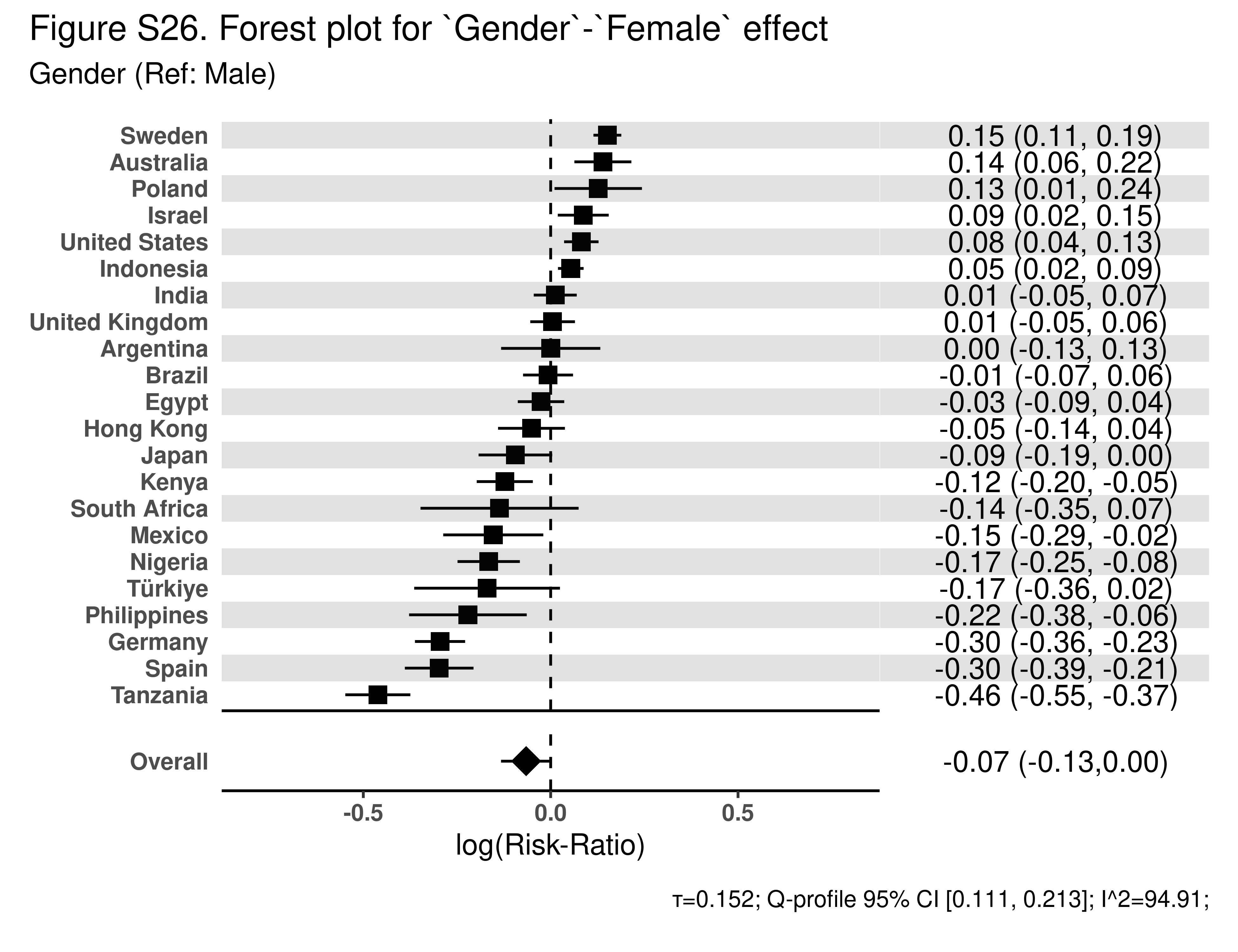 | 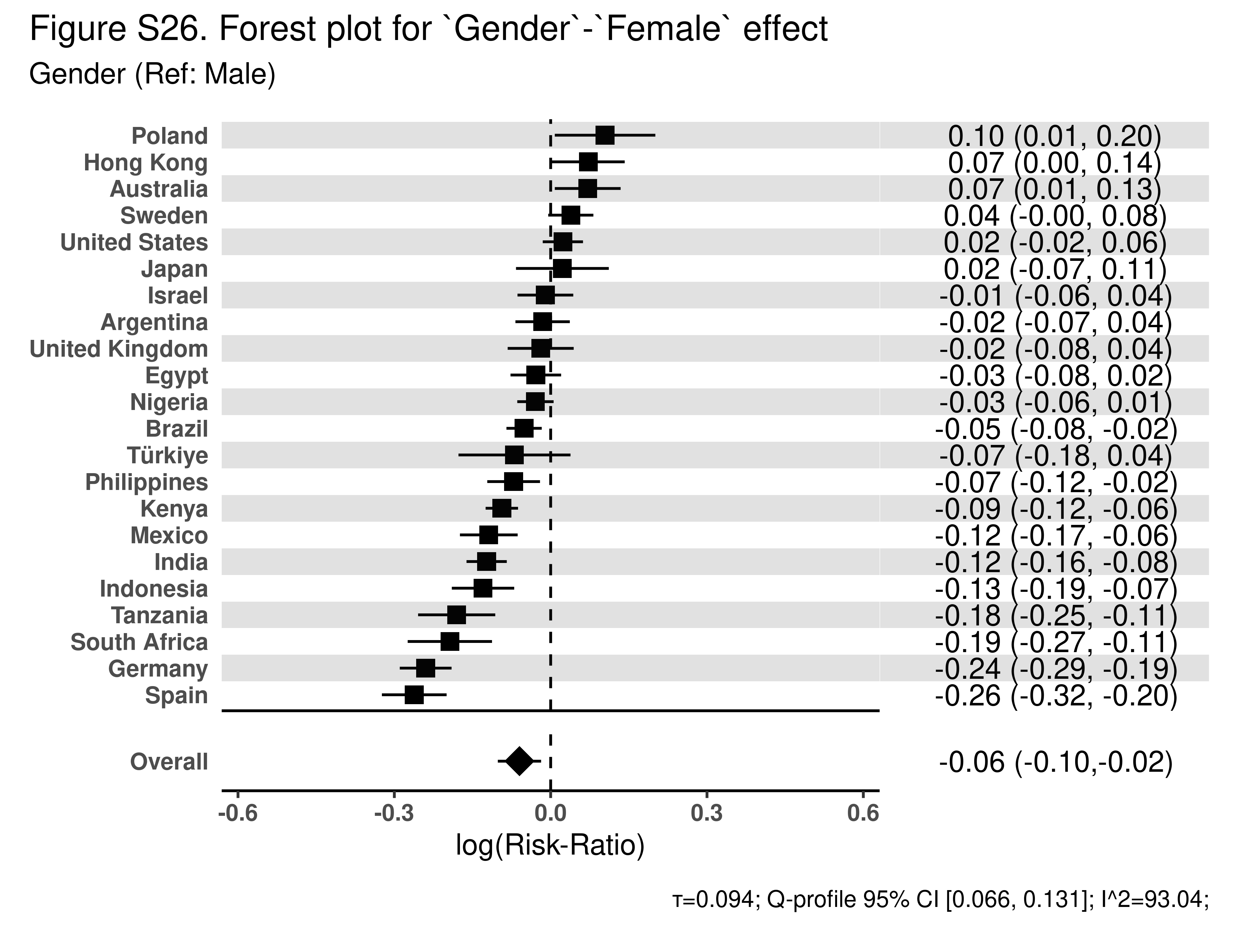 |  |
| ***Figure S27. Forest plot for ‘Gender’ – ‘Other’ effect*** | 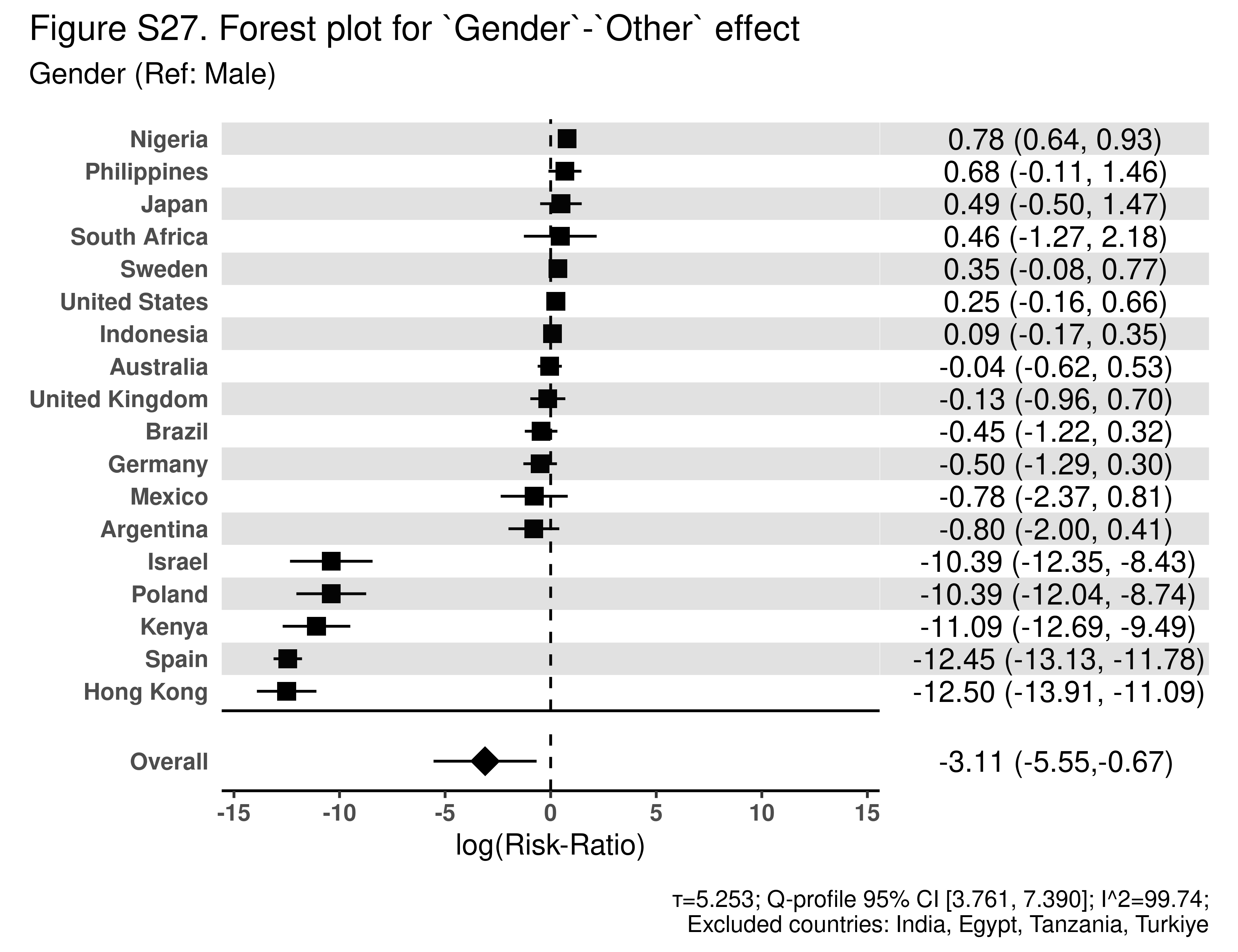 | 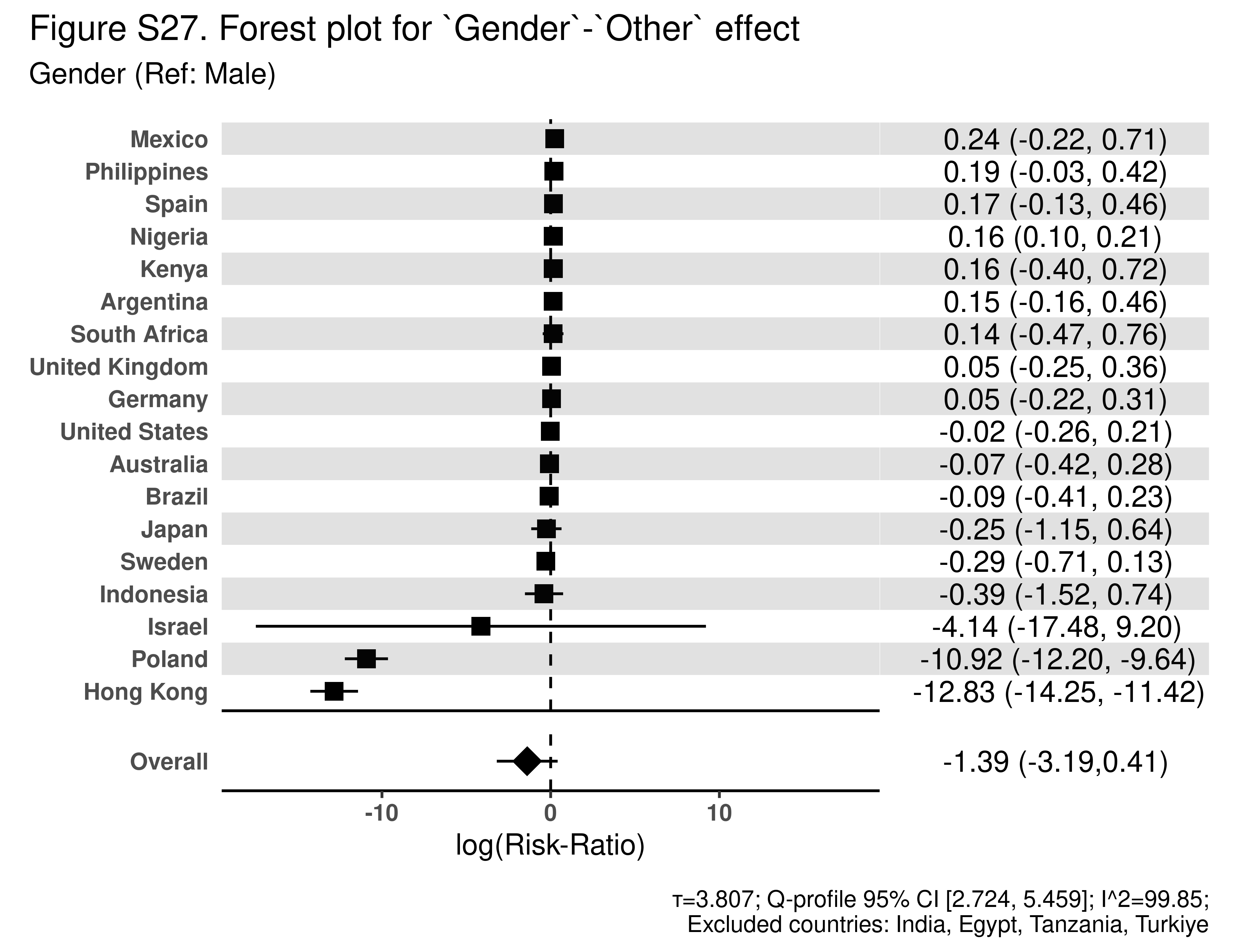 |  |
